# Late-Stage N‑Alkenylative Modifications of Indolic Scaffolds with Propiolates: Toward Bisconjugation and Macrocyclization

**DOI:** 10.1021/acs.orglett.5c01162

**Published:** 2025-05-14

**Authors:** Xiaoye Chen, Chi-Ming Au, Pengyuan Fang, Yunsheng Xue, Ken Cham-Fai Leung, Wai-Lun Chan

**Affiliations:** † The International Joint Institute of Tianjin University−National University of Singapore in Fuzhou, 12605 Tianjin University, Tianjin 300072, China; ‡ Department of Chemistry, National University of Singapore, 3 Science Drive, Singapore 117543; § Department of Chemistry, 26679 Hong Kong Baptist University, Kowloon Tong, Kowloon 999077, Hong Kong SAR, China; ∥ Jiangsu Key Laboratory of New Drug Research and Clinical Pharmacy, School of Pharmacy, 38044 Xuzhou Medical University, No. 209, Tongshan Road, Xuzhou, Jiangsu 221004, China

## Abstract

A facile, mild, and scalable late-stage N-alkenylative modification strategy is introduced on 1*H*-indoles, 9*H*-carbazoles, and their structural derivatives and analogues, including alkaloids, bioactive agents, and tryptophan motifs, via chemo- and regioselective phosphine-mediated propiolate hydroamination. Saliently, through this protocol, bisconjugation and macrocyclization on (bis)­indolic scaffolds can also be accomplished, with the installation of new α,β-unsaturated ester handles for potential further versatile synthetic manipulations.

## Graphical abstract

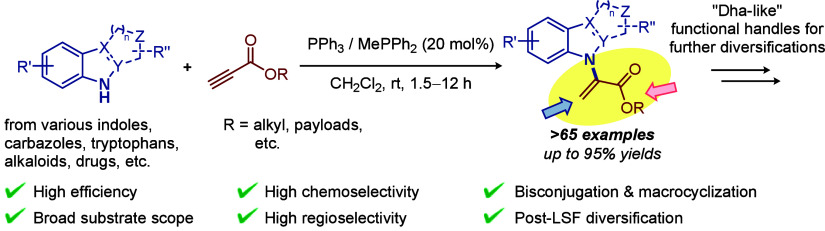

The recent renaissance of chimeric and macrocyclic compounds in medicinal chemistry has led to an overwhelming revolution of novel paradigm shifts and megatrends in drug discovery and development,^1^ in which more unprecedented practical synthetic methodologies for bisconjugation and macrocyclization are pressingly demanded.^2^ Indoles and their structurally related N-heterocycles (e.g., carbazoles and oxindoles) are among the most prevalent and privileged scaffolds in bioactive natural products, pharmaceuticals, and agrochemicals;^3^ indole-containing amino acid tryptophan (Trp) serves not merely as the building block of peptides and proteins, yet also the biosynthetic precursors for a few neurotransmitters and hormones.^4^ To chemically recruit indole-based alkaloids/small molecules or tryptophan motifs as carriers or payloads, time- and resource-intensive (*de novo*) total syntheses with unnatural or prefunctionalized feedstocks have to be implemented to install new ligation sites (e.g., carboxylic acids for esterifications and/or amidations, azides and/or terminal alkynes for click reactions, α,β-unsaturated ketones for conjugate additions, and halides for S_N_2/cross-coupling), which are extremely inefficient and challenging.^5^ In essence, nature has widely and wisely employed various indoles as versatile platforms to construct very complex three-dimensional alkaloid architectures and (poly)­macrocyclic peptides,^6^ which are still daunting inimitable tasks for synthetic chemists only with the conventional toolbox; therefore, developing simpler mild and efficient late-stage functionalization (LSF) strategies^7^ for indolic scaffolds is highly impressive and impactful.

Given the concerted global endeavor and dedication over the recent decades, a kaleidoscope of functionalization strategies on indoles,^8^ at different positions (C2–C7), have been elegantly documented, in highly efficient and selective fashions. Despite this, most of the literature-reported protocols would entail either (i) the use of specific directing protective groups, (ii) functional handles preinstalled, (iii) the use of special/strong reagents, or (iv) relatively harsh conditions, which would not be compatible with most delicate structures of indolic natural products and drugs (where certain C–H positions might not be available), not to mention peptides and proteins. Notably, indolic scaffolds with a free N–H group account for a significant reservoir of pharmaceutically important chemotypes (Figure 1a). Targeted functionalizations of indoles and their derivatives and analogues at the N–H position can provide a potential, highly sought-after “universal protocol” to their multifaceted modifications. To this end, with respect to the catalytic N-alkylations and N-arylations of indoles, indolines, and carbazoles, many groups had considerably contributed to this field.^9^ It should be noted that the C3/N1 chemoselectivity control of such reactions on simple indoles with the same electrophiles is always arduous (Figure 1b). However, translational LSF applications of most of the works would still be either inapplicable or unknown until very recently.^10^ Most LSF on indolic scaffolds are executed on “unprotected” tryptophan and peptides and proteins containing it, with their efficiency, selectivities, and compatibility being remarkable.^11^ Compared with the simple indole, the N–H group of tryptophan’s indole has far lower nucleophilicity (Trp-indolic NH < Lys or N-terminal NH_2_ < Cys-SH) and weaker basicity (Trp < His < Cys < N-terminus < Lys < Arg), which is more challenging for LSF. For instance, Lindner reported a selective condensation reaction between tryptophan and malondialdehyde;^12^ Studer and Hu illustrated two metal-free photoinduced selective modification methods on the tryptophan-based motifs.^13^ Krajcovicova developed a robust tryptophan-based multicomponent Petasis reaction approach for peptide LSF and stapling;^14^ Xu, Wang, and Sun innovated a tertiary amine-catalyzed N1-allylation on tryptophan with MBH adducts, enabling multipronged LSF.^15^

**Figure 1:**
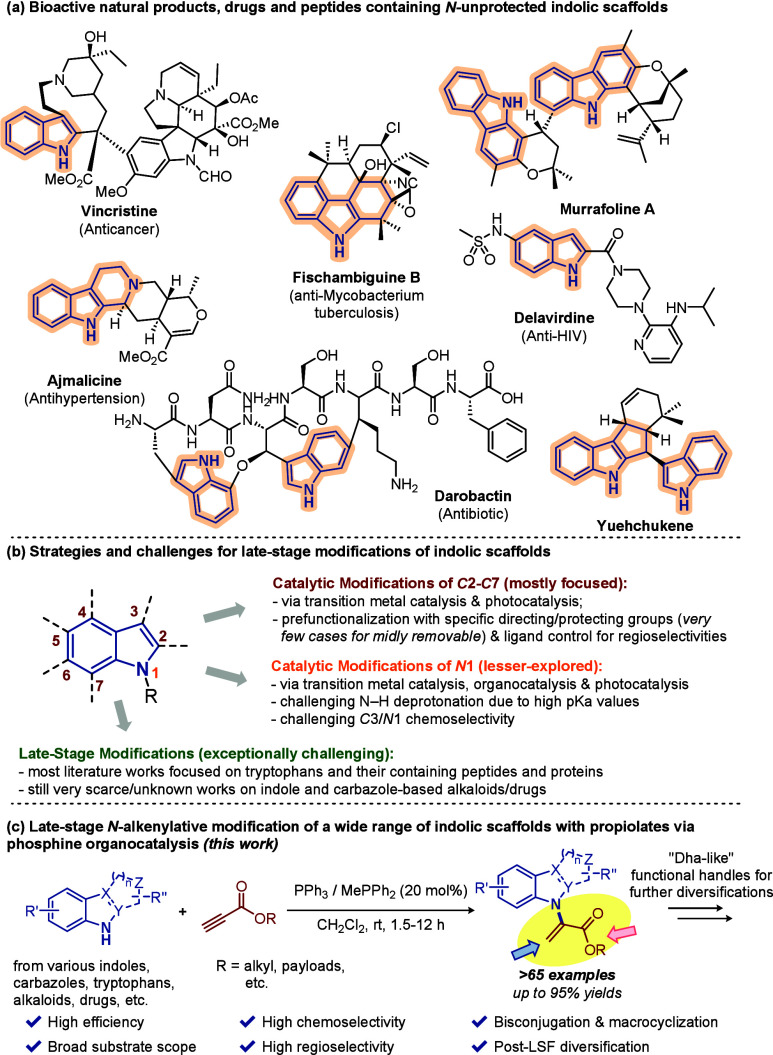
Bioactive N-undecorated indolic scaffolds and their late-stage modification strategies and challenges.

Based on our research expertise in phosphine organocatalysis, N-heterocyclic chemistry, supramolecularly assisted conjugation, and peptide macrocyclization,^16^ we envision that (1), mechanistically, a propiolate, upon phosphine activation, can generate *in situ* a strongly basic β-phosphonium allenolate intermediate, which can powerfully deprotonate indole, carbazole, and tryptophan’s N–H, and can then serve as a strong electrophile to complete the N-alkylation, where the reaction can pertain to LSF, and (2) the resulting alkenylated product possessing a new α,β-unsaturated dehydroalanine (Dha)-like acrylate moiety^17^ can be regarded as a many-sided late-stage-attached handle for synthetic manipulations. Early and relevant literature works focused on the comparative studies of such a reaction involving different electrophiles and Brønsted/Lewis bases for (*anti*) Markovniko adducts with very limited examples.^18^ Herein, we introduce a facile, mild, and scalable late-stage N-alkenylative modification strategy on 1*H*-indoles, 9*H*-carbazoles, and their structural derivatives and analogues (Figure 1c). Saliently, bisconjugation and macrocyclization on (bis)­indolic scaffolds, as well as subsequent cysteine ligation on the handle, were all successful, demonstrating a proof-of-concept phosphine–LSF approach on the indolic N–H bonds as a promising new linker conjugation protocol for potential translational applications.

With the optimized reaction conditions in hand after systematic screening (Table S3), we proceeded subsequently to establish the scope of propiolates, indoles, and their analogues (Table 1). To keep one variable constant (i.e., to avoid the keen C3 chemoselectivity issue), we intentionally used 3-methylindole to scrutinize the regioselectivity outcome of the scope of propiolates. Much to our delight, the dominant α-regioselectivity of the products (**4a**–**d**) was guaranteed; however, the susceptibility to the bulkiness of the ester groups was observed (yields decreased from ∼90% to ∼70%). We then screened and studied the scope of other different substituted indoles (**5**–**32** and **36**–**38**) with electron-withdrawing and -donating groups at different positions (C2–C7). To summarize, various functionalities at C3–C6 would all be tolerated, where electron-donating substituents often led to higher yields. Notably, for the cases of C7 (exerting steric hindrance on the phosphine-activated intermediate to approach N1), only halogen groups (**31** and **32**) were ordinarily feasible, while the methyl group (**36**) afforded inferior results. Similarly, considering the C2 position in the proximity of N1, the electron-withdrawing ester groups (**7** and **8**) would not only weaken the C3 nucleophilicity but also bolster the indolic N–H acidity to give high product yields. Electron-donating 2-methyl (unsuccessful substrate, not shown in Table 1), 2,3-dimethyl (**37**), and 2-phenyl (**38**) groups all failed to yield very clean reactions, as they activated the C3 nucleophiles while impairing the regioselective approach of the key intermediate owing to the steric effect. On the other hand, other structural analogues of indoles, e.g., benzimidazole (**33**), indazole (**34**), and isatin (**35**), belonging to other privileged scaffolds, were all found to function in this protocol.

**Table 1:**
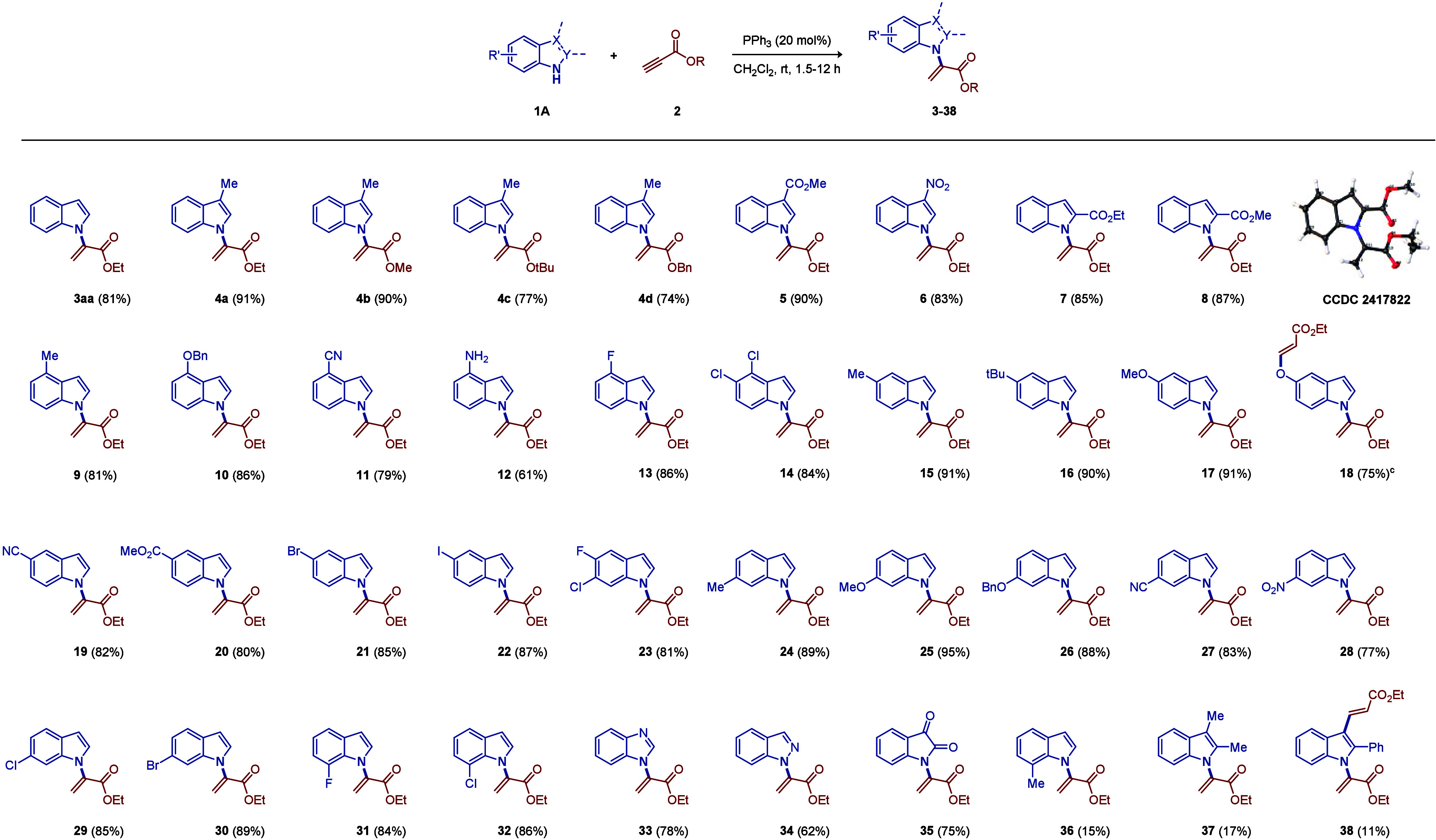
Scopes of Propiolates (**2**) and Indoles and Their Analogues (**1A**)­^a^,^b^

a Reaction conditions: **1A** (1 mmol), **2** (1.2 mmol), and the triphenylphosphine catalyst (20 mol %) in CH_2_Cl_2_ (1 mL) at room temperature.

b Yields refer to isolated products.

c With 2.1 mmol of **2** instead. Single-crystal X-ray crystallographic analysis of **8** was conducted, where the thermal ellipsoids are shown at the 50% probability level.

Encouraged by the above findings, we next strived to test the limit to investigate the scope of carbazoles and their analogues (Table 2). Based on nomenclature, the N–H group of unprotected carbazole is assigned as N9. Here, in general, diverse substituent patterns at C2–C4 (**39**–**46**) of, and even polyaromatic (**47**–**50**), carbazoles were well-tolerated. Their relatively lower product yields, compared with the indoles, were due to the poor solubility of carbazoles, and hence, significant product losses during column purification were inevitable. However, when it came to the C1 position, we attempted to use the 1-Br, 1-Cl, and 1-Me carbazoles (unsuccessful substrates, not shown in Table 2), but the former two halogenated always delivered mixtures of regioisomers in decent yields (∼40%), which were inseparable and decomposed easily; for the latter 1-Me, almost no reaction occurred even though it was heated to 90 °C. In brief, our protocol was found to be susceptible to steric indolic substrates. In other words, it did furnish a new, straightforward, and mild method for N-alkenylation on 9*H*-carbazoles and fused indolic structural analogues, such as tetrahydro-β-carboline (**51**), cyclohexa­[*b*]­indole (**52**), and cyclopenta­[*b*]­indole (**53**), with mediocre yields. Given that the majority of LSF literature concentrates on the tryptophan and the peptides and/or proteins containing it, we would like to surmount such a LSF challenge by employing our phosphine-catalyzed N-alkenylation reaction directly on cost-effective, commercially available indolic natural products, drugs, and tryptophan motifs (Table 3). Amazingly, the reactions proceeded smoothly with rutaecarpine (**54**, 73%), murrayafoline A (**55**, 66%), bisindolylmaleimide V (**56**, 64%), carprofen (**57**, 59% mixture), and lysergol (**58**, 44%). We then continued to apply our method to tryptophan Boc-Trp-OMe (**59**), with an excellent yield (93%), and tryptophan-containing dipeptide Boc-Trp-Phe-OMe (**60**), with a moderate yield (62%).

**Table 2:**
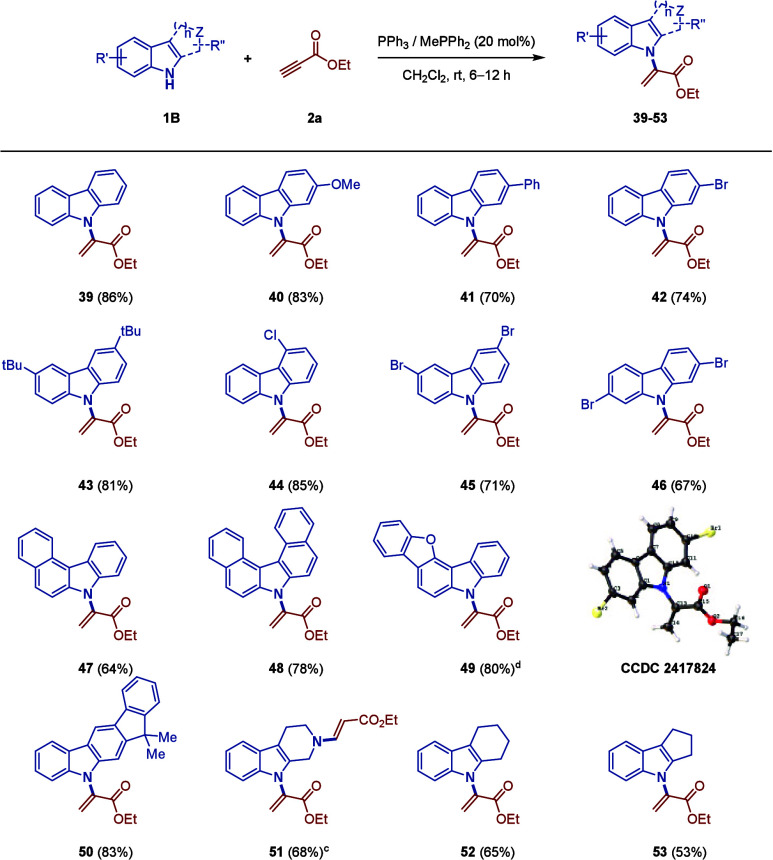
Scope of Carbazoles and Their Analogues (**1B**)­^a^,^b^

a Reaction conditions: **1B** (1 mmol), **2a** (1.2 mmol), and the triphenylphosphine catalyst (20 mol %) in CH_2_Cl_2_ (1 mL) at room temperature.

b Yields refer to isolated products.

c With 2.1 mmol of **2a** instead.

d With methyldiphenylphosphine instead. Single-crystal X-ray crystallographic analysis of **46** was conducted, where the thermal ellipsoids are shown at the 50% probability level.

**Table 3:**
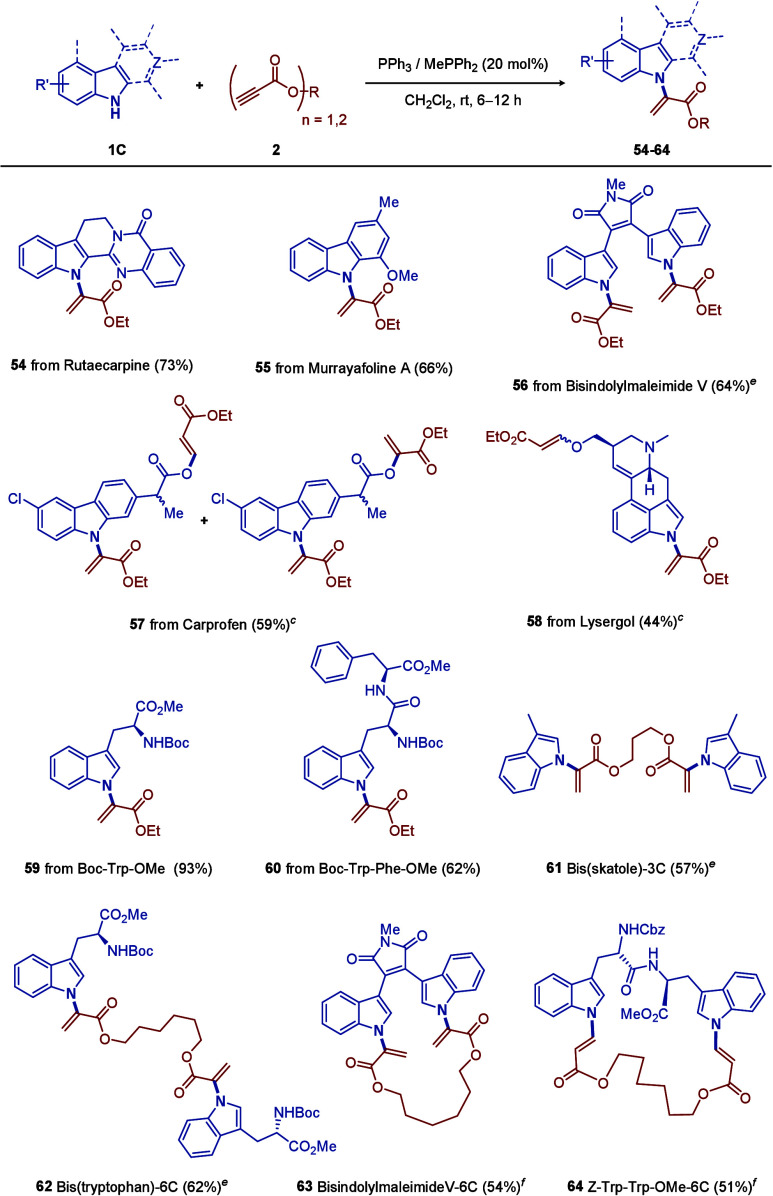
Scope of Bioactive Indolic Natural Products, Drugs, and Tryptophan Motifs (**1C**)­^a^,^b^

a Reaction conditions: **1C** (1 mmol), **2** (1.2 mmol), and the triphenylphosphine catalyst (20 mol %) in CH_2_Cl_2_ (1 mL) at room temperature.

b Yields refer to isolated products.

c With 2.1 mmol of **2** instead.

e With 2.2 mmol of **1C** instead.

f With 1.5 mmol of **1C** in CH_2_Cl_2_ (10 mL) instead. Single-crystal X-ray crystallographic analysis of **54** was conducted (CCDC 2419837 (see the Supporting Information)), where the thermal ellipsoids are shown at the 50% probability level.

To further substantiate our hypothesis that our methodology can facilely and valuably actualize challenging bisconjugation, we utilized commercially available propane-1,3-diyl dipropiolat and hexane-1,6-diyl dipropiolate as flexible hydrocarbon linkers, to react with two equivalent skatole (**61**) and Boc-Trp-OMe (**62**). The results were appealing; ∼60% yields of the double conjugates could be secured. To access macrocycles, we carried out the experiment between the 6C-linked dipropiolate and bisindolylmaleimide V (**63**, similar to LY379196) and Z-Trp-Trp-OMe (**64** and the β,β′-adduct were found), surprisingly discovering the reaction worked similarly well, both with ∼50% yields upon dilution. We scaled up a few pilot reactions (**4a′**, **5′**, and **59′**) to gram scale, and the reactions proceeded successfully with slightly decreased yields (**S1–S3**). Mechanistic studies were conducted experimentally and computationally. For other common substrates in phosphine catalysis, e.g., benzyl acrylate, benzyl 2,3-butadienoate, 3-butyn-2-one, dimethyl 2-butynedioate, etc., almost no or messy reactions were found (**S1–S4**), implying that it is the *in situ*-generated “basic-enough” β-phosphonium intermediate that can deprotonate the indolic N–H groups. We performed density functional theory (DFT) calculations on the second step (alkenylation). Four possible pathways were considered: Cα–N1, Cα–C2, Cα–C3, and Cβ–N1 pathways. The results of the calculated reaction Gibbs free energy (Δ_r_ *G*) and reaction enthalpy (Δ_r_ *H*) (Table S2 and Figure S1) indicate for the key transition states that the Δ_r_ *G* of the Cα–N1 pathway is just only 2.14 kcal/mol, significantly smaller than the other positive values (>10 kcal/mol), highlighting the preference of the Cα–N1 pathway. We then explored the kinetics of the reaction pathways. For the Cα–N1 pathway, the Gibbs free energy of activation (Δ*G* ^⧧^) was calculated to be 19.81 kcal/mol, significantly smaller than those of the other three pathways (>25 kcal/mol), indicating that the Cα–N1 pathway is kinetically favored, compared with the other three pathways. Natural population analysis and noncovalent interaction analysis were further performed. (i) The N1 atom in the deprotonated indole has the most negative charge of −0.67, followed by C3 (−0.41), indicating that position N1 has stronger nucleophilicity than position C3. On the other hand, the Cα site in IM1 is less negatively charged (−0.19), compared to Cβ (−0.49), implying the stronger electrophilicity of the Cα site. (ii) Compromised noncovalent interactions (mainly electrostatic interactions) and steric repulsion accounted for the Cα–N1 selectivity. All of these findings corroborated our proposed mechanistic cycle (**S1–S5**). Furthermore, product **59′** could be facilely post-LSF-diversified with Boc-Cys-OMe (**65**, 94%) via the Et_3_N-triggered thio-ene reaction, paving the way for peptide ligation, modification, and macrocyclization (**SI-3**).

In summary, we disclose a facile, mild, efficient, atom-economic, and upscalable late-stage N-alkenylative modification strategy on a wide range of indolic scaffolds at the recalcitrant N–H bonds with outstanding chemo- and regioselectivities. Further diversifications of the Dha-like products and life sciences studies of the bioactive agents and probes designed and developed thereof are currently underway via collaboration.

## Supplementary Material



## Data Availability

The data supporting the findings of this study are available in the article and its Supporting Information.

## Notes

General information, representative procedures, mechanistic studies, DFT calculations, characterization data, and NMR spectra (PDF)
