# Supplementary material for: Late-Stage N‑Alkenylative Modifications of Indolic Scaffolds with Propiolates: Toward Bisconjugation and Macrocyclization
Source: Org Lett. 2025 May 14;27(20):5081–6. doi: 10.1021/acs.orglett.5c01162 (PMC12105015; doi:10.1021/acs.orglett.5c01162)
Supplement: Supplementary file 1 [file ol5c01162_si_001.pdf]

Supporting Information

Late-Stage *N*-Alkenylative Modifications of Indolic Scaffolds with Propiolates: Towards Bisconjugation and Macrocyclization

Xiaoye Chen,[a,b] Chi-Ming Au,[b] Pengyuan Fang,[a] Yunsheng Xue,[d] Ken Cham-Fai Leung,*[c] and Wai-Lun Chan*[a,b]

Table of contents

A. General Information S2
B. Representative Procedures & Mechanistic Investigation S3
C. Reaction Development and Analytical Data S29
D. Crystal Structure of **8** S69
E. Crystal Structure of **46** S78
F. Crystal Structure of **54** S88
G. Copies of NMR Spectra (^{1}H, ^{13}C & ^{19}F) S117
H. References S186

A. General Information

Unless otherwise specified, the reagents and solvents in the experiments were all commercially available and employed without further purification. Thin-layer chromatography (TLC) was performed on silica gel plates (60F-254) using UV-light (254 & 365 nm). Flash column chromatography was conducted on silica gel (200–300 mesh). The ^{1}H, ^{13}C and ^{19}F NMR spectra were acquired on a Bruker spectrometer, operating at either 400 MHz or 500 MHz. The data are reported as follows: for ^{1}H NMR, chemical shifts were reported as parts per million (ppm) from tetramethylsilane (TMS) with the solvent as the internal standard ($CDCl_3$ δ 7.26 ppm and Acetone-d_6 δ 2.05 ppm), the multiplicity (s = singlet, d = doublet, t = triplet, q = quartet, m = multiplet or overlap of non-equivalent resonances), and the integration; for ^{13}C NMR, chemical shifts were reported in ppm referenced to TMS relative to carbon resonance ($CDCl_3$ δ 77.1 ppm and Acetone-d_6 δ 206.68 ppm & δ 29.92 ppm). Additionally, the coupling constants (*J*) were expressed in Hertz (Hz). High-resolution mass spectrometric (HRMS) analyses, reported as the mass-to-charge ratio (m/z), were performed using Agilent 6546 Q-TOF. The melting points were measured in open capillary tubes with a Cole-Parmer™ MP-250 series digital melting point apparatus, and were uncorrected. The single crystals were prepared via gas-phase diffusion of hexane into ethyl acetate solution, and were measured at low temperature (T = 100K) on a four circles goniometer Kappa geometry Bruker AXS D8 Venture equipped with a Photon III charge-integrating pixel array detector and using a Mo monochromatized (λ = 0.71073 Å) X-ray radiation source.

B. Representative Procedures & Mechanistic Investigation

1. General procedure for phosphine-catalyzed propiolate hydroamination with indolic scaffolds:

To a stirring solution of **1 indolic scaffolds** (1 mmol, 1.0 equiv.) and **triphenylphosphine** (20 mol%) in CH_2Cl_2 (1 ml, 1 M) at room temperature and pressure was added dropwise of **2 propiolates** (1.2 mmol, 1.2 equiv.). The colour of the reaction mixture changed from colourless to brown. Upon reaction completeness (monitored by TLC), the reaction mixture was directly purified by silica gel chromatography (hexane:ethyl acetate = 5:1 to 20:1) to afford the desired products (colourless oil, yellowish oil or white solid; *a few products would be light-sensitive).

2. General procedure for pilot scale-up reactions:

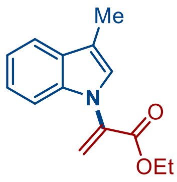

4a' (gram-scale = 85%)

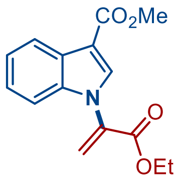

5' (gram-scale = 86%)

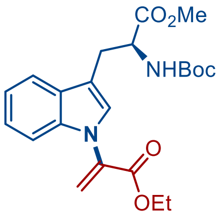

59' (gram-scale = 81%)

To a stirring solution of **1 indolic scaffolds** (5–10 mmol, 1.0 equiv.) (please refer to table S1) and **triphenylphosphine** (20 mol%) in CH_2Cl_2 (5–10 ml, 1 M) at room temperature and pressure was added dropwise of **2 propiolates** (6–12 mmol, 1.2 equiv.). The colour of the reaction mixture changed from colourless to brown. Upon reaction completeness (monitored by TLC), the reaction mixture was directly purified by silica gel chromatography (hexane:ethyl acetate = 8:1 to 15:1) to afford the desired products.

Table S1-Experimenta details of pilot scale-up reactions

	Amount of **indolic scaffolds**	Amount of **propiolates**	Solvent volumes	Yields	Eluent (hexane:ethyl acetate)
**4a´**	1.31g, 10 mmol	0.98 ml, 12 mmol	10 ml	1.95g, 85%	15:1
**5´**	1.40g, 8 mmol	0.94 ml, 9.6 mmol	8 ml	1.88g, 86%	15:1
**59´**	1.59g, 5 mmol	0.6 ml, 6 mmol	5 ml	1.70g, 81%	8:1

3. Procedure for post-LSF based-catalyzed thio-ene reactiom between *N*-alkenylated Boc-Trp-OMe (59) and Boc-Cys-OMe

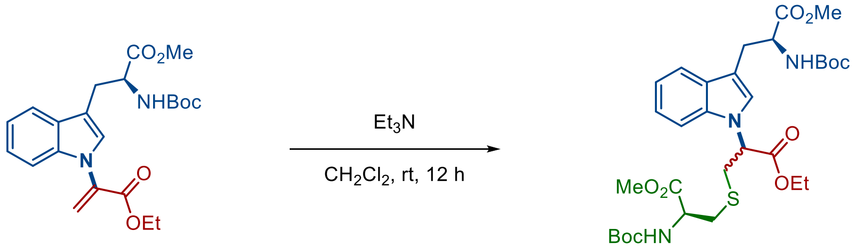

Product **59'**

65 (Boc-Trp-OMe)-A-(Boc-Cys-OMe) (94%)

To a stirring solution of **59´** (0.5 mmol, 1.0 equiv.) and Boc-Cys-OMe (0.6 mmol, 1.2 equiv.) in CH_2Cl_2 (2 ml, 0.25 M) at room temperature and pressure was added triethylamine (1.5 mmol, 3.0 equiv.). The reaction was then stirred for 12 h and, upon reaction completeness (monitored by TLC), directly purified by silica gel chromatography (hexane:ethyl acetate = 4:1) to afford the desired product **65** in 94% yield as colourless oil.

4. Mechanistic investigation

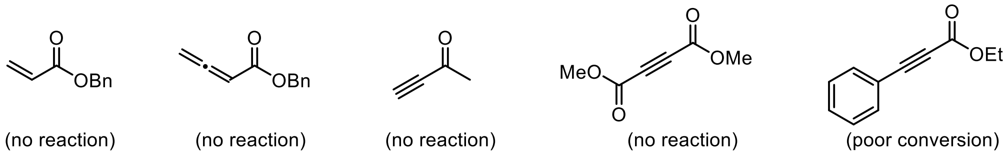

- Only propiolates are the uniquely feasible partner due to the *in-situ* generated strongly basic intermediate upon phosphine activation

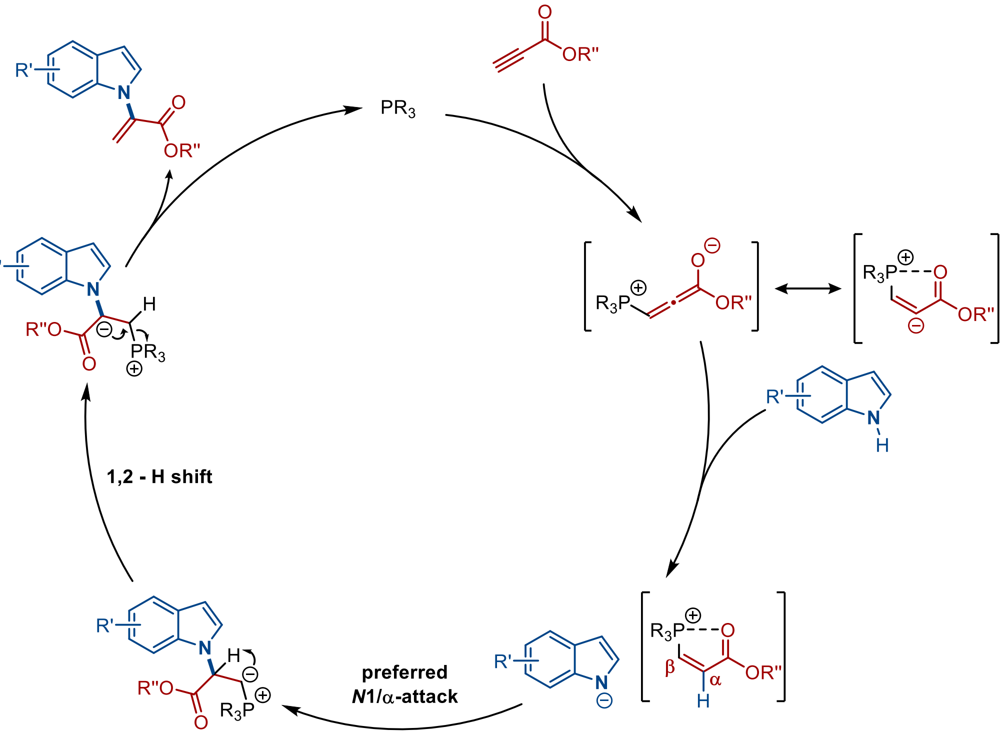

5. Computational studies

General details

All calculations were carried out using density functional theory (DFT) in Gaussian 09 packag.[1] Geometrical optimization of the studied species were performed using M06-2X functional[2] with 6-31G(d) basis set in the gas phase. Vibrational frequency calculation was performed at the same level of theory to confirm the stationary points as real minima (NIMAG = 0) or transition state (TS) (NIMAG = 1). Based on the optimized geometries, the energies were further improved by M06-2X/6-311++G(d,p) single-point calculations considering the solvent effect. Intrinsic reaction coordinate (IRC) analysis was performed to ensure that TS properly connects reactant and product. SMD solvation model[3] was used to take into account the solvent effect. 3D representations of the optimized structures were prepared using CYLView.[4] NCI (noncovalent interactions)

analysis was performed by Multiwfn program[5] according to the previously described methodology[6]. VMD software[7] was used to generate the NCI plots.

Results and Discussion

In order to get more details of the reaction mechanism, we performed DFT calculations. We focused on the second step (alkenylation) of the reaction, and four possible pathways were considered, Cα-N1, Cα-C2, Cα-C3 and Cβ-N1 pathways. The results of the calculated reaction Gibbs free energy ($\Delta_r G$) and reaction enthalpy ($\Delta_r H$) were presented in Table S2, and Figure S1 depicts the key transition states. As shown in Table S1, the $\Delta_r H$ of the reaction between phosphonium allenolate intermediate (IM1) and indole was calculated to be -26.41, -2.21, -18.12 and -16.94 kcal/mol for Cα-N1, Cα-C2, Cα-C3 and Cβ-N1 pathways, respectively, indicating that all these four pathways were exothermic processes, especially for the Cα-N1 pathway. Considering the reaction Gibbs free energy, although the calculated $\Delta_r G$ values were positive due to the entropic effects, the $\Delta_r G$ of the Cα-N1 pathway was just only 2.14 kcal/mol, significantly smaller than those of others (>10 kcal/mol). These results further highlight the preference of the Cα-N1 pathway, while others are thermodynamically unfavorable.

We further explored the kinetics of these reaction pathways. For Cα-N1 pathway, the Gibbs free energy of activation ($\Delta G^{\neq}$) was calculated to be 19.81 kcal/mol, significantly smaller than those of the other three pathways (>25 kcal/mol), indicating that the Cα-N1 pathway is kinetically favored compared to the other three pathways. Taken together, the Cα-N1 pathway was both thermodynamically and kinetically favorable among the possible pathways, which further explains the experimental observation of *N*-chemoselectivity and α-regioselectivity.

To further explore the character of the TS structures, natural population analysis and noncovalent interactions (NCI, Fig. S2) analysis were performed. NPA results show that the N1 atom in the deprotonated indole has the most negative charge of -0.67, followed by C3 (-0.41) and C2 (-0.05), indicating that N1 position has stronger nucleophilicity than C3 and C2 sites. On the other hand, the Cα site in IM1 is less negatively charged (-0.19) as compared to that of Cβ (-0.49), implying the stronger electrophilicity of Cα site. NCI analysis (Fig. S2) shows that several noncovalent interactions including electrostatic interactions, π–π and CH–π interactions presented between the indoles and phosphonium allenolate intermediate, which can help to stabilize the TS structure. Meanwhile, steric repulsion between the indoles and IM1 may also influence the stability of TSs and thus the selectivity. To sum up, the two factors, noncovalent

interactions (mainly the electrostatic interactions) and steric repulsion, are responsible for the Cα-N1 selectivity.

Table S2 Calculated reaction Gibbs free energy ($\Delta_r G$), reaction enthalpy ($\Delta_r H$) and Gibbs free energy of activation ($\Delta G^{\neq}$) at 298.15K

Site	$\Delta_r H$ (kcal/mol)	$\Delta_r G$ (kcal/mol)	$\Delta G^{\neq}$ (kcal/mol)
Cα-N1	-26.41	2.14	19.81
Cα-C2	-2.21	26.04	37.29
Cα-C3	-18.12	10.97	26.74
Cβ-N1	-16.94	13.58	25.27

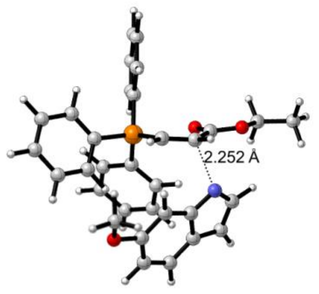

Cα-N1-TS

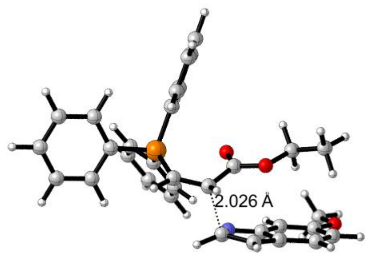

Cα-C2-TS

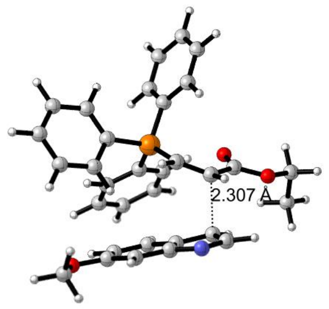

Cα-C3-TS

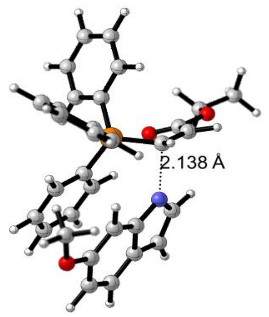

Cβ-N1-TS

Figure S1 The optimized transition state structures of different pathways.

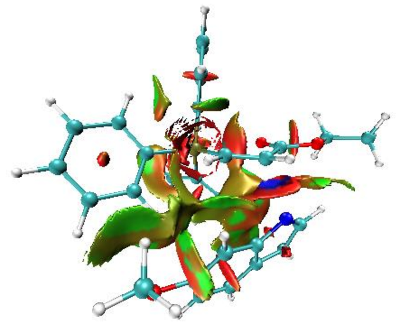

Cα-N1-TS

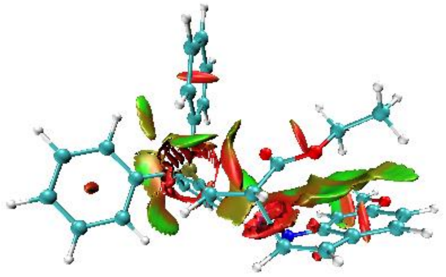

Cα-C2-TS

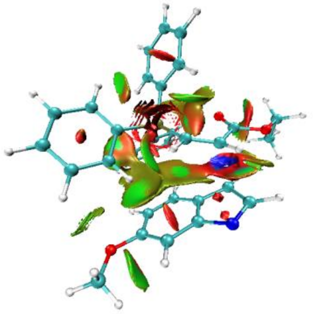

Cα-C3-TS

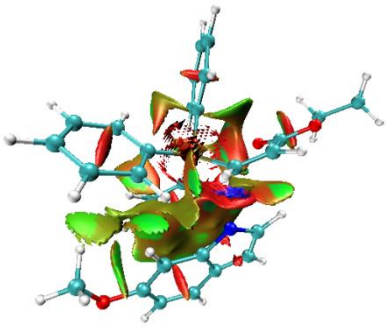

Cβ-N1-TS

Figure S2 NCI plots of the studied TSs in the gas phase. Blue regions refer to strong attractive interactions, green regions refer to weak dispersion-based interactions, and red regions refer to repulsive interactions.

xyz file giving the Cartesian coordinates

PP_3

P	0.00000000	0.00000000	1.27733800
C	0.37062100	1.60317600	0.44510000
C	1.17403000	1.73457700	-0.69176400
C	1.39029800	2.98806900	-1.26073900
C	0.80243100	4.12187000	-0.70634300
C	0.00000000	4.00136000	0.42611400
C	-0.20634200	2.75149300	1.00118800
C	1.20308100	-1.12255500	0.44510000

C	0.91517300	-1.88402900	-0.69176400
C	1.89259500	-2.69806800	-1.26073900
C	3.16842800	-2.75586100	-0.70634300
C	3.46527900	-2.00068000	0.42611400
C	2.48603400	-1.19704900	1.00118800
C	-1.57370200	-0.48062100	0.44510000
C	-2.27969200	-1.55444400	1.00118800
C	-3.46527900	-2.00068000	0.42611400
C	-3.97085900	-1.36600900	-0.70634300
C	-3.28289300	-0.29000100	-1.26073900
C	-2.08920300	0.14945100	-0.69176400
H	1.63277600	0.85480700	-1.13433900
H	2.01869200	3.07728500	-2.14192100
H	0.97251700	5.09707900	-1.15228600
H	-0.45753900	4.88189400	0.86684100
H	-0.82158400	2.66241800	1.89367400
H	-0.07610400	-1.84142900	-1.13433900
H	1.65566100	-3.28688100	-2.14192100
H	3.92794200	-3.39076400	-1.15228600
H	4.45661400	-2.04470700	0.86684100
H	2.71651400	-0.61969700	1.89367400
H	-1.89493000	-2.04272200	1.89367400
H	-3.99907500	-2.83718800	0.86684100
H	-4.90045900	-1.70631500	-1.15228600
H	-3.67435300	0.20959600	-2.14192100
H	-1.55667200	0.98662200	-1.13433900

propiolate

C	2.95250600	-0.92478100	-0.00003400
C	1.90933400	-0.32586700	0.00001500
C	0.66921900	0.44207200	0.00002000
O	0.61969400	1.64545100	-0.00002600
O	-0.39490600	-0.36650600	0.00005400
C	-2.73500300	-0.76251800	-0.00003200
C	-1.66559800	0.30663800	0.00001200
H	3.87845500	-1.45687400	-0.00000700
H	-3.72460200	-0.29789600	-0.00015900
H	-2.64726000	-1.39485900	-0.88695200
H	-2.64744500	-1.39477000	0.88697000
H	-1.72013300	0.94977800	0.88337200
H	-1.72006900	0.94979900	-0.88333600

indole

C	0.87956500	1.62329000	-0.00000100
C	1.42177600	0.31621100	-0.00000200
C	0.60368900	-0.80244700	0.00001000
C	-0.78031700	-0.57171200	0.00001000
C	-1.34911800	0.72024400	-0.00000200
C	-0.48226800	1.82774700	-0.00001300
N	-1.81651400	-1.47273000	0.00003600
C	-3.01738600	-0.79028900	-0.00005800
C	-2.77572400	0.55184100	0.00002500

O	2.78410500	0.26486000	0.00002000
C	3.37987600	-1.00944800	-0.00002400
H	1.57764800	2.45329000	0.00000700
H	0.99910800	-1.81132500	0.00003500
H	-0.88113200	2.83793800	-0.00001300
H	-1.71688300	-2.47491800	0.00005600
H	-3.95169700	-1.33254300	-0.00009900
H	-3.52219500	1.33276900	0.00004400
H	4.45733600	-0.84331500	0.00001100
H	3.09998200	-1.58218500	0.89355200
H	3.10003500	-1.58210800	-0.89366500

IM1

C	-0.01412600	-0.01266400	2.16201300
C	1.22617700	-0.37517100	2.49118800
P	-0.64234100	0.03408500	0.46571100
C	2.22593400	-0.79431100	1.55666000
O	2.26733900	-1.88380500	0.98768300
O	3.23968500	0.10811200	1.42099600
C	5.27902300	0.83760700	0.46791500
C	4.22968300	-0.25410900	0.46206100
C	-2.02477200	1.20549800	0.36928400
C	-3.12982100	0.96186500	1.19544400
C	-4.19301000	1.85629300	1.21774100
C	-4.16032100	2.99666500	0.41635400
C	-3.06509700	3.23988800	-0.40728600

C	-1.99473400	2.34871400	-0.43253900
C	0.63195200	0.57181800	-0.69757100
C	0.81028100	-0.06366900	-1.92859600
C	1.76381700	0.41821200	-2.82117300
C	2.52967300	1.53332900	-2.48864000
C	2.35703500	2.16225500	-1.25678100
C	1.41883500	1.67644500	-0.35204700
C	-1.30801400	-1.55515400	-0.10282800
C	-0.65214500	-2.73152600	0.27777100
C	-1.14346100	-3.95646000	-0.16694800
C	-2.27629300	-4.01148800	-0.97505600
C	-2.92488100	-2.83788600	-1.35471600
C	-2.44302400	-1.60738200	-0.92114000
H	-0.80456100	0.23272900	2.87670100
H	6.06587300	0.61357300	-0.25842600
H	4.83175900	1.80182300	0.20967700
H	5.73244100	0.92707000	1.45857900
H	4.65587200	-1.22698200	0.72499000
H	3.75877600	-0.35817300	-0.52411700
H	-3.16160100	0.06652000	1.81214600
H	-5.04665100	1.66372200	1.85976100
H	-4.99117800	3.69507200	0.43441900
H	-3.04153800	4.12591200	-1.03379800
H	-1.13959500	2.54248500	-1.07361300
H	0.21526800	-0.93597200	-2.18251700
H	1.90964700	-0.07930000	-3.77451900

H	3.27012700	1.90916300	-3.18826100
H	2.96211600	3.02382200	-0.99328700
H	1.30966900	2.13434600	0.62718400
H	0.24712600	-2.68080600	0.88886200
H	-0.63631700	-4.87067500	0.12439400
H	-2.65634800	-4.97135200	-1.31180700
H	-3.80738400	-2.87911400	-1.98537400
H	-2.95083800	-0.69220100	-1.21274800

IM1-protonated

C	-0.01213100	-0.05243500	-2.02371000
P	-0.67369100	-0.04039900	-0.33464000
C	2.26116300	0.70001600	-1.37486100
O	2.00816800	1.53730500	-0.53835600
O	3.41946500	0.08170500	-1.51799800
C	5.57421100	-0.55406000	-0.72647200
C	4.42630800	0.40709700	-0.52751300
C	-2.02438200	-1.23690100	-0.37196600
C	-3.08890600	-0.99691600	-1.25191200
C	-4.14352000	-1.89737700	-1.31632000
C	-4.14227900	-3.02987200	-0.50047400
C	-3.09199700	-3.26013700	0.38250700
C	-2.02648200	-2.36498400	0.45202900
C	0.58821100	-0.59705100	0.82345100
C	0.82830000	0.11165400	2.00167500
C	1.82816300	-0.32458700	2.86659600

C	2.58329100	-1.45224800	2.55138500
C	2.34403900	-2.15494500	1.36988100
C	1.34613600	-1.73014000	0.49993000
C	-1.36861200	1.56011100	0.09752400
C	-0.84472600	2.74219100	-0.43688300
C	-1.40982400	3.95897200	-0.07053600
C	-2.47736000	3.99559000	0.82377800
C	-2.99355500	2.81577600	1.35826200
C	-2.44716600	1.59169900	0.99247700
H	-0.72082100	-0.39966700	-2.77231600
H	6.36101300	-0.34132600	0.00145300
H	5.23982500	-1.58508800	-0.58488400
H	5.99572500	-0.45665100	-1.72940400
H	4.71821100	1.45084100	-0.67184100
H	3.96491400	0.31232100	0.46060500
H	-3.10290000	-0.10354700	-1.87279800
H	-4.96763300	-1.71575100	-1.99796700
H	-4.96807900	-3.73199200	-0.55253900
H	-3.09805700	-4.13743200	1.02045200
H	-1.20932800	-2.54500600	1.14376400
H	0.25717200	1.00670000	2.23016900
H	2.02282300	0.22305400	3.78252900
H	3.36359200	-1.78592300	3.22806500
H	2.93300500	-3.03305100	1.12611200
H	1.16363000	-2.27389100	-0.42414300
H	0.00331400	2.71033100	-1.11172500

H	-1.01349300	4.88014000	-0.48400800
H	-2.91303100	4.94920400	1.10467400
H	-3.82768000	2.84811200	2.05098400
H	-2.86109800	0.66940600	1.39150500
C	1.25021900	0.21055700	-2.36556000
H	1.58861100	0.06106900	-3.38799500

Cα-N1-TS2

C	0.36236400	-0.02054700	-1.52661900
P	0.81266000	-0.95605200	-0.09386000
C	1.51461600	2.06242400	-0.89276800
O	1.95197800	1.62340800	0.15339800
O	1.84305200	3.24906400	-1.39346300
C	2.81199500	5.40761000	-1.23027300
C	2.66168700	4.06809500	-0.54408200
C	0.03955500	-2.57508300	-0.40225200
C	-1.36184800	-2.60277900	-0.41773700
C	-2.02775500	-3.79977100	-0.65164200
C	-1.30146400	-4.97203500	-0.86110400
C	0.08980200	-4.94668500	-0.83378300
C	0.76596800	-3.74890900	-0.60356800
C	2.60337700	-1.23077100	0.01651200
C	3.28340000	-1.14064500	1.23125700
C	4.65414300	-1.38065000	1.27242800
C	5.34382000	-1.70697900	0.10791000
C	4.66643600	-1.78564900	-1.10750700

C	3.29796600	-1.54261500	-1.15642500
C	0.15632000	-0.48563800	1.52468900
C	-0.37510100	0.77922600	1.76224600
C	-0.93001100	1.06169900	3.00882900
C	-0.93292900	0.09967700	4.01146900
C	-0.39655000	-1.16713700	3.77276000
C	0.13400900	-1.46846400	2.52602200
H	-0.21451500	-0.60656600	-2.23296100
H	3.43019300	6.07187500	-0.62069800
H	3.28631300	5.28907000	-2.20765800
H	1.83445300	5.87474500	-1.37496000
H	2.16882500	4.15324900	0.42935100
H	3.62252700	3.56724700	-0.39134800
H	-1.93380100	-1.69342800	-0.24029700
H	-3.11360500	-3.80440800	-0.65982400
H	-1.82159700	-5.90812100	-1.03973400
H	0.65480400	-5.86023000	-0.98961500
H	1.85150000	-3.73516200	-0.57805300
H	2.74886500	-0.86386900	2.13458200
H	5.18439200	-1.30337900	2.21614900
H	6.41287400	-1.89182600	0.14466700
H	5.20454500	-2.02845900	-2.01819900
H	2.76870800	-1.58459300	-2.10529000
H	-0.36568400	1.54311100	0.99531100
H	-1.36488200	2.04322700	3.16899700
H	-1.36494100	0.32717400	4.98129200

H	-0.41009300	-1.92374700	4.55074000
H	0.51890700	-2.46614400	2.32679700
C	0.58863100	1.29935600	-1.76827100
H	0.37586300	1.72243300	-2.73986800
C	-4.29708200	0.61952200	0.55293200
C	-3.72584000	0.09325400	-0.62403200
C	-2.67467300	0.73037000	-1.26582700
C	-2.16205400	1.90986400	-0.68965300
C	-2.74592700	2.46505200	0.48969800
C	-3.82591100	1.80308500	1.09386700
N	-1.08945900	2.64614100	-1.10326800
C	-0.97738800	3.65965500	-0.18167200
C	-1.95568400	3.61988800	0.79216600
O	-4.26898400	-1.09779400	-1.06486700
C	-4.01444800	-1.44349500	-2.40467200
H	-5.12580900	0.08045800	0.99977000
H	-2.27018300	0.36109400	-2.20002600
H	-4.29416100	2.20879600	1.98780700
H	-0.19103500	4.40007400	-0.29377500
H	-2.10706300	4.33032200	1.59490500
H	-4.63983300	-2.31090800	-2.62523900
H	-4.27234300	-0.62024700	-3.08247600
H	-2.96183900	-1.71696800	-2.56548000

Cα-C2-TS2

C	1.07740000	0.32713300	-1.68063000

P	1.99675800	-0.01060200	-0.25666200
C	-0.93216700	1.13401600	-0.49150400
O	-0.58296100	0.68977500	0.58348000
O	-1.94916100	1.97259100	-0.65576200
C	-3.71468300	3.34174300	0.14641400
C	-2.74769600	2.23740100	0.51107700
C	3.68333300	-0.28837100	-0.90360800
C	3.86254500	-1.32899500	-1.82362200
C	5.12305200	-1.59143200	-2.34646100
C	6.21701000	-0.82432400	-1.94770400
C	6.04626300	0.20138400	-1.02420700
C	4.78282900	0.47096600	-0.50007400
C	2.15334500	1.39773500	0.89687600
C	2.07370300	1.25027500	2.28143300
C	2.21044500	2.36454700	3.10555100
C	2.42356900	3.62432500	2.55285100
C	2.49213100	3.77522200	1.16929500
C	2.35125700	2.66583200	0.34238000
C	1.69751300	-1.50500100	0.74344300
C	0.48528500	-2.18768300	0.65128600
C	0.30558300	-3.35328000	1.39476500
C	1.31669900	-3.82884200	2.22357000
C	2.52975000	-3.14571800	2.30908700
C	2.72524700	-1.98895100	1.56355200
H	1.67154700	0.26510000	-2.58519400
H	-3.17590300	4.24952500	-0.13894300

H	-4.34826400	3.02850000	-0.68678400
H	-4.35594400	3.57056600	1.00210000
H	-3.26717100	1.31285900	0.78520800
H	-2.08383800	2.51934100	1.33343700
H	3.01054000	-1.93282600	-2.12663300
H	5.25335700	-2.39631600	-3.06294000
H	7.20195700	-1.02961200	-2.35587300
H	6.89689400	0.79772700	-0.70908300
H	4.65806600	1.27301300	0.22082200
H	1.88359100	0.27344700	2.71485600
H	2.14001200	2.24740500	4.18253900
H	2.52589300	4.49071900	3.19933800
H	2.64609700	4.75762600	0.73363200
H	2.38279200	2.77847700	-0.73891000
H	-0.31347400	-1.82778800	0.00153600
H	-0.63543800	-3.88966700	1.31605600
H	1.16719400	-4.73818200	2.79863600
H	3.32504300	-3.51920300	2.94681100
H	3.67918800	-1.46773500	1.61426800
C	-0.25468200	0.78849200	-1.77618800
H	-0.47130100	1.47040100	-2.59244900
C	-5.70278500	0.11867700	-0.67724200
C	-5.14923500	-0.79595100	0.29399400
C	-3.89715100	-1.33115900	0.15776600
C	-3.13522400	-0.96234500	-0.99669200
C	-3.69211600	-0.04931400	-1.98535400

C	-5.00231500	0.48374500	-1.78557300
N	-1.90035400	-1.33677600	-1.28916400
C	-1.55987800	-0.61345200	-2.43555000
C	-2.69728100	0.12457500	-2.92382800
O	-5.99689800	-1.04762400	1.32896400
C	-5.53702200	-1.93248800	2.32341900
H	-6.70268300	0.49233500	-0.48191900
H	-3.45961400	-2.00937000	0.88073500
H	-5.43353900	1.17143500	-2.50842900
H	-0.81041300	-1.03608600	-3.09736500
H	-2.71856200	0.75711100	-3.80324100
H	-6.33808000	-2.01208000	3.05879200
H	-5.32342800	-2.92289100	1.90253400
H	-4.62961700	-1.54787600	2.80595900

Cα-C3-TS2

C	0.25070100	0.38978600	-1.34974500
P	0.67628600	-0.91520800	-0.26681200
C	1.94960600	2.11652300	-0.74502400
O	2.55942100	1.43590100	0.05918700
O	2.35331500	3.33262100	-1.13696400
C	3.12008900	4.42579900	0.89254200
C	3.50308900	3.86437900	-0.46454100
C	-0.50966800	-2.21472200	-0.73225200
C	-1.85440700	-1.84808300	-0.87480800
C	-2.81262100	-2.82025200	-1.13944600

C	-2.43347700	-4.15399600	-1.28100600
C	-1.09543600	-4.51626800	-1.15297400
C	-0.13153400	-3.55085500	-0.87028200
C	2.34617500	-1.58229300	-0.55781600
C	2.93192400	-2.45663100	0.36268300
C	4.17697500	-3.01657600	0.09245800
C	4.84222600	-2.69853200	-1.08917900
C	4.26230000	-1.82186800	-2.00337500
C	3.01479500	-1.26459300	-1.74103200
C	0.54256800	-0.79889100	1.55979500
C	1.51492200	-0.13501400	2.31782200
C	1.37529700	-0.04627100	3.69987700
C	0.28192800	-0.62688900	4.33778900
C	-0.66982600	-1.31355800	3.58965300
C	-0.54156100	-1.40258300	2.20708500
H	-0.55289800	0.11193400	-2.02169100
H	3.99127000	4.88360300	1.37018200
H	2.34270300	5.18715600	0.78448200
H	2.74930300	3.62819900	1.54161500
H	4.25679800	3.07875900	-0.37137500
H	3.87332800	4.64481700	-1.13152900
H	-2.15686300	-0.80902300	-0.75957100
H	-3.85516400	-2.52823900	-1.21960800
H	-3.18215500	-4.91236800	-1.48906500
H	-0.79793300	-5.55364200	-1.26978700
H	0.91021300	-3.84024400	-0.76652500

H	2.41848000	-2.69608400	1.29014700
H	4.62908100	-3.69595700	0.80821800
H	5.81605700	-3.13217000	-1.29547600
H	4.78397300	-1.56878900	-2.92096600
H	2.55497700	-0.57442300	-2.44302300
H	2.36918300	0.31466700	1.82794500
H	2.12856100	0.47955800	4.27838300
H	0.17656500	-0.55143800	5.41587700
H	-1.51895900	-1.78062900	4.07877200
H	-1.30150000	-1.92725600	1.63854100
C	0.71188700	1.69035300	-1.41881500
H	0.45289200	2.25289000	-2.30594200
C	-3.03425800	0.63565400	1.32876700
C	-3.93456700	0.70822300	0.24952300
C	-3.69637100	1.55792700	-0.82861400
C	-2.53398400	2.33659200	-0.80412900
C	-1.61033700	2.26470000	0.27395500
C	-1.87324900	1.39809300	1.33932100
N	-2.14042500	3.26396500	-1.76692000
C	-1.00732100	3.75180600	-1.30782600
C	-0.52794200	3.12237500	-0.10256600
O	-5.02114200	-0.12376000	0.34381500
C	-5.98468400	-0.03019800	-0.67719400
H	-3.27521900	-0.03560100	2.14755600
H	-4.37451700	1.64636700	-1.66932000
H	-1.19336800	1.32995600	2.18546900

H	-0.46135100	4.50755900	-1.86912500
H	0.22613400	3.53099600	0.55993600
H	-6.77869100	-0.73100900	-0.41480800
H	-6.39972000	0.98294900	-0.74643200
H	-5.56467900	-0.30508400	-1.65442200

Cβ-N1-TS2

C	-0.86677400	-0.11062100	-1.66870700
P	-0.52128000	0.70494600	-0.07638700
C	-2.95088700	-1.20891900	-0.95075000
O	-2.71541400	-1.20899200	0.25057000
O	-4.10056400	-1.69186300	-1.45257500
C	-6.22744600	-2.71840700	-1.25010200
C	-5.02175100	-2.21171000	-0.48823700
C	0.49271600	2.15310100	-0.53566000
C	1.28090200	2.18509400	-1.68871200
C	2.06545500	3.30137900	-1.97113500
C	2.07175000	4.38936700	-1.10395000
C	1.28573200	4.36558000	0.04658600
C	0.49612500	3.25669700	0.32768500
C	-2.02112300	1.47726000	0.59631800
C	-2.34847600	1.34975500	1.94603300
C	-3.47876500	1.99269600	2.44332100
C	-4.28097600	2.75267600	1.59656800
C	-3.95319400	2.87739600	0.24769800
C	-2.82093700	2.24544400	-0.25439900

C	0.37303000	-0.18568300	1.21576600
C	-0.13327700	-1.37134200	1.76572000
C	0.62111200	-2.05507500	2.71296200
C	1.86876200	-1.57995200	3.10464300
C	2.36714600	-0.39812100	2.56373700
C	1.62331100	0.30225300	1.62355500
H	-0.33103600	0.34937400	-2.48610200
H	-6.96544400	-3.12830200	-0.55541300
H	-6.69361300	-1.90728400	-1.81511500
H	-5.93519700	-3.50404300	-1.95122500
H	-4.53099800	-3.00762500	0.08033300
H	-5.28415500	-1.41656600	0.21686200
H	1.30922700	1.33938900	-2.36687500
H	2.67391500	3.31317200	-2.86989400
H	2.68530000	5.25704200	-1.32499600
H	1.28296000	5.21312300	0.72418000
H	-0.12121400	3.24748500	1.22195900
H	-1.73257400	0.73804100	2.59816300
H	-3.73573200	1.89131800	3.49280300
H	-5.16399300	3.24891600	1.98704700
H	-4.57852600	3.46825700	-0.41382800
H	-2.56617000	2.34058300	-1.30648800
H	-1.08781000	-1.75914700	1.43676400
H	0.23742200	-2.98359900	3.12310400
H	2.46107800	-2.13729400	3.82373200
H	3.35011200	-0.03488700	2.84466900

H	2.03823100	1.20247700	1.18476100
C	-2.06547200	-0.69093700	-1.97100800
H	-2.32917300	-0.86887900	-3.00635100
C	4.42784200	-2.16860100	0.29762200
C	4.22245400	-0.93805000	-0.36228800
C	3.02793900	-0.65045100	-1.00118900
C	1.99008200	-1.60196700	-0.94162200
C	2.19401500	-2.85737000	-0.29198300
C	3.42979400	-3.12234800	0.32186400
N	0.71214800	-1.53464400	-1.44587400
C	0.12075600	-2.72055500	-1.11246000
C	0.96467700	-3.56955500	-0.43250900
O	5.28846700	-0.07038800	-0.31374000
C	5.10415100	1.19015800	-0.89851400
H	5.39107900	-2.33726600	0.76795700
H	2.88999600	0.28711300	-1.52138000
H	3.60232300	-4.07375700	0.81944100
H	-0.91138600	-2.89976600	-1.39779300
H	0.74025900	-4.56846800	-0.08261500
H	6.02672100	1.74842300	-0.73113100
H	4.91901000	1.11465100	-1.97944000
H	4.26159800	1.73064000	-0.44132600

IM2-Cα-N1

C	0.43986700	-0.37702600	-1.37309100
P	1.71866400	-0.18164500	-0.28589200

C	-1.12896000	0.91345500	-0.00301000
O	-0.94285800	0.65390300	1.16273600
O	-1.39521800	2.14620000	-0.45090700
C	-1.61228600	4.48809300	-0.14361600
C	-1.41288800	3.16585300	0.56185000
C	3.22922100	-0.53680000	-1.24196000
C	3.33833800	-1.79106200	-1.85459700
C	4.47262500	-2.11210900	-2.59130200
C	5.51086900	-1.18993100	-2.71211500
C	5.41203500	0.05220900	-2.09383600
C	4.27419100	0.38093900	-1.35860100
C	1.92272700	1.52192300	0.37216300
C	2.20092700	1.80625000	1.71028500
C	2.35436200	3.12870300	2.12338700
C	2.24226100	4.16809400	1.20298700
C	1.95837300	3.88858300	-0.13346200
C	1.78202900	2.57071500	-0.54431700
C	1.89537900	-1.25866200	1.19204100
C	0.76791100	-1.83300700	1.78387700
C	0.91453800	-2.66945400	2.88791100
C	2.17935300	-2.93393200	3.40536200
C	3.30672300	-2.36070200	2.81974100
C	3.16646400	-1.52791500	1.71437300
H	0.73997600	-0.71526000	-2.35706600
H	-1.62041600	5.30371000	0.58460600
H	-0.79981300	4.66311500	-0.85404100

H	-2.55920100	4.49897600	-0.69051900
H	-2.22184200	2.94213700	1.26506600
H	-0.46801700	3.12965100	1.11298000
H	2.53278800	-2.51330500	-1.75205800
H	4.54940800	-3.08481200	-3.06708400
H	6.39736500	-1.44328600	-3.28531900
H	6.21970100	0.77194600	-2.18350600
H	4.20211200	1.35276200	-0.87989300
H	2.28361900	0.99898000	2.43180100
H	2.56078700	3.34594000	3.16683900
H	2.36968400	5.19660200	1.52748200
H	1.86468200	4.69711100	-0.85254400
H	1.52256000	2.34058000	-1.57544600
H	-0.21977500	-1.61457900	1.39154200
H	0.03421100	-3.11446900	3.34094300
H	2.28874700	-3.58969700	4.26412900
H	4.29505500	-2.56711100	3.21924900
H	4.04916900	-1.09124800	1.25259100
C	-1.00791200	-0.10326700	-1.13305300
H	-1.44962000	0.34815000	-2.03025500
C	-5.85755800	-1.63127000	-0.06935800
C	-5.36768100	-0.32686400	-0.30745600
C	-4.02515700	-0.09611100	-0.56447200
C	-3.18259400	-1.22008000	-0.57318200
C	-3.64523400	-2.53526900	-0.34759500
C	-5.01407900	-2.72187400	-0.08884000

N	-1.82766000	-1.27697800	-0.79540600
C	-1.42848200	-2.59710700	-0.73589100
C	-2.50133000	-3.39578800	-0.45768700
O	-6.31406000	0.65712800	-0.26517000
C	-5.87827600	1.97360600	-0.49601400
H	-6.91907200	-1.73947400	0.12573500
H	-3.63816800	0.89799100	-0.75700500
H	-5.40980000	-3.71743900	0.09133400
H	-0.38842300	-2.83859600	-0.89863100
H	-2.48151900	-4.47055100	-0.34782900
H	-6.76277200	2.60733300	-0.42244300
H	-5.13997400	2.28876400	0.25396300
H	-5.43522800	2.08060600	-1.49478300

C. Reaction development and analytical data

Table S3 Optimization of the reaction conditions for phosphine-catalyzed hydroamination of indole with ethyl propiolates.[a]

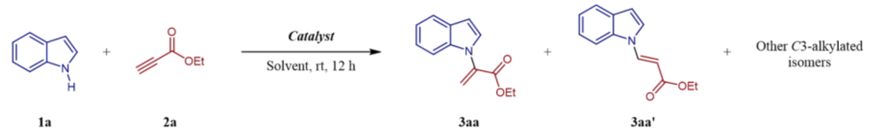

Entry	Catalyst	Solvent	Yield[b] (%) of 3aa	Yield[b] (%) of 3aa'
1	PPh_3	CH_2Cl_2	81	trace
2	$MePPh_2$	CH_2Cl_2	73	12
3	DABCO	CH_2Cl_2	40	47
5	DMAP	CH_2Cl_2	33	42
6	PPh_3	Toluene	69	15
7	PPh_3	Ether	61	21
8	PPh_3	1,4-Dioxane	64	trace
9	PPh_3	MeCN	75	13
10	PPh_3	CCl_3	78	10
11	PPh_3	EtOAc	80	trace
12	PPh_3	THF	44	34
13	PPh_3	Acetone	32	29
14	PPh_3	MeOH	28	36
15	PPh_3	DMSO	NA	NA
16	PPh_3	DMF	NA	NA

[a]Reaction conditions: **1a** (1 mmol), **2a** (1.2 mmol), and the catalyst (20 mol%) in the solvent specified (1 mL, 1M) at room temperature. [b] Yields refer to isolated products. NA = not applicable, either due to no reaction or too messy reactions.

We started our investigation by examining the *N*-alkenylation reaction of 1*H*-indole and ethyl propiolate in the presence of various Lewis bases in different solvents (**Table 1**). We initially quickly probed the performance of four commercially available nucleophilic organocatalysts: trphenylphosphine (PPh_3), methyldiphenylphosphine ($MePPh_2$), 1,4-diazabicyclo[2.2.2]octane (DABCO), and 4-dimethylaminopyridine (DMAP), and the results showed that PPh_3 gave the highest (81%) and cleanest **3aa** (trace amounts of side-products), which reproduced the result of the early literatures.[8] Solvent screening then followed. Dichloromethane (CH_2Cl_2), chloroform ($CHCl_3$) and ethyl acetate (EtOAc), all gave almost the same ~80% yields; since LSF is our ultimate goal, where their compromised solubilities towards a wide range of natural products, drugs and peptides, relative toxicity, and operational simplicity should be considered, CH_2Cl_2 was chosen as the optimal solvent, where the reaction was found complete after 1.5 h at the ambient condition.

Table S4 Scopes of propiolates (2), and indoles and their analogues (1A).[a]

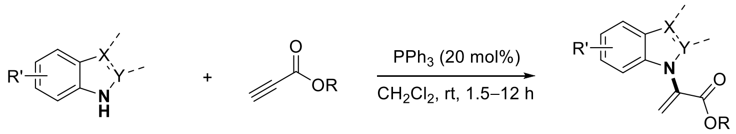

1A 2 3-38

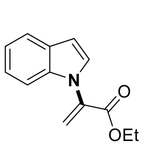

3aa

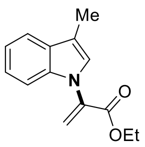

4a

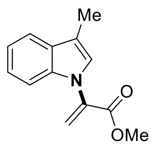

4b

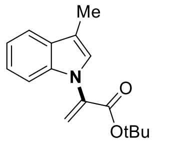

4c

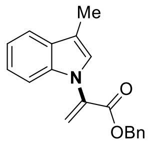

4d

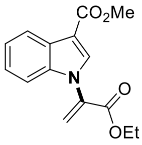

5

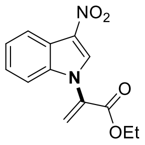

6

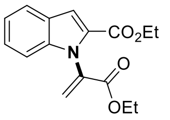

7

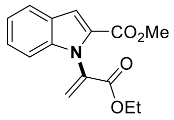

8

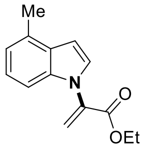

9

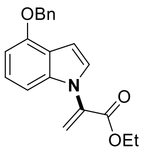

10

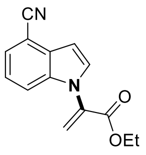

11

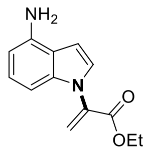

12

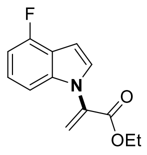

13

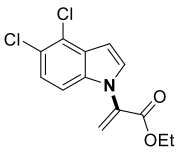

14

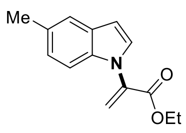

15

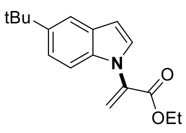

16

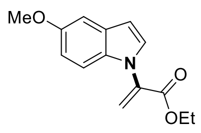

17

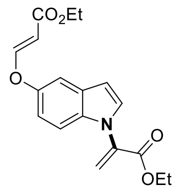

18

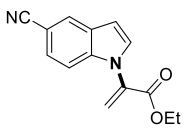

19

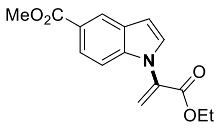

20

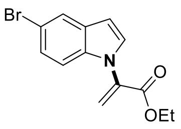

21

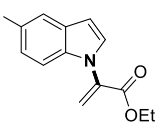

22

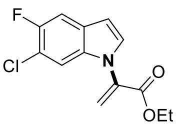

23

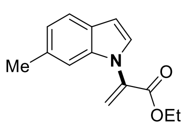

24

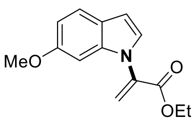

25

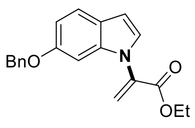

26

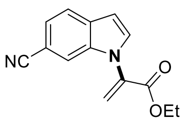

27

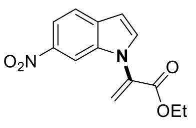

28

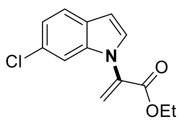

29

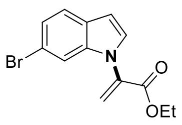

30

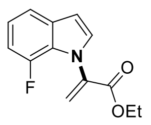

31

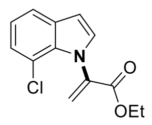

32

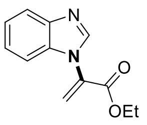

33

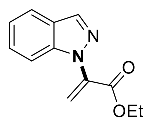

34

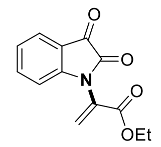

35

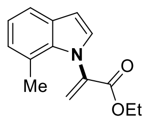

36

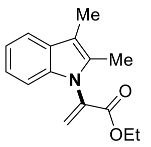

37

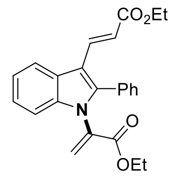

38

[a] Reaction conditions: **1A** (1 mmol), **2** (1.2 mmol), and the triphenylphosphine catalyst (20 mol%) in CH_2Cl_2 (1 mL) at room temperature. [b] Yields refer to isolated products. [c] 2.1 mmol of **2** instead.

Ethyl 2-(1H-indol-1-yl)acrylate (3aa)[8]

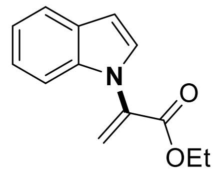

3aa

Colourless oil; 174 mg, 81% yield; flash chromatography on silica gel. (Hexane/EtOAc = 10:1); **^{1}H NMR (400 MHz, CDCl$_3$)** δ 7.52 (dt, *J* = 7.7, 1.1 Hz, 1H), 7.20 (dq, *J* = 8.3, 0.9 Hz, 1H), 7.13 – 7.08 (m, 1H), 7.07 (d, *J* = 3.3 Hz, 1H), 7.03 (ddd, *J* = 8.0, 7.0, 1.1 Hz, 1H), 6.50 (dd, *J* = 3.3, 0.9 Hz, 1H), 6.38 (s, 1H), 5.78 (s, 1H), 4.19 (q, *J* = 7.1 Hz, 2H), 1.19 (t, *J* = 7.1 Hz, 3H). **^{13}C NMR (101 MHz, CDCl$_3$)** δ 163.6, 136.5, 136.3, 129.2, 128.6, 122.5, 121.2, 120.9, 120.7, 110.8, 103.7, 62.1, 14.2. **HRMS (ESI)** m/z: $[M + H]^+$ Calcd for $C_{13}H_{14}NO_2$ 216.1019; Found 216.1021.

Ethyl 2-(3-methyl-1H-indol-1-yl)acrylate (4a)

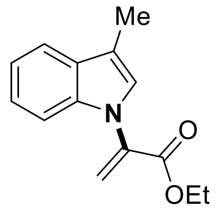

4a

Colourless oil; 209 mg, 91% yield; flash chromatography on silica gel. (Hexane/EtOAc = 10:1); **^{1}H NMR (400 MHz, CDCl$_3$)** δ 7.60 (dt, J = 7.7, 1.1 Hz, 1H), 7.32 (dt, J = 8.2, 1.0 Hz, 1H), 7.24 (ddd, J = 8.3, 7.0, 1.4 Hz, 1H), 7.18 (ddd, J = 8.0, 7.0, 1.2 Hz, 1H), 7.01 (q, J = 1.2 Hz, 1H), 6.45 (s, 1H), 5.88 (s, 1H), 4.36 (q, J = 7.1 Hz, 2H), 2.37 (d, J = 1.2 Hz, 3H), 1.36 (t, J = 7.1 Hz, 3H). **^{13}C NMR (101 MHz, CDCl$_3$)** δ 163.8, 136.6, 136.2, 129.7, 125.8, 122.4, 120.0, 119.4, 119.1, 112.8, 110.7, 61.9, 14.2, 9.6. **HRMS (ESI)** m/z: $[M + H]^+$ Calcd for $C_{14}H_{16}NO_2$ 230.1176; Found 230.1180.

Methyl 2-(3-methyl-1H-indol-1-yl)acrylate (4b)

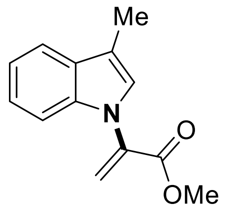

4b

Colourless oil; 194 mg, 90% yield; flash chromatography on silica gel. (Hexane/EtOAc = 10:1); **^{1}H NMR (400 MHz, CDCl$_3$)** 7.61 – 7.56 (m, 1H), 7.31 – 7.27 (m, 1H), 7.23 (ddt, J = 8.2, 6.9, 1.2 Hz, 1H), 7.20 – 7.14 (m, 1H), 6.98 (t, J = 1.2 Hz, 1H), 6.45 (s, 1H), 5.88 (s, 1H), 3.87 (s, 3H), 2.35 (s, 3H). **^{13}C NMR (101 MHz, CDCl$_3$)** δ 164.4, 136.6, 135.9, 129.7, 125.8, 122.5, 120.1, 120.0, 119.2, 112.9, 110.6, 52.8, 9.6. **HRMS (ESI)** m/z: $[M + H]^+$ Calcd for $C_{13}H_{14}NO_2$ 216.1019; Found 216.1015.

Ethyl 2-(3-methyl-1H-indol-1-yl)acrylate (4c)

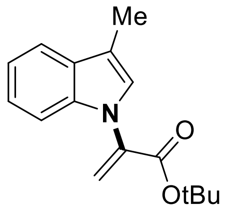

4c

Colourless oil; 177 mg, 77% yield; flash chromatography on silica gel. (Hexane/EtOAc = 10:1); **^{1}H NMR (500 MHz, CDCl$_3$)** δ 7.63 – 7.56 (m, 1H), 7.34 (dd, *J* = 8.3, 1.0 Hz, 1H), 7.24 (tt, *J* = 8.1, 1.1 Hz, 1H), 7.19 – 7.15 (m, 1H), 7.01 (t, *J* = 1.2 Hz, 1H), 6.36 (s, 1H), 5.82 (s, 1H), 2.36 (s, 3H), 1.56 (s, 9H). **^{13}C NMR (126 MHz, CDCl$_3$)** δ 162.7, 137.2, 136.5, 129.6, 126.0, 122.1, 119.8, 119.0, 118.5, 112.4, 110.7, 82.5, 27.9, 9.5. **HRMS (ESI)** m/z: $[M + H]^+$ Calcd for $C_{16}H_{20}NO_2$ 258.1489; Found 258.1487.

Benzyl 2-(3-methyl-1H-indol-1-yl)acrylate (4d)

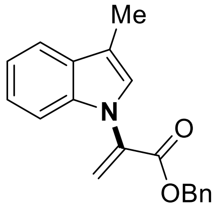

4d

Colourless oil; 216 mg, 74% yield; flash chromatography on silica gel. (Hexane/EtOAc = 10:1); **^{1}H NMR (500 MHz, CDCl$_3$)** δ 7.68 (dt, *J* = 7.5, 0.9 Hz, 1H), 7.55 – 7.40 (m, 5H), 7.39 – 7.33 (m, 1H), 7.33 – 7.24 (m, 2H), 7.08 (d, *J* = 1.2 Hz, 1H), 6.55 (s, 1H), 5.96 (s, 1H), 5.39 (s, 2H), 2.44 (d, *J* = 1.3 Hz, 3H). **^{13}C NMR (126 MHz, CDCl$_3$)** δ 163.5, 136.5, 135.8, 135.2, 129.6, 128.5, 128.4, 128.3, 125.8, 122.3, 120.0, 119.8, 119.0, 112.7, 110.6, 67.4, 9.5. **HRMS (ESI)** m/z: $[M + H]^+$ Calcd for $C_{19}H_{18}NO_2$ 292.1332; Found 292.1328.

Methyl 1-(3-ethoxy-3-oxoprop-1-en-2-yl)-1H-indole-3-carboxylate (5)

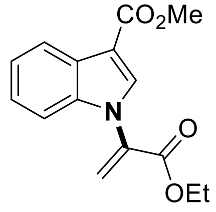

5

Colourless oil; 246 mg, 90% yield; flash chromatography on silica gel. (Hexane/EtOAc = 10:1); **^{1}H NMR (400 MHz, CDCl$_3$)** δ 8.29 – 8.18 (m, 1H), 7.92 (s, 1H), 7.37 – 7.24 (m, 3H), 6.67 (d, J = 0.7 Hz, 1H), 6.03 (d, J = 0.6 Hz, 1H), 4.33 (q, J = 7.1 Hz, 2H), 3.94 (s, 3H), 1.32 (t, J = 7.1 Hz, 3H). **^{13}C NMR (101 MHz, CDCl$_3$)** δ 165.2, 162.7, 137.0, 135.4, 134.7, 126.5, 123.8, 123.5, 122.5, 121.8, 110.9, 109.2, 62.3, 51.1, 14.1. **HRMS (ESI)** m/z: $[M + H]^+$ Calcd for $C_{15}H_{16}NO_4$ 274.1074; Found 274.1070.

Ethyl 2-(3-nitro-1H-indol-1-yl)acrylate (6)

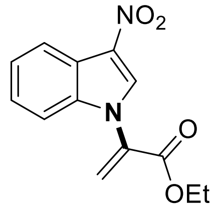

6

Yellowish oil; 216 mg, 83% yield; flash chromatography on silica gel. (Hexane/EtOAc = 8:1); **^{1}H NMR (400 MHz, CDCl$_3$)** δ 8.31 (ddd, J = 7.9, 1.4, 0.8 Hz, 1H), 8.18 (s, 1H), 7.46 – 7.29 (m, 3H), 6.80 (d, J = 1.0 Hz, 1H), 6.15 (d, J = 1.0 Hz, 1H), 4.34 (q, J = 7.1 Hz, 2H), 1.33 (t, J = 7.1 Hz, 3H). **^{13}C NMR (101 MHz, CDCl$_3$)** δ 161.9, 135.8, 134.6, 130.8, 130.6, 125.8, 125.1, 124.6, 121.0, 120.6, 111.4, 62.8, 14.1. **HRMS (ESI)** m/z: $[M + H]^+$ Calcd for $C_{13}H_{13}N_2O_4$ 261.0870; Found 261.0874.

Ethyl 1-(3-ethoxy-3-oxoprop-1-en-2-yl)-1H-indole-2-carboxylate (7)

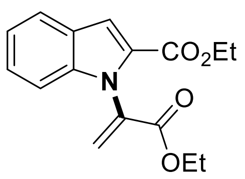

7

Colourless oil; 244 mg, 85% yield; flash chromatography on silica gel. (Hexane/EtOAc = 8:1); **^{1}H NMR (400 MHz, CDCl$_3$)** δ 7.71 (dt, *J* = 8.1, 1.1 Hz, 1H), 7.42 (d, *J* = 0.8 Hz, 1H), 7.41 – 7.32 (m, 2H), 7.21 (ddd, *J* = 8.0, 6.0, 1.9 Hz, 1H), 6.72 (s, 1H), 5.91 (s, 1H), 4.34 (q, *J* = 7.1 Hz, 2H), 4.26 (d, *J* = 11.2 Hz, 2H), 1.38 (t, *J* = 7.2 Hz, 3H), 1.26 (t, *J* = 7.2 Hz, 3H). **^{13}C NMR (101 MHz, CDCl$_3$)** δ 163.7, 161.7, 139.3, 136.3, 129.2, 126.8, 125.7, 122.7, 121.5, 111.2, 110.8, 61.6, 60.9, 14.3, 14.1. **HRMS (ESI)** m/z: $[M + H]^+$ Calcd for $C_{16}H_{18}NO_4$ 288.1230; Found 288.1229.

Methyl 1-(3-ethoxy-3-oxoprop-1-en-2-yl)-1H-indole-2-carboxylate (8)

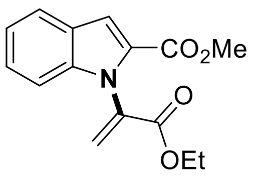

8

White solid; 238 mg, 87% yield; m.p. 86–90 ºC; flash chromatography on silica gel. (Hexane/EtOAc = 8:1); **^{1}H NMR (500 MHz, CDCl$_3$)** δ 7.74 (d, *J* = 8.0 Hz, 1H), 7.45 – 7.32 (m, 3H), 7.26 – 7.17 (m, 1H), 6.75 (s, 1H), 5.95 (s, 1H), 4.27 (s, 2H), 3.90 (s, 3H), 1.28 (t, *J* = 7.1 Hz, 3H). **^{13}C NMR (126 MHz, CDCl$_3$)** δ 163.6, 162.0, 139.2, 136.1, 128.7, 126.7, 125.7, 122.8, 122.7, 121.6, 111.3, 110.8, 61.6, 51.8, 14.0. **HRMS (ESI)** m/z: $[M + H]^+$ Calcd for $C_{15}H_{16}NO_4$ 274.1074; Found 274.1073.

Ethyl 2-(4-methyl-1H-indol-1-yl)acrylate (9)

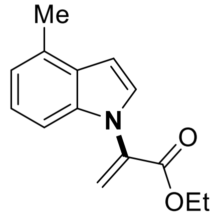

9

Colourless oil; 186 mg, 81% yield; flash chromatography on silica gel. (Hexane/EtOAc = 10:1); **^{1}H NMR (500 MHz, CDCl$_3$)** δ 7.94 (d, *J* = 3.4 Hz, 1H), 7.92 – 7.85 (m, 2H), 7.70 (dt, *J* = 6.8, 1.1 Hz, 1H), 7.38 (dd, *J* = 3.4, 0.8 Hz, 1H), 7.26 (s, 1H), 6.66 (s, 1H), 5.07 (q, *J* = 7.1 Hz, 2H), 3.31 (s, 3H), 2.07 (t, *J* = 7.1 Hz, 3H). **^{13}C NMR (126 MHz, CDCl$_3$)** δ 163.6, 136.3, 136.0, 130.5, 128.7, 127.8, 122.4, 120.8, 120.7, 108.2, 102.0, 61.9, 18.6, 14.1. **HRMS (ESI)** m/z: $[M + H]^+$ Calcd for $C_{14}H_{16}NO_2$ 230.1176; Found 230.1173.

Ethyl 2-(4-(benzyloxy)-1H-indol-1-yl)acrylate (10)

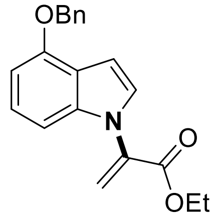

10

Colourless oil; 276 mg, 86% yield; flash chromatography on silica gel. (Hexane/EtOAc = 10:1); **^{1}H NMR (500 MHz, CDCl$_3$)** δ 7.55 – 7.49 (m, 2H), 7.45 – 7.38 (m, 2H), 7.38 – 7.32 (m, 1H), 7.16 – 7.08 (m, 2H), 6.96 (dd, *J* = 8.4, 0.8 Hz, 1H), 6.81 (dd, *J* = 3.3, 0.8 Hz, 1H), 6.64 (d, *J* = 7.8 Hz, 1H), 6.52 (s, 1H), 5.93 (s, 1H), 5.25 (s, 2H), 4.33 (q, *J* = 7.1 Hz, 2H), 1.33 (t, *J* = 7.1 Hz, 3H). **^{13}C NMR (126 MHz, CDCl$_3$)** δ 170.5, 159.6, 144.9, 144.5, 143.4, 135.5, 134.8, 134.3, 134.0, 130.2, 128.1, 126.9, 111.3, 109.0, 108.0, 77.0, 69.0, 21.1. **HRMS (ESI)** m/z: $[M + H]^+$ Calcd for $C_{20}H_{20}NO_3$ 322.1438; Found 322.1441.

Ethyl 2-(4-cyano-1H-indol-1-yl)acrylate (11)

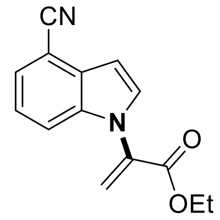

11

White solid; 190 mg, 79% yield; flash chromatography on silica gel. (Hexane/EtOAc = 10:1); m.p. 60–64 °C; **^{1}H NMR (500 MHz, CDCl$_3$)** δ 7.55 – 7.48 (m, 2H), 7.35 (d, *J* = 3.3 Hz, 1H), 7.32 – 7.21 (m, 1H), 6.87 – 6.79 (m, 1H), 6.64 (s, 1H), 5.97 (s, 1H), 4.33 (q, *J* = 7.1 Hz, 2H), 1.32 (t, *J* = 7.1 Hz, 3H). **^{13}C NMR (126 MHz, CDCl$_3$)** δ 162.8, 136.1, 135.5, 131.1, 130.3, 125.8, 123.0, 122.0, 118.4, 115.3, 103.5, 102.3, 62.3, 14.1. **HRMS (ESI)** m/z: $[M + H]^+$ Calcd for $C_{14}H_{13}N_2O_2$ 241.0972; Found 241.0973.

Ethyl 2-(4-amino-1H-indol-1-yl)acrylate (12)

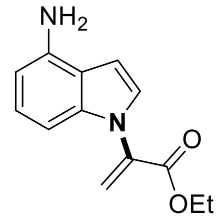

12

Yellowish oil; 140 mg, 61% yield; flash chromatography on silica gel. (Hexane/EtOAc = 5:1); **^{1}H NMR (500 MHz, CDCl$_3$)** δ 7.11 (d, *J* = 3.4 Hz, 1H), 7.05 (dd, *J* = 8.3, 7.5 Hz, 1H), 6.79 (dt, *J* = 8.3, 0.9 Hz, 1H), 6.54 (dd, *J* = 3.5, 0.9 Hz, 1H), 6.51 (s, 1H), 6.45 (dd, *J* = 7.6, 0.8 Hz, 1H), 5.93 (s, 1H), 4.33 (q, *J* = 7.1 Hz, 2H), 3.72 (s, 2H), 1.33 (t, *J* = 7.1 Hz, 3H). **^{13}C NMR (126 MHz, CDCl$_3$)** δ 163.5, 139.4, 137.4, 136.3, 126.7, 123.5, 121.0, 117.9, 105.0, 101.8, 99.7, 61.9, 14.1. **HRMS (ESI)** m/z: $[M + H]^+$ Calcd for $C_{13}H_{15}N_2O_2$ 231.1128; Found 231.1124.

Ethyl 2-(4-fluoro-1H-indol-1-yl)acrylate (13)

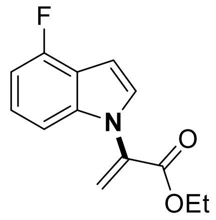

13

Colourless oil; 201 mg, 86% yield; flash chromatography on silica gel. (Hexane/EtOAc = 10:1); **^{1}H NMR (500 MHz, CDCl$_3$)** δ 7.30 – 7.14 (m, 3H), 6.92 (ddd, *J* = 10.1, 7.6, 0.9 Hz, 1H), 6.80 (dd, *J* = 3.3, 0.8 Hz, 1H), 6.66 (s, 1H), 6.04 (s, 1H), 4.42 (q, *J* = 7.2 Hz, 2H), 1.42 (t, *J* = 7.1 Hz, 3H). **^{13}C NMR (126 MHz, CDCl$_3$)** δ 163.3, 156.4 (d, $J_{C\text{-}F}$ = 247.2 Hz), 139.0 (d, $J_{C\text{-}F}$ = 10.7 Hz), 136.1, 128.6, 123.0 (d, $J_{C\text{-}F}$ = 7.7 Hz), 122.0, 118.1 (d, $J_{C\text{-}F}$ = 22.9 Hz), 106.9 (d, $J_{C\text{-}F}$ = 3.5 Hz), 105.5 (d, $J_{C\text{-}F}$ = 18.9 Hz), 99.6, 62.2, 14.2. **^{19}F NMR** (471 MHz, CDCl$_3$) δ -122.09, -122.10. **HRMS (ESI)** m/z: $[M + H]^+$ Calcd for $C_{13}H_{13}FNO_2$ 234.0925; Found 234.0924.

Ethyl 2-(4,5-dichloro-1H-indol-1-yl)acrylate (14)

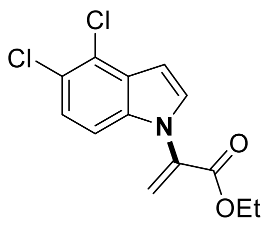

14

Colourless oil; 240 mg, 84% yield; flash chromatography on silica gel. (Hexane/EtOAc = 10:1); **^{1}H NMR (500 MHz, CDCl$_3$)** δ 7.27 – 7.18 (m, 2H), 7.12 (d, *J* = 8.7 Hz, 1H), 6.71 (d, *J* = 3.3 Hz, 1H), 6.59 (s, 1H), 5.94 (s, 1H), 4.32 (q, *J* = 7.1 Hz, 2H), 1.32 (t, *J* = 7.1 Hz, 3H). **^{13}C NMR (126 MHz, CDCl$_3$)** δ 162.9, 135.7, 135.1, 130.1, 129.0, 124.1, 124.0, 123.8, 122.5, 110.1, 102.5, 62.2, 14.1. **HRMS (ESI)** m/z: $[M + H]^+$ Calcd for $C_{13}H_{12}Cl_2NO_2$ 284.0240; Found 284.0243.

Ethyl 2-(5-methyl-1H-indol-1-yl)acrylate (15)

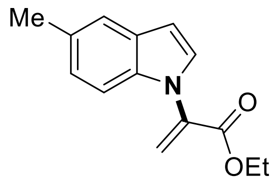

15

Colourless oil; 209 mg, 91% yield; flash chromatography on silica gel. (Hexane/EtOAc = 10:1); **^{1}H NMR (500 MHz, $CDCl_3$)** δ 7.43 (dt, *J* = 1.7, 0.8 Hz, 1H), 7.25 – 7.19 (m, 1H), 7.17 (d, *J* = 3.3 Hz, 1H), 7.05 (dd, *J* = 8.4, 1.7 Hz, 1H), 6.54 (dd, *J* = 3.3, 0.9 Hz, 1H), 6.48 (s, 1H), 5.89 (s, 1H), 4.33 (q, *J* = 7.1 Hz, 2H), 2.46 (d, *J* = 0.8 Hz, 3H), 1.33 (t, *J* = 7.1 Hz, 3H). **^{13}C NMR (126 MHz, $CDCl_3$)** δ 163.6, 136.2, 134.7, 129.8, 129.3, 128.5, 123.9, 120.7, 120.1, 110.4, 103.1, 61.9, 21.3, 14.1. **HRMS (ESI)** m/z: $[M + H]^+$ Calcd for $C_{14}H_{16}NO_2$ 230.1176; Found 230.1173.

Ethyl 2-(5-(tert-butyl)-1H-indol-1-yl)acrylate (16)

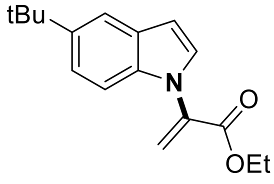

16

Colourless oil; 244 mg, 90% yield; flash chromatography on silica gel. (Hexane/EtOAc = 10:1); **^{1}H NMR (500 MHz, $CDCl_3$)** δ 7.66 (d, *J* = 1.8 Hz, 1H), 7.33 (dd, *J* = 8.8, 1.9 Hz, 1H), 7.29 (d, *J* = 8.7 Hz, 1H), 7.19 (d, *J* = 3.3 Hz, 1H), 6.60 (dd, *J* = 3.3, 0.7 Hz, 1H), 6.49 (s, 1H), 5.91 (s, 1H), 4.35 (q, *J* = 7.1 Hz, 2H), 1.41 (s, 9H), 1.35 (t, *J* = 7.1 Hz, 3H). **^{13}C NMR (126 MHz, $CDCl_3$)** δ 163.6, 143.4, 136.2, 134.4, 128.8, 128.5, 120.6, 120.0, 117.0, 110.2, 103.6, 61.9, 34.5, 31.8, 14.1. **HRMS (ESI)** m/z: $[M + H]^+$ Calcd for $C_{17}H_{22}NO_2$ 272.1645; Found 272.1648.

Ethyl 2-(5-methoxy-1H-indol-1-yl) acrylate (17)

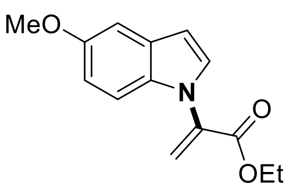

17

Colourless oil; 223 mg, 91% yield; flash chromatography on silica gel. (Hexane/EtOAc = 10:1); **^{1}H NMR (500 MHz, CDCl$_3$)** δ 7.23 (d, *J* = 8.9 Hz, 1H), 7.19 (d, *J* = 3.3 Hz, 1H), 7.11 (d, *J* = 2.5 Hz, 1H), 6.89 (dt, *J* = 9.0, 1.7 Hz, 1H), 6.55 (d, *J* = 3.3 Hz, 1H), 6.47 (s, 1H), 5.89 (s, 1H), 4.34 (q, *J* = 7.1 Hz, 2H), 3.87 (s, 3H), 1.34 (t, *J* = 7.1 Hz, 3H). **^{13}C NMR (126 MHz, CDCl$_3$)** δ 163.5, 154.5, 136.1, 131.4, 129.5, 128.9, 119.9, 112.3, 111.4, 103.2, 102.7, 61.9, 55.7, 14.1. **HRMS (ESI)** m/z: $[M + H]^+$ Calcd for $C_{14}H_{16}NO_3$ 246.1125; Found 246.1122.

Ethyl 2-(5-((3-ethoxy-3-oxoprop-1-en-1-yl) oxy)-1H-indol-1-yl)acrylate (18)

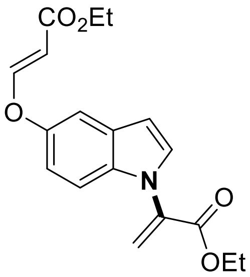

18

Colourless oil; 247 mg, 75% yield; flash chromatography on silica gel. (Hexane/EtOAc = 10:1); **^{1}H NMR (500 MHz, CDCl$_3$)** δ 7.84 (d, *J* = 12.2 Hz, 1H), 7.30 (d, *J* = 2.4 Hz, 1H), 7.26 (dd, *J* = 8.7, 0.8 Hz, 1H), 7.24 (d, *J* = 3.3 Hz, 1H), 6.95 (dd, *J* = 8.9, 2.4 Hz, 1H), 6.58 (dd, *J* = 3.3, 0.9 Hz, 1H), 6.54 (s, 1H), 5.93 (s, 1H), 5.47 (d, *J* = 12.2 Hz, 1H), 4.33 (q, *J* = 7.1 Hz, 2H), 4.18 (q, *J* = 7.2 Hz, 2H), 1.33 (t, *J* = 7.1 Hz, 3H), 1.27 (t, *J* = 7.2 Hz, 3H). **^{13}C NMR (126 MHz, CDCl$_3$)** δ 167.6, 163.3, 161.0, 150.4, 136.0, 133.8, 130.1, 129.5, 121.4, 114.1, 111.6, 109.9, 103.6, 100.9, 62.1, 59.9, 14.3, 14.1. **HRMS (ESI)** m/z: $[M + H]^+$ Calcd for $C_{18}H_{20}NO_5$ 330.1336; Found 330.1330.

Ethyl 2-(5-cyano-1H-indol-1-yl)acrylate (19)

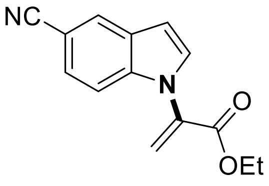

19

Colourless oil; 198 mg, 82% yield; flash chromatography on silica gel. (Hexane/EtOAc = 10:1); **^{1}H NMR (500 MHz, CDCl$_3$)** δ 7.99 – 7.95 (m, 1H), 7.44 (dd, *J* = 8.6, 1.6 Hz, 1H), 7.36 – 7.31 (m, 1H), 7.29 (d, *J* = 3.4 Hz, 1H), 6.68 (dd, *J* = 3.4, 0.9 Hz, 1H), 6.64 (d, *J* = 0.6 Hz, 1H), 5.98 (d, *J* = 0.6 Hz, 1H), 4.33 (q, *J* = 7.1 Hz, 2H), 1.32 (t, *J* = 7.1 Hz, 3H). **^{13}C NMR (126 MHz, CDCl$_3$)** δ 162.8, 138.0, 135.6, 130.8, 128.7, 126.5, 125.4, 123.2, 120.4, 111.5, 104.1, 103.7, 62.3, 14.1. **HRMS (ESI)** m/z: $[M + H]^+$ Calcd for $C_{14}H_{13}N_2O_2$ 241.0972; Found 241.0970.

Methyl 1-(3-ethoxy-3-oxoprop-1-en-2-yl)-1H-indole-5-carboxylate (20)

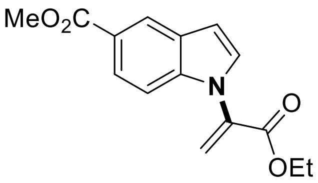

20

Colourless oil; 219 mg, 80% yield; flash chromatography on silica gel. (Hexane/EtOAc = 10:1); **^{1}H NMR (500 MHz, CDCl$_3$)** δ 8.39 (d, *J* = 1.1 Hz, 1H), 7.91 (dd, *J* = 8.7, 1.7 Hz, 1H), 7.30 (d, *J* = 8.8 Hz, 1H), 7.24 (d, *J* = 3.4 Hz, 1H), 6.69 (dd, *J* = 3.3, 0.9 Hz, 1H), 6.59 (s, 1H), 5.97 (s, 1H), 4.32 (q, *J* = 7.1 Hz, 2H), 3.93 (s, 3H), 1.31 (t, *J* = 7.1 Hz, 3H). **^{13}C NMR (126 MHz, CDCl$_3$)** δ 167.9, 163.1, 138.9, 135.8, 129.8, 128.5, 123.9, 123.7, 122.6, 122.2, 110.3, 104.7, 62.1, 51.9, 14.1. **HRMS (ESI)** m/z: $[M + H]^+$ Calcd for $C_{15}H_{16}NO_4$ 274.1074; Found 274.1076.

Ethyl 2-(5-bromo-1H-indol-1-yl)acrylate (21)

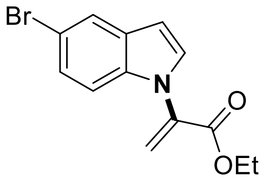

21

Colourless oil; 249 mg, 85% yield; flash chromatography on silica gel. (Hexane/EtOAc = 10:1); **^{1}H NMR (500 MHz, $CDCl_3$)** δ 7.76 (d, *J* = 1.9 Hz, 1H), 7.29 (dd, *J* = 8.8, 1.9 Hz, 1H), 7.21 – 7.14 (m, 2H), 6.55 (s, 2H), 5.91 (s, 1H), 4.32 (q, *J* = 7.1 Hz, 2H), 1.32 (t, *J* = 7.1 Hz, 3H). **^{13}C NMR (126 MHz, $CDCl_3$)** δ 163.2, 135.9, 135.1, 130.6, 129.6, 125.1, 123.5, 121.6, 113.7, 112.1, 103.0, 62.1, 14.1. **HRMS (ESI)** m/z: $[M + H]^+$ Calcd for $C_{13}H_{13}BrNO_2$ 294.0124; Found 294.0126.

Ethyl 2-(5-iodo-1H-indol-1-yl)acrylate (22)

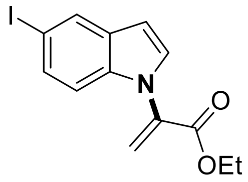

22

Colourless oil; 297 mg, 87% yield; flash chromatography on silica gel. (Hexane/EtOAc = 10:1); **^{1}H NMR (500 MHz, $CDCl_3$)** δ 7.97 (d, *J* = 1.8 Hz, 1H), 7.46 (dd, *J* = 8.6, 1.9 Hz, 1H), 7.15 (d, *J* = 3.4 Hz, 1H), 7.08 (d, *J* = 8.5 Hz, 1H), 6.55 (d, *J* = 1.5 Hz, 1H), 6.54 (d, *J* = 3.1 Hz, 1H), 5.94 – 5.87 (m, 1H), 4.32 (qd, *J* = 7.2, 1.2 Hz, 2H), 1.39 – 1.27 (m, 3H). **^{13}C NMR (126 MHz, $CDCl_3$)** δ 163.1, 135.7, 135.5, 131.4, 130.6, 129.8, 129.2, 121.7, 112.6, 102.7, 84.0, 62.1, 14.1. **HRMS (ESI)** m/z: $[M + H]^+$ Calcd for $C_{13}H_{13}INO_2$ 341.9985; Found 341.9990.

Ethyl 2-(6-chloro-5-fluoro-1H-indol-1-yl)acrylate (23)

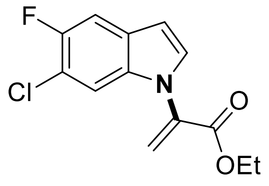

23

Colourless oil; 216 mg, 81% yield; flash chromatography on silica gel. (Hexane/EtOAc = 10:1); **^{1}H NMR (500 MHz, CDCl$_3$)** δ 7.35 (d, *J* = 9.4 Hz, 1H), 7.32 (dd, *J* = 6.1, 0.9 Hz, 1H), 7.22 (d, *J* = 3.3 Hz, 1H), 6.57 (s, 1H), 6.55 (dd, *J* = 3.4, 0.9 Hz, 1H), 5.93 (s, 1H), 4.33 (q, *J* = 7.1 Hz, 2H), 1.34 (t, *J* = 7.2 Hz, 3H). **^{13}C NMR (126 MHz, CDCl$_3$)** δ 163.1, 153.5 (d, J_{C-F} = 239.0 Hz), 135.8, 132.8, 130.6, 127.9 (d, J_{C-F} = 8.9 Hz), 122.2, 116.2 (d, J_{C-F} = 21.4 Hz), 112.1, 107.1 (d, J_{C-F} = 23.4 Hz), 103.6 (d, J_{C-F} = 4.3 Hz), 62.3, 14.2. **^{19}F NMR (471 MHz, CDCl$_3$)** δ -126.05, -126.05. **^{19}F NMR (471 MHz, CDCl$_3$)** δ -126.05, -126.05. **HRMS (ESI)** m/z: [M + H]$^+$ Calcd for $C_{13}H_{12}ClFNO_2$ 268.0535; Found 268.0527.

Ethyl 2-(6-methyl-1H-indol-1-yl)acrylate (24)

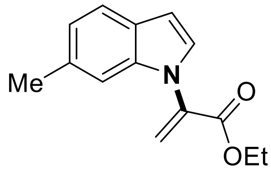

24

Colourless oil; 205 mg, 89% yield; flash chromatography on silica gel. (Hexane/EtOAc = 10:1); **^{1}H NMR (500 MHz, CDCl$_3$)** δ 7.52 (d, *J* = 7.9 Hz, 1H), 7.17 – 7.08 (m, 2H), 6.99 (dd, *J* = 8.2, 1.4 Hz, 1H), 6.57 (dd, *J* = 3.3, 0.9 Hz, 1H), 6.51 (s, 1H), 5.92 (s, 1H), 4.33 (d, *J* = 7.1 Hz, 2H), 2.47 (s, 3H), 1.33 (t, *J* = 7.1 Hz, 3H). **^{13}C NMR (126 MHz, CDCl$_3$)** δ 163.7, 136.8, 136.3, 132.2, 127.9, 126.8, 122.3, 120.8, 120.7, 110.7, 103.4, 62.0, 21.9, 14.2. **HRMS (ESI)** m/z: [M + H]$^+$ Calcd for $C_{14}H_{16}NO_2$ 230.1176; Found 230.1177.

Ethyl 2-(6-methoxy-1H-indol-1-yl)acrylate (25)

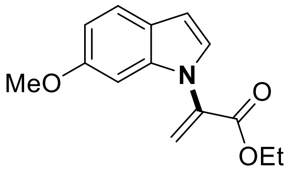

25

Colourless oil; 233 mg, 95% yield; flash chromatography on silica gel. (Hexane/EtOAc = 10:1); **^{1}H NMR (500 MHz, CDCl$_3$)** δ 7.52 (d, *J* = 7.9 Hz, 1H), 7.17 – 7.08 (m, 2H), 6.99 (dd, *J* = 8.2, 1.4 Hz, 1H), 6.57 (dd, *J* = 3.3, 0.9 Hz, 1H), 6.51 (s, 1H), 5.92 (s, 1H), 4.33 (d, *J* = 7.1 Hz, 2H), 2.47 (s, 3H), 1.33 (t, *J* = 7.1 Hz, 3H). **^{13}C NMR (126 MHz, CDCl$_3$)** δ 163.5, 156.6, 137.1, 136.2, 127.4, 123.0, 121.5, 120.9, 110.1, 103.4, 94.6, 61.9, 55.6, 14.1. **HRMS (ESI)** m/z: $[M + H]^+$ Calcd for $C_{14}H_{16}NO_3$ 246.1125; Found 246.1127.

Ethyl 2-(6-(benzyloxy)-1H-indol-1-yl)acrylate (26)

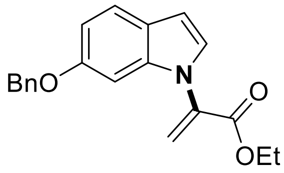

26

Colourless oil; 283 mg, 88% yield; flash chromatography on silica gel. (Hexane/EtOAc = 10:1); **^{1}H NMR (500 MHz, CDCl$_3$)** δ 7.50 (d, *J* = 8.5 Hz, 1H), 7.48 – 7.43 (m, 2H), 7.42 – 7.35 (m, 2H), 7.35 – 7.29 (m, 1H), 7.08 (d, *J* = 3.4 Hz, 1H), 6.92 – 6.85 (m, 2H), 6.54 (dd, *J* = 3.4, 0.9 Hz, 1H), 6.49 (s, 1H), 5.86 (s, 1H), 5.08 (s, 2H), 4.30 (q, *J* = 7.1 Hz, 2H), 1.31 (t, *J* = 7.1 Hz, 3H). **^{13}C NMR (126 MHz, CDCl$_3$)** δ 163.6, 155.8, 137.4, 137.1, 136.2, 128.6, 127.9, 127.7, 127.6, 123.4, 121.6, 120.9, 110.8, 103.5, 96.3, 70.7, 62.0, 14.2. **HRMS (ESI)** m/z: $[M + H]^+$ Calcd for $C_{20}H_{20}NO_3$ $[M + H]^+$ 322.1438; Found 322.1439.

Ethyl 2-(6-cyano-1H-indol-1-yl)acrylate (27)

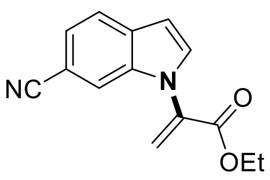

27

White solid; 200 mg, 83% yield; flash chromatography on silica gel. (Hexane/EtOAc = 10:1); m.p. 84–86 ºC; **^{1}H NMR (500 MHz, CDCl$_3$)** δ 7.68 (dd, *J* = 8.2, 0.7 Hz, 1H), 7.63 (q, *J* = 0.9 Hz, 1H), 7.42 – 7.34 (m, 2H), 6.68 (dd, *J* = 3.3, 0.8 Hz, 1H), 6.65 (d, *J* = 0.7 Hz, 1H), 5.98 (d, *J* = 0.7 Hz, 1H), 4.34 (q, *J* = 7.1 Hz, 2H), 1.34 (t, *J* = 7.1 Hz, 3H). **^{13}C NMR (126 MHz, CDCl$_3$)** δ 162.8, 135.3, 132.1, 132.1, 123.4, 123.0, 123.0, 121.8, 120.4, 115.5, 105.0, 104.1, 62.3, 14.1. **HRMS (ESI)** m/z: $[M + H]^+$ Calcd for $C_{14}H_{13}N_2O_2$ 241.0972; Found 241.0970.

Ethyl 2-(6-nitro-1H-indol-1-yl)acrylate (28)

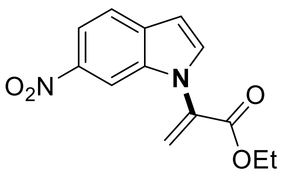

28

Yellowish oil; 200 mg, 77% yield; flash chromatography on silica gel. (Hexane/EtOAc = 8:1); **^{1}H NMR (500 MHz, CDCl$_3$)** δ 8.26 (d, *J* = 2.2 Hz, 1H), 8.05 (dt, *J* = 8.7, 1.7 Hz, 1H), 7.68 (dd, *J* = 8.7, 1.2 Hz, 1H), 7.47 (dd, *J* = 3.3, 1.0 Hz, 1H), 6.72 (dd, *J* = 3.3, 1.0 Hz, 1H), 6.70 (d, *J* = 0.8 Hz, 1H), 6.05 (d, *J* = 0.8 Hz, 1H), 4.35 (qd, *J* = 7.1, 1.1 Hz, 2H), 1.40 – 1.30 (m, 3H). **^{13}C NMR (126 MHz, CDCl$_3$)** δ 162.7, 143.6, 135.2, 134.0, 133.7, 123.5, 121.0, 116.0, 107.5, 104.1, 62.4, 14.1. **HRMS (ESI)** m/z: $[M + H]^+$ Calcd for $C_{13}H_{13}N_2O_4$ 261.0870; Found 261.0871.

Ethyl 2-(6-chloro-1H-indol-1-yl)acrylate (29)

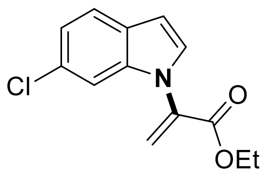

29

Colourless oil; 212 mg, 85% yield; flash chromatography on silica gel. (Hexane/EtOAc = 10:1); **^{1}H NMR (400 MHz, CDCl$_3$)** δ 7.53 (dd, *J* = 8.4, 0.5 Hz, 1H), 7.32 – 7.29 (m, 1H), 7.17 (d, *J* = 3.3 Hz, 1H), 7.12 (dd, *J* = 8.4, 1.8 Hz, 1H), 6.58 (dd, *J* = 3.4, 0.9 Hz, 1H), 6.57 (s, 1H), 5.94 (s, 1H), 4.33 (d, *J* = 7.1 Hz, 2H), 1.33 (t, *J* = 7.1 Hz, 3H). **^{13}C NMR (126 MHz, CDCl$_3$)** δ 163.1, 136.8, 135.8, 129.2, 128.4, 127.4, 121.9, 121.8, 121.2, 110.7, 103.5, 62.1, 14.1. **HRMS (ESI)** m/z: $[M + H]^+$ Calcd for $C_{13}H_{13}ClNO_2$ 250.0629; Found 250.0632.

Ethyl 2-(6-bromo-1H-indol-1-yl)acrylate (30)

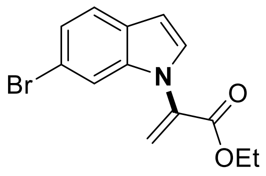

30

Colourless oil; 261 mg, 89% yield; flash chromatography on silica gel. (Hexane/EtOAc = 10:1); **^{1}H NMR (500 MHz, CDCl$_3$)** δ 7.50 – 7.44 (m, 2H), 7.24 (dd, *J* = 8.4, 1.7 Hz, 1H), 7.16 (d, *J* = 3.3 Hz, 1H), 6.60 – 6.54 (m, 2H), 5.94 (s, 1H), 4.33 (q, *J* = 7.1 Hz, 2H), 1.33 (t, *J* = 7.1 Hz, 3H). **^{13}C NMR (126 MHz, CDCl$_3$)** δ 163.1, 137.1, 135.7, 129.1, 127.7, 123.8, 122.2, 122.0, 116.0, 113.7, 103.6, 62.1, 14.1. **HRMS (ESI)** m/z: $[M + H]^+$ Calcd for $C_{13}H_{13}BrNO_2$ 294.0124; Found 294.0127.

Ethyl 2-(7-fluoro-1H-indol-1-yl)acrylate (31)

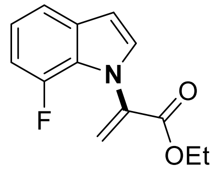

31

Colourless oil; 196 mg, 84% yield; flash chromatography on silica gel. (Hexane/EtOAc = 10:1); **^{1}H NMR (500 MHz, $CDCl_3$)** δ 7.39 (d, J = 7.9 Hz, 1H), 7.09 (d, J = 3.2 Hz, 1H), 7.04 (td, J = 7.9, 4.5 Hz, 1H), 6.90 (dd, J = 12.3, 7.9 Hz, 1H), 6.62 (t, J = 2.9 Hz, 1H), 6.42 (s, 1H), 5.83 (s, 1H), 4.31 (q, J = 7.1 Hz, 2H), 1.27 (t, J = 7.1 Hz, 3H). **^{13}C NMR (126 MHz, $CDCl_3$)** δ163.6, 150.1 (d, J_{C-F} = 244.7 Hz), 138.1, 132.9 (d, J_{C-F} = 4.5 Hz), 130.0, 124.5 (d, J_{C-F} = 9.3 Hz), 121.1, 121.0 (d, J_{C-F} = 6.6 Hz), 117.0 (d, J_{C-F} = 3.7 Hz), 108.3 (d, J_{C-F} = 17.9 Hz), 104.2 (d, J_{C-F} = 1.9 Hz), 62.0, 14.2. **^{19}F NMR (471 MHz, $CDCl_3$)** δ -131.84. **HRMS (ESI)** m/z: $[M + H]^+$ Calcd for $C_{13}H_{13}FNO_2$ 234.0925; Found 234.0929.

Ethyl 2-(7-chloro-1H-indol-1-yl)acrylate (32)

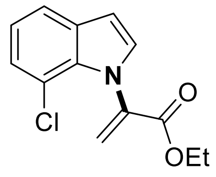

32

Colourless oil; 215 mg, 86% yield; flash chromatography on silica gel. (Hexane/EtOAc = 10:1); **^{1}H NMR (500 MHz, $CDCl_3$)** δ 7.55 (dd, J = 7.8, 1.0 Hz, 1H), 7.19 (dd, J = 7.6, 1.0 Hz, 1H), 7.06 (t, J = 7.7 Hz, 1H), 7.04 (d, J = 3.2 Hz, 1H), 6.62 (d, J = 3.3 Hz, 1H), 6.56 (s, 1H), 5.90 (s, 1H), 4.29 (d, J = 7.1 Hz, 2H), 1.25 (t, J = 7.2 Hz, 3H). **^{13}C NMR (126 MHz, $CDCl_3$)** δ 163.9, 138.9, 132.6, 131.5, 131.3, 123.6, 123.3, 121.2, 120.0, 117.3, 103.7, 62.0, 14.0. **HRMS (ESI)** m/z: $[M + H]^+$ Calcd for $C_{13}H_{13}ClNO_2$ 250.0629; Found 250.0628.

Ethyl 2-(1H-benzo[d]imidazol-1-yl)acrylate (33)

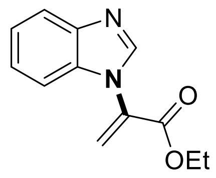

33

Colourless oil; 168 mg,78% yield; flash chromatography on silica gel. (Hexane/EtOAc = 8:1); **^{1}H NMR (400 MHz, $CDCl_3$)** δ 7.92 (s, 1H), 7.74 – 7.64 (m, 1H), 7.24 – 7.21 (m, 1H), 7.20 – 7.16 (m, 2H), 6.49 (d, *J* = 0.8 Hz, 1H), 5.90 (d, *J* = 0.8 Hz, 1H), 4.20 (q, *J* = 7.1 Hz, 2H), 1.19 (t, *J* = 7.1 Hz, 3H). **^{13}C NMR (126 MHz, $CDCl_3$)** δ 162.3, 143.3, 143.0, 133.6, 133.3, 123.7, 122.8, 122.6, 120.4, 110.6, 62.4, 14.0. **HRMS (ESI)** m/z: $[M + H]^+$ Calcd for $C_{12}H_{13}N_2O_2$ 217.0972; Found 217.0975.

Ethyl 2-(1H-indazol-1-yl)acrylate (34)

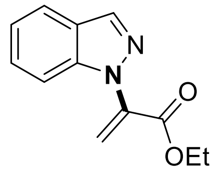

34

Colourless oil; 134 mg, 62% yield; flash chromatography on silica gel. (Hexane/EtOAc = 10:1); **^{1}H NMR (500 MHz, $CDCl_3$)** δ 8.15 (d, *J* = 0.9 Hz, 1H), 7.75 (dt, *J* = 8.1, 1.0 Hz, 1H), 7.45 – 7.33 (m, 2H), 7.20 (ddd, *J* = 7.9, 6.6, 1.2 Hz, 1H), 6.49 (s, 1H), 6.05 (s, 1H), 4.35 (q, *J* = 7.1 Hz, 2H), 1.32 (t, *J* = 7.1 Hz, 3H). **^{13}C NMR (126 MHz, $CDCl_3$)** δ 163.0, 139.8, 137.0, 135.7, 127.0, 124.7, 121.5, 121.1, 121.1, 110.4, 62.0, 14.0. **HRMS (ESI)** m/z: $[M + H]^+$ Calcd for $C_{12}H_{13}N_2O_2$ 217.0972; Found 217.0971.

Ethyl 2-(2,3-dioxoindolin-1-yl)acrylate (35)

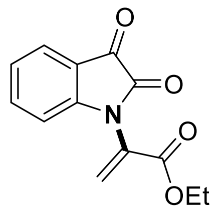

35

Yellowish oil; 184 mg, 75% yield; flash chromatography on silica gel. (Hexane/EtOAc = 8:1);**^{1}H NMR (500 MHz, CDCl$_3$)** δ 7.65 (dt, *J* = 7.5, 1.0 Hz, 1H), 7.57 (td, *J* = 7.8, 1.4 Hz, 1H), 7.16 (td, *J* = 7.5, 0.8 Hz, 1H), 6.78 (d, *J* = 0.8 Hz, 1H), 6.76 (dt, *J* = 7.9, 0.8 Hz, 1H), 6.08 (d, *J* = 0.7 Hz, 1H), 4.27 (q, *J* = 7.1 Hz, 2H), 1.27 (t, *J* = 7.1 Hz, 3H). **^{13}C NMR (126 MHz, CDCl$_3$)** δ 181.8, 161.7, 157.3, 150.8, 138.4, 130.5, 128.6, 125.5, 124.3, 117.4, 111.3, 62.3, 14.0 **HRMS (ESI)** m/z: $[M + H]^+$ Calcd for $C_{13}H_{12}NO_4$ 246.0761; Found 246.0767.

Ethyl 2-(7-methyl-1H-indol-1-yl)acrylate (36)

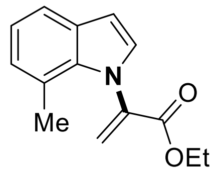

36

Yellowish oil; 34 mg, 15% yield; flash chromatography on silica gel. (Hexane/EtOAc = 15:1); **^{1}H NMR (500 MHz, CDCl$_3$)** δ 7.48 (d, *J* = 7.9 Hz, 1H), 7.04 (dd, *J* = 7.9, 7.1 Hz, 1H), 6.98 (d, *J* = 3.3 Hz, 1H), 6.95 (dt, *J* = 7.0, 1.1 Hz, 1H), 6.59 (s, 1H), 6.58 (d, *J* = 3.3 Hz, 1H), 5.88 (s, 1H), 4.27 (q, *J* = 7.2 Hz, 2H), 2.42 (s, 3H), 1.26 (t, *J* = 7.1 Hz, 3H). **^{13}C NMR (126 MHz, CDCl$_3$)** δ 164.1, 139.3, 130.1, 129.5, 124.9, 124.4, 121.3, 119.03, 103.5, 61.9, 19.1, 14.1. **HRMS (ESI)** m/z: $[M + H]^+$ Calcd for $C_{14}H_{16}NO_2$ 230.1176; Found 230.1174.

Ethyl 2-(2,3-dimethyl-1H-indol-1-yl)acrylate (37)

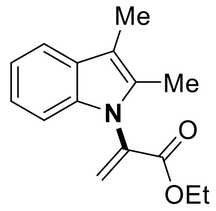

37

Brownish oil; 41 mg, 17% yield; flash chromatography on silica gel. (Hexane/EtOAc = 15:1); **^{1}H NMR (500 MHz, CDCl$_3$)** δ 7.54 – 7.46 (m, 1H), 7.17 – 7.08 (m, 3H), 6.74 (s, 1H), 5.91 (s, 1H), 4.26 (q, *J* = 7.2 Hz, 2H), 2.27 (d, *J* = 0.8 Hz, 3H), 2.23 (d, *J* = 0.8 Hz, 3H), 1.27 (t, *J* = 7.1 Hz, 3H). **^{13}C NMR (126 MHz, CDCl$_3$)** δ 164.0, 137.0, 135.5, 132.5, 129.1, 126.3, 121.3, 119.6, 118.0, 117.9, 109.3, 61.8, 14.1, 10.4, 8.8. **HRMS (ESI)** m/z: $[M + H]^+$ Calcd for $C_{15}H_{18}NO_2$ 244.1332; Found 244.1331.

Ethyl (E)-2-(3-(3-ethoxy-3-oxoprop-1-en-1-yl)-2-phenyl-1H-indol-1-yl)acrylate (38)

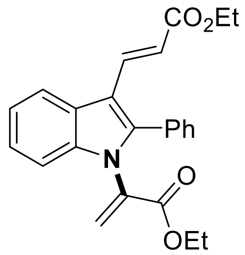

38

Colourless oil; 43 mg, 11% yield; flash chromatography on silica gel. (Hexane/EtOAc = 15:1); **^{1}H NMR (500 MHz, CDCl$_3$)** δ 8.08 (d, *J* = 14.4 Hz, 1H), 7.82 (dt, *J* = 8.4, 0.8 Hz, 1H), 7.58 (ddd, *J* = 7.9, 1.3, 0.7 Hz, 1H), 7.49 – 7.41 (m, 3H), 7.41 – 7.35 (m, 3H), 7.31 (ddd, *J* = 8.0, 7.2, 0.9 Hz, 1H), 6.52 (d, *J* = 1.6 Hz, 1H), 6.08 (d, *J* = 14.4 Hz, 1H), 5.85 (d, *J* = 1.7 Hz, 1H), 4.20 (q, *J* = 7.1 Hz, 2H), 3.92 (q, *J* = 7.1 Hz, 2H), 1.28 (t, *J* = 7.1 Hz, 3H), 1.05 (t, *J* = 7.1 Hz, 3H). **^{13}C NMR (126 MHz, CDCl$_3$)** δ 167.5, 166.6, 138.9, 138.3, 135.6, 133.7, 130.8, 130.3, 129.7, 129.6, 129.0, 128.7, 124.2, 122.9, 120.0, 116.2, 112.7, 104.4, 61.0, 60.3, 14.3, 14.0. **HRMS (ESI)** m/z: $[M + H]^+$ Calcd for $C_{24}H_{24}NO_4$ 390.1700; Found 390.1702.

Table S5 Scope of carbazoles and their analogues (1B).[a]

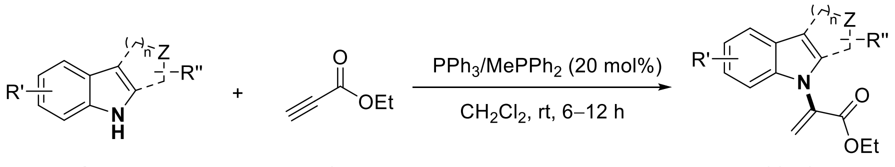

1A **2** **39-53**

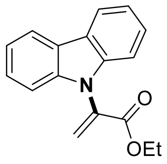

39

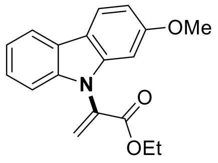

40

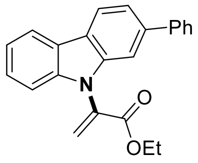

41

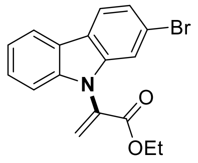

42

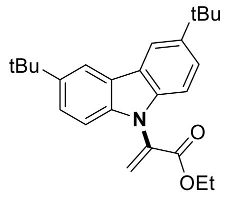

43

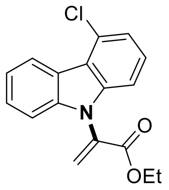

44

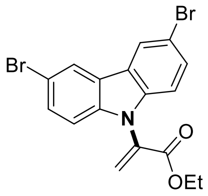

45

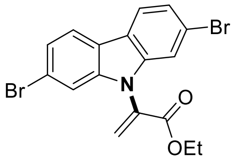

46

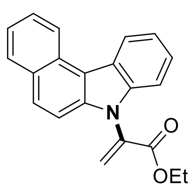

47

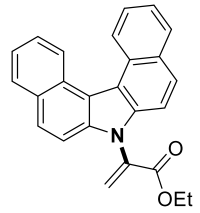

48

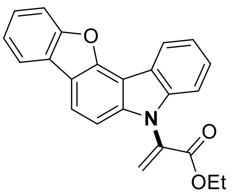

49

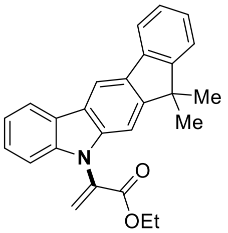

50

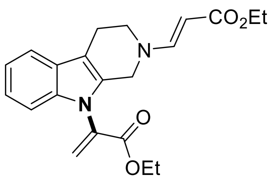

51

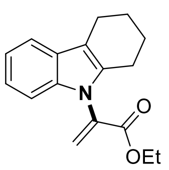

52

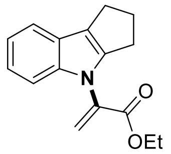

53

[a] Reaction conditions: **1B** (1 mmol), **2a** (1.2 mmol), and the triphenylphosphine catalyst (20 mol%) in CH_2Cl_2 (1 mL) at room temperature. [b] Yields refer to isolated products. [c] 2.1 mmol of **2a** instead. [d] Methyldiphenylphosphine instead.

Ethyl 2-(9H-carbazol-9-yl)acrylate (39)

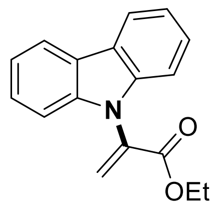

39

Colourless oil; 228 mg, 86% yield; flash chromatography on silica gel. (Hexane/EtOAc = 8:1); **^{1}H NMR (500 MHz, CDCl$_3$)** δ 8.20 – 8.08 (m, 2H), 7.49 (ddd, J = 8.4, 7.3, 1.3 Hz, 2H), 7.42 – 7.25 (m, 4H), 6.92 (s, 1H), 6.14 (s, 1H), 4.29 (q, J = 7.1 Hz, 2H), 1.24 (t, J = 7.1 Hz, 3H). **^{13}C NMR (126 MHz, CDCl$_3$)** δ 163.7, 140.8, 135.0, 126.3, 126.1, 123.6, 120.4, 120.3, 110.0, 61.9, 14.1. **HRMS (ESI)** m/z: $[M + H]^+$ Calcd for $C_{17}H_{16}NO_2$ 266.1176; Found 266.1174.

Ethyl 2-(2-methoxy-9H-carbazol-9-yl)acrylate (40)

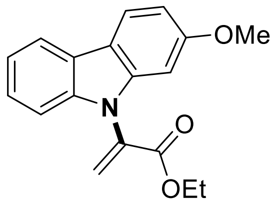

40

Colourless oil; 245 mg, 83% yield; flash chromatography on silica gel. (Hexane/EtOAc = 6:1); **^{1}H NMR (500 MHz, CDCl$_3$)** δ 8.04 – 7.89 (m, 2H), 7.35 (ddd, J = 8.3, 7.2, 1.3 Hz, 1H), 7.23 (ddd, J = 10.1, 7.8, 1.0 Hz, 2H), 6.89 (d, J = 10.3 Hz, 2H), 6.75 (s, 1H), 6.12 (s, 1H), 4.25 (q, J = 7.1 Hz, 2H), 3.88 (s, 3H), 1.20 (t, J = 7.1 Hz, 3H). **^{13}C NMR (126 MHz, CDCl$_3$)** δ 163.6, 159.1, 142.0, 140.8, 134.9, 126.5, 124.6, 123.6, 121.0, 120.3, 119.4, 117.3, 109.6, 108.6, 94.3, 61.8, 55.6, 14.1. **HRMS (ESI)** m/z: $[M + H]^+$ Calcd for $C_{18}H_{18}NO_3$ 296.1281; Found 296.1279.

Ethyl 2-(2-phenyl-9H-carbazol-9-yl)acrylate (41)

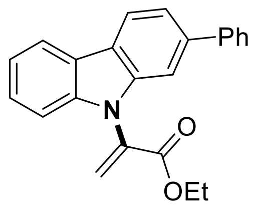

41

Colourless oil; 239 mg, 70% yield; flash chromatography on silica gel. (Hexane/EtOAc = 6:1); **^{1}H NMR (500 MHz, $CDCl_3$)** δ 8.18 – 8.05 (m, 2H), 7.74 – 7.63 (m, 2H), 7.54 (dd, *J* = 8.1, 1.5 Hz, 1H), 7.46 (dt, *J* = 15.8, 7.5 Hz, 4H), 7.40 – 7.33 (m, 1H), 7.33 – 7.23 (m, 2H), 6.93 (s, 1H), 6.17 (s, 1H), 4.26 (q, *J* = 7.1 Hz, 2H), 1.20 (t, *J* = 7.1 Hz, 3H). **^{13}C NMR (126 MHz, $CDCl_3$)** δ 163.5, 141.8, 141.2, 141.1, 139.4, 134.7, 128.7, 127.4, 127.1, 126.7, 126.0, 123.2, 122.7, 120.5, 120.3, 120.3, 119.9, 109.9, 108.3, 61.8, 14.0. **HRMS (ESI)** m/z: $[M + H]^+$ Calcd for $C_{23}H_{20}NO_2$ 342.1489; Found: 342.1483.

Ethyl 2-(2-bromo-9H-carbazol-9-yl)acrylate (42)

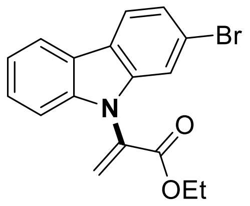

42

Colourless oil; 254 mg,74% yield; flash chromatography on silica gel. (Hexane/EtOAc = 8:1); **^{1}H NMR (500 MHz, $CDCl_3$)** δ 8.05 (dd, *J* = 7.7, 1.1 Hz, 1H), 7.93 (dd, *J* = 8.2, 1.0 Hz, 1H), 7.49 – 7.41 (m, 2H), 7.39 (dd, *J* = 8.3, 1.7 Hz, 1H), 7.32 – 7.24 (m, 2H), 6.92 (d, *J* = 1.0 Hz, 1H), 6.14 (d, *J* = 1.1 Hz, 1H), 4.26 (q, *J* = 7.0 Hz, 2H), 1.22 (t, *J* = 7.2 Hz, 3H). **^{13}C NMR (126 MHz, CDCl3)** δ 163.1, 141.4, 140.8, 134.4, 127.2, 126.4, 123.3, 122.8, 122.4, 121.4, 120.6, 120.2, 119.4, 113.0, 110.0, 62.0, 14.0. **HRMS (ESI)** m/z: $[M + H]^+$ Calcd for $C_{17}H_{15}BrNO_2$ 344.0281; Found: 344.0275.

Ethyl 2-(3,6-di-tert-butyl-9H-carbazol-9-yl)acrylate (43)

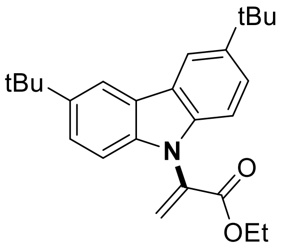

43

White solid; 306 mg, 81% yield; flash chromatography on silica gel. (Hexane/EtOAc = 4:1); m.p. 182–185 °C; **^{1}H NMR (500 MHz, CDCl$_3$)** δ 8.19 – 7.99 (m, 2H), 7.47 (dd, *J* = 8.6, 1.9 Hz, 2H), 7.19 (d, *J* = 8.6 Hz, 2H), 6.79 (s, 1H), 6.05 (s, 1H), 4.26 (q, *J* = 7.1 Hz, 2H), 1.46 (d, *J* = 0.8 Hz, 18H), 1.23 (t, *J* = 7.1 Hz, 3H). **^{13}C NMR (126 MHz, CDCl$_3$)** δ 163.8, 143.0, 139.0, 135.1, 124.9, 123.6, 123.4, 116.2, 109.3, 61.7, 34.7, 32.0, 14.1. **HRMS (ESI)** m/z: $[M + H]^+$ Calcd for $C_{25}H_{32}NO_2$ 378.2428; Found 378.2433.

Ethyl 2-(4-chloro-9H-carbazol-9-yl)acrylate (44)

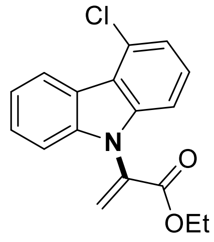

44

Colourless oil; 255 mg, 85% yield; flash chromatography on silica gel. (Hexane/EtOAc = 8:1); **^{1}H NMR (500 MHz, CDCl$_3$)** δ 8.65 (d, *J* = 7.9 Hz, 1H), 7.50 (t, *J* = 7.7 Hz, 1H), 7.35 (t, *J* = 7.9 Hz, 2H), 7.32 – 7.26 (m, 2H), 7.19 (d, *J* = 8.1 Hz, 1H), 6.96 (s, 1H), 6.16 (s, 1H), 4.26 (q, *J* = 7.2 Hz, 2H), 1.21 (t, *J* = 7.1 Hz, 3H). **^{13}C NMR (126 MHz, CDCl$_3$)** δ 163.2, 141.7, 140.7, 134.5, 128.7, 127.5, 126.5, 126.2, 123.1, 122.4, 121.0, 120.8, 120.5, 109.5, 108.2, 61.9, 14.0. **HRMS (ESI)** m/z: $[M + H]^+$ Calcd for $C_{17}H_{15}ClNO_2$ 300.0786; Found 300.0783.

Ethyl 2-(3,6-dibromo-9H-carbazol-9-yl)acrylate (45)

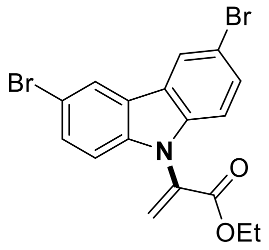

45

White solid; 300 mg, 71% yield flash chromatography on silica gel. (Hexane/EtOAc = 4:1);; m.p. 192–196 °C; **^{1}H NMR (500 MHz, CDCl$_3$)** δ 8.12 (d, *J* = 1.9 Hz, 2H), 7.52 (dd, *J* = 8.7, 2.0 Hz, 2H), 7.13 (d, *J* = 8.6 Hz, 2H), 6.91 (s, 1H), 6.11 (s, 1H), 4.24 (q, *J* = 7.1 Hz, 2H), 1.21 (t, *J* = 7.1 Hz, 3H). **^{13}C NMR (126 MHz, CDCl$_3$)** δ 162.8, 139.6, 134.1, 129.4, 127.4, 124.0, 123.2, 113.4, 111.5, 62.0, 14.0. **HRMS (ESI)** m/z: $[M + H]^+$ Calcd for $C_{17}H_{14}Br_2NO_2$ 421.9386; Found 421.9387.

Ethyl 2-(2,7-dibromo-9H-carbazol-9-yl)acrylate (46)

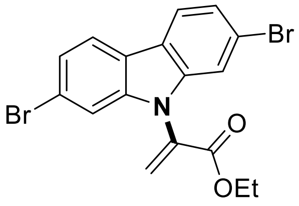

46

White solid; 284 mg, 67% yield; flash chromatography on silica gel. (Hexane/EtOAc = 4:1); m.p. 210–216 °C; **^{1}H NMR (500 MHz, CDCl$_3$)** δ 7.86 (d, *J* = 8.3 Hz, 2H), 7.47 – 7.33 (m, 4H), 6.94 (s, 1H), 6.14 (s, 1H), 4.27 (q, *J* = 7.1 Hz, 2H), 1.24 (t, *J* = 7.1 Hz, 3H). **^{13}C NMR (126 MHz, CDCl$_3$)** δ 162.6, 141.5, 133.9, 128.1, 123.9, 121.8, 121.4, 119.9, 113.1, 62.1, 13.1. **HRMS (ESI)** m/z: $[M + H]^+$ Calcd for $C_{17}H_{14}Br_2NO_2$ 421.9386; Found 421.9387.

Ethyl 2-(7H-benzo[c]carbazol-7-yl)acrylate (47)

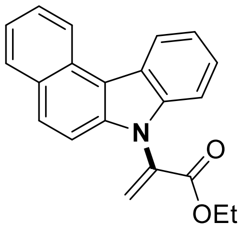

47

Colourless oil; 202 mg, 64% yield; flash chromatography on silica gel. (Hexane/EtOAc = 5:1); **^{1}H NMR (500 MHz, CDCl$_3$)** δ 8.83 (dd, J = 8.4, 1.1 Hz, 1H), 8.66 – 8.57 (m, 1H), 8.07 – 7.98 (m, 1H), 7.89 (d, J = 8.9 Hz, 1H), 7.74 (ddd, J = 8.3, 6.9, 1.4 Hz, 1H), 7.55 – 7.40 (m, 5H), 7.00 (d, J = 1.0 Hz, 1H), 6.19 (d, J = 1.0 Hz, 1H), 4.25 (q, J = 7.1 Hz, 2H), 1.19 (t, J = 7.1 Hz, 3H). **^{13}C NMR (126 MHz, CDCl$_3$)** δ 163.5, 139.8, 138.3, 134.7, 129.7, 129.5, 129.2, 127.5, 127.4, 127.0, 124.5, 124.1, 123.3, 123.3, 122.1, 120.9, 115.8, 111.4, 110.2, 61.9, 14.0. **HRMS (ESI)** m/z: $[M + H]^+$ Calcd for $C_{21}H_{18}NO_2$ 316.1332; Found 316.1338.

Ethyl 2-(7H-dibenzo[c,g]carbazol-7-yl)acrylate (48)

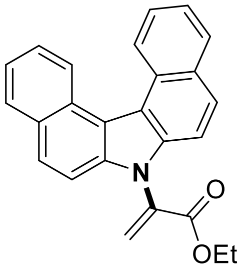

48

Colourless oil; 285 mg,78% yield; flash chromatography on silica gel. (Hexane/EtOAc = 5:1); **^{1}H NMR (500 MHz, CDCl$_3$)** δ 9.27 (d, J = 8.5 Hz, 2H), 8.08 (dd, J = 8.2, 1.4 Hz, 2H), 7.91 (d, J = 8.8 Hz, 2H), 7.74 (ddd, J = 8.4, 6.7, 1.4 Hz, 2H), 7.57 (dd, J = 8.4, 6.7 Hz, 4H), 7.08 (s, 1H), 6.22 (s, 1H), 4.27 (q, J = 7.1 Hz, 2H), 1.20 (t, J = 7.1 Hz, 3H). **^{13}C NMR (126 MHz, CDCl$_3$)** δ 163.4, 137.6, 134.5, 133.8, 133.6, 130.2, 129.1, 129.0, 128.7, 128.6, 128.5, 128.4, 126.9, 125.5, 125.3, 123.5, 117.9, 111.4, 62.0, 14.0. **HRMS (ESI)** m/z: $[M + H]^+$ Calcd for $C_{25}H_{20}NO_2$ 366.1489; Found 366.1489.

Ethyl 2-(5H-benzofuro[3,2-c]carbazol-5-yl)acrylate (49)

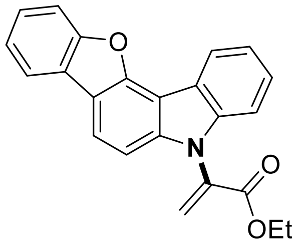

49

White solid; 284 mg, 80% yield; flash chromatography on silica gel. (Hexane/EtOAc = 4:1); m.p. 240–250 ºC; **^{1}H NMR (500 MHz, $CDCl_3$)** δ 8.52 (dt, *J* = 7.7, 1.0 Hz, 1H), 8.02 – 7.90 (m, 2H), 7.72 (dt, *J* = 8.2, 0.9 Hz, 1H), 7.53 – 7.31 (m, 5H), 7.26 (s, 1H), 7.24 (d, *J* = 1.5 Hz, 1H), 6.95 (s, 1H), 4.24 (q, *J* = 7.1 Hz, 2H), 1.18 (t, *J* = 7.1 Hz, 3H). **^{13}C NMR (126 MHz, $CDCl_3$)** δ 163.4, 156.3, 151.1, 141.1, 140.3, 135.0, 127.2, 125.8, 125.4, 125.0, 122.9, 122.7, 121.2, 120.9, 119.7, 118.0, 116.8, 111.7, 109.8, 108.9, 105.5, 61.9, 14.0. **HRMS (ESI)** m/z: $[M + H]^+$ Calcd for $C_{23}H_{18}NO_3$ 356.1281; Found 356.1277.

Ethyl 2-(7,7-dimethylindeno[2,1-b]carbazol-5(7H)-yl)acrylate (50)

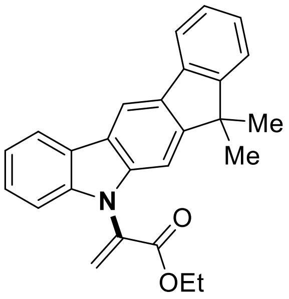

50

White solid; 317 mg, 83% yield; flash chromatography on silica gel. (Hexane/EtOAc = 4:1); m.p. 195–204 ºC; **^{1}H NMR (500 MHz, $CDCl_3$)** δ 8.40 (s, 1H), 8.20 – 8.11 (m, 1H), 7.86 (d, *J* = 7.4 Hz, 1H), 7.49 – 7.35 (m, 3H), 7.34 – 7.23 (m, 4H), 6.94 (s, 1H), 6.17 (s, 1H), 4.26 (q, *J* = 7.2 Hz, 2H), 1.56 (s, 6H), 1.18 (t, *J* = 7.1 Hz, 3H). **^{13}C NMR (126 MHz, $CDCl_3$)** δ 178.9, 168.4, 168.2, 156.2, 156.2, 154.8, 150.1, 147.7, 142.2, 141.6, 141.5, 140.8, 139.0, 138.2, 137.7, 135.4, 135.3, 134.6, 126.5, 125.1, 119.4, 77.0, 61.9, 43.1, 29.2. **HRMS (ESI)** m/z: $[M + H]^+$ Calcd for $C_{26}H_{24}NO_2$ 382.1802; Found 382.1797.

Ethyl (E)-2-(2-(3-ethoxy-3-oxoprop-1-en-1-yl)-1,2,3,4-tetrahydro-9H-pyrido[3,4-b]indol-9-yl)acrylate (51)

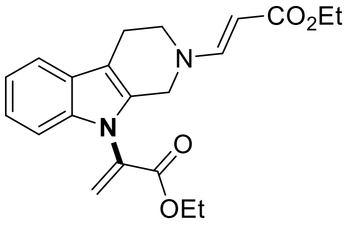

51

Colourless oil; 251 mg, 68% yield; flash chromatography on silica gel. (Hexane/EtOAc = 8:1); **^{1}H NMR (500 MHz, CDCl$_3$)** δ 7.62 (d, *J* = 13.1 Hz, 1H), 7.49 (dt, *J* = 7.6, 1.1 Hz, 1H), 7.22 – 7.12 (m, 3H), 6.75 (s, 1H), 5.98 (s, 1H), 4.73 (d, *J* = 13.1 Hz, 1H), 4.30 (q, *J* = 7.1 Hz, 2H), 4.20 (s, 2H), 4.15 (q, *J* = 7.1 Hz, 2H), 3.64 (t, *J* = 5.7 Hz, 2H), 2.90 (ddd, *J* = 5.7, 4.0, 1.7 Hz, 2H), 1.31 (t, *J* = 7.1 Hz, 3H), 1.27 (t, *J* = 7.1 Hz, 3H). **^{13}C NMR (126 MHz, CDCl$_3$)** δ 169.5, 163.2, 152.0, 137.7, 134.3, 130.3, 126.8, 126.5, 122.4, 120.4, 118.2, 109.9, 109.9, 85.7, 62.2, 59.1, 21.3, 14.6, 14.1. **HRMS (ESI)** m/z: $[M + H]^+$ Calcd for $C_{21}H_{25}N_2O_4$ 369.1809; Found 369.1808.

Ethyl 2-(1,2,3,4-tetrahydro-9H-carbazol-9-yl)acrylate (52)

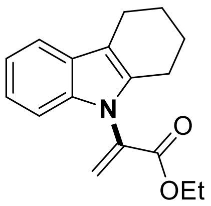

52

Yellowish oil; 175 mg, 65% yield; flash chromatography on silica gel. (Hexane/EtOAc = 10:1); **^{1}H NMR (500 MHz, CDCl$_3$)** δ 7.52 – 7.44 (m, 1H), 7.20 – 7.06 (m, 3H), 6.67 (s, 1H), 5.88 (s, 1H), 4.28 (q, *J* = 7.1 Hz, 2H), 2.79 – 2.72 (m, 2H), 2.58 (dq, *J* = 5.4, 2.8 Hz, 2H), 1.90 (qd, *J* = 4.8, 2.4 Hz, 4H), 1.29 (t, *J* = 7.1 Hz, 3H). **^{13}C NMR (126 MHz, CDCl$_3$)** δ 164.0, 137.1, 135.7, 135.1, 128.0, 125.0, 121.3, 119.7, 117.8, 111.3, 109.6, 61.7, 23.2, 23.0, 22.4, 20.9, 14.1. **HRMS (ESI)** m/z: $[M + H]^+$ Calcd for $C_{17}H_{20}NO_2$ 270.1489; Found 270.1491.

Ethyl 2-(2,3-dihydrocyclopenta[b]indol-4(1H)-yl)acrylate (53)

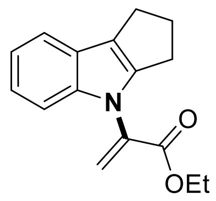

53

Yellowish oil; 135 mg, 53% yield; flash chromatography on silica gel. (Hexane/EtOAc = 10:1); **^{1}H NMR (500 MHz, $CDCl_3$)** δ 7.50 – 7.40 (m, 1H), 7.25 – 7.18 (m, 1H), 7.16 – 7.07 (m, 2H), 6.50 (s, 1H), 5.85 (s, 1H), 4.32 (q, *J* = 7.1 Hz, 2H), 2.92 – 2.84 (m, 2H), 2.84 – 2.76 (m, 2H), 2.58 – 2.50 (m, 2H), 1.32 (t, *J* = 7.1 Hz, 3H). **^{13}C NMR (126 MHz, CDCl3):** δ 163.8, 145.7, 141.5, 135.5, 125.2, 121.4, 120.8, 120.5, 120.2, 118.6, 110.8, 61.8, 28.2, 25.8, 24.5, 14.1. **HRMS (ESI)** m/z: $[M + H]^+$ Calcd for $C_{16}H_{18}NO_2$ 256.1332; Found 256.1333.

Table S6 Scope of bioactive indolic natural product, drugs and dipeptides (1C).[a]

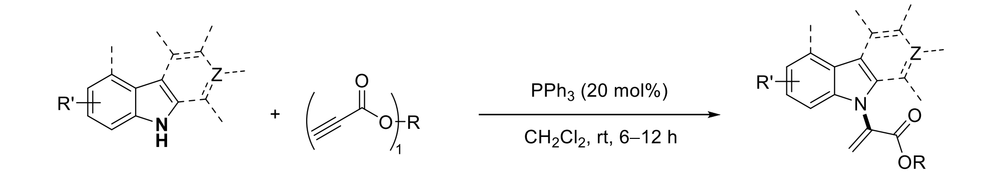

1c 2 54-64

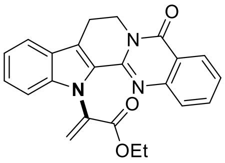

54

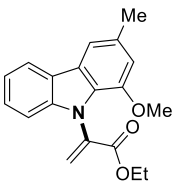

55

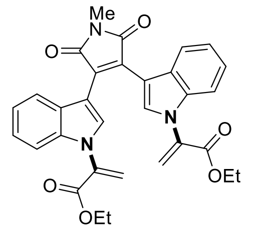

56

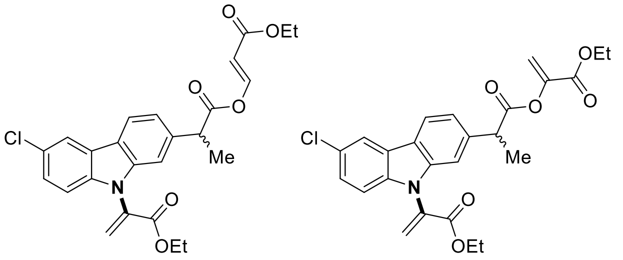

57

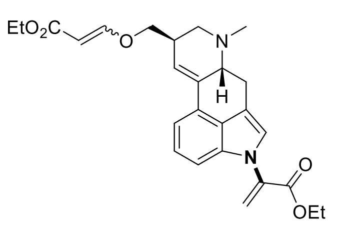

58

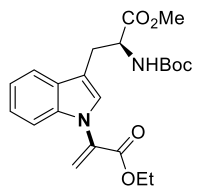

59

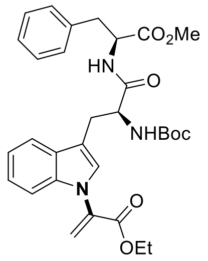

60

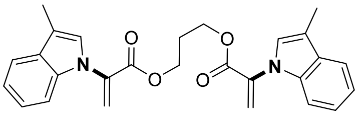

61

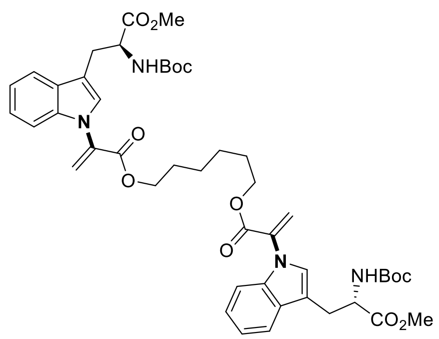

62

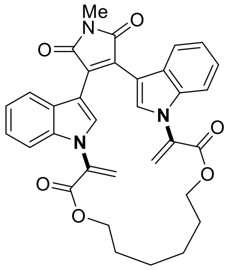

63

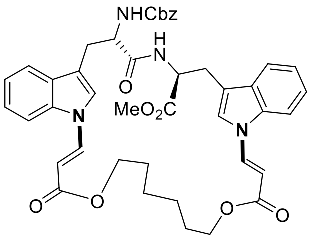

64

[a] Reaction conditions: **1C** (1 mmol), **2** (1.2 mmol), and the triphenylphosphine catalyst (20 mol%) in CH_2Cl_2 (1 mL) at room temperature. [b] Yields refer to isolated products. [c] 2.1 mmol of **2** instead. [e] 2.2 mmol of 1C instead. [f] 1.5 mmol of **1C** in CH_2Cl_2 (10 mL) instead.

From Rutaecarpine (ethyl 2-(5-oxo-7,8-dihydroindolo[2',3':3,4]pyrido[2,1-b]quinazolin-13(5H)-yl)acrylate) (54)

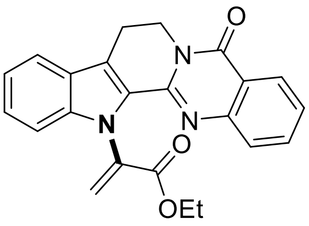

54

While solid; 281 mg, 73% yield; flash chromatography on silica gel. (Hexane/EtOAc = 8:1); m.p. 163–169 °C; **^{1}H NMR (500 MHz, CDCl$_3$)** δ 8.27 (dd, J = 8.0, 1.5 Hz, 1H), 7.72 – 7.62 (m, 2H), 7.59 (dd, J = 8.2, 1.2 Hz, 1H), 7.45 (dd, J = 8.6, 1.0 Hz, 1H), 7.43 – 7.34 (m, 2H), 7.26 – 7.22 (m, 1H), 6.68 (s, 1H), 5.94 (s, 1H), 4.57 (d, J = 237.6 Hz, 2H), 4.22 – 4.00 (m, 2H), 3.25 (s, 2H), 1.11 (t, J = 7.1 Hz, 3H). **^{13}C NMR (126 MHz, CDCl$_3$)** δ 164.3, 161.5, 147.1, 144.6, 139.9, 136.8, 134.1, 127.6, 126.9, 126.9, 126.3, 125.9, 125.2, 121.4, 120.8, 120.5, 120.1, 119.8, 110.9, 61.4, 40.7, 19.6, 14.1. **HRMS (ESI)** m/z: [M + H]$^+$ Calcd for $C_{23}H_{20}N_3O_3$ 386.1499; Found 386.1496.

From Murrayafoline A (ethyl 2-(1-methoxy-3-methyl-9H-carbazol-9-yl)acrylate (55)

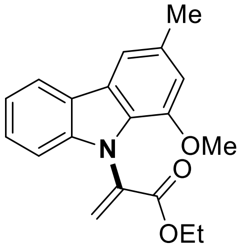

55

Colourless oil; 204 mg, 66% yield; flash chromatography on silica gel. (Hexane/EtOAc = 10:1); **^{1}H NMR (500 MHz, CDCl$_3$)** δ 8.02 (d, J = 7.7 Hz, 1H), 7.49 (s, 1H), 7.45 – 7.37 (m, 2H), 7.24 (ddd, J = 7.9, 5.8, 2.3 Hz, 1H), 6.72 (d, J = 1.3 Hz, 1H), 6.64 (s, 1H), 5.91 (s, 1H), 4.26 – 4.12 (m, 2H), 3.85 (s, 3H), 2.52 (s, 3H), 1.17 (t, J = 7.1 Hz, 3H). **^{13}C NMR (126 MHz, CDCl$_3$)** δ 164.4, 146.3, 140.8, 137.1, 130.4, 128.4, 125.7, 124.9, 123.7, 121.2, 120.3, 119.9, 112.6, 109.5, 109.2, 61.2, 55.5, 21.7, 14.1. **HRMS (ESI)** m/z: [M + H]$^+$ Calcd for $C_{19}H_{20}NO_3$ 310.1438; Found 310.1431.

From Bisindolylmaleimide V (diethyl 2,2'-((1-methyl-2,5-dioxo-2,5-dihydro-1H-pyrrole-3,4-diyl)bis(1H-indole-3,1-diyl))diacrylate) (56)

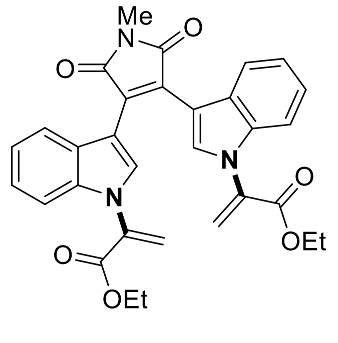

56

Orange oil; 344 mg, 64% yield; flash chromatography on silica gel. (Hexane/EtOAc = 6:1); **^{1}H NMR (500 MHz, CDCl$_3$)** δ 7.74 (s, 2H), 7.24 (dt, *J* = 8.3, 0.9 Hz, 2H), 7.11 (ddd, *J* = 8.3, 7.0, 1.2 Hz, 2H), 7.06 (dt, *J* = 8.1, 0.9 Hz, 2H), 6.83 (ddd, *J* = 8.1, 7.0, 1.0 Hz, 2H), 6.59 (s, 2H), 5.92 (s, 2H), 4.33 (q, *J* = 7.2 Hz, 4H), 3.22 (s, 3H), 1.32 (t, *J* = 7.1 Hz, 6H). **^{13}C NMR (126 MHz, CDCl$_3$)** δ 172.0, 162.9, 136.5, 135.7, 132.1, 127.7, 126.3, 122.9, 122.9, 122.3, 120.9, 110.7, 107.7, 62.1, 24.2, 14.1. **HRMS (ESI)** m/z: $[M + H]^+$ Calcd for $C_{.31}H_{28}N_3O_6$, 538.1973; Found 538.1977.

From Carprofen (ethyl 2-(6-chloro-2-(1-((3-ethoxy-3-oxoprop-1-en-1-yl)oxy)-1-oxopropan-2-yl)-9H-carbazol-9-yl)acrylate) (57)

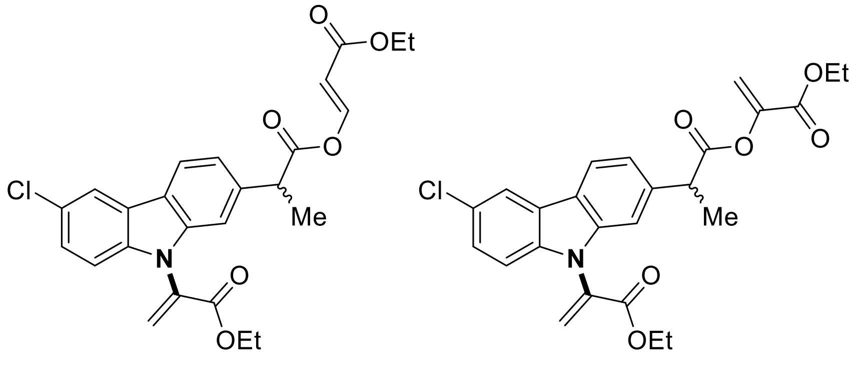

57

Colourless oil (mixture or regioisomers); 273 mg, 59% yield; flash chromatography on silica gel. (Hexane/EtOAc = 8:1); **^{1}H NMR (500 MHz, CDCl$_3$)** δ 8.33 (s, 1H), 8.05 – 7.92 (m, 2H), 7.37 (dd, *J* = 8.6, 2.1 Hz, 1H), 7.22 – 7.08 (m, 3H), 6.92 (s, 1H), 6.37 (d, *J* = 1.2 Hz, 1H), 6.13 (s, 1H), 5.45 (d, *J* = 1.3 Hz, 1H), 4.24 (q, *J* = 7.1 Hz, 2H), 4.18 (q, *J* = 7.0 Hz, 2H), 4.10 (qd, *J* = 7.0, 2.4 Hz, 2H), 1.61 (d, *J* = 7.2 Hz, 3H), 1.24 (t, *J* = 7.1 Hz, 3H), 1.20 (t, *J* = 6.5 Hz, 3H), 1.18 (t, *J* = 6.5 Hz, 3H). **^{13}C NMR (126 MHz, CDCl$_3$)** δ 170.1, 165.7, 165.6, 163.1, 145.2,

141.4, 139.4, 137.5, 134.4, 132.1, 130.3, 127.3, 126.3, 126.0, 124.2, 122.2, 120.9, 120.3, 120.1, 115.2, 111.1, 109.1, 62.1, 61.1, 61.1, 45.8, 18.2, 14.2, 14.1, 14.0. **HRMS (ESI)** m/z: $[M + H]^+$ Calcd for $C_{25}H_{25}ClNO_6$ 470.1365; Found 470.1366.

From Lysergol (ethyl 2-((6aR,9R)-9-((((E)-3-ethoxy-3-oxoprop-1-en-1-yl)oxy)methyl)-7-methyl-6a,7,8,9-tetrahydroindolo[4,3-fg]quinolin-4(6H)-yl)acrylate) (58)

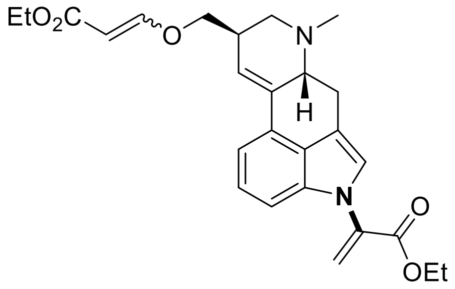

58

Brownish oil; 198 mg, 44% yield (mixture of regioisomer); flash chromatography on silica gel. (Hexane/EtOAc = 6:1); **^{1}H NMR (500 MHz, $CDCl_3$)** δ 7.67 – 7.63 (m, 1H), 7.55 – 7.44 (m, 1H), 7.22 – 7.13 (m, 3H), 6.95 (d, *J* = 1.7 Hz, 1H), 6.37 (s, 1H), 6.30 (d, *J* = 2.4 Hz, 1H), 5.85 (s, 1H), 5.26 (d, *J* = 12.7 Hz, 1H), 4.34 (q, *J* = 7.1 Hz, 2H), 4.18 (q, *J* = 7.1 Hz, 2H), 3.89 (dd, *J* = 9.7, 5.4 Hz, 1H), 3.80 (dd, *J* = 9.7, 7.2 Hz, 1H), 3.51 (dd, *J* = 14.7, 5.4 Hz, 1H), 3.20 – 3.12 (m, 2H), 3.13 – 3.08 (m, 1H), 2.70 (ddd, *J* = 14.7, 11.3, 1.9 Hz, 1H), 2.57 (s, 3H), 2.37 – 2.26 (m, 1H), 1.34 (t, *J* = 7.1 Hz, 3H), 1.28 (t, *J* = 7.1 Hz, 3H). **^{13}C NMR (126 MHz, $CDCl_3$)** δ 167.7, 163.6, 162.2, 136.3, 136.2, 134.5, 132.1, 132.0, 131.9, 131.9, 128.5, 128.5, 128.4, 127.2, 123.7, 122.5, 120.2, 118.2, 113.3, 112.1, 109.7, 96.8, 72.7, 62.7, 61.9, 59.8, 56.1, 43.7, 36.0, 26.8, 14.3, 14.1. **HRMS (ESI)** m/z: $[M + H]^+$ Calcd for $C_{26}H_{31}N_2O_5$ 451.2227; Found 451.2226.

From Boc-Trp-OMe (ethyl(S)-2-(3-(2-((tert-butoxycarbonyl)amino)-3-methoxy-3-oxopropyl)-1H-indol-1-yl)acrylate) (59)

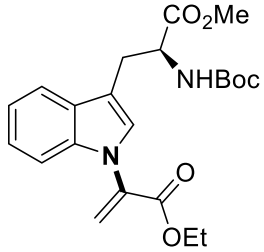

59

Colourless oil; 387 mg, 93% yield; flash chromatography on silica gel. (Hexane/EtOAc = 5:1); **^{1}H NMR (500 MHz, CDCl3)** δ 7.55 (d, J = 7.8 Hz, 1H), 7.32 – 7.27 (m, 1H), 7.21 (ddd, J =

8.3, 7.0, 1.3 Hz, 1H), 7.18 – 7.12 (m, 1H), 7.00 (s, 1H), 6.49 (s, 1H), 5.89 (s, 1H), 5.10 (d, J = 8.3 Hz, 1H), 4.67 (q, J = 6.2 Hz, 1H), 4.32 (q, J = 7.1 Hz, 2H), 3.69 (s, 3H), 3.29 (t, J = 5.6 Hz, 2H), 1.43 (s, 9H), 1.33 (t, J = 7.1 Hz, 3H). **^{13}C NMR (126 MHz, $CDCl_3$)** δ 172.6, 163.4, 155.2, 136.5, 135.8, 128.9, 126.9, 122.6, 120.5, 120.4, 119.1, 111.2, 110.7, 79.8, 62.0, 54.0, 52.3, 28.3, 27.8, 14.1. **HRMS (ESI)** m/z: $[M + H]^+$ Calcd for $C_{22}H_{29}N_2O_6$ 417.2020; Found 417.2020.

he-OMe (ethyl 2-(3-((S)-2-((tert-butoxycarbonyl)amino)-3-(((S)-1-methoxy-1-oxo-3-phenylpropan-2-yl)amino)-3-oxopropyl)-1H-indol-1-yl)acrylate)(60)

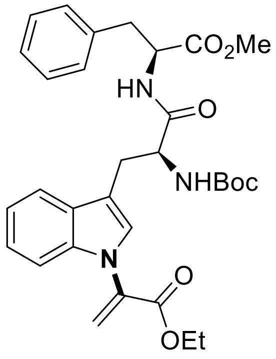

60

Colourless oil; 350 mg, 62% yield; flash chromatography on silica gel. (Hexane/EtOAc = 3:1); **^{1}H NMR (400 MHz, $CDCl_3$)** δ 7.67 (d, *J* = 7.7 Hz, 1H), 7.30 – 7.26 (m, 1H), 7.22 (ddd, *J* = 8.3, 6.9, 1.4 Hz, 1H), 7.19 – 7.10 (m, 4H), 7.08 (s, 1H), 6.89 – 6.78 (m, 2H), 6.46 (s, 1H), 6.33 (s, 1H), 5.86 (s, 1H), 5.21 (s, 1H), 4.72 (q, *J* = 6.4 Hz, 1H), 4.54 – 4.37 (m, 1H), 4.29 (q, *J* = 7.1 Hz, 2H), 3.60 (s, 3H), 3.39 – 3.21 (m, 1H), 3.14 (dd, *J* = 14.5, 7.1 Hz, 1H), 2.95 (d, *J* = 5.9 Hz, 2H), 1.42 (s, 9H), 1.30 (t, *J* = 7.1 Hz, 3H). **^{13}C NMR (101 MHz, $CDCl_3$)** δ 171.2, 171.0, 163.3, 155.2, 136.6, 135.8, 135.6, 129.0, 128.5, 128.3, 127.4, 126.9, 122.6, 120.5, 119.2, 111.5, 110.7, 79.9, 77.2, 61.8, 54.7, 53.2, 52.0, 37.7, 28.1, 28.0, 14.0. **HRMS (ESI)** m/z: $[M + H]^+$ Calcd for $C_{31}H_{38}N_3O_7$ 564.2129; Found 564.2133.

Bis(skatole)-3C (propane-1,3-diyl bis(2-(3-methyl-1H-indol-1-yl)acrylate)) (61)

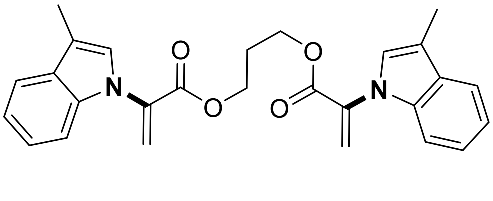

61

Colourless oil; 252 mg, 57% yield; flash chromatography on silica gel. (Hexane/EtOAc = 4:1); **^{1}H NMR (500 MHz, $CDCl_3$)** δ 7.63 – 7.54 (m, 2H), 7.28 (dq, J = 8.1, 1.0 Hz, 2H), 7.22 (ddt, J

= 8.1, 6.8, 1.2 Hz, 2H), 7.18 (ddt, J = 8.0, 7.0, 1.1 Hz, 2H), 6.97 (t, J = 1.2 Hz, 2H), 6.44 (s, 2H), 5.89 (s, 2H), 4.31 (t, J = 6.2 Hz, 4H), 2.35 (s, 6H), 2.07 (p, J = 6.2 Hz, 2H). **^{13}C NMR (126 MHz, CDCl$_3$)** δ 163.6, 136.5, 135.8, 129.6, 125.7, 122.4, 120.0, 120.0, 119.1, 112.9, 110.5, 62.1, 27.7, 9.5. **HRMS (ESI)** m/z: $[M + H]^+$ Calcd for $C_{27}H_{27}N_2O_4$ 443.1965; Found 443.1950.

Bis(tryptophan)-6C (hexane-1,6-diyl bis(2-(3-((S)-2-((tert-butoxycarbonyl)amino)-3-methoxy-3-oxopropyl)-1H-indol-1-yl)acrylate)) (62)

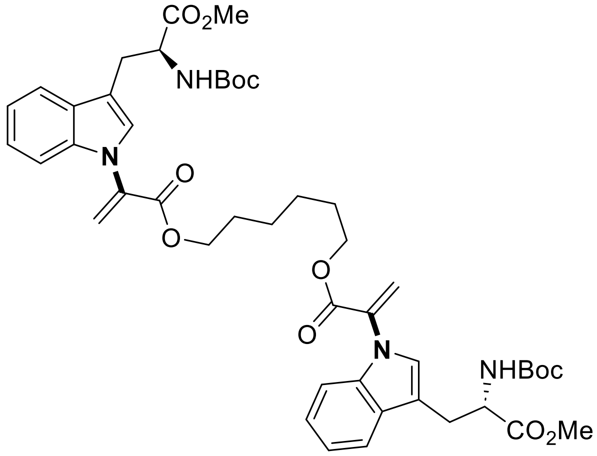

62

Colourless oil; 533 mg, 62% yield; flash chromatography on silica gel. (Hexane/EtOAc = 4:1); **^{1}H NMR (500 MHz, CDCl$_3$)** δ 7.54 (d, *J* = 7.8 Hz, 2H), 7.27 (d, *J* = 8.2 Hz, 2H), 7.20 (ddd, *J* = 8.2, 7.0, 1.2 Hz, 2H), 7.14 (t, *J* = 7.4 Hz, 2H), 6.99 (s, 2H), 6.47 (s, 2H), 5.89 (s, 2H), 5.11 (d, *J* = 8.3 Hz, 2H), 4.66 (q, *J* = 6.2 Hz, 2H), 4.22 (t, *J* = 6.7 Hz, 4H), 3.69 (s, 6H), 3.28 (dq, *J* = 15.8, 7.2 Hz, 4H), 1.65 (t, *J* = 6.7 Hz, 4H), 1.43 (s, 18H), 1.32 (t, *J* = 3.9 Hz, 4H). **^{13}C NMR (126 MHz, CDCl$_3$)** δ 171.9, 163.1, 137.2, 135.5, 132.0, 128.4, 125.6, 123.7, 123.2, 122.7, 121.4, 110.8, 107.5, 66.2, 27.7, 25.6, 24.2. **HRMS (ESI)** m/z: $[M + H]^+$ Calcd for $C_{46}H_{59}N_4O_{12}$ 859.4124; Found 859.4129.

BisindolylmaleimideV-6C ((1^2E,3^2E)-21-methyl-4,15-dimethylene-2^2,2^5-dihydro-1^1H,2^1H,3^1H-6,13-dioxa-1,3(3,1)-diindola-2(3,4)-pyrrolacyclopentadecaphane-2^2,2^5,5,14-tetraone) (63)

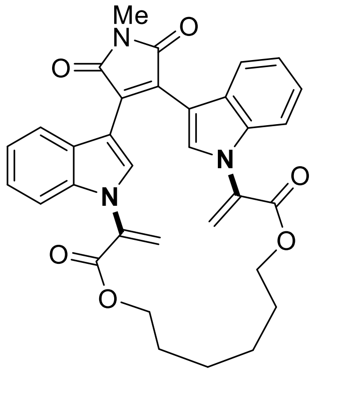

63

Orange oil; 304 mg, 54% yield; flash chromatography on silica gel. (Hexane/EtOAc = 3:1); **^{1}H NMR (500 MHz, CDCl$_3$)** δ 7.49 (s, 2H), 7.29 (ddt, *J* = 8.2, 4.0, 0.9 Hz, 4H), 7.22 (ddd, *J* = 8.2, 7.0, 1.2 Hz, 2H), 7.04 (ddd, *J* = 8.1, 7.0, 1.0 Hz, 2H), 6.65 (s, 2H), 6.00 (s, 2H), 4.02 – 3.91 (m, 4H), 3.21 (s, 3H), 1.33 (p, *J* = 6.8 Hz, 4H), 0.94 (p, *J* = 3.5 Hz, 4H). **^{13}C NMR (126 MHz, CDCl$_3$)** δ 171.9, 163.1, 137.2, 135.5, 132.0, 128.4, 125.6, 123.7, 123.2, 122.7, 121.4, 110.8, 107.5, 66.2, 27.7, 25.6, 24.2. **HRMS (ESI)** m/z: $[M + H]^+$ Calcd for $C_{33}H_{30}N_3O_6$ 564.2129; Found 564.2133

Z-Trp-Trp-OMe-6C (methyl (11S,12Z,81R,82Z,3S,6S,9E,21E)-6-(((benzyloxy)carbonyl)amino)-5,11,20-trioxo-11H,81H-12,19-dioxa-4-aza-1,8(3,1)-diindolacyclodocosaphane-9,21-diene-3-carboxylate) (64)

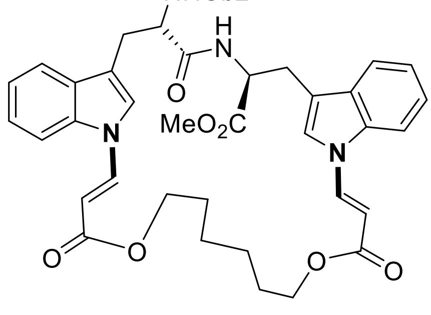

64

Colourless oil; 388 mg, 51% yield (mixture of regioisomers); flash chromatography on silica gel. (Hexane/EtOAc = 5:1→2:1); **^{1}H NMR (500 MHz, Acetone-*d$_6$*)** δ 7.57 (dt, *J* = 8.0, 1.0 Hz, 1H), 7.37 – 7.32 (m, 1H), 7.32 – 7.27 (m, 2H), 7.25 – 7.17 (m, 2H), 7.12 (dddd, *J* = 7.9, 7.0, 3.5, 1.0 Hz, 1H), 6.48 (d, *J* = 13.9 Hz, 1H), 5.95 (d, *J* = 26.6 Hz, 1H), 5.12 – 4.94 (m, 1H), 4.23

– 4.08 (m, 2H), 3.65 (s, 1H), 3.32 (dd, J = 14.9, 4.8 Hz, 1H), 3.25 (d, J = 6.3 Hz, 1H), 2.92 (s, 2H), 1.98 (s, 1H), 1.55 (dt, J = 18.8, 6.7 Hz, 2H), 1.21 – 1.10 (m, 2H). **^{13}C NMR (126 MHz, Acetone-d_6)** δ 171.8, 171.0, 163.5, 163.4, 156.1, 137.2, 136.7, 136.7, 136.2, 135.8, 128.9, 128.5, 128.3, 128.0, 127.7, 127.7, 127.3, 122.4, 122.4, 120.3, 120.2, 120.2, 120.1, 119.1, 118.7, 112.4, 111.0, 110.6, 110.6, 66.0, 65.5, 65.2, 55.6, 52.2, 51.6, 28.0, 27.9, 27.5, 26.8, 25.1, 24.9. **HRMS (ESI)** m/z: $[M + H]^+$ Calcd for $C_{43}H_{45}N_4O_9$ 761.3181; Found 761.3184. **LCMS** (Phenomenex Gemini® 5um C18 110 Å 2.0 x 100 mm, H_2O, 0.2% FA/MeCN, 0.1% FA = gradient program, flow rate = 0.20 mL/min, ESI mass range = 50–1500 amu) tR = 19.196 min.

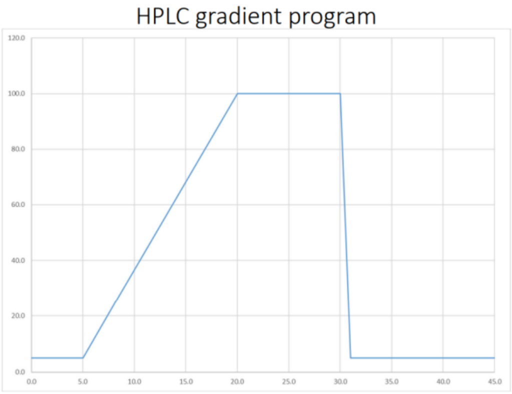

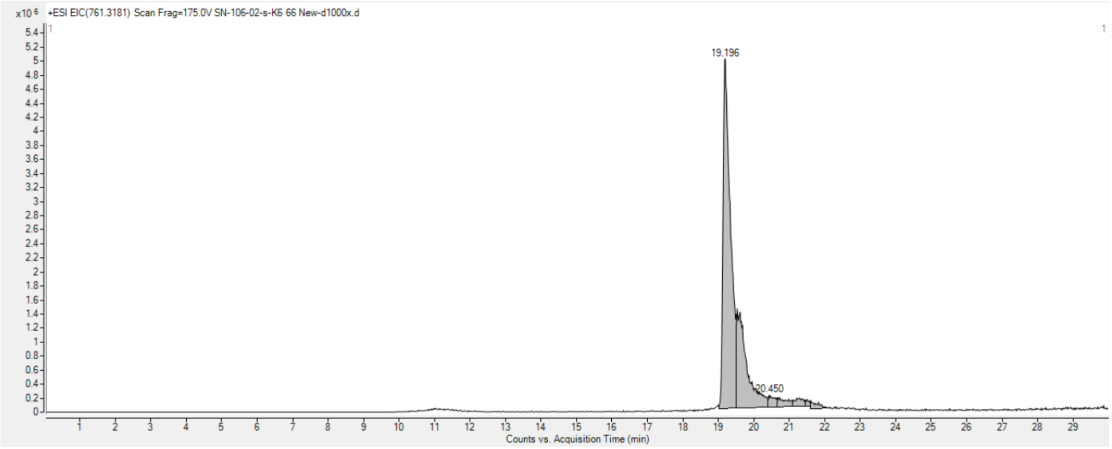

(c) Post-LSF thio-ene diversification of product **59**.

Boc-Trp-OMe-Cys (Ethyl 2-(3-((S)-2-((tert-butoxycarbonyl)amino)-3-methoxy-3-oxopropyl)indolin-1-yl)-3-(((S)-2-((tert-butoxycarbonyl)amino)-3-methoxy-3-oxopropyl)thio)propanoate) (65)

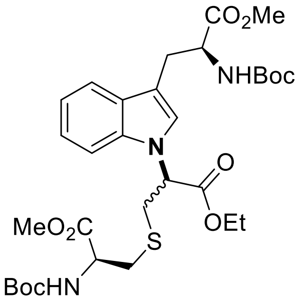

65

Colourless oil; 613 mg, 94% yield; flash chromatography on silica gel. (Hexane/EtOAc = 4:1); **^{1}H NMR (500 MHz, CDCl$_3$)** δ 7.53 (dq, *J* = 7.9, 1.2 Hz, 1H), 7.34 (dd, *J* = 8.2, 2.4 Hz, 1H), 7.22 (tdd, *J* = 8.3, 2.3, 1.2 Hz, 1H), 7.12 (t, *J* = 7.5 Hz, 1H), 7.09 – 6.99 (m, 1H), 5.34 (d, *J* = 7.9 Hz, 1H), 5.22 – 4.98 (m, 2H), 4.63 (d, *J* = 8.7 Hz, 1H), 4.44 (d, *J* = 19.3 Hz, 1H), 4.26 – 4.14 (m, 2H), 3.72 – 3.63 (m, 6H), 3.37 – 3.22 (m, 3H), 3.15 (ddd, *J* = 14.0, 11.3, 8.2 Hz, 1H), 2.89 – 2.56 (m, 2H), 1.42 (d, *J* = 7.9 Hz, 18H), 1.23 (td, *J* = 7.1, 3.2 Hz, 3H). **^{13}C NMR (126 MHz, CDCl$_3$)** δ 172.6, 172.5, 171.3, 171.2, 169.1, 155.2, 155.1, 136.4, 128.5, 128.4, 124.4, 124.4, 122.4, 122.4, 120.0, 120.0, 119.3, 119.3, 110.9, 110.8, 109.4, 80.3, 79.8, 79.8, 62.1, 60.4, 59.1, 58.8, 54.3, 54.1, 53.4, 53.3, 52.6, 52.3, 35.0, 34.7, 34.2, 34.2, 28.4, 28.3, 28.0, 27.9. **HRMS (ESI)** m/z: Calcd $[M + H]^+$ for $C_{31}H_{46}N_3O_{10}S$ 652.2904; Found 652.2894.

D. Crystal Structure of 8

The Cambridge Crystallographic Data Centre (CCDC) has received the crystallographic data for compound **8** (CCDC 2417822).

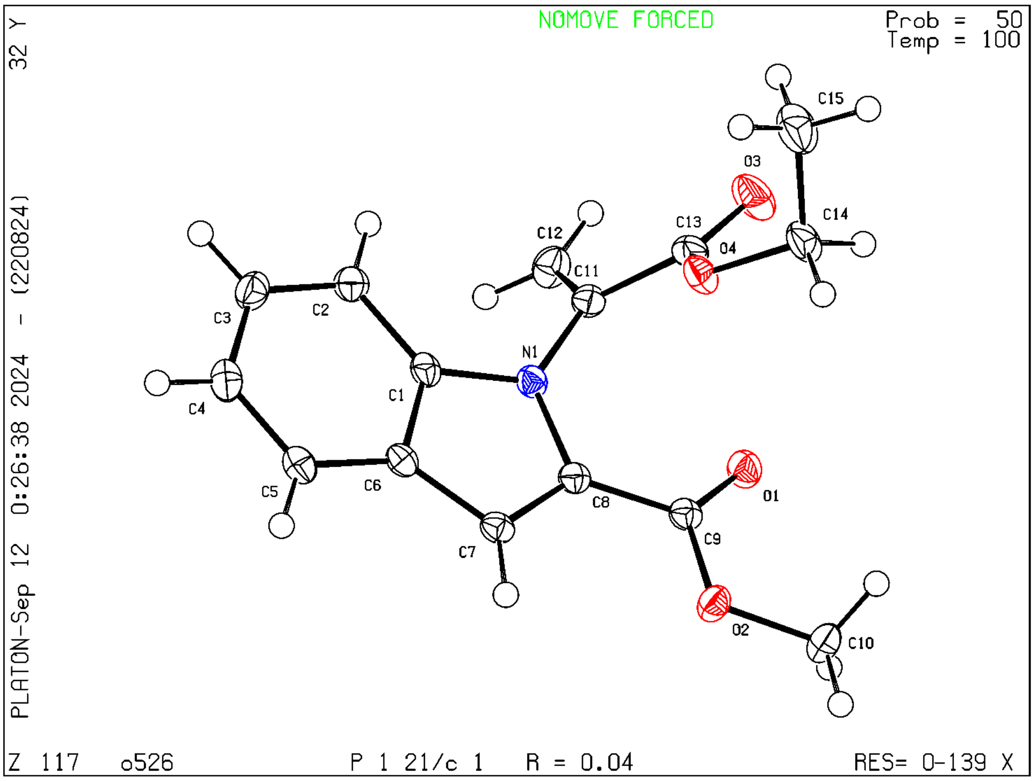

<u>**Single Crystal Structure X-ray Analysis**</u>

Sample Code: O526

Sample ID: Ku-0911

Student/Researcher: Kulice Chan Wai Lun

Supervisor: Lu Yixin

CDCC:

Date: 12-09-2024

Note: The sample crystallizes in a monoclinic space group $P2_1/c$. The asymmetric unit comprises an indole with molecular formula of $C_{15}H_{15}NO_4$. Final R values are R_1=0.0360 and wR_2=0.0886 for 2-theta up to 133.2°.

Table S7 Crystal data and structure refinement for O526.

Identification code	O526	
Empirical formula	C15 H15 N O4	
Formula weight	273.28	
Temperature	100.00 K	
Wavelength	1.54178 Å	
Crystal system	Monoclinic	
Space group	P 1 21/c 1	
Unit cell dimensions	a = 8.3961(8) Å	a= 90°.
	b = 19.0545(17) Å	b= 95.321(2)°.
	c = 8.4533(8) Å	g = 90°.
Volume	1346.6(2) $Å^3$	
Z	4	
Density (calculated)	1.348 Mg/m^3	
Absorption coefficient	0.816 mm^{-1}	
F(000)	576	
Crystal size	0.202 x 0.197 x 0.175 mm^3	
Theta range for data collection	5.746 to 66.589°.	
Index ranges	-9<=h<=9, -22<=k<=22, -10<=l<=10	
Reflections collected	20664	
Independent reflections	2352 [R(int) = 0.0342]	
Completeness to theta = 66.589°	99.1 %	
Absorption correction	Semi-empirical from equivalents	
Max. and min. transmission	0.7533 and 0.6879	
Refinement method	Full-matrix least-squares on F^2	
Data / restraints / parameters	2352 / 0 / 183	
Goodness-of-fit on F^2	1.088	
Final R indices [I>2sigma(I)]	R1 = 0.0360, wR2 = 0.0886	
R indices (all data)	R1 = 0.0361, wR2 = 0.0886	
Extinction coefficient	n/a	
Largest diff. peak and hole	0.228 and -0.279 e.$Å^{-3}$	

Table S8 Atomic coordinates (x 10^4) and equivalent isotropic displacement parameters ($Å^2$x 10^3) for O526. U(eq) is defined as one third of the trace of the orthogonalized U^{ij} tensor.

	x	y	z	U(eq)
O(1)	2351(1)	4671(1)	2420(1)	21(1)
O(2)	4885(1)	4649(1)	1751(1)	20(1)
O(3)	-842(1)	5712(1)	2200(1)	31(1)
O(4)	1452(1)	6130(1)	1387(1)	20(1)
N(1)	3114(1)	5925(1)	4223(1)	16(1)
C(1)	3940(1)	6466(1)	5031(1)	16(1)
C(2)	3368(2)	7015(1)	5921(1)	20(1)
C(3)	4460(2)	7510(1)	6522(2)	22(1)
C(4)	6089(2)	7464(1)	6270(1)	22(1)
C(5)	6652(2)	6921(1)	5398(1)	20(1)
C(6)	5565(1)	6410(1)	4750(1)	16(1)
C(7)	5705(1)	5817(1)	3742(1)	16(1)
C(8)	4210(1)	5538(1)	3434(1)	15(1)
C(9)	3690(1)	4914(1)	2505(1)	16(1)
C(10)	4455(2)	4041(1)	769(2)	22(1)
C(11)	1420(1)	5849(1)	4096(1)	18(1)
C(12)	630(2)	5774(1)	5368(2)	24(1)
C(13)	537(1)	5884(1)	2472(2)	20(1)
C(14)	760(2)	6102(1)	-264(1)	25(1)
C(15)	-128(2)	6760(1)	-736(2)	34(1)

Table S9 Bond lengths [Å] and angles [°] for O526.

O(1)-C(9)	1.2116(15)
O(2)-C(9)	1.3364(14)
O(2)-C(10)	1.4518(14)
O(3)-C(13)	1.2051(16)
O(4)-C(13)	1.3347(15)
O(4)-C(14)	1.4624(14)
N(1)-C(1)	1.3867(15)
N(1)-C(8)	1.3956(15)
N(1)-C(11)	1.4236(15)

C(1)-C(2)	1.3993(17)
C(1)-C(6)	1.4107(17)
C(2)-H(2)	0.9500
C(2)-C(3)	1.3798(18)
C(3)-H(3)	0.9500
C(3)-C(4)	1.4061(19)
C(4)-H(4)	0.9500
C(4)-C(5)	1.3790(18)
C(5)-H(5)	0.9500
C(5)-C(6)	1.4097(17)
C(6)-C(7)	1.4263(17)
C(7)-H(7)	0.9500
C(7)-C(8)	1.3653(17)
C(8)-C(9)	1.4707(16)
C(10)-H(10A)	0.9800
C(10)-H(10B)	0.9800
C(10)-H(10C)	0.9800
C(11)-C(12)	1.3219(18)
C(11)-C(13)	1.5001(17)
C(12)-H(12A)	0.9500
C(12)-H(12B)	0.9500
C(14)-H(14A)	0.9900
C(14)-H(14B)	0.9900
C(14)-C(15)	1.495(2)
C(15)-H(15A)	0.9800
C(15)-H(15B)	0.9800
C(15)-H(15C)	0.9800

C(9)-O(2)-C(10)	114.57(9)
C(13)-O(4)-C(14)	116.04(10)
C(1)-N(1)-C(8)	107.78(10)
C(1)-N(1)-C(11)	124.28(10)
C(8)-N(1)-C(11)	127.53(10)
N(1)-C(1)-C(2)	129.76(11)
N(1)-C(1)-C(6)	107.99(10)
C(2)-C(1)-C(6)	122.17(11)
C(1)-C(2)-H(2)	121.3

C(3)-C(2)-C(1)	117.35(12)
C(3)-C(2)-H(2)	121.3
C(2)-C(3)-H(3)	119.2
C(2)-C(3)-C(4)	121.63(12)
C(4)-C(3)-H(3)	119.2
C(3)-C(4)-H(4)	119.5
C(5)-C(4)-C(3)	120.93(12)
C(5)-C(4)-H(4)	119.5
C(4)-C(5)-H(5)	120.5
C(4)-C(5)-C(6)	118.96(12)
C(6)-C(5)-H(5)	120.5
C(1)-C(6)-C(7)	107.12(10)
C(5)-C(6)-C(1)	118.96(11)
C(5)-C(6)-C(7)	133.86(11)
C(6)-C(7)-H(7)	126.4
C(8)-C(7)-C(6)	107.29(10)
C(8)-C(7)-H(7)	126.4
N(1)-C(8)-C(9)	120.38(10)
C(7)-C(8)-N(1)	109.82(10)
C(7)-C(8)-C(9)	129.75(11)
O(1)-C(9)-O(2)	124.17(11)
O(1)-C(9)-C(8)	124.67(11)
O(2)-C(9)-C(8)	111.16(10)
O(2)-C(10)-H(10A)	109.5
O(2)-C(10)-H(10B)	109.5
O(2)-C(10)-H(10C)	109.5
H(10A)-C(10)-H(10B)	109.5
H(10A)-C(10)-H(10C)	109.5
H(10B)-C(10)-H(10C)	109.5
N(1)-C(11)-C(13)	118.02(10)
C(12)-C(11)-N(1)	121.51(11)
C(12)-C(11)-C(13)	120.44(11)
C(11)-C(12)-H(12A)	120.0
C(11)-C(12)-H(12B)	120.0
H(12A)-C(12)-H(12B)	120.0
O(3)-C(13)-O(4)	124.70(11)
O(3)-C(13)-C(11)	123.14(11)

O(4)-C(13)-C(11)	112.16(10)
O(4)-C(14)-H(14A)	109.3
O(4)-C(14)-H(14B)	109.3
O(4)-C(14)-C(15)	111.62(11)
H(14A)-C(14)-H(14B)	108.0
C(15)-C(14)-H(14A)	109.3
C(15)-C(14)-H(14B)	109.3
C(14)-C(15)-H(15A)	109.5
C(14)-C(15)-H(15B)	109.5
C(14)-C(15)-H(15C)	109.5
H(15A)-C(15)-H(15B)	109.5
H(15A)-C(15)-H(15C)	109.5
H(15B)-C(15)-H(15C)	109.5

Symmetry transformations used to generate equivalent atoms:

#1 x+1,y,z #2 -x,-y+1,-z

Table S10 Anisotropic displacement parameters ($Å^2$x 10^3) for O526. The anisotropic displacement factor exponent takes the form: $-2p^2[h^2 a^{*2}U^{11} + ... + 2 h k a^* b^* U^{12}]$

	U^{11}	U^{22}	U^{33}	U^{23}	U^{13}	U^{12}
O(1)	17(1)	23(1)	23(1)	-3(1)	2(1)	-3(1)
O(2)	19(1)	20(1)	21(1)	-6(1)	5(1)	-2(1)
O(3)	14(1)	47(1)	31(1)	0(1)	0(1)	-4(1)
O(4)	16(1)	30(1)	15(1)	1(1)	-1(1)	-1(1)
N(1)	14(1)	19(1)	14(1)	-1(1)	1(1)	0(1)
C(1)	19(1)	18(1)	11(1)	2(1)	-2(1)	0(1)
C(2)	21(1)	23(1)	16(1)	0(1)	1(1)	3(1)
C(3)	30(1)	20(1)	17(1)	-3(1)	0(1)	3(1)
C(4)	27(1)	20(1)	18(1)	0(1)	-3(1)	-4(1)
C(5)	19(1)	22(1)	18(1)	2(1)	-2(1)	-2(1)
C(6)	17(1)	18(1)	12(1)	3(1)	-1(1)	1(1)
C(7)	16(1)	19(1)	14(1)	3(1)	1(1)	2(1)
C(8)	16(1)	17(1)	12(1)	2(1)	2(1)	2(1)
C(9)	17(1)	18(1)	13(1)	3(1)	1(1)	2(1)
C(10)	25(1)	19(1)	22(1)	-6(1)	5(1)	-1(1)

C(11)	15(1)	19(1)	19(1)	-1(1)	3(1)	0(1)
C(12)	24(1)	25(1)	23(1)	-3(1)	7(1)	-3(1)
C(13)	14(1)	24(1)	21(1)	-1(1)	2(1)	2(1)
C(14)	22(1)	39(1)	14(1)	-2(1)	-3(1)	3(1)
C(15)	39(1)	34(1)	28(1)	5(1)	-11(1)	-3(1)

Table S11 Hydrogen coordinates (x 10^4) and isotropic displacement parameters (Å^{2}x 10 ^{3}) for O526.

	x	y	z	U(eq)
H(2)	2271	7045	6105	24
H(3)	4103	7892	7120	27
H(4)	6812	7812	6707	27
H(5)	7755	6892	5237	23
H(7)	6660	5648	3356	19
H(10A)	3539	4156	10	33
H(10B)	4171	3651	1444	33
H(10C)	5365	3904	189	33
H(12A)	1193	5769	6395	28
H(12B)	-500	5724	5253	28
H(14A)	20	5697	-401	30
H(14B)	1624	6030	-968	30
H(15A)	-597	6719	-1838	51
H(15B)	612	7159	-644	51
H(15C)	-981	6834	-37	51

Table S12 Torsion angles [°] for O526.

N(1)-C(1)-C(2)-C(3)	-176.05(11)
N(1)-C(1)-C(6)-C(5)	177.55(10)
N(1)-C(1)-C(6)-C(7)	0.06(12)
N(1)-C(8)-C(9)-O(1)	-4.06(17)
N(1)-C(8)-C(9)-O(2)	175.47(10)

N(1)-C(11)-C(13)-O(3)	167.22(12)
N(1)-C(11)-C(13)-O(4)	-13.26(15)
C(1)-N(1)-C(8)-C(7)	0.75(13)
C(1)-N(1)-C(8)-C(9)	178.23(10)
C(1)-N(1)-C(11)-C(12)	-60.34(16)
C(1)-N(1)-C(11)-C(13)	117.39(12)
C(1)-C(2)-C(3)-C(4)	-0.77(18)
C(1)-C(6)-C(7)-C(8)	0.40(13)
C(2)-C(1)-C(6)-C(5)	0.55(17)
C(2)-C(1)-C(6)-C(7)	-176.94(10)
C(2)-C(3)-C(4)-C(5)	0.51(19)
C(3)-C(4)-C(5)-C(6)	0.30(18)
C(4)-C(5)-C(6)-C(1)	-0.81(17)
C(4)-C(5)-C(6)-C(7)	175.86(12)
C(5)-C(6)-C(7)-C(8)	-176.55(12)
C(6)-C(1)-C(2)-C(3)	0.24(17)
C(6)-C(7)-C(8)-N(1)	-0.71(13)
C(6)-C(7)-C(8)-C(9)	-177.88(11)
C(7)-C(8)-C(9)-O(1)	172.85(12)
C(7)-C(8)-C(9)-O(2)	-7.62(17)
C(8)-N(1)-C(1)-C(2)	176.21(11)
C(8)-N(1)-C(1)-C(6)	-0.48(12)
C(8)-N(1)-C(11)-C(12)	127.96(13)
C(8)-N(1)-C(11)-C(13)	-54.31(16)
C(10)-O(2)-C(9)-O(1)	1.18(16)
C(10)-O(2)-C(9)-C(8)	-178.35(9)
C(11)-N(1)-C(1)-C(2)	3.12(19)
C(11)-N(1)-C(1)-C(6)	-173.58(10)
C(11)-N(1)-C(8)-C(7)	173.56(11)
C(11)-N(1)-C(8)-C(9)	-8.96(17)
C(12)-C(11)-C(13)-O(3)	-15.03(19)
C(12)-C(11)-C(13)-O(4)	164.49(11)
C(13)-O(4)-C(14)-C(15)	90.87(14)
C(14)-O(4)-C(13)-O(3)	-7.79(18)
C(14)-O(4)-C(13)-C(11)	172.70(10)

Symmetry transformations used to generate equivalent atoms:

#1 x+1,y,z #2 -x,-y+1,-z

Table S13 Hydrogen bonds for O526 [Å and °].

D-H...A	d(D-H)	d(H...A)	d(D...A)	<(DHA)
C(7)-H(7)...O(3)#1	0.95	2.40	3.2934(15)	157.1
C(14)-H(14A)...O(1)#2	0.99	2.60	3.3807(15)	136.2

Symmetry transformations used to generate equivalent atoms:

#1 x+1,y,z #2 -x,-y+1,-z

E. Crystal Structure of 46

The Cambridge Crystallographic Data Centre (CCDC) has received the crystallographic data for compound **46** (CCDC 2417824).

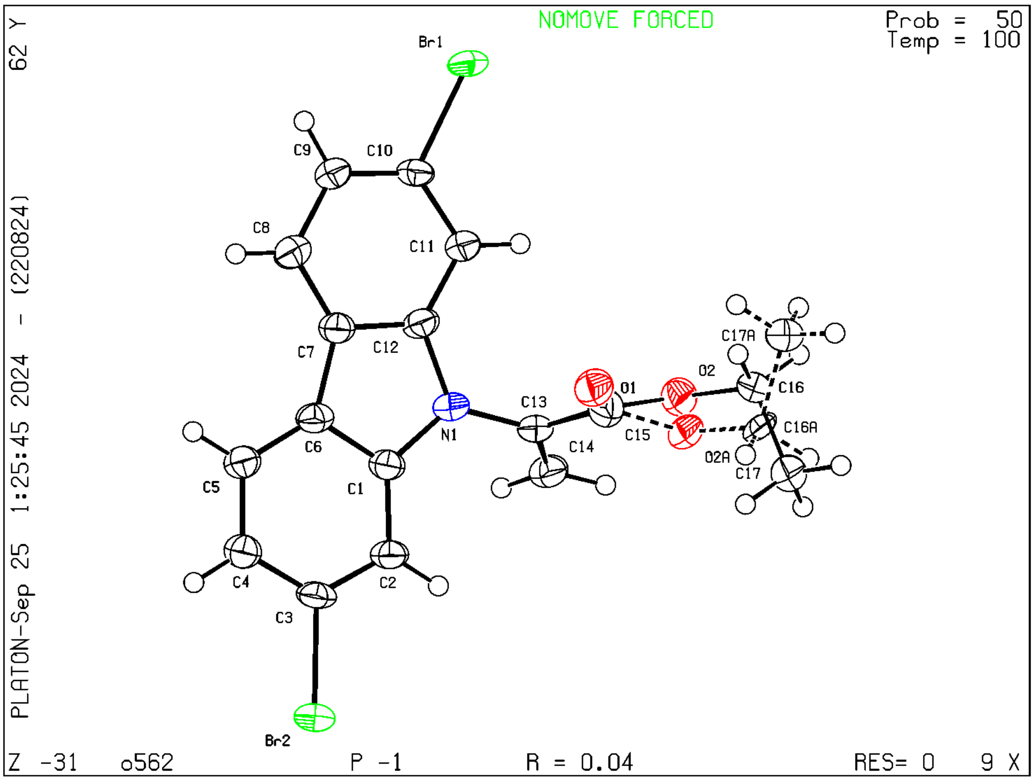

<u>Single Crystal Structure X-ray Analysis</u>

Sample Code:	O562
Sample ID:	Ku-0920
Student/Researcher:	Kulice Chan Wai Lun
Supervisor:	Lu Yixin
CDCC:	2417824
Date:	25-09-2024

Note: The sample crystallizes in a triclinic space group P-1. The asymmetric unit comprises a carbazole with molecular formula of $C_{17}H_{13}Br_2NO_2$. Part of the ester group is disordered into two positions with an occupancy ratio of 50:50. Constraint in thermal parameters have been applied to these disordered moieties. Final R values are R_1=0.0439 and wR_2=0.1319 for 2-theta up to 141.1°.

Table S14 Crystal data and structure refinement for O562.

Identification code	O562	
Empirical formula	C17 H13 Br2 N O2	
Formula weight	423.10	
Temperature	100.00 K	
Wavelength	1.54178 Å	
Crystal system	Triclinic	
Space group	P-1	
Unit cell dimensions	a = 6.8556(6) Å	a= 78.209(4)°.
	b = 10.1307(8) Å	b= 76.336(5)°.
	c = 11.8391(10) Å	g = 78.549(5)°.
Volume	772.30(11) $Å^3$	
Z	2	
Density (calculated)	1.819 Mg/m^3	
Absorption coefficient	6.712 mm^{-1}	
F(000)	416	
Crystal size	0.147 x 0.083 x 0.08 mm^3	
Theta range for data collection	3.896 to 70.560°.	
Index ranges	-8<=h<=8, -12<=k<=11, -14<=l<=14	
Reflections collected	28723	
Independent reflections	2937 [R(int) = 0.0620]	
Completeness to theta = 67.679°	99.9 %	
Absorption correction	Semi-empirical from equivalents	
Max. and min. transmission	0.7329 and 0.4363	
Refinement method	Full-matrix least-squares on F^2	
Data / restraints / parameters	2937 / 21 / 229	
Goodness-of-fit on F^2	1.170	
Final R indices [I>2sigma(I)]	R1 = 0.0439, wR2 = 0.1281	
R indices (all data)	R1 = 0.0485, wR2 = 0.1319	
Extinction coefficient	n/a	
Largest diff. peak and hole	0.612 and -0.996 e.$Å^{-3}$	

Table S15 Atomic coordinates (x 10^4) and equivalent isotropic displacement parameters ($Å^2$x 10^3) for O562. U(eq) is defined as one third of the trace of the orthogonalized U^{ij} tensor.

	x	y	z	U(eq)
Br(1)	4636(1)	9154(1)	1444(1)	40(1)
Br(2)	1639(1)	-288(1)	6462(1)	39(1)
O(1)	7607(5)	3472(3)	2144(3)	37(1)
N(1)	3473(5)	4012(3)	3169(3)	32(1)
C(1)	2814(6)	3330(4)	4312(4)	31(1)
C(2)	2595(6)	1959(4)	4679(4)	33(1)
C(3)	1936(6)	1571(4)	5872(4)	32(1)
C(4)	1487(6)	2464(4)	6695(4)	34(1)
C(5)	1725(6)	3818(4)	6303(4)	32(1)
C(6)	2414(6)	4263(4)	5108(4)	31(1)
C(7)	2864(6)	5556(4)	4416(4)	31(1)
C(8)	2755(6)	6843(4)	4701(4)	33(1)
C(9)	3290(6)	7905(4)	3795(4)	32(1)
C(10)	3911(6)	7665(4)	2647(4)	32(1)
C(11)	4076(6)	6403(4)	2319(4)	31(1)
C(12)	3527(6)	5362(4)	3235(4)	31(1)
C(13)	4142(6)	3404(4)	2132(4)	31(1)
C(14)	2902(7)	2876(4)	1714(4)	36(1)
C(15)	6376(7)	3291(5)	1633(4)	38(1)
O(2)	6704(13)	3264(11)	479(9)	36(2)
C(16)	8852(15)	2977(11)	-66(9)	36(2)
C(17)	9591(14)	1471(11)	83(8)	37(2)
O(2A)	6920(14)	2680(10)	635(9)	33(2)
C(16A)	9072(14)	2318(12)	134(10)	33(3)
C(17A)	9983(15)	3576(9)	-456(8)	38(3)

Table S16 Bond lengths [Å] and angles [°] for O562.

Br(1)-C(10)	1.907(4)
Br(2)-C(3)	1.904(4)
O(1)-C(15)	1.208(5)
N(1)-C(1)	1.402(5)
N(1)-C(12)	1.394(5)
N(1)-C(13)	1.425(5)
C(1)-C(2)	1.395(5)
C(1)-C(6)	1.412(6)
C(2)-H(2)	0.9500
C(2)-C(3)	1.377(6)
C(3)-C(4)	1.403(6)
C(4)-H(4)	0.9500
C(4)-C(5)	1.384(6)
C(5)-H(5)	0.9500
C(5)-C(6)	1.392(6)
C(6)-C(7)	1.444(5)
C(7)-C(8)	1.396(6)
C(7)-C(12)	1.406(6)
C(8)-H(8)	0.9500
C(8)-C(9)	1.393(6)
C(9)-H(9)	0.9500
C(9)-C(10)	1.382(6)
C(10)-C(11)	1.387(6)
C(11)-H(11)	0.9500
C(11)-C(12)	1.388(6)
C(13)-C(14)	1.320(6)
C(13)-C(15)	1.496(6)
C(14)-H(14A)	0.9500
C(14)-H(14B)	0.9500
C(15)-O(2)	1.336(11)
C(15)-O(2A)	1.385(11)
O(2)-C(16)	1.458(11)
C(16)-H(16A)	0.9900
C(16)-H(16B)	0.9900
C(16)-C(17)	1.498(13)

C(17)-H(17A)	0.9800
C(17)-H(17B)	0.9800
C(17)-H(17C)	0.9800
O(2A)-C(16A)	1.457(11)
C(16A)-H(16C)	0.9900
C(16A)-H(16D)	0.9900
C(16A)-C(17A)	1.499(12)
C(17A)-H(17D)	0.9800
C(17A)-H(17E)	0.9800
C(17A)-H(17F)	0.9800

C(1)-N(1)-C(13)	125.8(3)
C(12)-N(1)-C(1)	108.3(3)
C(12)-N(1)-C(13)	125.6(3)
N(1)-C(1)-C(6)	108.8(3)
C(2)-C(1)-N(1)	128.6(4)
C(2)-C(1)-C(6)	122.5(4)
C(1)-C(2)-H(2)	122.2
C(3)-C(2)-C(1)	115.6(4)
C(3)-C(2)-H(2)	122.2
C(2)-C(3)-Br(2)	118.7(3)
C(2)-C(3)-C(4)	124.0(4)
C(4)-C(3)-Br(2)	117.3(3)
C(3)-C(4)-H(4)	120.5
C(5)-C(4)-C(3)	119.0(4)
C(5)-C(4)-H(4)	120.5
C(4)-C(5)-H(5)	120.2
C(4)-C(5)-C(6)	119.6(4)
C(6)-C(5)-H(5)	120.2
C(1)-C(6)-C(7)	106.7(4)
C(5)-C(6)-C(1)	119.3(4)
C(5)-C(6)-C(7)	134.0(4)
C(8)-C(7)-C(6)	133.2(4)
C(8)-C(7)-C(12)	119.5(4)
C(12)-C(7)-C(6)	107.3(4)
C(7)-C(8)-H(8)	120.7
C(9)-C(8)-C(7)	118.5(4)

C(9)-C(8)-H(8)	120.7
C(8)-C(9)-H(9)	120.1
C(10)-C(9)-C(8)	119.7(4)
C(10)-C(9)-H(9)	120.1
C(9)-C(10)-Br(1)	117.9(3)
C(9)-C(10)-C(11)	124.1(4)
C(11)-C(10)-Br(1)	118.0(3)
C(10)-C(11)-H(11)	122.4
C(10)-C(11)-C(12)	115.2(4)
C(12)-C(11)-H(11)	122.4
N(1)-C(12)-C(7)	109.0(4)
C(11)-C(12)-N(1)	128.0(4)
C(11)-C(12)-C(7)	123.0(4)
N(1)-C(13)-C(15)	114.8(4)
C(14)-C(13)-N(1)	121.9(4)
C(14)-C(13)-C(15)	123.0(4)
C(13)-C(14)-H(14A)	120.0
C(13)-C(14)-H(14B)	120.0
H(14A)-C(14)-H(14B)	120.0
O(1)-C(15)-C(13)	124.2(4)
O(1)-C(15)-O(2)	125.3(5)
O(1)-C(15)-O(2A)	122.8(6)
O(2)-C(15)-C(13)	109.0(5)
O(2A)-C(15)-C(13)	111.7(5)
C(15)-O(2)-C(16)	113.4(9)
O(2)-C(16)-H(16A)	109.5
O(2)-C(16)-H(16B)	109.5
O(2)-C(16)-C(17)	110.8(8)
H(16A)-C(16)-H(16B)	108.1
C(17)-C(16)-H(16A)	109.5
C(17)-C(16)-H(16B)	109.5
C(16)-C(17)-H(17A)	109.5
C(16)-C(17)-H(17B)	109.5
C(16)-C(17)-H(17C)	109.5
H(17A)-C(17)-H(17B)	109.5
H(17A)-C(17)-H(17C)	109.5
H(17B)-C(17)-H(17C)	109.5

C(15)-O(2A)-C(16A)	118.7(9)
O(2A)-C(16A)-H(16C)	109.6
O(2A)-C(16A)-H(16D)	109.6
O(2A)-C(16A)-C(17A)	110.4(9)
H(16C)-C(16A)-H(16D)	108.1
C(17A)-C(16A)-H(16C)	109.6
C(17A)-C(16A)-H(16D)	109.6
C(16A)-C(17A)-H(17D)	109.5
C(16A)-C(17A)-H(17E)	109.5
C(16A)-C(17A)-H(17F)	109.5
H(17D)-C(17A)-H(17E)	109.5
H(17D)-C(17A)-H(17F)	109.5
H(17E)-C(17A)-H(17F)	109.5

Symmetry transformations used to generate equivalent atoms:

#1 -x+1,-y+1,-z

Table S17 Anisotropic displacement parameters ($Å^2$x 10^3) for O562. The anisotropic displacement factor exponent takes the form: $-2p^2[h^2 a^{*2}U^{11} + ... + 2 h k a^* b^* U^{12}]$

	U^{11}	U^{22}	U^{33}	U^{23}	U^{13}	U^{12}
Br(1)	60(1)	16(1)	39(1)	0(1)	-5(1)	-7(1)
Br(2)	50(1)	22(1)	41(1)	2(1)	-3(1)	-10(1)
O(1)	39(2)	36(2)	38(2)	-10(1)	-8(1)	-9(1)
N(1)	38(2)	21(2)	36(2)	-5(1)	-3(2)	-9(1)
C(1)	31(2)	24(2)	34(2)	-1(2)	-4(2)	-7(2)
C(2)	35(2)	22(2)	41(2)	-2(2)	-3(2)	-8(2)
C(3)	30(2)	21(2)	43(2)	2(2)	-4(2)	-10(2)
C(4)	35(2)	30(2)	35(2)	-3(2)	-6(2)	-8(2)
C(5)	34(2)	26(2)	37(2)	-5(2)	-6(2)	-4(2)
C(6)	31(2)	24(2)	38(2)	-4(2)	-7(2)	-8(2)
C(7)	30(2)	24(2)	36(2)	-3(2)	-4(2)	-6(2)
C(8)	34(2)	26(2)	39(2)	-7(2)	-8(2)	-3(2)
C(9)	35(2)	22(2)	37(2)	-6(2)	-5(2)	-2(2)
C(10)	37(2)	19(2)	35(2)	2(2)	-4(2)	-6(2)
C(11)	34(2)	22(2)	35(2)	-4(2)	-3(2)	-3(2)

C(12)	34(2)	20(2)	38(2)	-6(2)	-6(2)	-2(2)
C(13)	39(2)	18(2)	32(2)	0(2)	-4(2)	-6(2)
C(14)	39(2)	27(2)	44(2)	-9(2)	-4(2)	-9(2)
C(15)	42(2)	40(2)	33(2)	-9(2)	-3(2)	-12(2)
O(2)	33(4)	37(5)	38(4)	-9(4)	-2(3)	-8(4)
C(16)	41(5)	27(5)	35(5)	-5(5)	5(4)	-13(5)
C(17)	39(5)	35(5)	36(5)	-8(4)	-1(4)	-9(4)
O(2A)	38(4)	28(5)	35(4)	-12(4)	-6(3)	-6(4)
C(16A)	37(5)	18(6)	44(6)	-16(5)	1(4)	-4(5)
C(17A)	49(6)	28(5)	36(5)	-4(4)	-3(4)	-10(4)

Table S18 Hydrogen coordinates (x 10^4) and isotropic displacement parameters (Å^{2}x 10 ^{3}) for O562.

	x	y	z	U(eq)
H(2)	2882	1333	4141	40
H(4)	1026	2144	7509	41
H(5)	1419	4439	6846	39
H(8)	2325	6992	5495	39
H(9)	3229	8790	3967	38
H(11)	4533	6261	1524	38
H(14A)	1519	2896	2106	44
H(14B)	3391	2477	1020	44
H(16A)	9654	3420	299	43
H(16B)	9051	3364	-916	43
H(17A)	11016	1299	-324	55
H(17B)	8765	1029	-254	55
H(17C)	9477	1098	923	55
H(16C)	9236	1707	-448	39
H(16D)	9793	1821	766	39
H(17D)	9268	4066	-1082	57
H(17E)	11424	3315	-797	57
H(17F)	9852	4168	126	57

Table S19 Torsion angles [°] for O562.

Br(1)-C(10)-C(11)-C(12)	180.0(3)
Br(2)-C(3)-C(4)-C(5)	178.7(3)
O(1)-C(15)-O(2)-C(16)	21.1(12)
O(1)-C(15)-O(2A)-C(16A)	-4.0(12)
N(1)-C(1)-C(2)-C(3)	179.2(4)
N(1)-C(1)-C(6)-C(5)	179.7(4)
N(1)-C(1)-C(6)-C(7)	-0.4(5)
N(1)-C(13)-C(15)-O(1)	12.2(6)
N(1)-C(13)-C(15)-O(2)	-154.4(6)
N(1)-C(13)-C(15)-O(2A)	179.4(6)
C(1)-N(1)-C(12)-C(7)	-1.1(4)
C(1)-N(1)-C(12)-C(11)	-179.6(4)
C(1)-N(1)-C(13)-C(14)	65.7(6)
C(1)-N(1)-C(13)-C(15)	-108.6(5)
C(1)-C(2)-C(3)-Br(2)	-178.9(3)
C(1)-C(2)-C(3)-C(4)	0.3(6)
C(1)-C(6)-C(7)-C(8)	179.5(4)
C(1)-C(6)-C(7)-C(12)	-0.3(5)
C(2)-C(1)-C(6)-C(5)	-1.7(6)
C(2)-C(1)-C(6)-C(7)	178.3(4)
C(2)-C(3)-C(4)-C(5)	-0.5(7)
C(3)-C(4)-C(5)-C(6)	-0.4(6)
C(4)-C(5)-C(6)-C(1)	1.4(6)
C(4)-C(5)-C(6)-C(7)	-178.5(4)
C(5)-C(6)-C(7)-C(8)	-0.6(8)
C(5)-C(6)-C(7)-C(12)	179.6(5)
C(6)-C(1)-C(2)-C(3)	0.8(6)
C(6)-C(7)-C(8)-C(9)	-179.1(4)
C(6)-C(7)-C(12)-N(1)	0.9(5)
C(6)-C(7)-C(12)-C(11)	179.4(4)
C(7)-C(8)-C(9)-C(10)	0.0(6)
C(8)-C(7)-C(12)-N(1)	-179.0(4)
C(8)-C(7)-C(12)-C(11)	-0.4(6)
C(8)-C(9)-C(10)-Br(1)	-179.8(3)
C(8)-C(9)-C(10)-C(11)	-1.0(7)

C(9)-C(10)-C(11)-C(12)	1.3(6)
C(10)-C(11)-C(12)-N(1)	177.7(4)
C(10)-C(11)-C(12)-C(7)	-0.5(6)
C(12)-N(1)-C(1)-C(2)	-177.6(4)
C(12)-N(1)-C(1)-C(6)	1.0(4)
C(12)-N(1)-C(13)-C(14)	-120.1(5)
C(12)-N(1)-C(13)-C(15)	65.6(5)
C(12)-C(7)-C(8)-C(9)	0.7(6)
C(13)-N(1)-C(1)-C(2)	-2.6(7)
C(13)-N(1)-C(1)-C(6)	176.0(4)
C(13)-N(1)-C(12)-C(7)	-176.2(4)
C(13)-N(1)-C(12)-C(11)	5.4(7)
C(13)-C(15)-O(2)-C(16)	-172.5(7)
C(13)-C(15)-O(2A)-C(16A)	-171.5(8)
C(14)-C(13)-C(15)-O(1)	-162.1(4)
C(14)-C(13)-C(15)-O(2)	31.3(7)
C(14)-C(13)-C(15)-O(2A)	5.2(7)
C(15)-O(2)-C(16)-C(17)	84.4(11)
C(15)-O(2A)-C(16A)-C(17A)	-72.4(12)

Symmetry transformations used to generate equivalent atoms:

#1 -x+1,-y+1,-z #2 x-1,y,z

Table S20 Hydrogen bonds for O562 [Å and °].

D-H...A	d(D-H)	d(H...A)	d(D...A)	<(DHA)
C(11)-H(11)...O(2)#1	0.95	2.63	3.418(12)	141.1
C(14)-H(14A)...O(1)#2	0.95	2.62	3.490(5)	152.3

Symmetry transformations used to generate equivalent atoms:

#1 -x+1,-y+1,-z #2 x-1,y,z

F. Crystal Structure of 54

The Cambridge Crystallographic Data Centre (CCDC) has received the crystallographic data for compound **54** (CCDC 2419837).

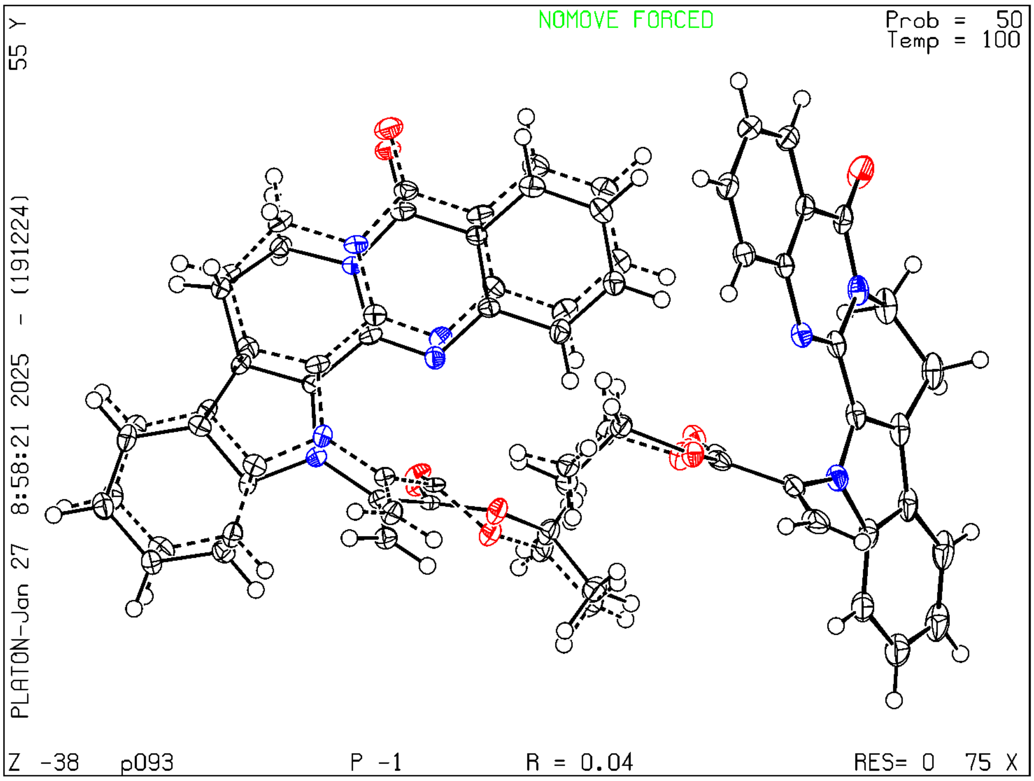

<u>Single Crystal Structure X-ray Analysis</u>

Sample Code:	P093
Sample ID:	Ku-54
Student/Researcher:	Kulice Chan Wai Lun
Supervisor:	Lu Yixin
Date:	27-01-2025

Note: The sample crystallizes in a triclinic space group P-1. The asymmetric unit comprises two fused pentacyclic compounds, each molecule having a molecular formula of $C_{23}H_{19}N_3O_3$. One of the whole petacycle is disordered into two positions with an occupancy ratio of 50:50, whereas the ethyl carboxylate moiety of the other pentacycle is disordered into two positions with an occupancy ratio of 50:50. Restraints and constraints in thermal parameters and bond lengths have been applied to these disordered molecules. Final R values are R_1=0.0409 and wR_2=0.1121 for 2-theta up to 59.2°.

Table S21 Crystal data and structure refinement for P093.

Identification code	p093	
Empirical formula	C23 H19 N3 O3	
Formula weight	385.41	
Temperature	100.00 K	
Wavelength	0.71073 Å	
Crystal system	Triclinic	
Space group	P-1	
Unit cell dimensions	a = 8.6669(5) Å	a= 82.643(2)°.
	b = 11.5774(6) Å	b= 80.073(2)°.
	c = 19.0390(11) Å	g = 75.024(2)°.
Volume	1810.85(18) $Å^3$	
Z	4	
Density (calculated)	1.414 Mg/m^3	
Absorption coefficient	0.096 mm^{-1}	
F(000)	808	
Crystal size	0.251 x 0.172 x 0.144 mm^3	
Theta range for data collection	2.459 to 29.594°.	
Index ranges	-12<=h<=12, -16<=k<=16, -26<=l<=26	
Reflections collected	110882	
Independent reflections	10158 [R(int) = 0.0604]	
Completeness to theta = 25.242°	99.8 %	
Absorption correction	Semi-empirical from equivalents	
Max. and min. transmission	0.7458 and 0.7136	
Refinement method	Full-matrix least-squares on F^2	
Data / restraints / parameters	10158 / 1208 / 835	
Goodness-of-fit on F^2	1.024	
Final R indices [I>2sigma(I)]	R1 = 0.0409, wR2 = 0.1014	
R indices (all data)	R1 = 0.0530, wR2 = 0.1121	
Extinction coefficient	n/a	
Largest diff. peak and hole	0.343 and -0.256 e.$Å^{-3}$	

Table S22 Atomic coordinates (x 10^4) and equivalent isotropic displacement parameters (Å^{2}x 10^3) for P093. U(eq) is defined as one third of the trace of the orthogonalized U^{ij} tensor.

	x	y	z	U(eq)
O(1)	7438(1)	12463(1)	6529(1)	33(1)
N(1)	9268(1)	8940(1)	6154(1)	18(1)
N(2)	7756(1)	10920(1)	5842(1)	21(1)
N(3)	8410(1)	8166(1)	4906(1)	22(1)
C(1)	9599(1)	9352(1)	6751(1)	18(1)
C(2)	10498(1)	8526(1)	7226(1)	20(1)
C(3)	10872(1)	8913(1)	7816(1)	23(1)
C(4)	10356(2)	10125(1)	7951(1)	28(1)
C(5)	9439(2)	10941(1)	7499(1)	27(1)
C(6)	9055(1)	10560(1)	6897(1)	21(1)
C(7)	8036(1)	11405(1)	6427(1)	23(1)
C(8)	6510(1)	11690(1)	5421(1)	27(1)
C(9)	6793(1)	11435(1)	4643(1)	28(1)
C(10)	7238(1)	10111(1)	4591(1)	25(1)
C(11)	7993(1)	9338(1)	5105(1)	21(1)
C(12)	8382(1)	9710(1)	5738(1)	19(1)
C(13)	7122(1)	9410(1)	4051(1)	27(1)
C(14)	6416(2)	9687(1)	3419(1)	35(1)
C(15)	6439(2)	8765(2)	3027(1)	42(1)
C(16)	7159(2)	7573(2)	3243(1)	40(1)
C(17)	7885(2)	7268(1)	3858(1)	31(1)
C(18)	7855(1)	8201(1)	4257(1)	25(1)
C(19)	9226(2)	7106(1)	5281(1)	24(1)
C(20)	10543(2)	6384(1)	4972(1)	29(1)
O(2)	7158(5)	7100(3)	6214(3)	22(1)
C(21)	8563(7)	6722(6)	6019(3)	19(1)
O(3)	9657(3)	5921(3)	6360(2)	18(1)
C(22)	9055(4)	5567(2)	7094(1)	20(1)
C(23)	8089(3)	4645(2)	7138(1)	23(1)
O(2A)	6834(6)	7447(3)	6242(3)	23(1)
C(21A)	8207(7)	6922(7)	6018(3)	22(1)
O(3A)	9081(3)	6015(3)	6407(2)	21(1)

C(22A)	8326(4)	5633(2)	7127(1)	21(1)
C(23A)	9161(3)	4354(2)	7308(1)	21(1)
C(24)	4738(6)	7185(3)	8629(2)	15(1)
C(25)	5559(5)	7482(3)	7941(2)	19(1)
C(26)	6183(4)	8469(3)	7829(2)	20(1)
C(27)	6059(4)	9191(3)	8379(2)	22(1)
C(28)	5231(5)	8933(3)	9050(2)	20(1)
C(29)	4563(6)	7935(4)	9167(2)	16(1)
C(30)	3615(7)	7674(5)	9863(3)	17(1)
O(4)	3268(7)	8342(4)	10351(2)	23(1)
N(5)	3041(5)	6640(4)	9918(2)	15(1)
C(31)	1813(3)	6466(2)	10546(1)	18(1)
C(32)	1908(4)	5151(3)	10803(2)	17(1)
C(33)	2133(10)	4435(5)	10182(3)	15(1)
C(34)	2819(6)	4829(4)	9510(2)	14(1)
C(35)	3383(7)	5916(5)	9357(3)	15(1)
N(4)	4192(7)	6142(4)	8734(3)	17(1)
C(36)	1897(8)	3282(4)	10129(3)	17(1)
C(37)	1252(4)	2466(3)	10623(2)	20(1)
C(38)	1187(4)	1394(3)	10398(2)	22(1)
C(39)	1748(7)	1132(3)	9683(3)	19(1)
C(40)	2387(10)	1921(6)	9188(4)	19(1)
C(41)	2470(8)	2994(5)	9412(3)	15(1)
N(6)	3023(4)	3957(3)	9036(2)	16(1)
C(42)	3782(4)	3948(3)	8308(2)	16(1)
C(43)	4986(3)	3034(2)	8093(2)	19(1)
O(5)	1742(9)	5600(10)	7909(5)	21(1)
C(44)	3041(5)	4939(4)	7799(2)	15(1)
O(6)	4417(3)	5098(3)	7280(1)	20(1)
C(45)	3789(5)	5842(4)	6670(2)	21(1)
C(46)	4791(6)	5323(6)	6002(3)	26(1)
C(24A)	4657(6)	7532(4)	8709(3)	17(1)
C(25A)	5372(5)	7872(3)	8016(2)	21(1)
C(26A)	5958(4)	8892(3)	7906(2)	21(1)
C(27A)	5808(5)	9607(3)	8462(2)	23(1)
C(28A)	5043(6)	9306(3)	9132(2)	22(1)
C(29A)	4465(6)	8269(4)	9258(3)	19(1)

C(30A)	3686(8)	7921(5)	9974(3)	18(1)
O(4A)	3474(7)	8502(5)	10490(3)	26(1)
N(5A)	3251(5)	6832(4)	10033(2)	17(1)
C(31A)	2690(3)	6347(2)	10772(1)	20(1)
C(32A)	1525(4)	5568(3)	10796(2)	20(1)
C(33A)	2109(10)	4725(5)	10222(4)	17(1)
C(34A)	3038(6)	5031(5)	9601(3)	16(1)
C(35A)	3523(7)	6146(5)	9455(3)	16(1)
N(4A)	4176(7)	6454(4)	8807(3)	17(1)
C(36A)	1795(8)	3601(4)	10155(3)	16(1)
C(37A)	858(4)	2875(3)	10590(2)	21(1)
C(38A)	769(4)	1835(3)	10347(2)	24(1)
C(39A)	1585(8)	1491(4)	9668(3)	25(1)
C(40A)	2506(10)	2182(6)	9225(4)	20(1)
C(41A)	2588(9)	3241(6)	9473(4)	17(1)
N(6A)	3354(4)	4128(3)	9137(2)	17(1)
C(42A)	4184(4)	4099(3)	8421(2)	16(1)
C(43A)	5427(4)	3199(2)	8230(2)	19(1)
O(5A)	1978(9)	5609(11)	7998(5)	21(1)
C(44A)	3391(5)	5045(4)	7894(2)	16(1)
O(6A)	3996(3)	4948(3)	7169(1)	20(1)
C(45A)	3357(5)	5766(4)	6590(2)	22(1)
C(46A)	4391(6)	5309(6)	5907(3)	24(1)

Table S23 Bond lengths [Å] and angles [°] for P093.

O(1)-C(7)	1.2264(13)
N(1)-C(1)	1.3855(13)
N(1)-C(12)	1.2988(13)
N(2)-C(7)	1.3926(15)
N(2)-C(8)	1.4898(13)
N(2)-C(12)	1.3926(13)
N(3)-C(11)	1.3953(14)
N(3)-C(18)	1.3933(13)
N(3)-C(19)	1.4203(14)
C(1)-C(2)	1.4065(14)

C(1)-C(6)	1.4031(14)
C(2)-H(2)	0.9500
C(2)-C(3)	1.3775(15)
C(3)-H(3)	0.9500
C(3)-C(4)	1.4005(16)
C(4)-H(4)	0.9500
C(4)-C(5)	1.3801(17)
C(5)-H(5)	0.9500
C(5)-C(6)	1.3989(16)
C(6)-C(7)	1.4623(15)
C(8)-H(8A)	0.9900
C(8)-H(8B)	0.9900
C(8)-C(9)	1.5143(18)
C(9)-H(9A)	0.9900
C(9)-H(9B)	0.9900
C(9)-C(10)	1.4921(17)
C(10)-C(11)	1.3714(14)
C(10)-C(13)	1.4209(18)
C(11)-C(12)	1.4482(15)
C(13)-C(14)	1.4075(16)
C(13)-C(18)	1.4151(17)
C(14)-H(14)	0.9500
C(14)-C(15)	1.372(2)
C(15)-H(15)	0.9500
C(15)-C(16)	1.401(2)
C(16)-H(16)	0.9500
C(16)-C(17)	1.3874(17)
C(17)-H(17)	0.9500
C(17)-C(18)	1.3915(18)
C(19)-C(20)	1.3231(18)
C(19)-C(21)	1.481(4)
C(19)-C(21A)	1.545(5)
C(20)-H(20A)	0.9500
C(20)-H(20B)	0.9500
O(2)-C(21)	1.193(4)
C(21)-O(3)	1.336(4)
O(3)-C(22)	1.451(4)

C(22)-H(22A)	0.9900
C(22)-H(22B)	0.9900
C(22)-C(23)	1.504(3)
C(23)-H(23A)	0.9800
C(23)-H(23B)	0.9800
C(23)-H(23C)	0.9800
O(2A)-C(21A)	1.218(5)
C(21A)-O(3A)	1.345(4)
O(3A)-C(22A)	1.478(4)
C(22A)-H(22C)	0.9900
C(22A)-H(22D)	0.9900
C(22A)-C(23A)	1.496(3)
C(23A)-H(23D)	0.9800
C(23A)-H(23E)	0.9800
C(23A)-H(23F)	0.9800
C(24)-C(25)	1.425(5)
C(24)-C(29)	1.390(4)
C(24)-N(4)	1.387(5)
C(25)-H(25)	0.9500
C(25)-C(26)	1.364(4)
C(26)-H(26)	0.9500
C(26)-C(27)	1.396(4)
C(27)-H(27)	0.9500
C(27)-C(28)	1.390(5)
C(28)-H(28)	0.9500
C(28)-C(29)	1.399(5)
C(29)-C(30)	1.474(6)
C(30)-O(4)	1.233(4)
C(30)-N(5)	1.397(5)
N(5)-C(31)	1.487(5)
N(5)-C(35)	1.387(5)
C(31)-H(31A)	0.9900
C(31)-H(31B)	0.9900
C(31)-C(32)	1.524(4)
C(32)-H(32A)	0.9900
C(32)-H(32B)	0.9900
C(32)-C(33)	1.486(6)

C(33)-C(34)	1.387(6)
C(33)-C(36)	1.420(6)
C(34)-C(35)	1.443(5)
C(34)-N(6)	1.397(4)
C(35)-N(4)	1.302(6)
C(36)-C(37)	1.398(6)
C(36)-C(41)	1.420(7)
C(37)-H(37)	0.9500
C(37)-C(38)	1.381(4)
C(38)-H(38)	0.9500
C(38)-C(39)	1.408(6)
C(39)-H(39)	0.9500
C(39)-C(40)	1.375(7)
C(40)-H(40)	0.9500
C(40)-C(41)	1.388(6)
C(41)-N(6)	1.392(5)
N(6)-C(42)	1.429(4)
C(42)-C(43)	1.330(4)
C(42)-C(44)	1.489(5)
C(43)-H(43A)	0.9500
C(43)-H(43B)	0.9500
O(5)-C(44)	1.188(8)
C(44)-O(6)	1.447(5)
O(6)-C(45)	1.451(5)
C(45)-H(45A)	0.9900
C(45)-H(45B)	0.9900
C(45)-C(46)	1.510(7)
C(46)-H(46A)	0.9800
C(46)-H(46B)	0.9800
C(46)-H(46C)	0.9800
C(24A)-C(25A)	1.413(6)
C(24A)-C(29A)	1.395(5)
C(24A)-N(4A)	1.396(5)
C(25A)-H(25A)	0.9500
C(25A)-C(26A)	1.380(5)
C(26A)-H(26A)	0.9500
C(26A)-C(27A)	1.393(4)

C(27A)-H(27A)	0.9500
C(27A)-C(28A)	1.380(5)
C(28A)-H(28A)	0.9500
C(28A)-C(29A)	1.394(5)
C(29A)-C(30A)	1.470(7)
C(30A)-O(4A)	1.222(4)
C(30A)-N(5A)	1.391(5)
N(5A)-C(31A)	1.495(5)
N(5A)-C(35A)	1.390(5)
C(31A)-H(31C)	0.9900
C(31A)-H(31D)	0.9900
C(31A)-C(32A)	1.510(4)
C(32A)-H(32C)	0.9900
C(32A)-H(32D)	0.9900
C(32A)-C(33A)	1.493(6)
C(33A)-C(34A)	1.373(7)
C(33A)-C(36A)	1.422(6)
C(34A)-C(35A)	1.439(5)
C(34A)-N(6A)	1.398(4)
C(35A)-N(4A)	1.312(6)
C(36A)-C(37A)	1.413(6)
C(36A)-C(41A)	1.418(7)
C(37A)-H(37A)	0.9500
C(37A)-C(38A)	1.369(4)
C(38A)-H(38A)	0.9500
C(38A)-C(39A)	1.414(6)
C(39A)-H(39A)	0.9500
C(39A)-C(40A)	1.383(7)
C(40A)-H(40A)	0.9500
C(40A)-C(41A)	1.392(6)
C(41A)-N(6A)	1.394(6)
N(6A)-C(42A)	1.427(4)
C(42A)-C(43A)	1.327(4)
C(42A)-C(44A)	1.499(5)
C(43A)-H(43C)	0.9500
C(43A)-H(43D)	0.9500
O(5A)-C(44A)	1.226(8)

C(44A)-O(6A)	1.397(5)
O(6A)-C(45A)	1.447(5)
C(45A)-H(45C)	0.9900
C(45A)-H(45D)	0.9900
C(45A)-C(46A)	1.516(7)
C(46A)-H(46D)	0.9800
C(46A)-H(46E)	0.9800
C(46A)-H(46F)	0.9800

C(12)-N(1)-C(1)	117.53(9)
C(7)-N(2)-C(8)	116.52(9)
C(12)-N(2)-C(7)	121.15(9)
C(12)-N(2)-C(8)	121.30(9)
C(11)-N(3)-C(19)	128.05(9)
C(18)-N(3)-C(11)	107.31(9)
C(18)-N(3)-C(19)	124.64(10)
N(1)-C(1)-C(2)	118.59(9)
N(1)-C(1)-C(6)	122.19(9)
C(6)-C(1)-C(2)	119.22(10)
C(1)-C(2)-H(2)	120.1
C(3)-C(2)-C(1)	119.88(10)
C(3)-C(2)-H(2)	120.1
C(2)-C(3)-H(3)	119.7
C(2)-C(3)-C(4)	120.66(10)
C(4)-C(3)-H(3)	119.7
C(3)-C(4)-H(4)	119.9
C(5)-C(4)-C(3)	120.11(10)
C(5)-C(4)-H(4)	119.9
C(4)-C(5)-H(5)	120.1
C(4)-C(5)-C(6)	119.80(10)
C(6)-C(5)-H(5)	120.1
C(1)-C(6)-C(7)	119.22(10)
C(5)-C(6)-C(1)	120.29(10)
C(5)-C(6)-C(7)	120.45(10)
O(1)-C(7)-N(2)	120.86(10)
O(1)-C(7)-C(6)	124.15(11)
N(2)-C(7)-C(6)	114.98(9)

N(2)-C(8)-H(8A)	108.9
N(2)-C(8)-H(8B)	108.9
N(2)-C(8)-C(9)	113.54(9)
H(8A)-C(8)-H(8B)	107.7
C(9)-C(8)-H(8A)	108.9
C(9)-C(8)-H(8B)	108.9
C(8)-C(9)-H(9A)	109.7
C(8)-C(9)-H(9B)	109.7
H(9A)-C(9)-H(9B)	108.2
C(10)-C(9)-C(8)	109.62(9)
C(10)-C(9)-H(9A)	109.7
C(10)-C(9)-H(9B)	109.7
C(11)-C(10)-C(9)	120.95(11)
C(11)-C(10)-C(13)	107.23(10)
C(13)-C(10)-C(9)	131.70(10)
N(3)-C(11)-C(12)	125.51(9)
C(10)-C(11)-N(3)	110.14(10)
C(10)-C(11)-C(12)	124.25(10)
N(1)-C(12)-N(2)	124.85(10)
N(1)-C(12)-C(11)	120.33(9)
N(2)-C(12)-C(11)	114.81(9)
C(14)-C(13)-C(10)	133.37(12)
C(14)-C(13)-C(18)	119.38(12)
C(18)-C(13)-C(10)	107.21(10)
C(13)-C(14)-H(14)	120.7
C(15)-C(14)-C(13)	118.58(13)
C(15)-C(14)-H(14)	120.7
C(14)-C(15)-H(15)	119.4
C(14)-C(15)-C(16)	121.16(12)
C(16)-C(15)-H(15)	119.4
C(15)-C(16)-H(16)	119.0
C(17)-C(16)-C(15)	121.90(14)
C(17)-C(16)-H(16)	119.0
C(16)-C(17)-H(17)	121.5
C(16)-C(17)-C(18)	116.93(13)
C(18)-C(17)-H(17)	121.5
N(3)-C(18)-C(13)	108.09(10)

C(17)-C(18)-N(3)	129.81(11)
C(17)-C(18)-C(13)	122.04(11)
N(3)-C(19)-C(21)	121.0(2)
N(3)-C(19)-C(21A)	108.6(2)
C(20)-C(19)-N(3)	121.51(10)
C(20)-C(19)-C(21)	117.4(2)
C(20)-C(19)-C(21A)	129.4(2)
C(19)-C(20)-H(20A)	120.0
C(19)-C(20)-H(20B)	120.0
H(20A)-C(20)-H(20B)	120.0
O(2)-C(21)-C(19)	117.9(4)
O(2)-C(21)-O(3)	129.1(4)
O(3)-C(21)-C(19)	112.9(4)
C(21)-O(3)-C(22)	114.0(3)
O(3)-C(22)-H(22A)	109.2
O(3)-C(22)-H(22B)	109.2
O(3)-C(22)-C(23)	112.0(2)
H(22A)-C(22)-H(22B)	107.9
C(23)-C(22)-H(22A)	109.2
C(23)-C(22)-H(22B)	109.2
C(22)-C(23)-H(23A)	109.5
C(22)-C(23)-H(23B)	109.5
C(22)-C(23)-H(23C)	109.5
H(23A)-C(23)-H(23B)	109.5
H(23A)-C(23)-H(23C)	109.5
H(23B)-C(23)-H(23C)	109.5
O(2A)-C(21A)-C(19)	129.3(4)
O(2A)-C(21A)-O(3A)	122.4(4)
O(3A)-C(21A)-C(19)	108.3(3)
C(21A)-O(3A)-C(22A)	118.3(3)
O(3A)-C(22A)-H(22C)	110.2
O(3A)-C(22A)-H(22D)	110.2
O(3A)-C(22A)-C(23A)	107.8(2)
H(22C)-C(22A)-H(22D)	108.5
C(23A)-C(22A)-H(22C)	110.2
C(23A)-C(22A)-H(22D)	110.2
C(22A)-C(23A)-H(23D)	109.5

C(22A)-C(23A)-H(23E)	109.5
C(22A)-C(23A)-H(23F)	109.5
H(23D)-C(23A)-H(23E)	109.5
H(23D)-C(23A)-H(23F)	109.5
H(23E)-C(23A)-H(23F)	109.5
C(29)-C(24)-C(25)	118.5(4)
N(4)-C(24)-C(25)	118.3(4)
N(4)-C(24)-C(29)	123.2(4)
C(24)-C(25)-H(25)	120.1
C(26)-C(25)-C(24)	119.8(3)
C(26)-C(25)-H(25)	120.1
C(25)-C(26)-H(26)	119.3
C(25)-C(26)-C(27)	121.4(3)
C(27)-C(26)-H(26)	119.3
C(26)-C(27)-H(27)	120.1
C(28)-C(27)-C(26)	119.8(3)
C(28)-C(27)-H(27)	120.1
C(27)-C(28)-H(28)	120.4
C(27)-C(28)-C(29)	119.2(3)
C(29)-C(28)-H(28)	120.4
C(24)-C(29)-C(28)	121.2(4)
C(24)-C(29)-C(30)	118.4(4)
C(28)-C(29)-C(30)	120.3(3)
O(4)-C(30)-C(29)	123.4(4)
O(4)-C(30)-N(5)	121.6(5)
N(5)-C(30)-C(29)	114.9(4)
C(30)-N(5)-C(31)	117.0(3)
C(35)-N(5)-C(30)	121.4(4)
C(35)-N(5)-C(31)	120.7(3)
N(5)-C(31)-H(31A)	108.9
N(5)-C(31)-H(31B)	108.9
N(5)-C(31)-C(32)	113.4(2)
H(31A)-C(31)-H(31B)	107.7
C(32)-C(31)-H(31A)	108.9
C(32)-C(31)-H(31B)	108.9
C(31)-C(32)-H(32A)	109.6
C(31)-C(32)-H(32B)	109.6

H(32A)-C(32)-H(32B)	108.1
C(33)-C(32)-C(31)	110.2(3)
C(33)-C(32)-H(32A)	109.6
C(33)-C(32)-H(32B)	109.6
C(34)-C(33)-C(32)	120.6(4)
C(34)-C(33)-C(36)	107.2(4)
C(36)-C(33)-C(32)	132.0(5)
C(33)-C(34)-C(35)	124.3(4)
C(33)-C(34)-N(6)	109.4(4)
N(6)-C(34)-C(35)	126.0(4)
N(5)-C(35)-C(34)	115.2(4)
N(4)-C(35)-N(5)	124.6(4)
N(4)-C(35)-C(34)	120.2(4)
C(35)-N(4)-C(24)	117.2(4)
C(37)-C(36)-C(33)	132.9(5)
C(37)-C(36)-C(41)	119.7(4)
C(41)-C(36)-C(33)	107.4(4)
C(36)-C(37)-H(37)	120.6
C(38)-C(37)-C(36)	118.7(3)
C(38)-C(37)-H(37)	120.6
C(37)-C(38)-H(38)	119.7
C(37)-C(38)-C(39)	120.6(3)
C(39)-C(38)-H(38)	119.7
C(38)-C(39)-H(39)	119.2
C(40)-C(39)-C(38)	121.7(4)
C(40)-C(39)-H(39)	119.2
C(39)-C(40)-H(40)	121.0
C(39)-C(40)-C(41)	118.0(6)
C(41)-C(40)-H(40)	121.0
C(40)-C(41)-C(36)	121.3(5)
C(40)-C(41)-N(6)	130.8(5)
N(6)-C(41)-C(36)	107.9(4)
C(34)-N(6)-C(42)	128.5(3)
C(41)-N(6)-C(34)	108.0(3)
C(41)-N(6)-C(42)	123.3(3)
N(6)-C(42)-C(44)	116.4(3)
C(43)-C(42)-N(6)	120.9(3)

C(43)-C(42)-C(44)	122.4(3)
C(42)-C(43)-H(43A)	120.0
C(42)-C(43)-H(43B)	120.0
H(43A)-C(43)-H(43B)	120.0
O(5)-C(44)-C(42)	126.0(6)
O(5)-C(44)-O(6)	129.1(7)
O(6)-C(44)-C(42)	102.5(3)
C(44)-O(6)-C(45)	107.0(3)
O(6)-C(45)-H(45A)	110.2
O(6)-C(45)-H(45B)	110.2
O(6)-C(45)-C(46)	107.5(4)
H(45A)-C(45)-H(45B)	108.5
C(46)-C(45)-H(45A)	110.2
C(46)-C(45)-H(45B)	110.2
C(45)-C(46)-H(46A)	109.5
C(45)-C(46)-H(46B)	109.5
C(45)-C(46)-H(46C)	109.5
H(46A)-C(46)-H(46B)	109.5
H(46A)-C(46)-H(46C)	109.5
H(46B)-C(46)-H(46C)	109.5
C(29A)-C(24A)-C(25A)	119.5(4)
C(29A)-C(24A)-N(4A)	123.3(4)
N(4A)-C(24A)-C(25A)	117.2(4)
C(24A)-C(25A)-H(25A)	120.5
C(26A)-C(25A)-C(24A)	119.1(3)
C(26A)-C(25A)-H(25A)	120.5
C(25A)-C(26A)-H(26A)	119.4
C(25A)-C(26A)-C(27A)	121.2(3)
C(27A)-C(26A)-H(26A)	119.4
C(26A)-C(27A)-H(27A)	120.1
C(28A)-C(27A)-C(26A)	119.8(3)
C(28A)-C(27A)-H(27A)	120.1
C(27A)-C(28A)-H(28A)	120.0
C(27A)-C(28A)-C(29A)	120.1(3)
C(29A)-C(28A)-H(28A)	120.0
C(24A)-C(29A)-C(30A)	119.0(4)
C(28A)-C(29A)-C(24A)	120.3(4)

C(28A)-C(29A)-C(30A)	120.7(4)
O(4A)-C(30A)-C(29A)	123.9(5)
O(4A)-C(30A)-N(5A)	121.6(5)
N(5A)-C(30A)-C(29A)	114.5(4)
C(30A)-N(5A)-C(31A)	116.4(3)
C(35A)-N(5A)-C(30A)	122.3(4)
C(35A)-N(5A)-C(31A)	120.8(4)
N(5A)-C(31A)-H(31C)	108.8
N(5A)-C(31A)-H(31D)	108.8
N(5A)-C(31A)-C(32A)	113.7(2)
H(31C)-C(31A)-H(31D)	107.7
C(32A)-C(31A)-H(31C)	108.8
C(32A)-C(31A)-H(31D)	108.8
C(31A)-C(32A)-H(32C)	109.7
C(31A)-C(32A)-H(32D)	109.7
H(32C)-C(32A)-H(32D)	108.2
C(33A)-C(32A)-C(31A)	110.0(4)
C(33A)-C(32A)-H(32C)	109.7
C(33A)-C(32A)-H(32D)	109.7
C(34A)-C(33A)-C(32A)	120.6(4)
C(34A)-C(33A)-C(36A)	107.7(4)
C(36A)-C(33A)-C(32A)	131.6(5)
C(33A)-C(34A)-C(35A)	123.8(4)
C(33A)-C(34A)-N(6A)	109.6(4)
N(6A)-C(34A)-C(35A)	126.6(4)
N(5A)-C(35A)-C(34A)	115.9(4)
N(4A)-C(35A)-N(5A)	124.4(4)
N(4A)-C(35A)-C(34A)	119.6(4)
C(35A)-N(4A)-C(24A)	116.5(4)
C(37A)-C(36A)-C(33A)	134.0(5)
C(37A)-C(36A)-C(41A)	118.9(4)
C(41A)-C(36A)-C(33A)	107.0(4)
C(36A)-C(37A)-H(37A)	120.5
C(38A)-C(37A)-C(36A)	119.0(3)
C(38A)-C(37A)-H(37A)	120.5
C(37A)-C(38A)-H(38A)	119.6
C(37A)-C(38A)-C(39A)	120.9(3)

C(39A)-C(38A)-H(38A)	119.6
C(38A)-C(39A)-H(39A)	119.1
C(40A)-C(39A)-C(38A)	121.8(5)
C(40A)-C(39A)-H(39A)	119.1
C(39A)-C(40A)-H(40A)	121.5
C(39A)-C(40A)-C(41A)	117.1(5)
C(41A)-C(40A)-H(40A)	121.5
C(40A)-C(41A)-C(36A)	122.3(5)
C(40A)-C(41A)-N(6A)	129.7(5)
N(6A)-C(41A)-C(36A)	108.0(4)
C(34A)-N(6A)-C(42A)	128.8(3)
C(41A)-N(6A)-C(34A)	107.8(4)
C(41A)-N(6A)-C(42A)	123.3(3)
N(6A)-C(42A)-C(44A)	114.8(3)
C(43A)-C(42A)-N(6A)	121.4(3)
C(43A)-C(42A)-C(44A)	123.1(3)
C(42A)-C(43A)-H(43C)	120.0
C(42A)-C(43A)-H(43D)	120.0
H(43C)-C(43A)-H(43D)	120.0
O(5A)-C(44A)-C(42A)	123.9(6)
O(5A)-C(44A)-O(6A)	113.2(5)
O(6A)-C(44A)-C(42A)	117.4(3)
C(44A)-O(6A)-C(45A)	124.7(3)
O(6A)-C(45A)-H(45C)	110.5
O(6A)-C(45A)-H(45D)	110.5
O(6A)-C(45A)-C(46A)	105.9(4)
H(45C)-C(45A)-H(45D)	108.7
C(46A)-C(45A)-H(45C)	110.5
C(46A)-C(45A)-H(45D)	110.5
C(45A)-C(46A)-H(46D)	109.5
C(45A)-C(46A)-H(46E)	109.5
C(45A)-C(46A)-H(46F)	109.5
H(46D)-C(46A)-H(46E)	109.5
H(46D)-C(46A)-H(46F)	109.5
H(46E)-C(46A)-H(46F)	109.5

Symmetry transformations used to generate equivalent atoms:

#1 -x+2,-y+2,-z+1 #2 x+1,y,z #3 x,y-1,z #4 -x+1,-y+1,-z+2

Table S24 Anisotropic displacement parameters ($Å^2 x 10^3$) for P093. The anisotropic displacement factor exponent takes the form: $-2p^2[h^2 a^{*2}U^{11} + ... + 2 h k a^* b^* U^{12}]$

	U^{11}	U^{22}	U^{33}	U^{23}	U^{13}	U^{12}
O(1)	42(1)	20(1)	25(1)	1(1)	6(1)	5(1)
N(1)	17(1)	19(1)	20(1)	2(1)	-4(1)	-6(1)
N(2)	17(1)	20(1)	23(1)	5(1)	-1(1)	-2(1)
N(3)	24(1)	26(1)	19(1)	5(1)	-8(1)	-12(1)
C(1)	15(1)	19(1)	18(1)	1(1)	0(1)	-5(1)
C(2)	18(1)	20(1)	21(1)	0(1)	-3(1)	-5(1)
C(3)	23(1)	28(1)	18(1)	2(1)	-3(1)	-8(1)
C(4)	40(1)	28(1)	18(1)	-2(1)	-2(1)	-13(1)
C(5)	38(1)	21(1)	20(1)	-3(1)	3(1)	-8(1)
C(6)	21(1)	20(1)	18(1)	1(1)	3(1)	-5(1)
C(7)	22(1)	20(1)	20(1)	2(1)	6(1)	-2(1)
C(8)	16(1)	26(1)	32(1)	11(1)	-2(1)	0(1)
C(9)	19(1)	32(1)	29(1)	14(1)	-4(1)	-4(1)
C(10)	18(1)	32(1)	24(1)	11(1)	-5(1)	-10(1)
C(11)	18(1)	25(1)	22(1)	6(1)	-5(1)	-9(1)
C(12)	14(1)	19(1)	22(1)	4(1)	-2(1)	-6(1)
C(13)	20(1)	42(1)	22(1)	12(1)	-6(1)	-17(1)
C(14)	27(1)	57(1)	26(1)	19(1)	-12(1)	-22(1)
C(15)	41(1)	72(1)	24(1)	18(1)	-17(1)	-36(1)
C(16)	47(1)	63(1)	23(1)	9(1)	-13(1)	-38(1)
C(17)	36(1)	45(1)	21(1)	7(1)	-9(1)	-26(1)
C(18)	23(1)	39(1)	19(1)	8(1)	-7(1)	-19(1)
C(19)	37(1)	22(1)	21(1)	6(1)	-13(1)	-18(1)
C(20)	45(1)	19(1)	28(1)	4(1)	-17(1)	-12(1)
O(2)	24(2)	21(2)	22(1)	3(1)	-6(1)	-11(1)
C(21)	26(2)	14(2)	20(1)	2(1)	-13(1)	-7(1)
O(3)	19(1)	18(1)	18(1)	3(1)	-6(1)	-2(1)
C(22)	22(1)	25(1)	15(1)	-1(1)	-3(1)	-9(1)
C(23)	25(1)	26(1)	19(1)	2(1)	-3(1)	-11(1)
O(2A)	26(2)	19(2)	24(1)	3(1)	-4(1)	-7(1)

C(21A)	29(3)	18(2)	24(2)	10(1)	-18(2)	-13(2)
O(3A)	18(1)	20(1)	20(1)	3(1)	-5(1)	0(1)
C(22A)	22(1)	23(1)	16(1)	2(1)	1(1)	-5(1)
C(23A)	26(1)	20(1)	18(1)	-1(1)	-3(1)	-6(1)
C(24)	14(1)	17(2)	16(1)	-2(1)	-5(1)	-5(1)
C(25)	17(1)	23(2)	17(1)	-1(1)	0(1)	-6(1)
C(26)	20(1)	21(2)	20(1)	-1(1)	0(1)	-7(1)
C(27)	21(1)	19(2)	26(1)	0(1)	-3(1)	-8(1)
C(28)	21(1)	19(2)	22(1)	-4(1)	-7(1)	-4(1)
C(29)	17(1)	17(2)	15(2)	-2(1)	-4(1)	-6(1)
C(30)	20(1)	14(2)	17(2)	-5(1)	-7(1)	1(1)
O(4)	34(2)	17(1)	17(2)	-4(1)	-4(1)	-5(1)
N(5)	17(1)	14(1)	14(1)	-3(1)	-1(1)	-5(1)
C(31)	17(1)	21(1)	14(1)	-4(1)	0(1)	-5(1)
C(32)	18(1)	18(1)	14(1)	-2(1)	0(1)	-6(1)
C(33)	16(1)	18(2)	11(1)	-2(1)	-1(1)	-4(2)
C(34)	16(1)	13(2)	14(1)	-1(1)	-3(1)	-5(1)
C(35)	14(1)	16(2)	14(1)	-5(1)	-2(1)	0(1)
N(4)	17(1)	18(2)	16(1)	0(1)	-1(1)	-6(1)
C(36)	16(1)	15(2)	20(1)	-2(1)	-4(1)	-4(2)
C(37)	19(2)	25(2)	14(1)	4(1)	-2(1)	-6(1)
C(38)	24(1)	21(1)	22(1)	4(1)	-4(1)	-9(1)
C(39)	19(1)	18(2)	20(1)	1(2)	-1(1)	-6(2)
C(40)	18(2)	16(2)	21(1)	-3(2)	-2(1)	-3(2)
C(41)	14(1)	17(2)	13(1)	1(1)	0(1)	-4(1)
N(6)	19(1)	15(1)	14(1)	-4(1)	1(1)	-5(1)
C(42)	18(1)	19(1)	13(1)	-4(1)	0(1)	-8(1)
C(43)	19(1)	20(1)	18(1)	-2(1)	-3(1)	-6(1)
O(5)	26(2)	21(1)	15(2)	-3(1)	-4(1)	-4(2)
C(44)	22(2)	13(1)	12(1)	-4(1)	-3(1)	-7(1)
O(6)	23(1)	22(1)	13(1)	2(1)	0(1)	-2(1)
C(45)	24(2)	21(1)	16(1)	1(1)	-2(1)	-2(1)
C(46)	35(2)	24(1)	18(2)	-1(1)	-2(2)	-7(2)
C(24A)	16(1)	17(2)	21(2)	-1(1)	-4(1)	-6(1)
C(25A)	16(1)	24(2)	20(1)	-5(1)	-2(1)	0(1)
C(26A)	19(1)	19(2)	24(1)	-1(1)	-3(1)	-5(1)
C(27A)	24(1)	20(2)	28(1)	-2(1)	-9(1)	-6(1)

C(28A)	24(2)	19(2)	24(1)	-2(1)	-9(1)	-4(1)
C(29A)	20(1)	20(2)	17(1)	-6(1)	-7(1)	-1(2)
C(30A)	22(1)	13(2)	18(2)	-3(1)	-6(1)	-2(1)
O(4A)	36(2)	23(2)	19(2)	-6(1)	-6(1)	-4(1)
N(5A)	18(1)	16(2)	16(1)	-6(1)	-3(1)	-3(1)
C(31A)	22(1)	24(1)	14(1)	-4(1)	-3(1)	-4(1)
C(32A)	21(1)	25(2)	15(1)	-6(1)	-2(1)	-3(1)
C(33A)	14(1)	18(2)	20(1)	0(1)	-6(1)	-5(2)
C(34A)	15(2)	20(2)	16(1)	-7(1)	-3(1)	-2(1)
C(35A)	15(1)	16(2)	18(2)	-2(1)	-5(1)	-2(1)
N(4A)	15(1)	18(2)	17(1)	-2(1)	-2(1)	-2(2)
C(36A)	16(1)	21(2)	11(1)	-1(2)	-2(1)	-4(2)
C(37A)	18(1)	26(2)	18(1)	-1(1)	-3(1)	-5(1)
C(38A)	24(2)	26(2)	20(1)	4(1)	0(1)	-10(1)
C(39A)	27(2)	20(2)	28(1)	-2(2)	-5(1)	-8(2)
C(40A)	20(2)	21(3)	16(2)	0(2)	1(1)	-5(2)
C(41A)	16(1)	16(2)	20(1)	-2(1)	-5(1)	-3(1)
N(6A)	18(1)	18(1)	14(1)	-1(1)	0(1)	-6(1)
C(42A)	18(1)	17(1)	14(1)	-2(1)	-3(1)	-6(1)
C(43A)	18(1)	20(1)	16(1)	-2(1)	-1(1)	-4(1)
O(5A)	25(2)	24(1)	13(2)	-4(1)	-6(1)	-4(2)
C(44A)	22(2)	18(1)	13(1)	-6(1)	-3(1)	-9(1)
O(6A)	22(1)	21(1)	14(1)	0(1)	-1(1)	-2(1)
C(45A)	26(2)	21(1)	16(1)	4(1)	-4(1)	-3(1)
C(46A)	27(2)	26(1)	15(2)	-2(1)	1(1)	-4(2)

Table S25 Hydrogen coordinates (x 10^4) and isotropic displacement parameters (Å^{2}x 10^{3}) for P093.

	x	y	z	U(eq)
H(2)	10847	7701	7140	24
H(3)	11486	8354	8134	28
H(4)	10637	10384	8355	33
H(5)	9069	11759	7597	32

H(8A)	6493	12542	5446	32
H(8B)	5437	11568	5644	32
H(9A)	5801	11803	4425	34
H(9B)	7672	11789	4378	34
H(14)	5934	10494	3266	43
H(15)	5958	8939	2601	50
H(16)	7149	6956	2961	48
H(17)	8381	6458	4000	38
H(20A)	10963	6569	4487	35
H(20B)	11068	5680	5233	35
H(22A)	8369	6284	7323	24
H(22B)	9978	5235	7363	24
H(23A)	7148	4983	6891	34
H(23B)	7728	4416	7641	34
H(23C)	8763	3935	6910	34
H(22C)	7161	5708	7129	25
H(22D)	8442	6146	7483	25
H(23D)	9076	3860	6942	32
H(23E)	8651	4067	7775	32
H(23F)	10301	4296	7327	32
H(25)	5672	6994	7561	23
H(26)	6713	8668	7367	25
H(27)	6538	9856	8296	26
H(28)	5121	9430	9425	24
H(31A)	724	6843	10418	21
H(31B)	1959	6882	10944	21
H(32A)	2822	4835	11078	20
H(32B)	902	5078	11123	20
H(37)	867	2645	11105	24
H(38)	759	828	10729	27
H(39)	1683	392	9539	23
H(40)	2761	1736	8706	23
H(43A)	5351	2376	8425	22
H(43B)	5480	3040	7607	22
H(45A)	3864	6678	6681	25
H(45B)	2643	5844	6681	25
H(46A)	4422	5826	5579	39

H(46B)	4673	4507	5987	39
H(46C)	5928	5299	6005	39
H(25A)	5449	7406	7629	25
H(26A)	6473	9110	7444	25
H(27A)	6231	10300	8379	28
H(28A)	4909	9805	9508	26
H(31C)	2161	7028	11065	24
H(31D)	3641	5872	10990	24
H(32C)	1428	5106	11270	24
H(32D)	444	6079	10728	24
H(37A)	298	3105	11045	25
H(38A)	150	1337	10638	28
H(39A)	1500	766	9512	30
H(40A)	3058	1944	8771	24
H(43C)	5782	2555	8572	22
H(43D)	5961	3199	7750	22
H(45C)	3425	6592	6639	26
H(45D)	2215	5771	6587	26
H(46D)	3950	5789	5493	35
H(46E)	4392	4467	5889	35
H(46F)	5498	5376	5900	35

Table S26 Torsion angles [°] for P093.

N(1)-C(1)-C(2)-C(3)	178.74(9)
N(1)-C(1)-C(6)-C(5)	-178.97(10)
N(1)-C(1)-C(6)-C(7)	3.20(15)
N(2)-C(8)-C(9)-C(10)	44.29(13)
N(3)-C(11)-C(12)-N(1)	4.19(16)
N(3)-C(11)-C(12)-N(2)	-176.56(9)
N(3)-C(19)-C(21)-O(2)	21.0(10)
N(3)-C(19)-C(21)-O(3)	-162.7(4)
N(3)-C(19)-C(21A)-O(2A)	9.8(11)
N(3)-C(19)-C(21A)-O(3A)	-171.8(5)
C(1)-N(1)-C(12)-N(2)	0.76(15)
C(1)-N(1)-C(12)-C(11)	179.93(9)

C(1)-C(2)-C(3)-C(4)	0.45(16)
C(1)-C(6)-C(7)-O(1)	176.30(10)
C(1)-C(6)-C(7)-N(2)	-2.78(14)
C(2)-C(1)-C(6)-C(5)	1.57(15)
C(2)-C(1)-C(6)-C(7)	-176.26(9)
C(2)-C(3)-C(4)-C(5)	1.13(17)
C(3)-C(4)-C(5)-C(6)	-1.34(18)
C(4)-C(5)-C(6)-C(1)	-0.01(17)
C(4)-C(5)-C(6)-C(7)	177.79(10)
C(5)-C(6)-C(7)-O(1)	-1.52(17)
C(5)-C(6)-C(7)-N(2)	179.39(10)
C(6)-C(1)-C(2)-C(3)	-1.78(15)
C(7)-N(2)-C(8)-C(9)	152.01(10)
C(7)-N(2)-C(12)-N(1)	-0.57(15)
C(7)-N(2)-C(12)-C(11)	-179.78(9)
C(8)-N(2)-C(7)-O(1)	-9.06(15)
C(8)-N(2)-C(7)-C(6)	170.06(9)
C(8)-N(2)-C(12)-N(1)	-168.54(10)
C(8)-N(2)-C(12)-C(11)	12.24(13)
C(8)-C(9)-C(10)-C(11)	-27.84(14)
C(8)-C(9)-C(10)-C(13)	156.77(11)
C(9)-C(10)-C(11)-N(3)	-174.71(9)
C(9)-C(10)-C(11)-C(12)	1.72(16)
C(9)-C(10)-C(13)-C(14)	-7.5(2)
C(9)-C(10)-C(13)-C(18)	174.96(11)
C(10)-C(11)-C(12)-N(1)	-171.70(10)
C(10)-C(11)-C(12)-N(2)	7.56(15)
C(10)-C(13)-C(14)-C(15)	-176.23(12)
C(10)-C(13)-C(18)-N(3)	-0.20(12)
C(10)-C(13)-C(18)-C(17)	177.15(10)
C(11)-N(3)-C(18)-C(13)	1.20(12)
C(11)-N(3)-C(18)-C(17)	-175.87(12)
C(11)-N(3)-C(19)-C(20)	-126.06(12)
C(11)-N(3)-C(19)-C(21)	58.5(5)
C(11)-N(3)-C(19)-C(21A)	60.8(4)
C(11)-C(10)-C(13)-C(14)	176.66(12)
C(11)-C(10)-C(13)-C(18)	-0.90(12)

C(12)-N(1)-C(1)-C(2)	177.35(9)
C(12)-N(1)-C(1)-C(6)	-2.12(14)
C(12)-N(2)-C(7)-O(1)	-177.59(10)
C(12)-N(2)-C(7)-C(6)	1.53(14)
C(12)-N(2)-C(8)-C(9)	-39.48(14)
C(13)-C(10)-C(11)-N(3)	1.68(12)
C(13)-C(10)-C(11)-C(12)	178.12(10)
C(13)-C(14)-C(15)-C(16)	-0.56(19)
C(14)-C(13)-C(18)-N(3)	-178.17(10)
C(14)-C(13)-C(18)-C(17)	-0.81(17)
C(14)-C(15)-C(16)-C(17)	-0.3(2)
C(15)-C(16)-C(17)-C(18)	0.59(19)
C(16)-C(17)-C(18)-N(3)	176.69(11)
C(16)-C(17)-C(18)-C(13)	-0.03(17)
C(18)-N(3)-C(11)-C(10)	-1.82(12)
C(18)-N(3)-C(11)-C(12)	-178.20(10)
C(18)-N(3)-C(19)-C(20)	54.88(15)
C(18)-N(3)-C(19)-C(21)	-120.6(5)
C(18)-N(3)-C(19)-C(21A)	-118.3(4)
C(18)-C(13)-C(14)-C(15)	1.09(17)
C(19)-N(3)-C(11)-C(10)	179.00(10)
C(19)-N(3)-C(11)-C(12)	2.62(17)
C(19)-N(3)-C(18)-C(13)	-179.57(10)
C(19)-N(3)-C(18)-C(17)	3.35(18)
C(19)-C(21)-O(3)-C(22)	177.6(4)
C(19)-C(21A)-O(3A)-C(22A)	-177.3(4)
C(20)-C(19)-C(21)-O(2)	-154.6(6)
C(20)-C(19)-C(21)-O(3)	21.6(8)
C(20)-C(19)-C(21A)-O(2A)	-162.6(7)
C(20)-C(19)-C(21A)-O(3A)	15.8(9)
O(2)-C(21)-O(3)-C(22)	-6.7(12)
C(21)-O(3)-C(22)-C(23)	80.0(5)
O(2A)-C(21A)-O(3A)-C(22A)	1.2(11)
C(21A)-O(3A)-C(22A)-C(23A)	155.3(6)
C(24)-C(25)-C(26)-C(27)	1.1(6)
C(24)-C(29)-C(30)-O(4)	-173.3(5)
C(24)-C(29)-C(30)-N(5)	2.8(7)

C(25)-C(24)-C(29)-C(28)	-2.6(6)
C(25)-C(24)-C(29)-C(30)	175.8(4)
C(25)-C(24)-N(4)-C(35)	-177.1(5)
C(25)-C(26)-C(27)-C(28)	-2.5(5)
C(26)-C(27)-C(28)-C(29)	1.3(6)
C(27)-C(28)-C(29)-C(24)	1.2(6)
C(27)-C(28)-C(29)-C(30)	-177.2(4)
C(28)-C(29)-C(30)-O(4)	5.1(8)
C(28)-C(29)-C(30)-N(5)	-178.7(4)
C(29)-C(24)-C(25)-C(26)	1.5(6)
C(29)-C(24)-N(4)-C(35)	4.8(8)
C(29)-C(30)-N(5)-C(31)	-168.0(4)
C(29)-C(30)-N(5)-C(35)	1.3(7)
C(30)-N(5)-C(31)-C(32)	-148.6(4)
C(30)-N(5)-C(35)-C(34)	175.3(5)
C(30)-N(5)-C(35)-N(4)	-2.7(8)
O(4)-C(30)-N(5)-C(31)	8.3(8)
O(4)-C(30)-N(5)-C(35)	177.5(5)
N(5)-C(31)-C(32)-C(33)	-43.5(5)
N(5)-C(35)-N(4)-C(24)	-0.3(8)
C(31)-N(5)-C(35)-C(34)	-15.8(6)
C(31)-N(5)-C(35)-N(4)	166.2(5)
C(31)-C(32)-C(33)-C(34)	24.3(8)
C(31)-C(32)-C(33)-C(36)	-162.0(7)
C(32)-C(33)-C(34)-C(35)	1.1(10)
C(32)-C(33)-C(34)-N(6)	175.0(5)
C(32)-C(33)-C(36)-C(37)	6.1(13)
C(32)-C(33)-C(36)-C(41)	-173.8(7)
C(33)-C(34)-C(35)-N(5)	-6.7(8)
C(33)-C(34)-C(35)-N(4)	171.4(6)
C(33)-C(34)-N(6)-C(41)	-0.4(6)
C(33)-C(34)-N(6)-C(42)	-175.2(5)
C(33)-C(36)-C(37)-C(38)	179.9(7)
C(33)-C(36)-C(41)-C(40)	-179.2(6)
C(33)-C(36)-C(41)-N(6)	-0.8(8)
C(34)-C(33)-C(36)-C(37)	-179.6(7)
C(34)-C(33)-C(36)-C(41)	0.5(8)

C(34)-C(35)-N(4)-C(24)	-178.2(5)
C(34)-N(6)-C(42)-C(43)	124.6(4)
C(34)-N(6)-C(42)-C(44)	-61.7(5)
C(35)-N(5)-C(31)-C(32)	42.1(5)
C(35)-C(34)-N(6)-C(41)	173.4(5)
C(35)-C(34)-N(6)-C(42)	-1.4(7)
N(4)-C(24)-C(25)-C(26)	-176.7(4)
N(4)-C(24)-C(29)-C(28)	175.4(5)
N(4)-C(24)-C(29)-C(30)	-6.1(7)
C(36)-C(33)-C(34)-C(35)	-174.0(5)
C(36)-C(33)-C(34)-N(6)	-0.1(7)
C(36)-C(37)-C(38)-C(39)	-0.4(6)
C(36)-C(41)-N(6)-C(34)	0.7(7)
C(36)-C(41)-N(6)-C(42)	175.9(4)
C(37)-C(36)-C(41)-C(40)	0.8(10)
C(37)-C(36)-C(41)-N(6)	179.3(5)
C(37)-C(38)-C(39)-C(40)	0.5(8)
C(38)-C(39)-C(40)-C(41)	0.1(10)
C(39)-C(40)-C(41)-C(36)	-0.8(10)
C(39)-C(40)-C(41)-N(6)	-178.8(7)
C(40)-C(41)-N(6)-C(34)	179.0(6)
C(40)-C(41)-N(6)-C(42)	-5.9(9)
C(41)-C(36)-C(37)-C(38)	-0.2(8)
C(41)-N(6)-C(42)-C(43)	-49.5(5)
C(41)-N(6)-C(42)-C(44)	124.2(5)
N(6)-C(34)-C(35)-N(5)	-179.6(4)
N(6)-C(34)-C(35)-N(4)	-1.5(8)
N(6)-C(42)-C(44)-O(5)	-14.2(7)
N(6)-C(42)-C(44)-O(6)	149.3(3)
C(42)-C(44)-O(6)-C(45)	166.8(3)
C(43)-C(42)-C(44)-O(5)	159.3(6)
C(43)-C(42)-C(44)-O(6)	-37.2(4)
O(5)-C(44)-O(6)-C(45)	-30.4(7)
C(44)-O(6)-C(45)-C(46)	-138.1(4)
C(24A)-C(25A)-C(26A)-C(27A)	-2.1(6)
C(24A)-C(29A)-C(30A)-O(4A)	179.5(6)
C(24A)-C(29A)-C(30A)-N(5A)	1.9(7)

C(25A)-C(24A)-C(29A)-C(28A)	-3.0(7)
C(25A)-C(24A)-C(29A)-C(30A)	178.6(5)
C(25A)-C(24A)-N(4A)-C(35A)	179.6(5)
C(25A)-C(26A)-C(27A)-C(28A)	-0.9(5)
C(26A)-C(27A)-C(28A)-C(29A)	1.9(6)
C(27A)-C(28A)-C(29A)-C(24A)	0.0(7)
C(27A)-C(28A)-C(29A)-C(30A)	178.4(5)
C(28A)-C(29A)-C(30A)-O(4A)	1.0(9)
C(28A)-C(29A)-C(30A)-N(5A)	-176.5(5)
C(29A)-C(24A)-C(25A)-C(26A)	4.0(6)
C(29A)-C(24A)-N(4A)-C(35A)	-0.3(8)
C(29A)-C(30A)-N(5A)-C(31A)	170.9(4)
C(29A)-C(30A)-N(5A)-C(35A)	-0.7(8)
C(30A)-N(5A)-C(31A)-C(32A)	151.9(4)
C(30A)-N(5A)-C(35A)-C(34A)	178.8(5)
C(30A)-N(5A)-C(35A)-N(4A)	-1.3(9)
O(4A)-C(30A)-N(5A)-C(31A)	-6.7(8)
O(4A)-C(30A)-N(5A)-C(35A)	-178.3(6)
N(5A)-C(31A)-C(32A)-C(33A)	44.2(5)
N(5A)-C(35A)-N(4A)-C(24A)	1.8(8)
C(31A)-N(5A)-C(35A)-C(34A)	7.6(7)
C(31A)-N(5A)-C(35A)-N(4A)	-172.4(5)
C(31A)-C(32A)-C(33A)-C(34A)	-28.4(8)
C(31A)-C(32A)-C(33A)-C(36A)	156.8(7)
C(32A)-C(33A)-C(34A)-C(35A)	0.5(10)
C(32A)-C(33A)-C(34A)-N(6A)	-176.8(5)
C(32A)-C(33A)-C(36A)-C(37A)	-0.7(13)
C(32A)-C(33A)-C(36A)-C(41A)	176.0(7)
C(33A)-C(34A)-C(35A)-N(5A)	11.3(8)
C(33A)-C(34A)-C(35A)-N(4A)	-168.6(6)
C(33A)-C(34A)-N(6A)-C(41A)	0.7(6)
C(33A)-C(34A)-N(6A)-C(42A)	175.9(5)
C(33A)-C(36A)-C(37A)-C(38A)	177.6(7)
C(33A)-C(36A)-C(41A)-C(40A)	-178.8(7)
C(33A)-C(36A)-C(41A)-N(6A)	-0.3(8)
C(34A)-C(33A)-C(36A)-C(37A)	-176.0(7)
C(34A)-C(33A)-C(36A)-C(41A)	0.7(8)

C(34A)-C(35A)-N(4A)-C(24A)	-178.3(5)
C(34A)-N(6A)-C(42A)-C(43A)	125.8(4)
C(34A)-N(6A)-C(42A)-C(44A)	-63.3(5)
C(35A)-N(5A)-C(31A)-C(32A)	-36.4(5)
C(35A)-C(34A)-N(6A)-C(41A)	-176.5(5)
C(35A)-C(34A)-N(6A)-C(42A)	-1.3(7)
N(4A)-C(24A)-C(25A)-C(26A)	-175.9(4)
N(4A)-C(24A)-C(29A)-C(28A)	176.9(5)
N(4A)-C(24A)-C(29A)-C(30A)	-1.5(7)
C(36A)-C(33A)-C(34A)-C(35A)	176.4(5)
C(36A)-C(33A)-C(34A)-N(6A)	-0.9(7)
C(36A)-C(37A)-C(38A)-C(39A)	-0.6(6)
C(36A)-C(41A)-N(6A)-C(34A)	-0.2(7)
C(36A)-C(41A)-N(6A)-C(42A)	-175.7(5)
C(37A)-C(36A)-C(41A)-C(40A)	-1.5(10)
C(37A)-C(36A)-C(41A)-N(6A)	177.0(5)
C(37A)-C(38A)-C(39A)-C(40A)	0.2(8)
C(38A)-C(39A)-C(40A)-C(41A)	-0.4(10)
C(39A)-C(40A)-C(41A)-C(36A)	1.1(10)
C(39A)-C(40A)-C(41A)-N(6A)	-177.0(8)
C(40A)-C(41A)-N(6A)-C(34A)	178.1(7)
C(40A)-C(41A)-N(6A)-C(42A)	2.6(10)
C(41A)-C(36A)-C(37A)-C(38A)	1.2(8)
C(41A)-N(6A)-C(42A)-C(43A)	-59.7(6)
C(41A)-N(6A)-C(42A)-C(44A)	111.3(5)
N(6A)-C(34A)-C(35A)-N(5A)	-171.9(5)
N(6A)-C(34A)-C(35A)-N(4A)	8.2(8)
N(6A)-C(42A)-C(44A)-O(5A)	-17.7(7)
N(6A)-C(42A)-C(44A)-O(6A)	-170.0(3)
C(42A)-C(44A)-O(6A)-C(45A)	178.7(3)
C(43A)-C(42A)-C(44A)-O(5A)	153.0(6)
C(43A)-C(42A)-C(44A)-O(6A)	0.8(5)
O(5A)-C(44A)-O(6A)-C(45A)	23.6(8)
C(44A)-O(6A)-C(45A)-C(46A)	179.8(4)

Symmetry transformations used to generate equivalent atoms:

#1 -x+2,-y+2,-z+1 #2 x+1,y,z #3 x,y-1,z #4 -x+1,-y+1,-z+2

Table S27 Hydrogen bonds for P093 [Å and °].

D-H...A	d(D-H)	d(H...A)	d(D...A)	<(DHA)
C(20)-H(20A)...O(1)#1	0.95	2.50	3.3933(16)	157.0
C(22)-H(22B)...O(5)#2	0.99	2.14	3.022(8)	147.1
C(23)-H(23A)...O(6)	0.98	2.33	3.059(4)	130.6
C(23)-H(23C)...O(1)#3	0.98	2.52	3.124(2)	119.5
C(32)-H(32A)...N(4)#4	0.99	2.61	3.538(7)	156.3
C(43)-H(43B)...O(1)#3	0.95	2.47	3.378(3)	160.0
C(31A)-H(31D)...N(6A)#4	0.99	2.57	3.361(4)	136.4
C(43A)-H(43C)...O(4A)#4	0.95	2.13	3.055(6)	164.5
C(43A)-H(43D)...O(1)#3	0.95	2.59	3.499(3)	161.2

Symmetry transformations used to generate equivalent atoms:

#1 -x+2,-y+2,-z+1 #2 x+1,y,z #3 x,y-1,z #4 -x+1,-y+1,-z+2

G. Copies of NMR Spectra

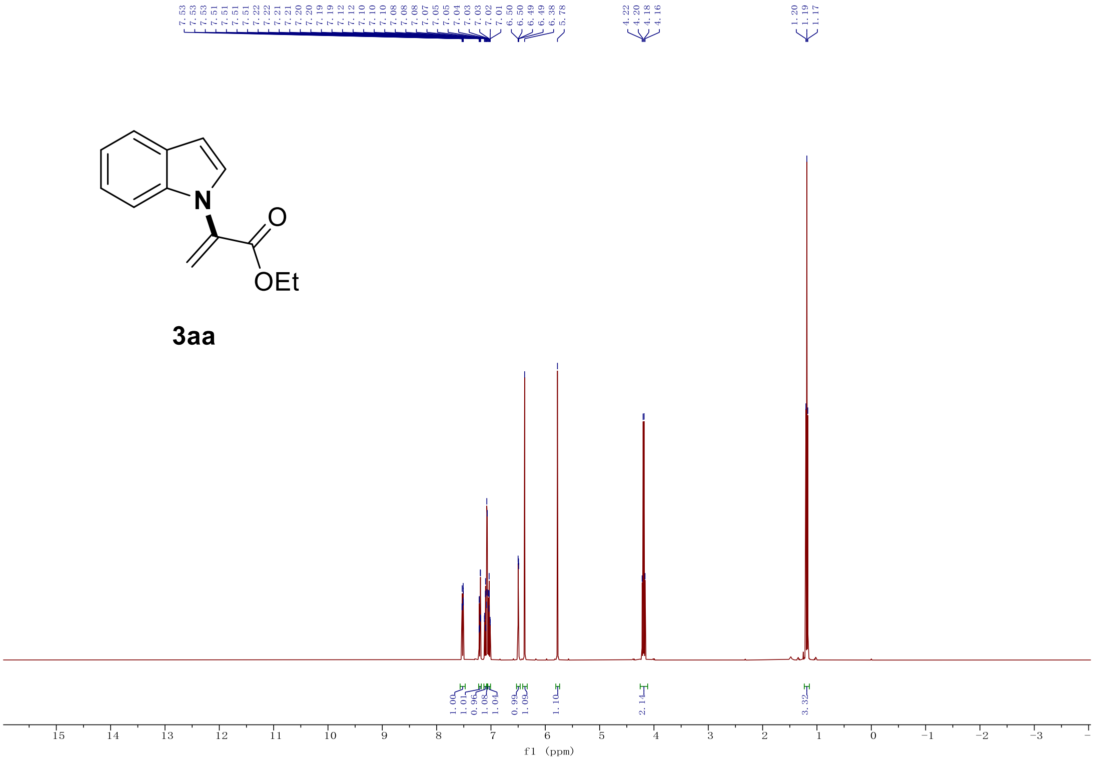

^{1}H NMR spectrum ($CDCl_3$, 400 MHz) of (**3aa**)

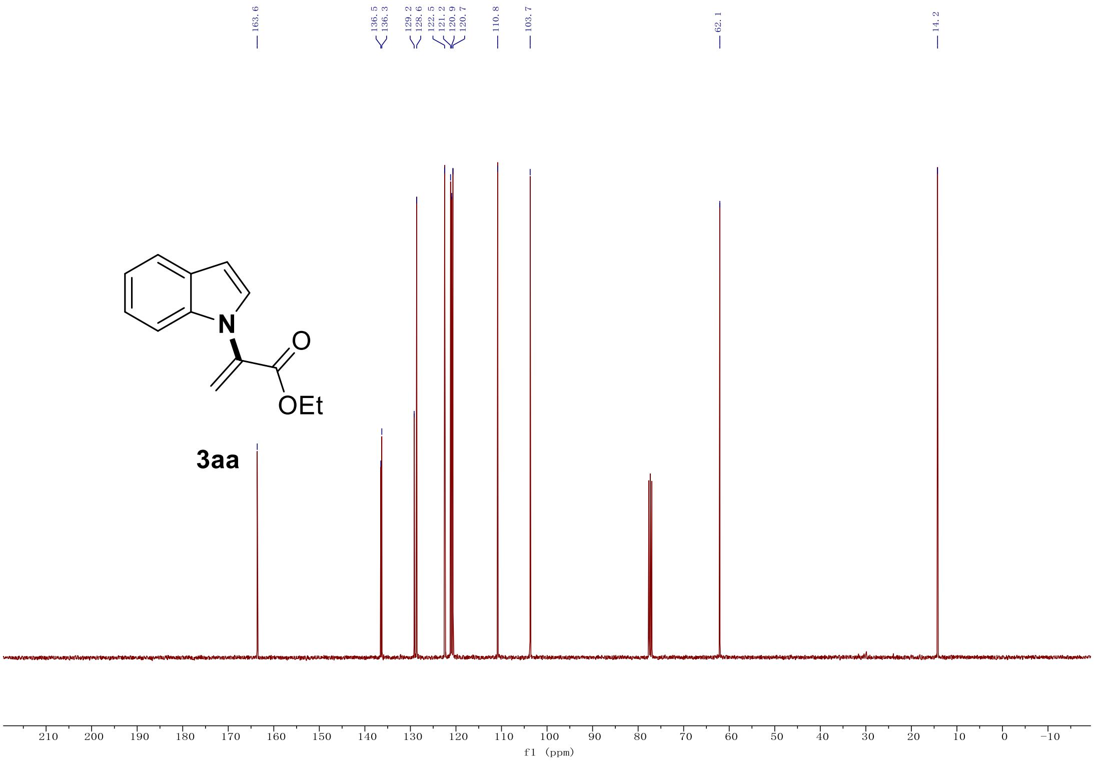

^{13}C NMR spectrum ($CDCl_3$, 101 MHz) of (**3aa**)

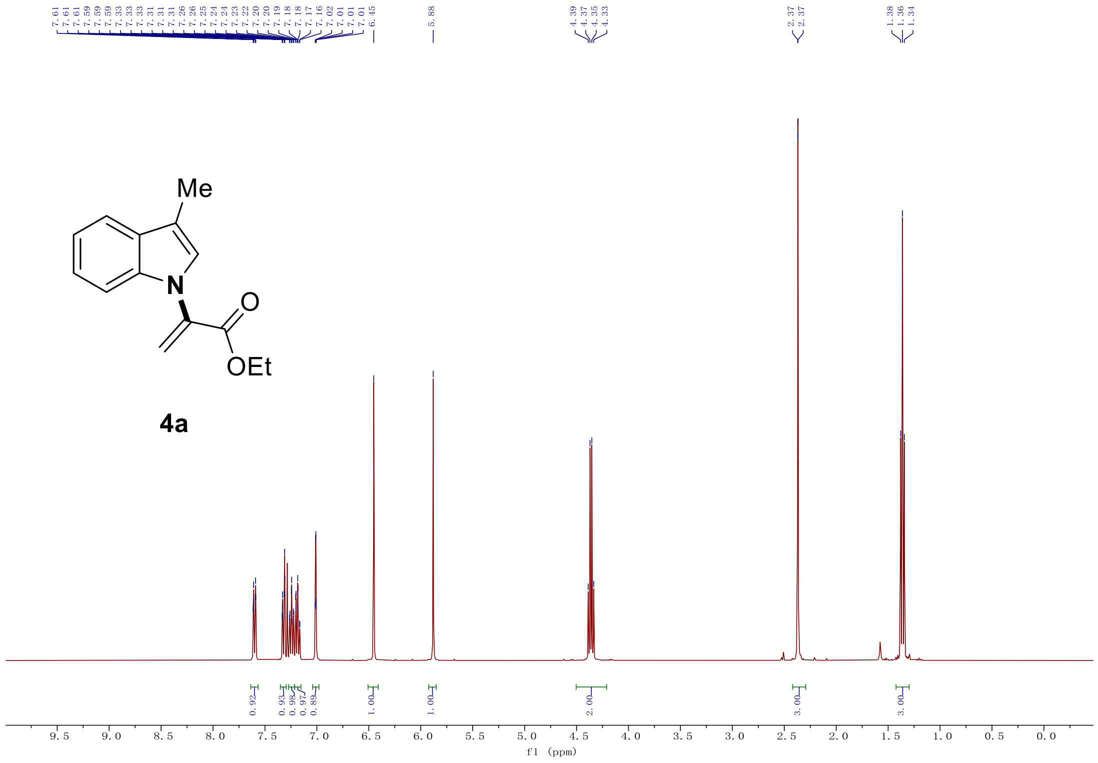

^{1}H NMR spectrum ($CDCl_3$, 400 MHz) of (**4a**)

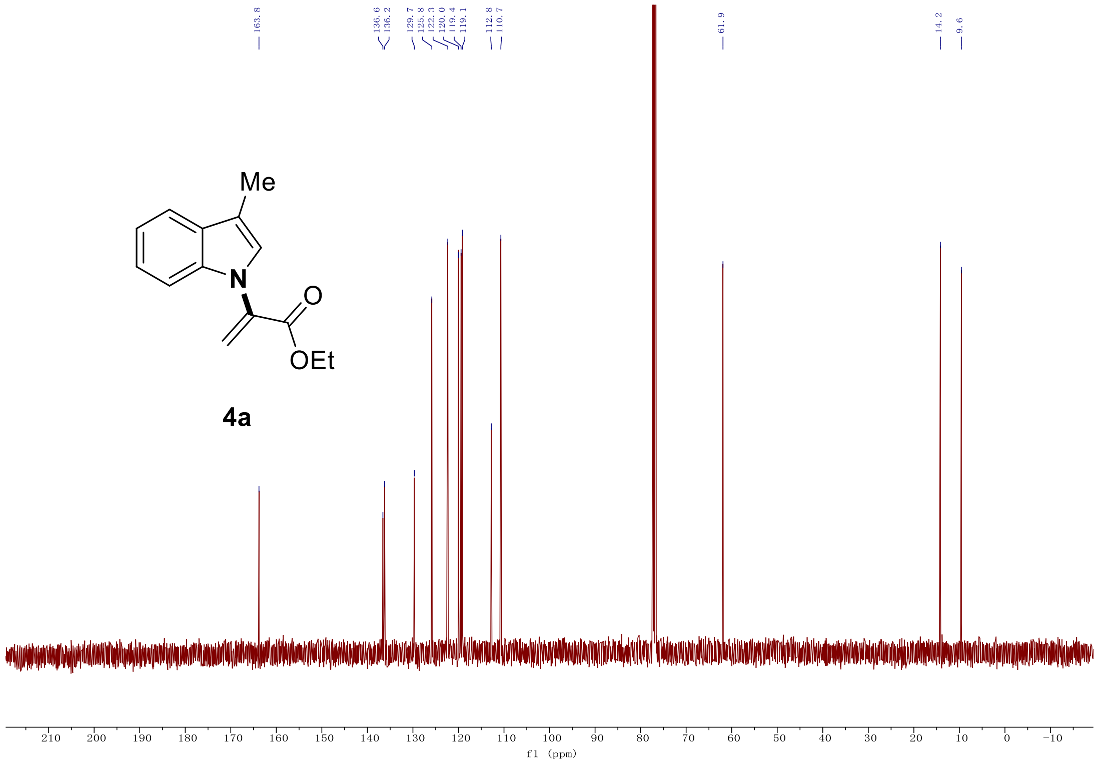

^{13}C NMR spectrum ($CDCl_3$, 101 MHz) of (**4a**)

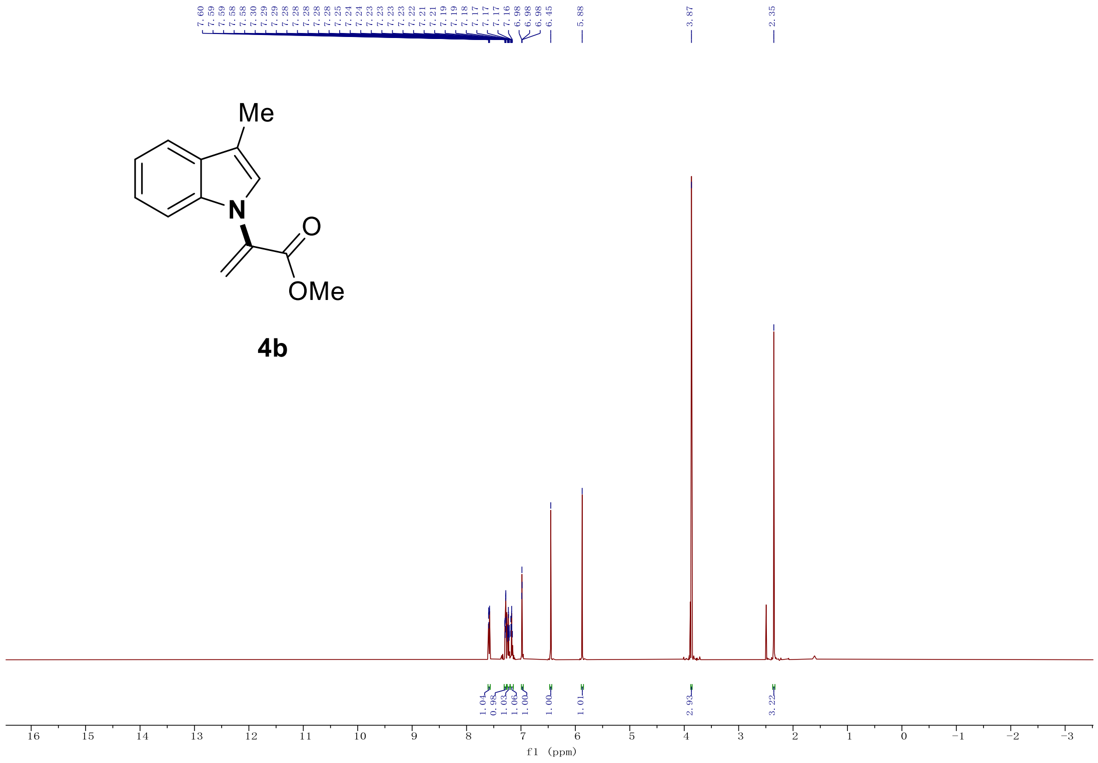

^{1}H NMR spectrum ($CDCl_3$, 400 MHz) of (**4b**)

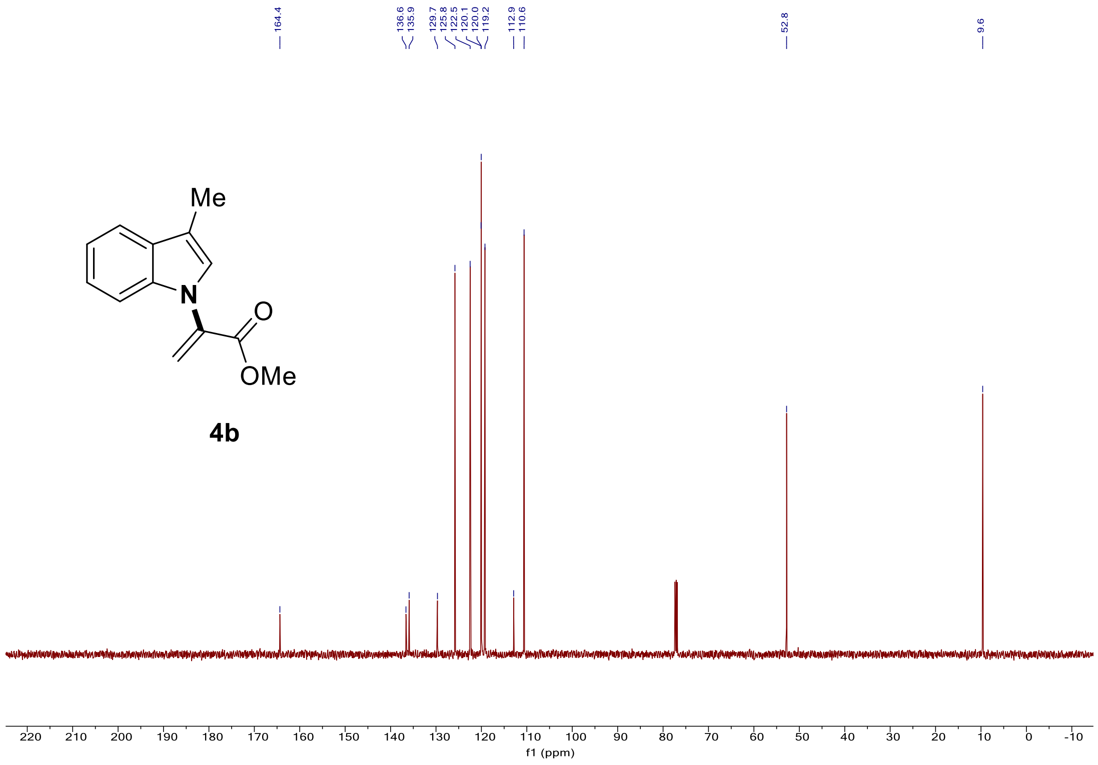

^{13}C NMR spectrum ($CDCl_3$, 101 MHz) of (**4b**)

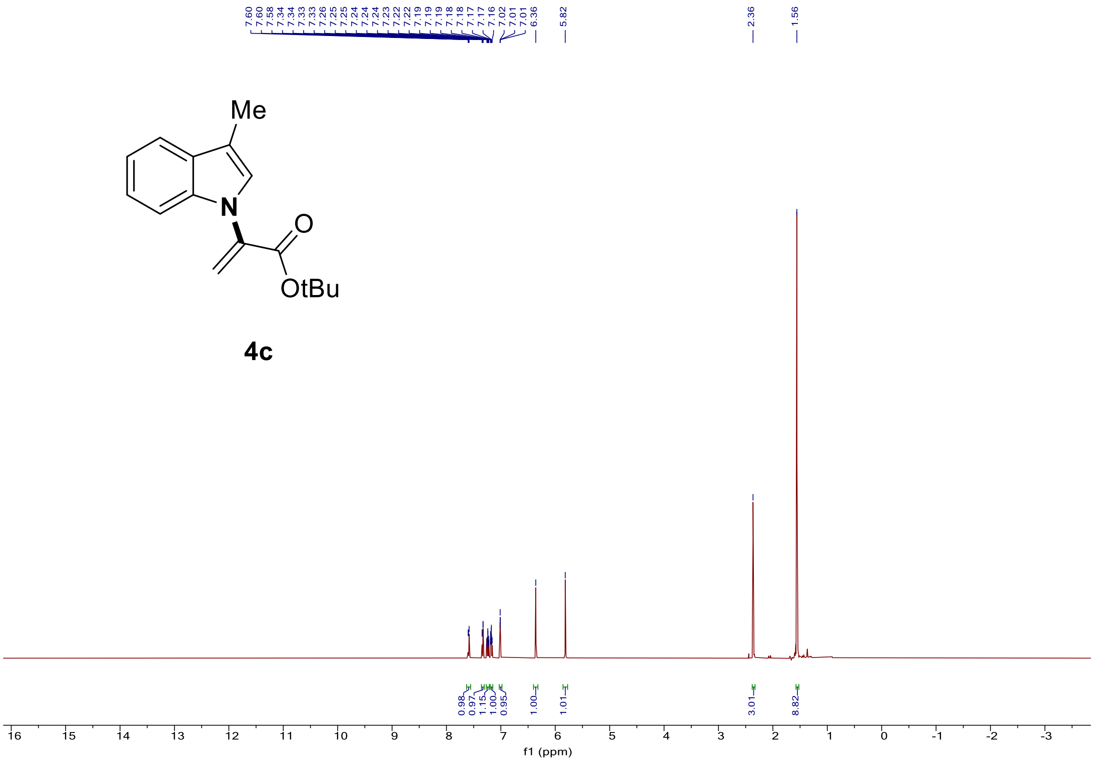

^1H NMR spectrum ($CDCl_3$, 500 MHz) of (**4c**)

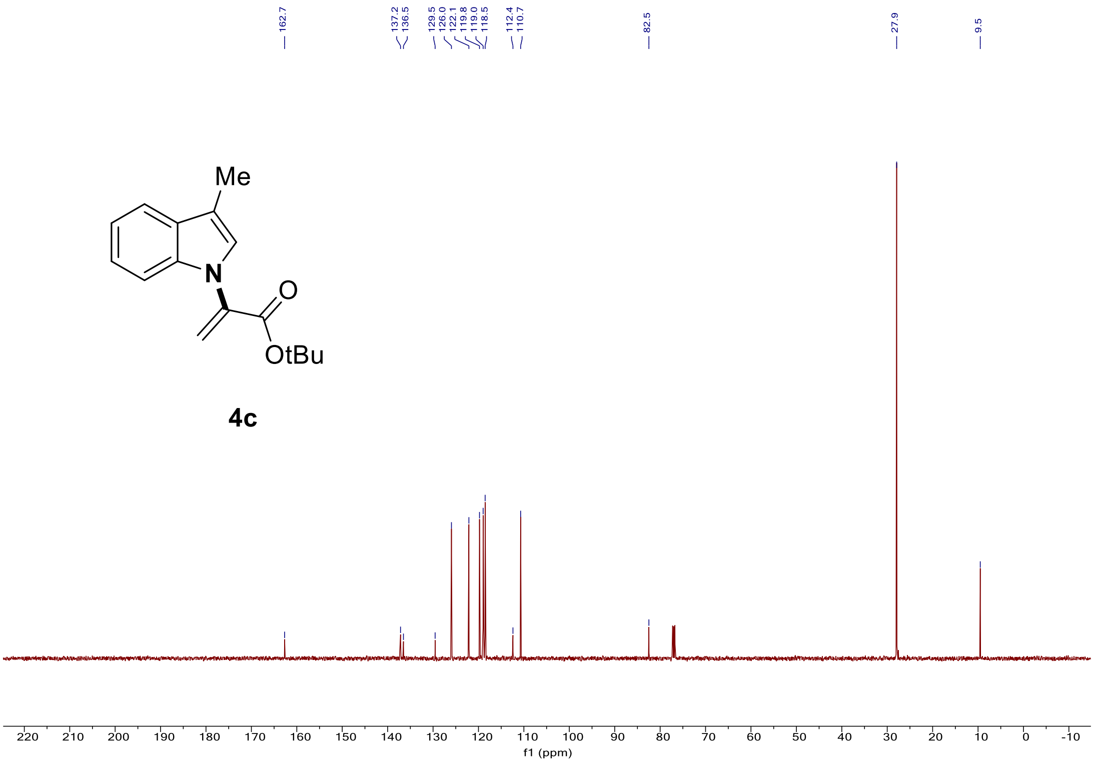

^{13}C NMR spectrum ($CDCl_3$, 126 MHz) of (**4c**)

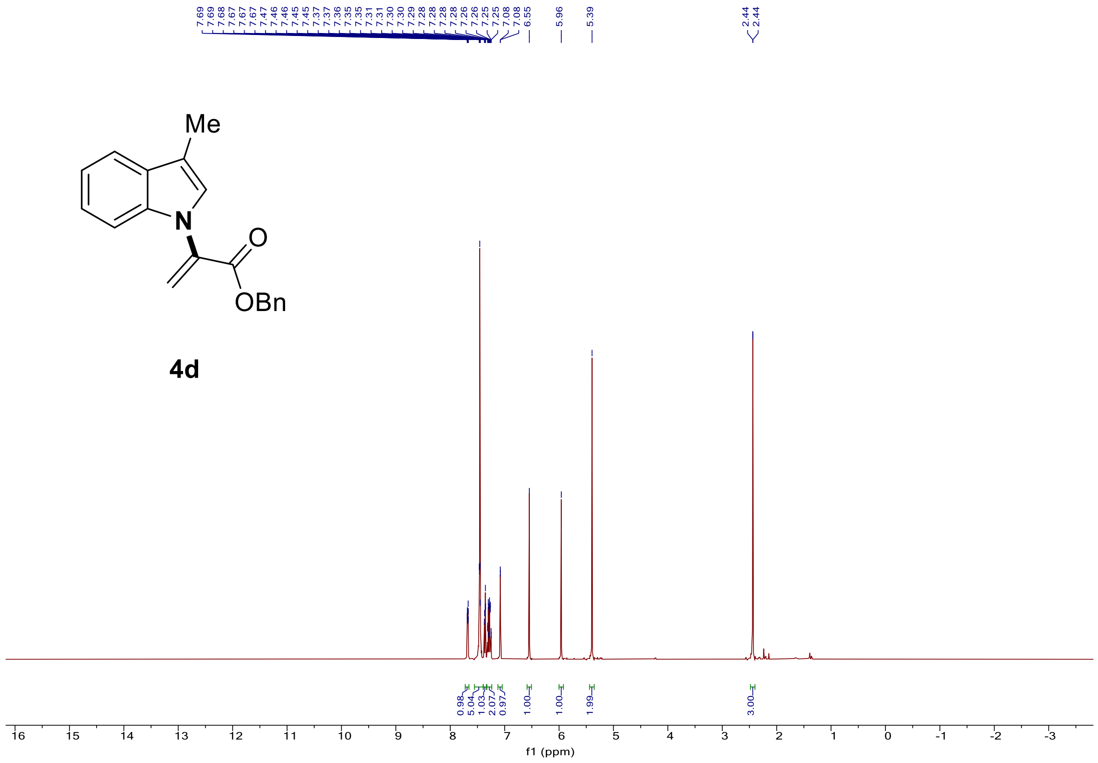

^{1}H NMR spectrum (CDCl$_3$, 500 MHz) of (**4d**)

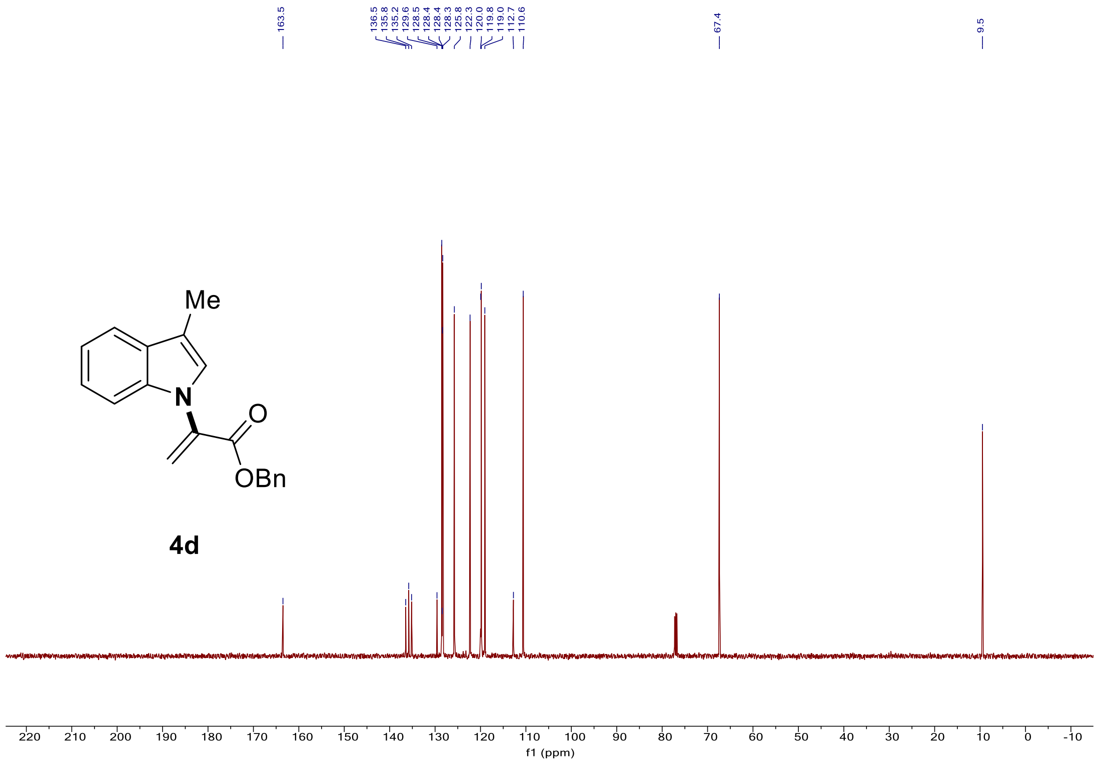

^{13}C NMR spectrum (CDCl$_3$, 126 MHz) of (**4d**)

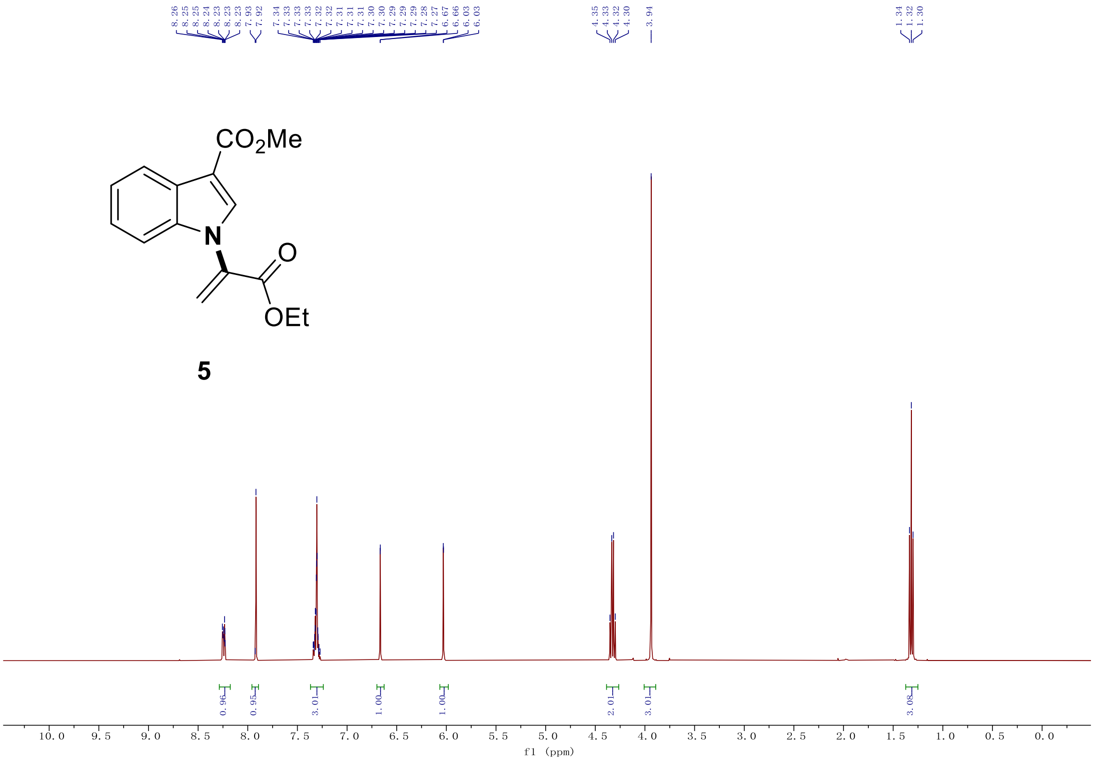

^{1}H NMR spectrum ($CDCl_3$, 400 MHz) of (**5**)

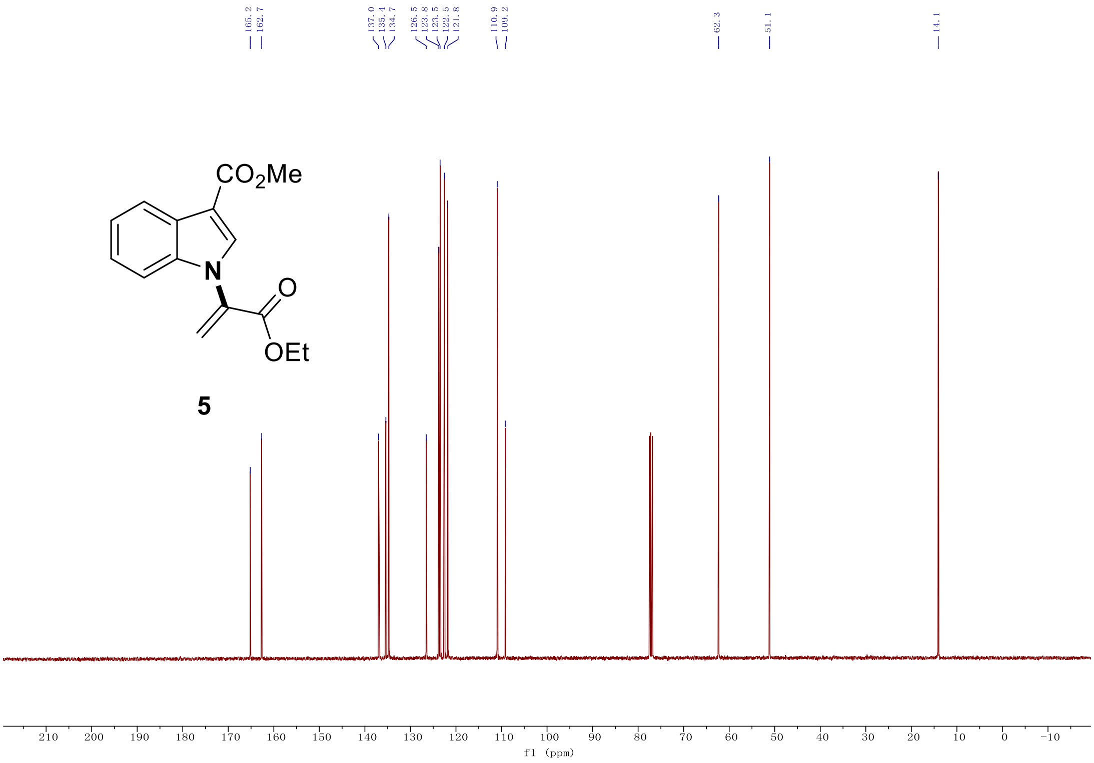

^{13}C NMR spectrum ($CDCl_3$, 101 MHz) of (**5**)

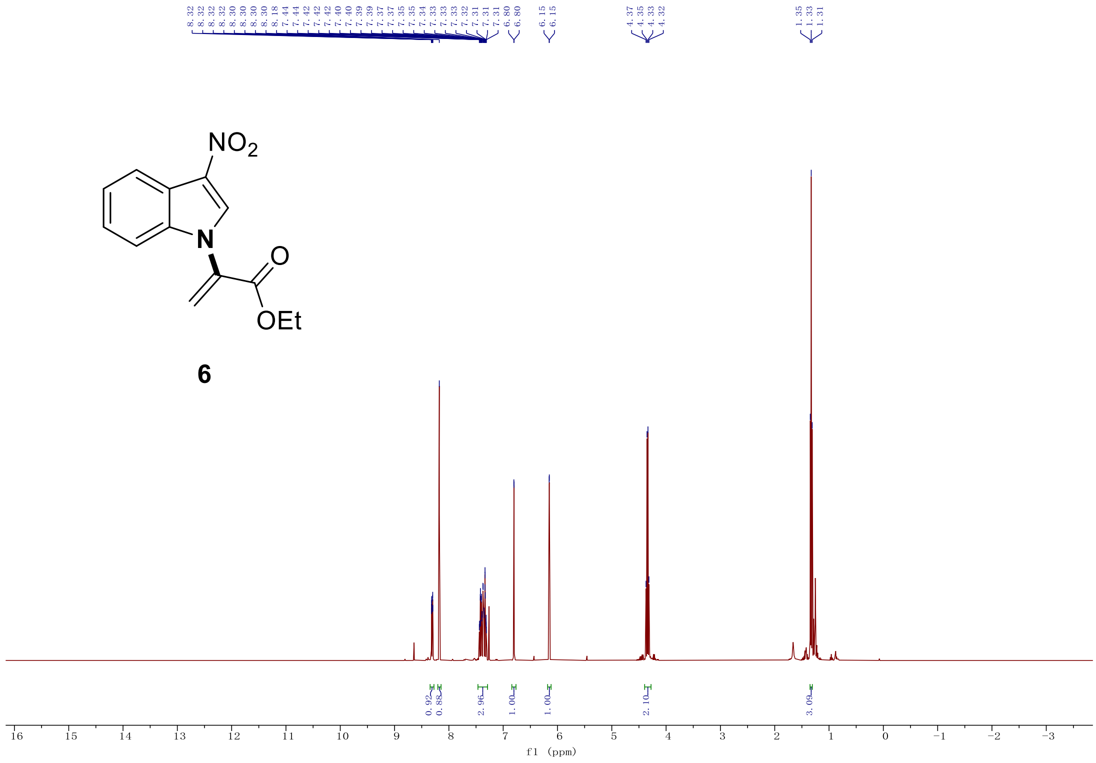

^{1}H NMR spectrum ($CDCl_3$, 400 MHz) of (**6**)

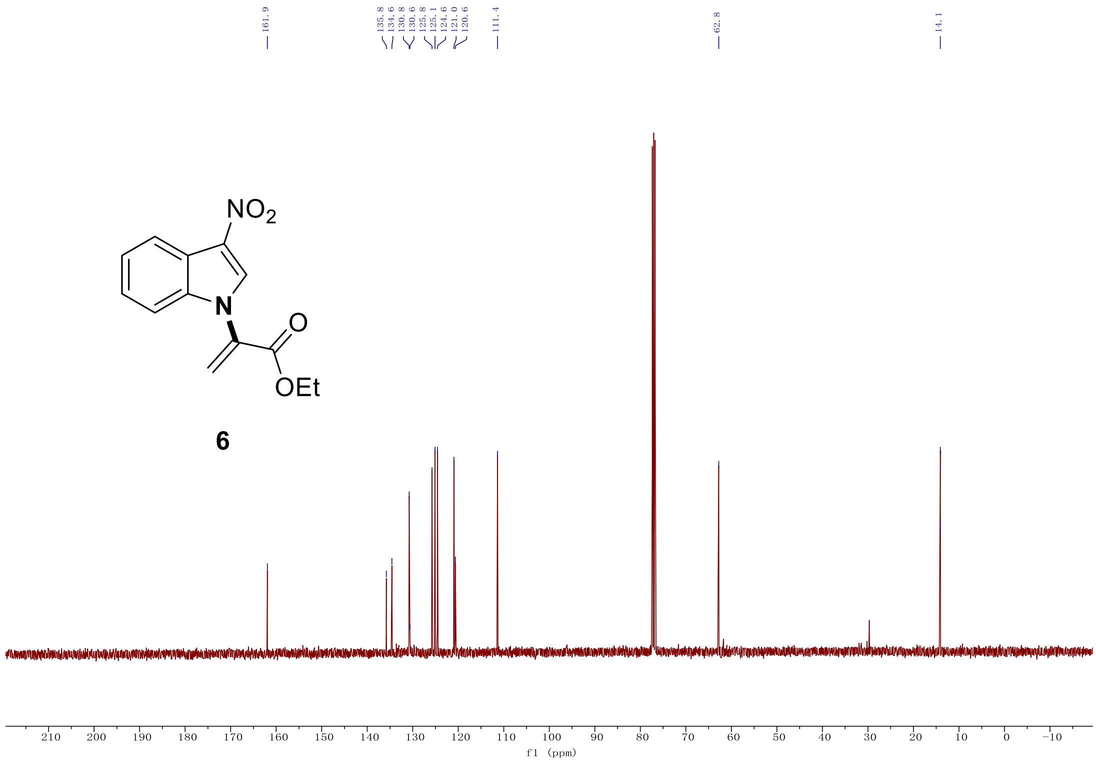

^{13}C NMR spectrum ($CDCl_3$, 101 MHz) of (**6**)

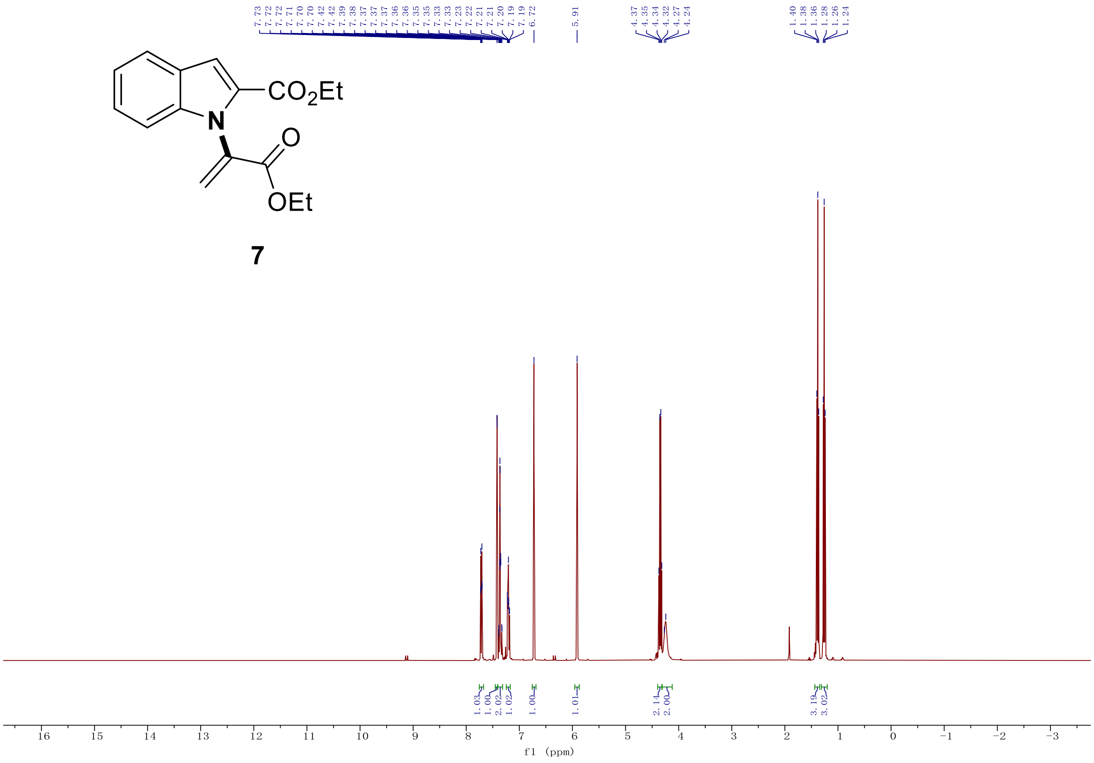

^{1}H NMR spectrum ($CDCl_3$, 400 MHz) of (**7**)

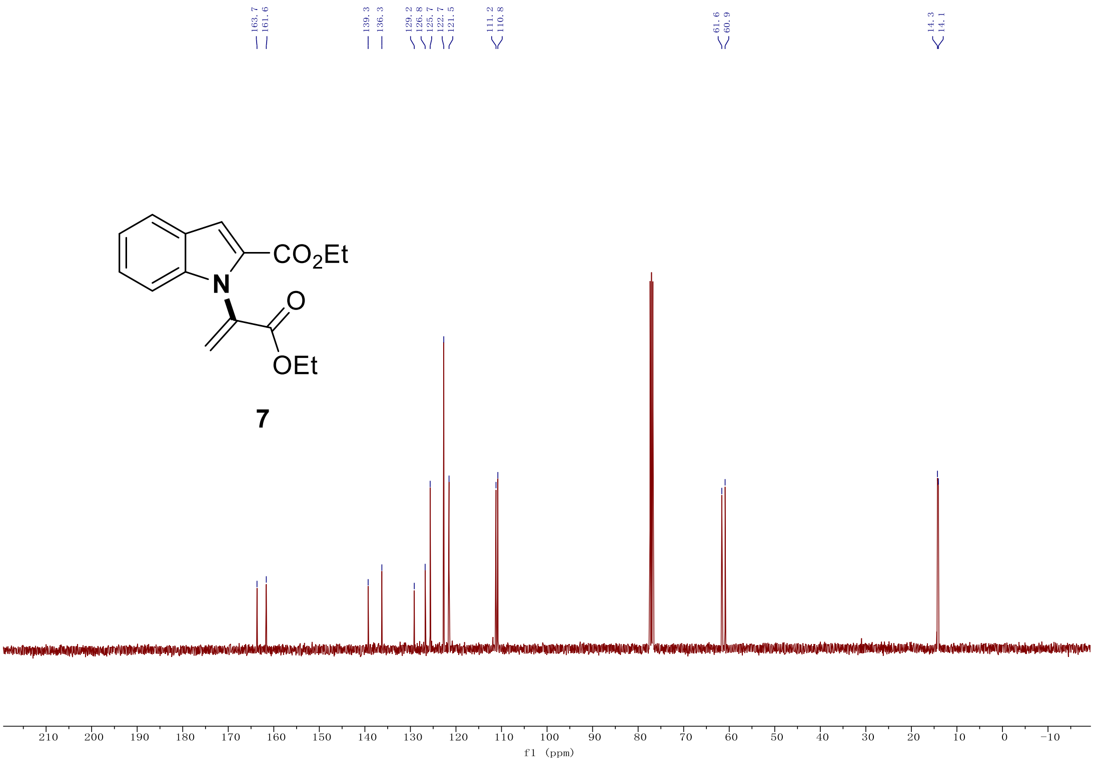

^{13}C NMR spectrum ($CDCl_3$, 101 MHz) of (**7**)

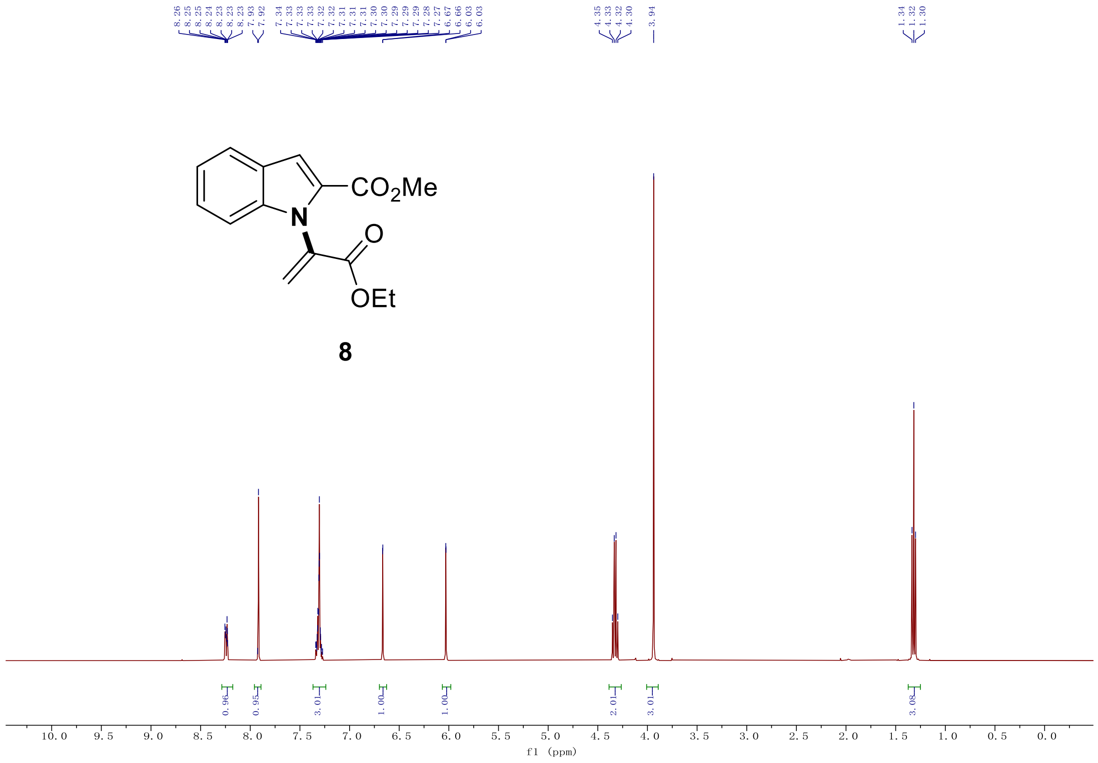

^{1}H NMR spectrum (CDCl$_3$, 500 MHz) of (**8**)

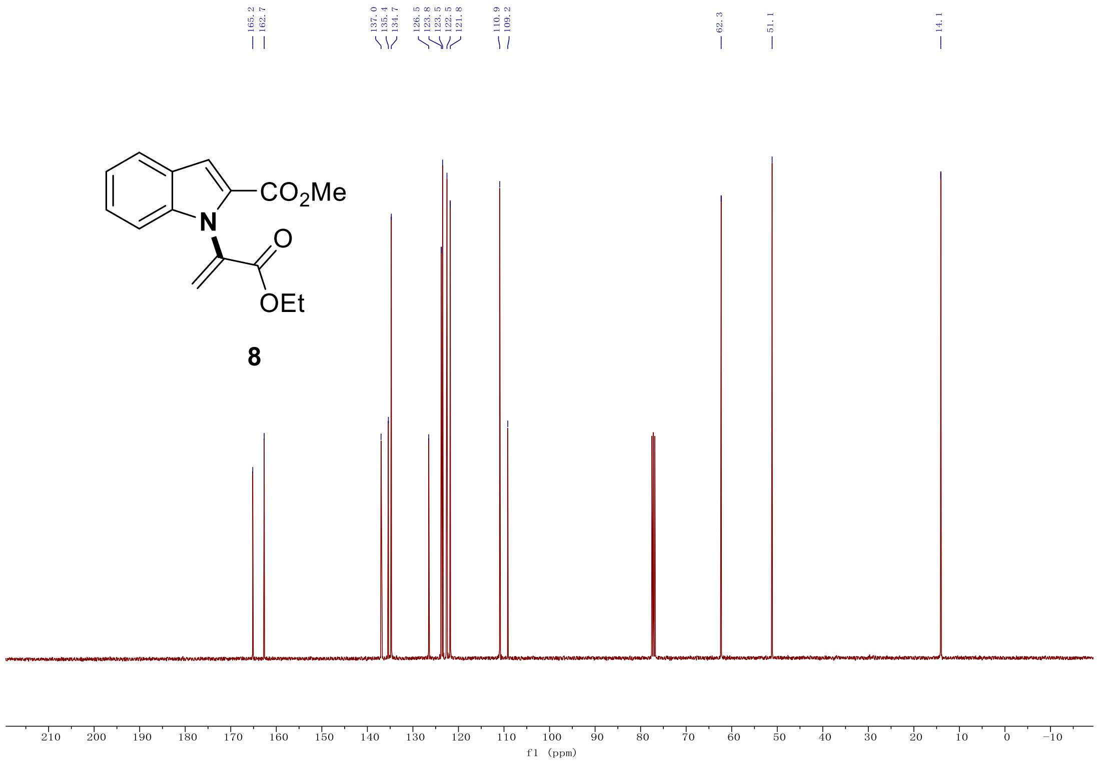

^{13}C NMR spectrum (CDCl$_3$, 126 MHz) of (**8**)

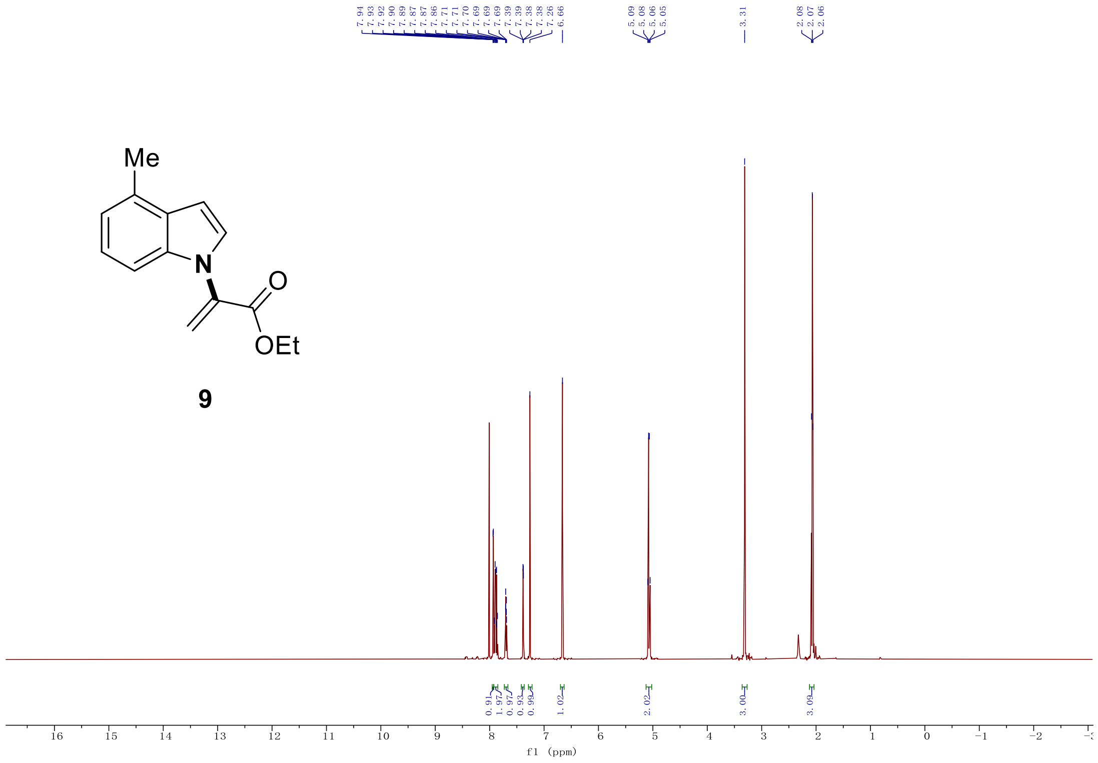

^{1}H NMR spectrum (CDCl$_3$, 500 MHz) of (**9**)

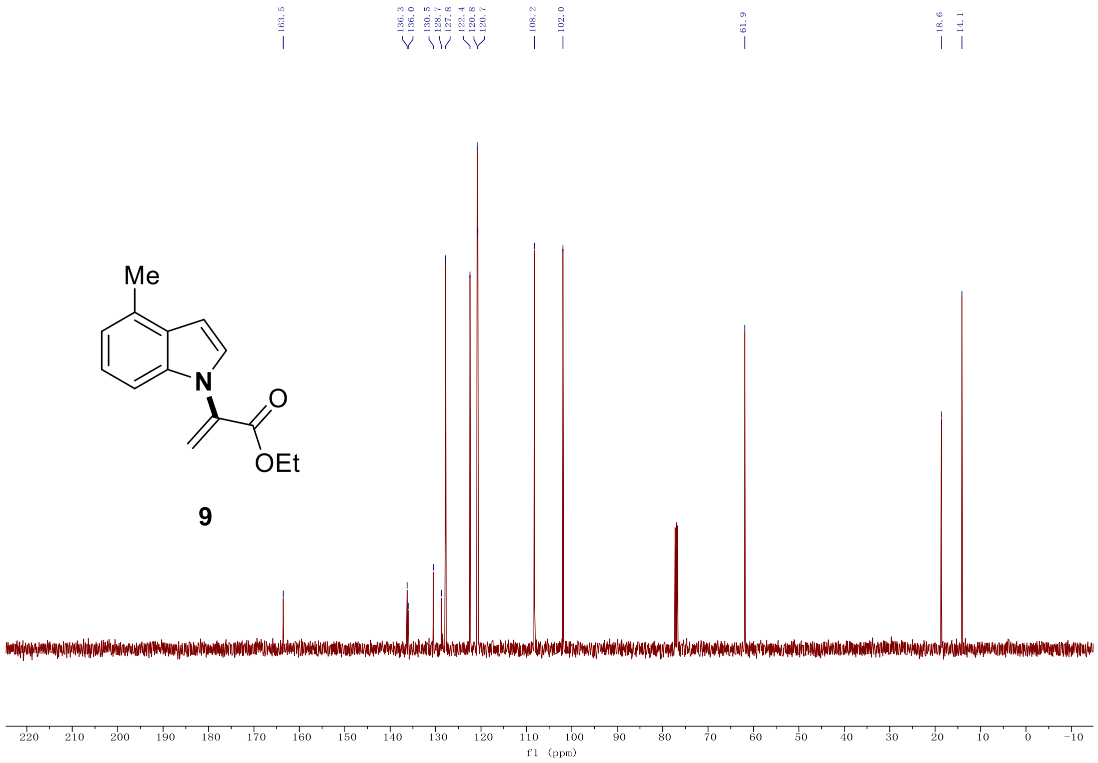

^{13}C NMR spectrum (CDCl$_3$, 126 MHz) of (**9**)

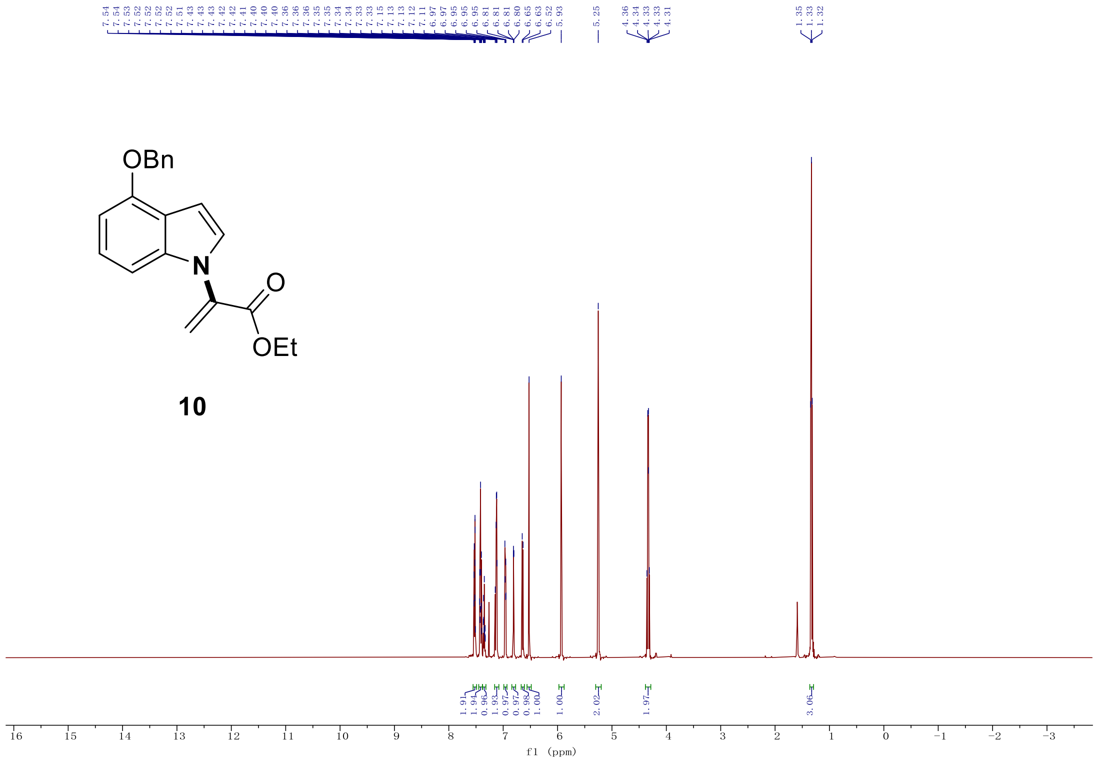

^{1}H NMR spectrum ($CDCl_3$, 500 MHz) of (**10**)

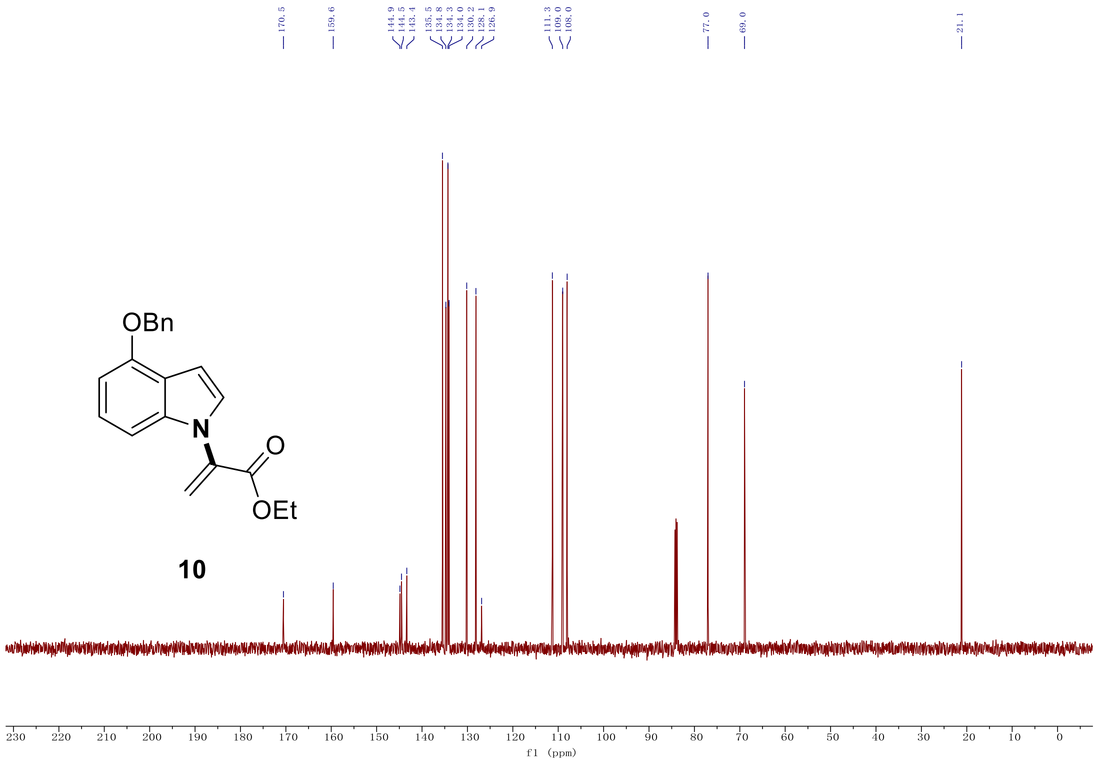

^{13}C NMR spectrum ($CDCl_3$, 126 MHz) of (**10**)

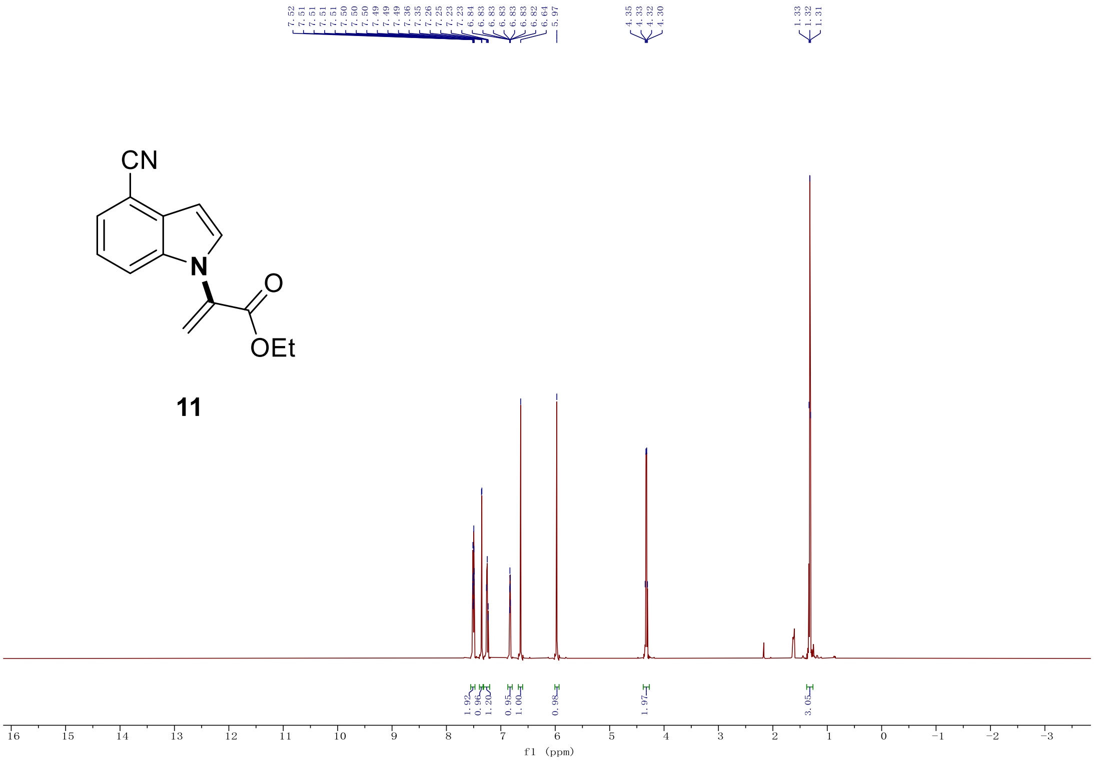

^{1}H NMR spectrum ($CDCl_3$, 500 MHz) of (**11**)

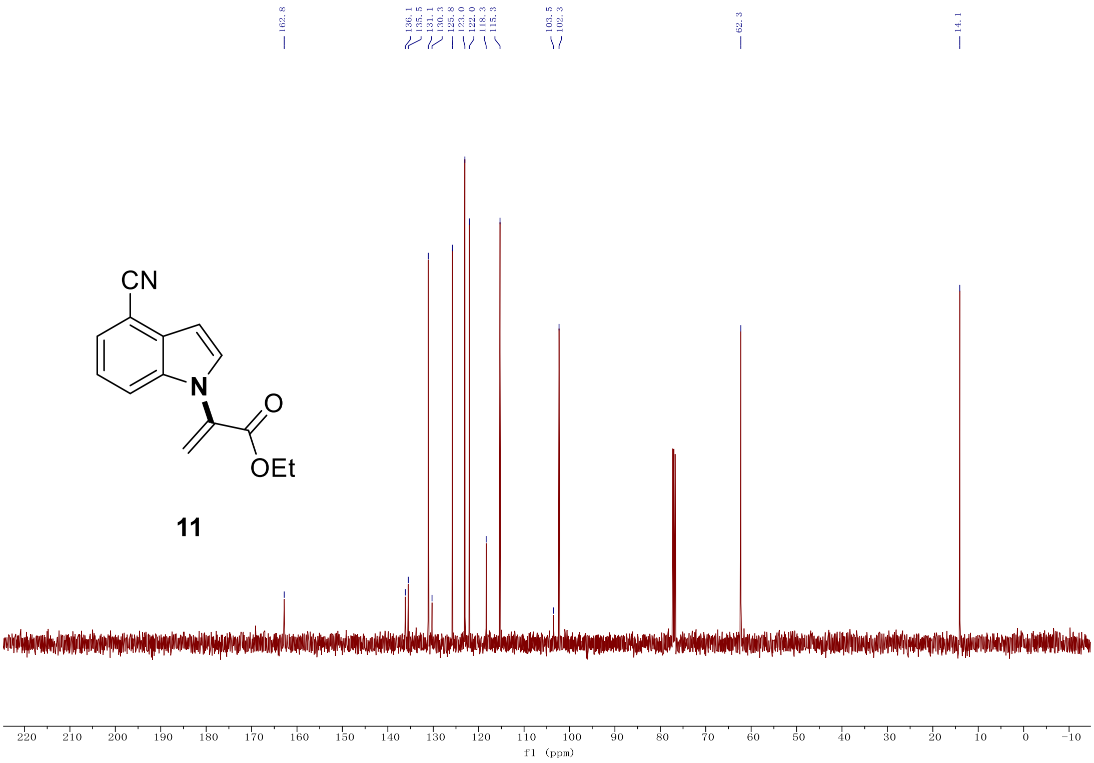

^{13}C NMR spectrum ($CDCl_3$, 126 MHz) of (**11**)

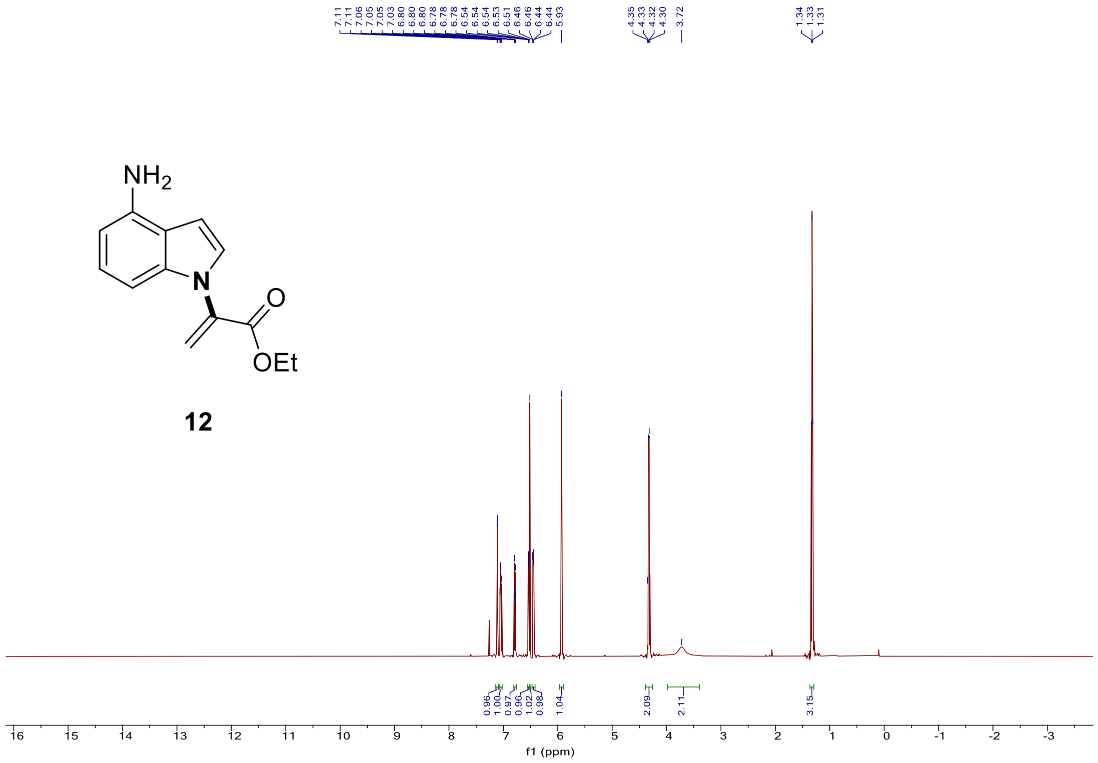

^{1}H NMR spectrum ($CDCl_3$, 500 MHz) of (**12**)

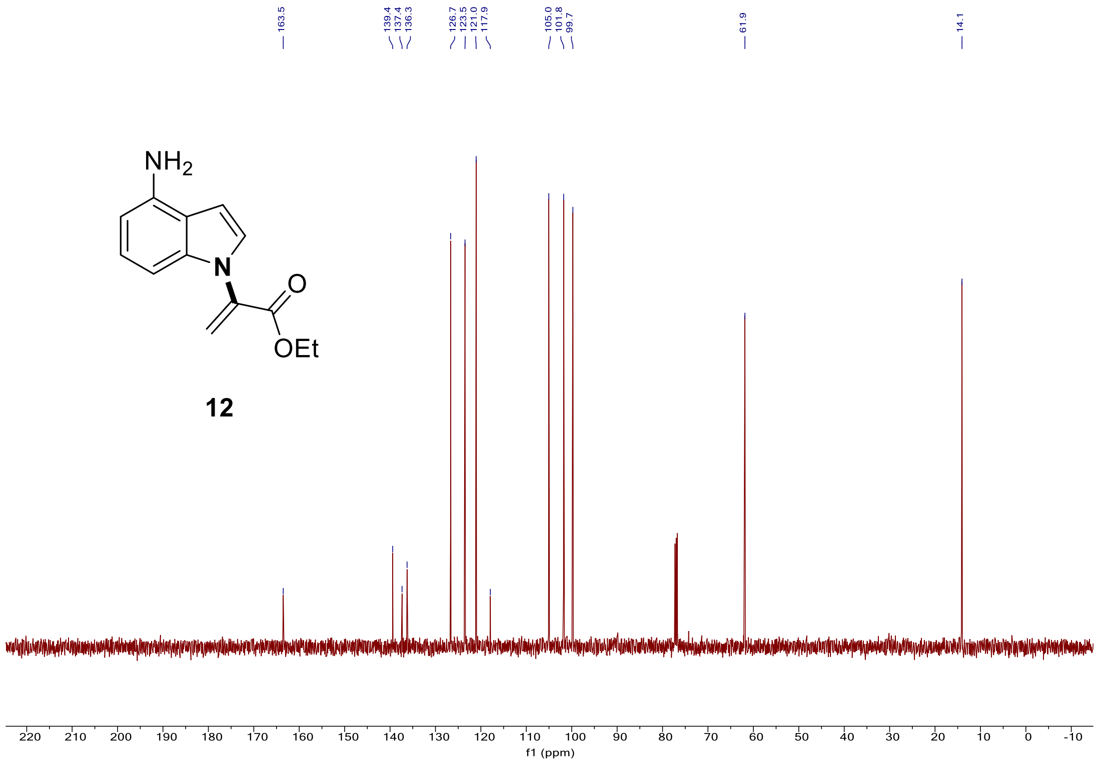

^{13}C NMR spectrum ($CDCl_3$, 126 MHz) of (**12**)

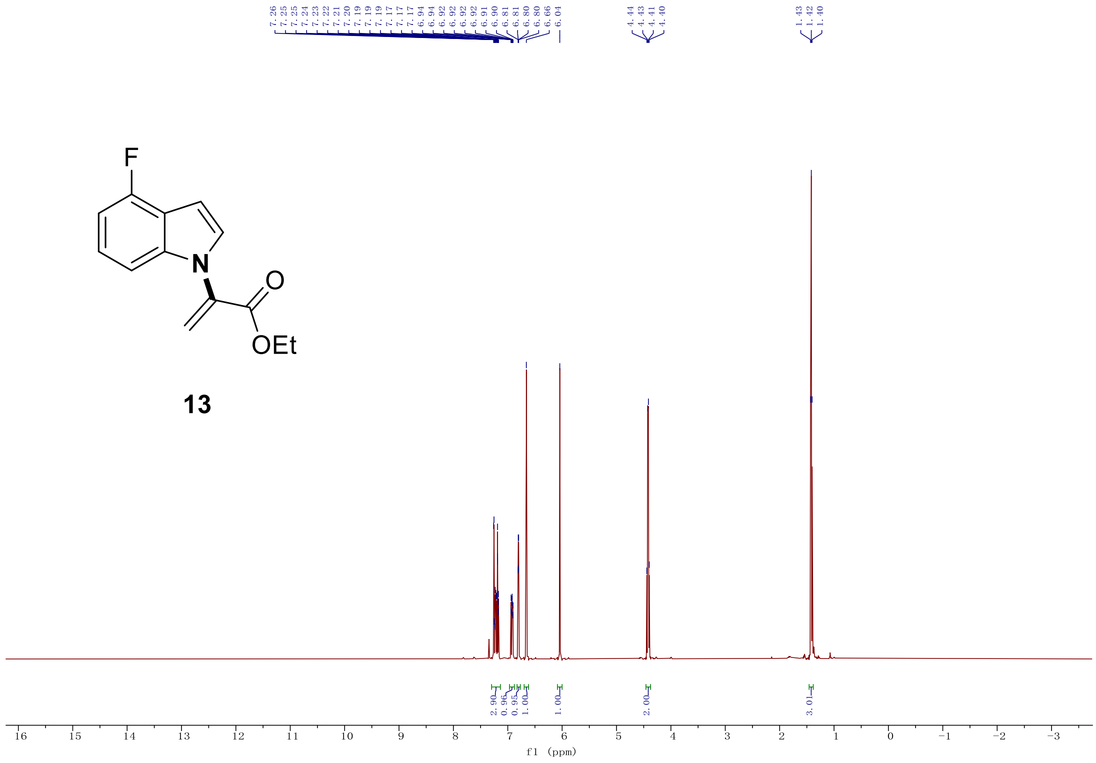

^{1}H NMR spectrum ($CDCl_3$, 500 MHz) of (**13**)

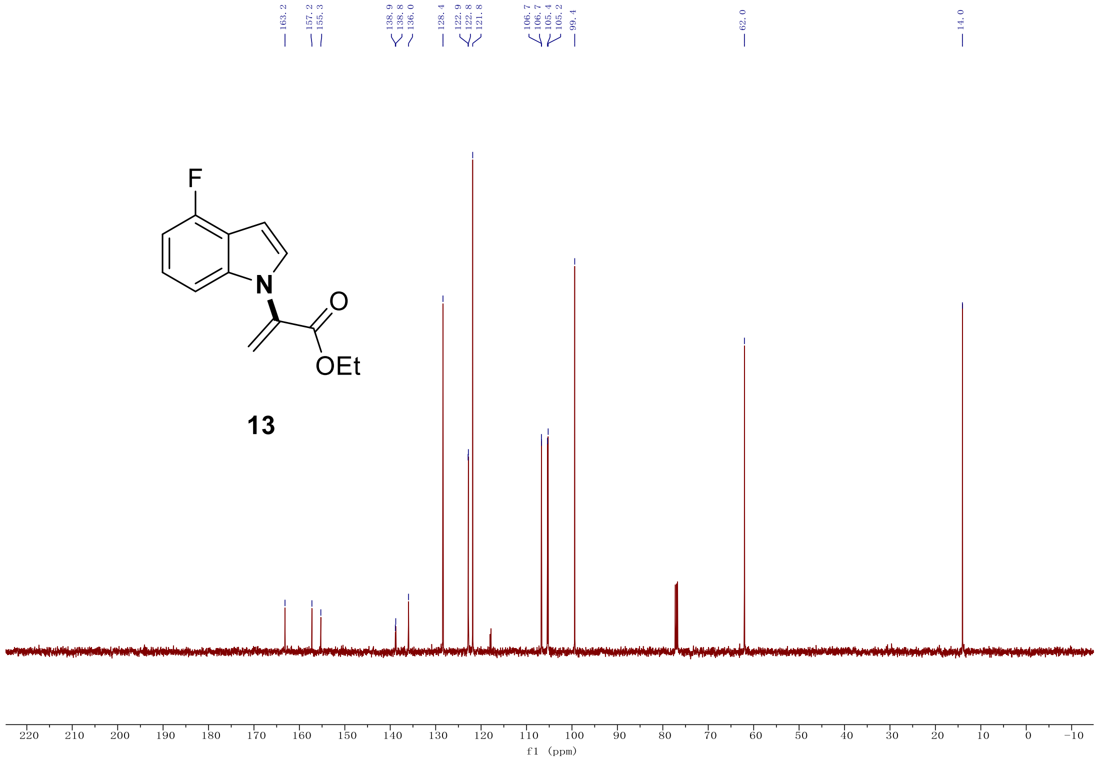

^{13}C NMR spectrum ($CDCl_3$, 126 MHz) of (**13**)

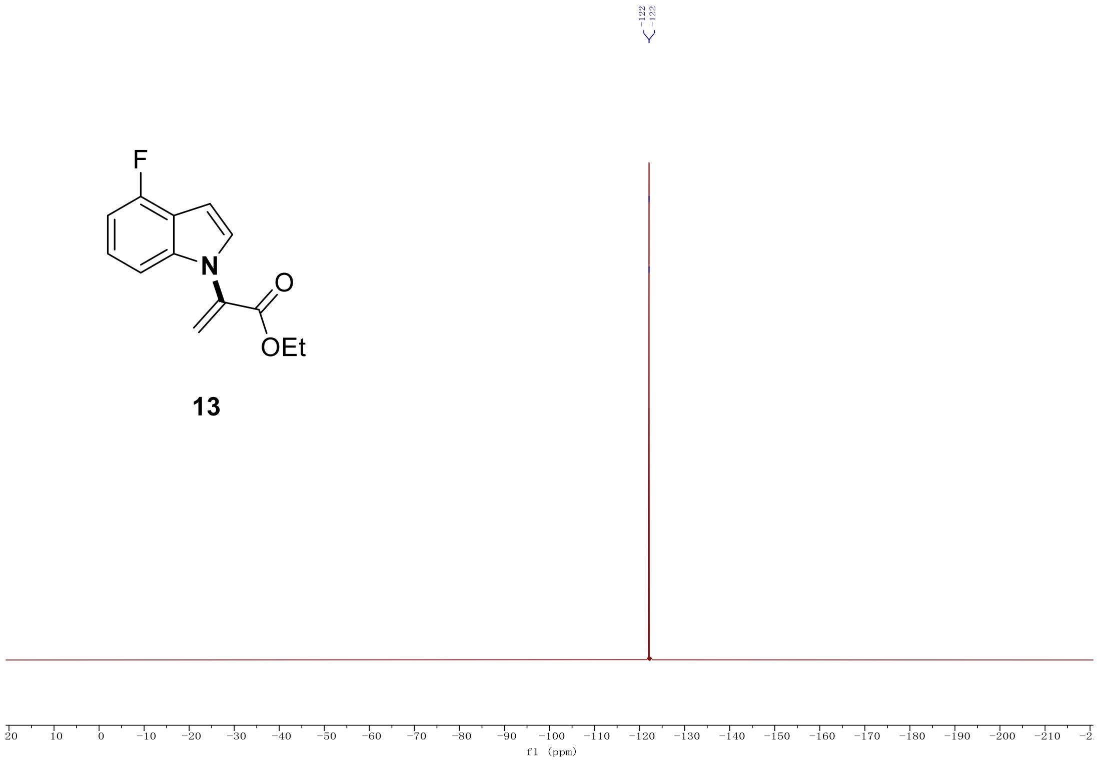

^{19}F NMR spectrum ($CDCl_3$, 471 MHz) of (**13**)

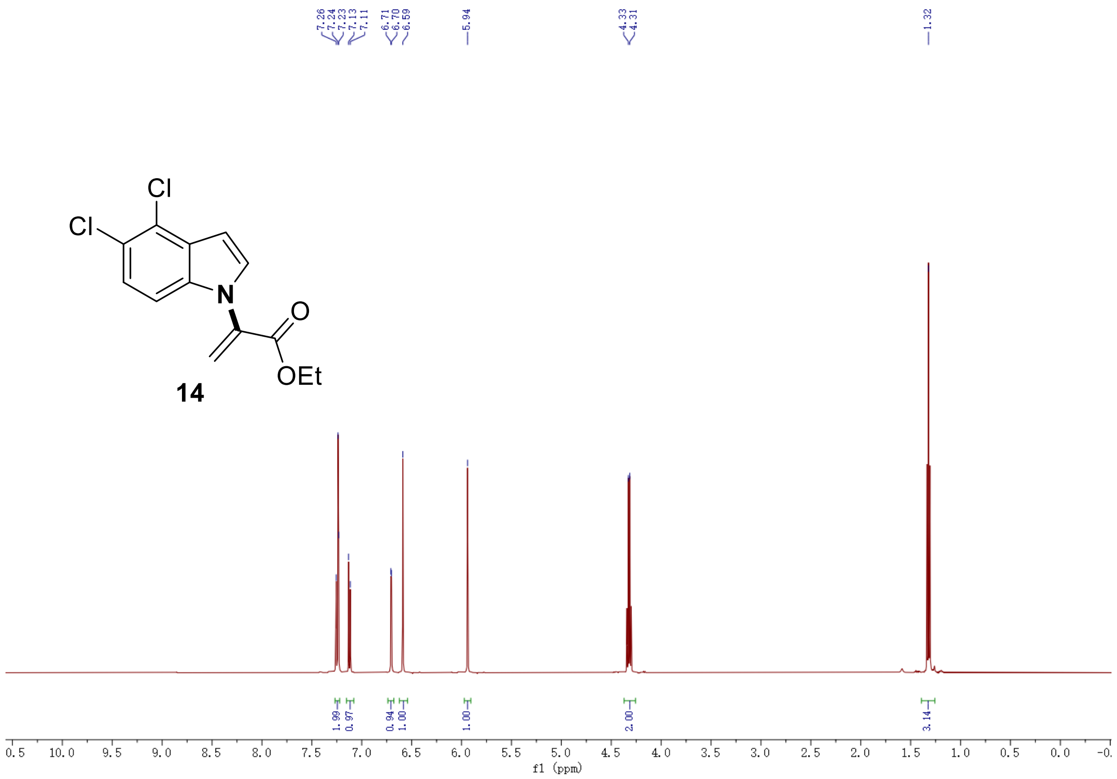

^{1}H NMR spectrum (CDCl$_3$, 500 MHz) of (**14**)

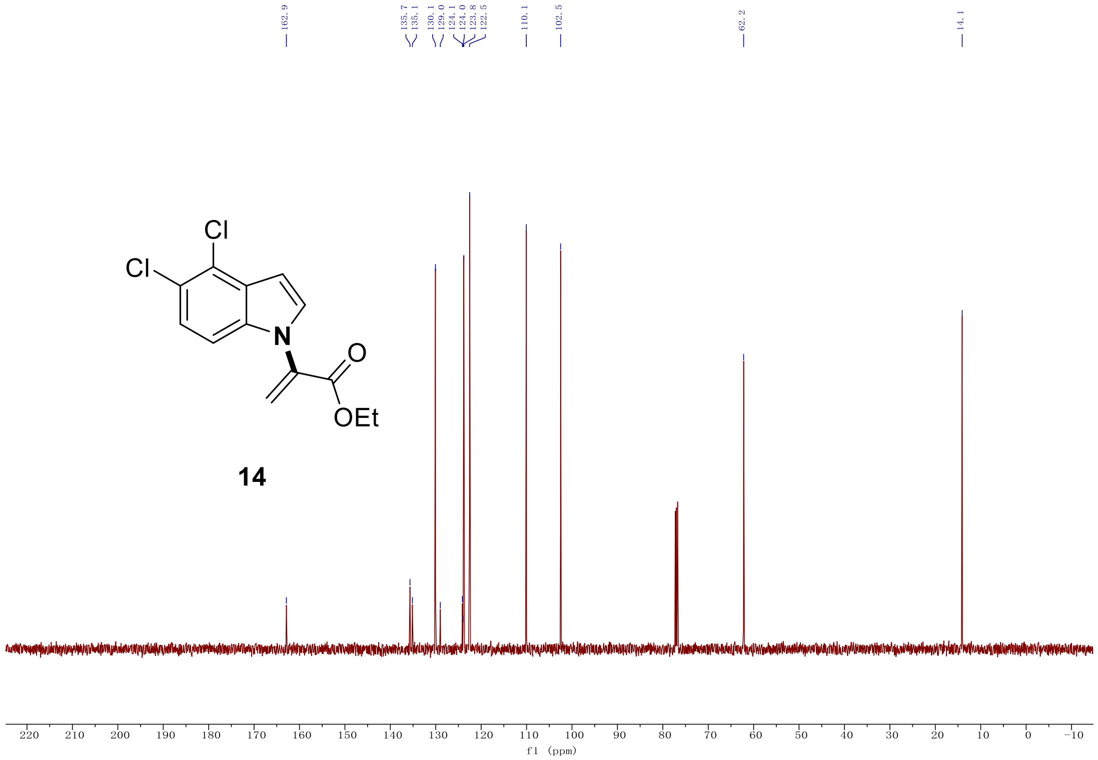

^{13}C NMR spectrum (CDCl$_3$, 126 MHz) of (**14**)

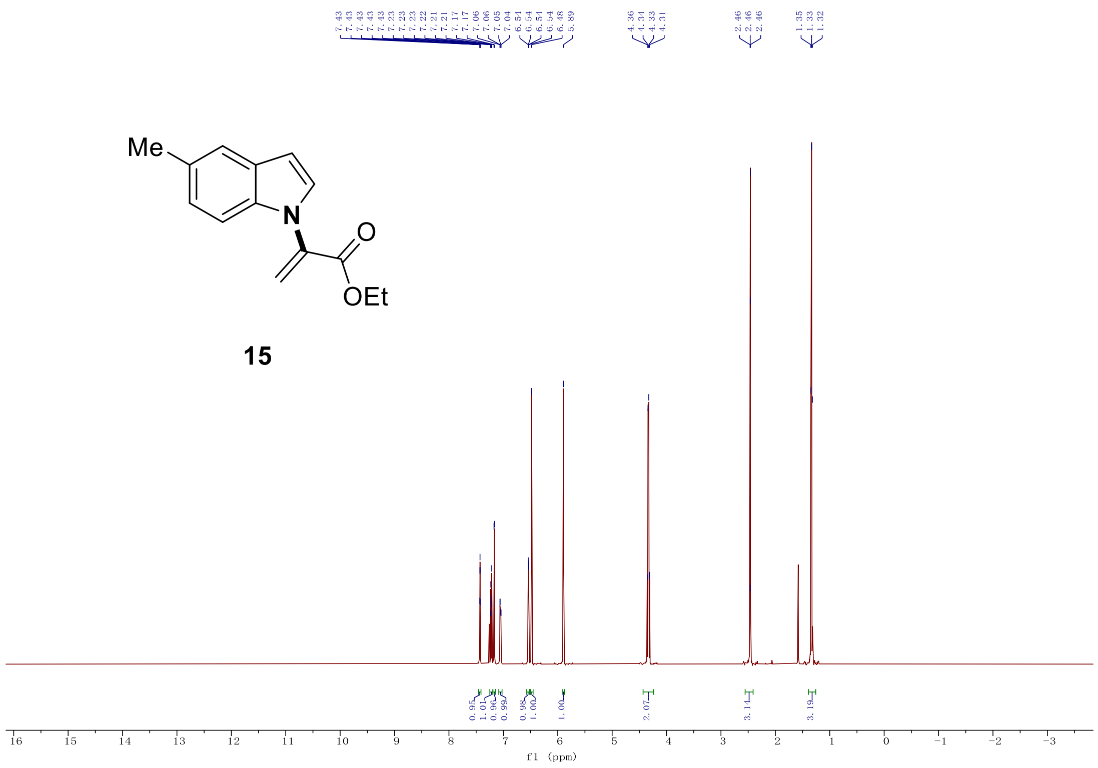

^{1}H NMR spectrum ($CDCl_3$, 500 MHz) of (**15**)

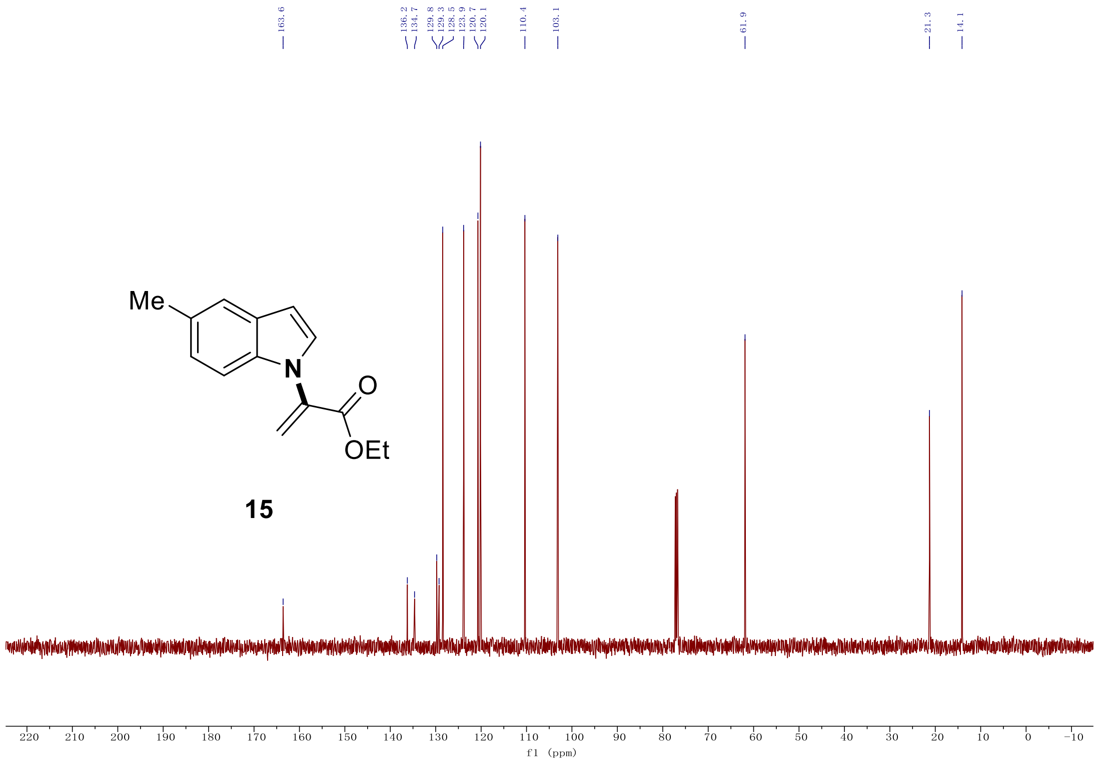

^{13}C NMR spectrum ($CDCl_3$, 126 MHz) of (**15**)

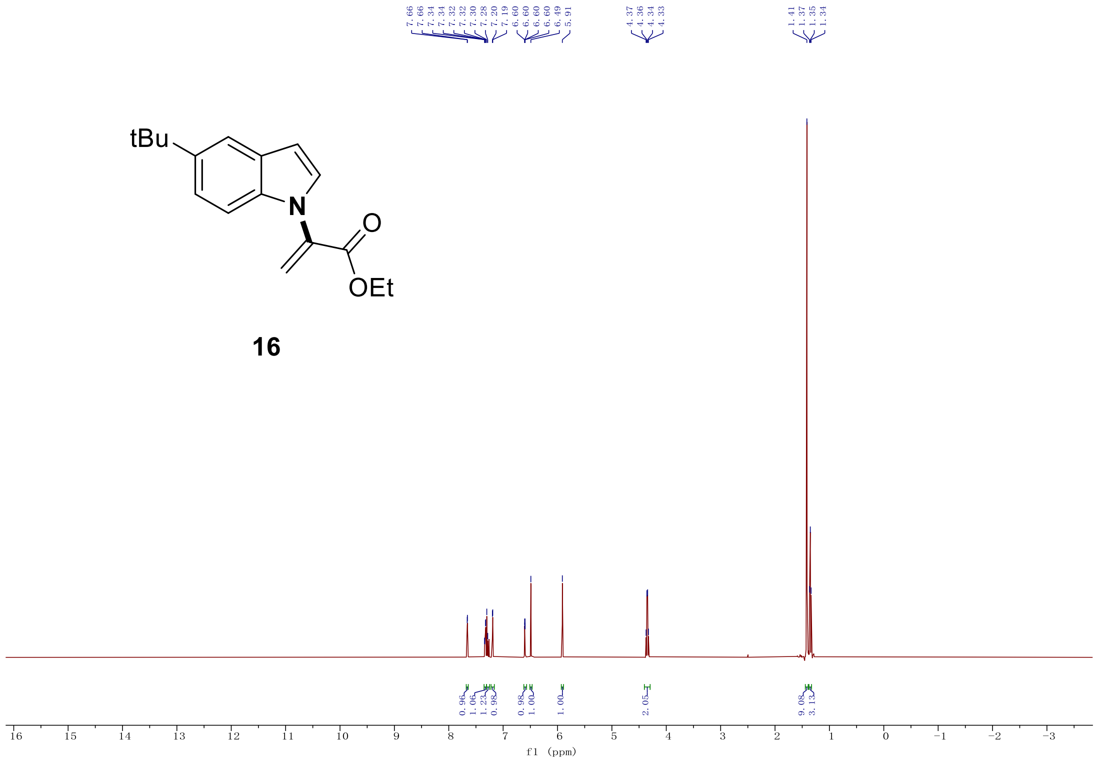

^{1}H NMR spectrum ($CDCl_3$, 500 MHz) of (**16**)

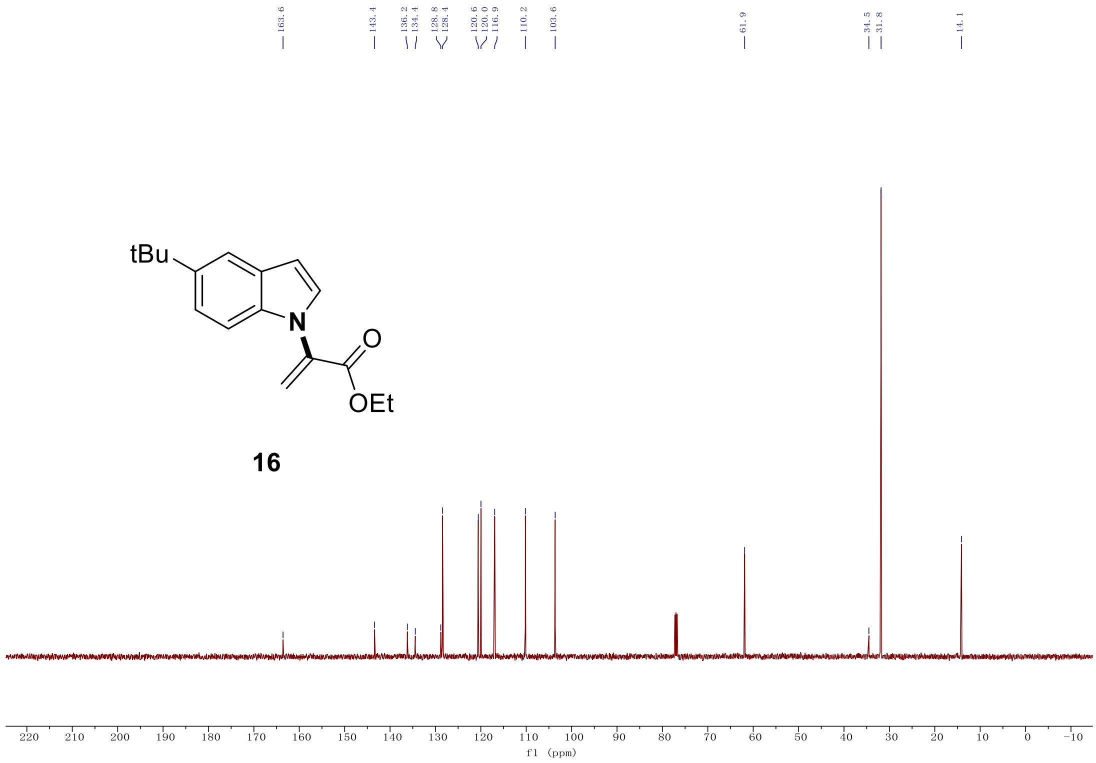

^{13}C NMR spectrum ($CDCl_3$, 126 MHz) of (**16**)

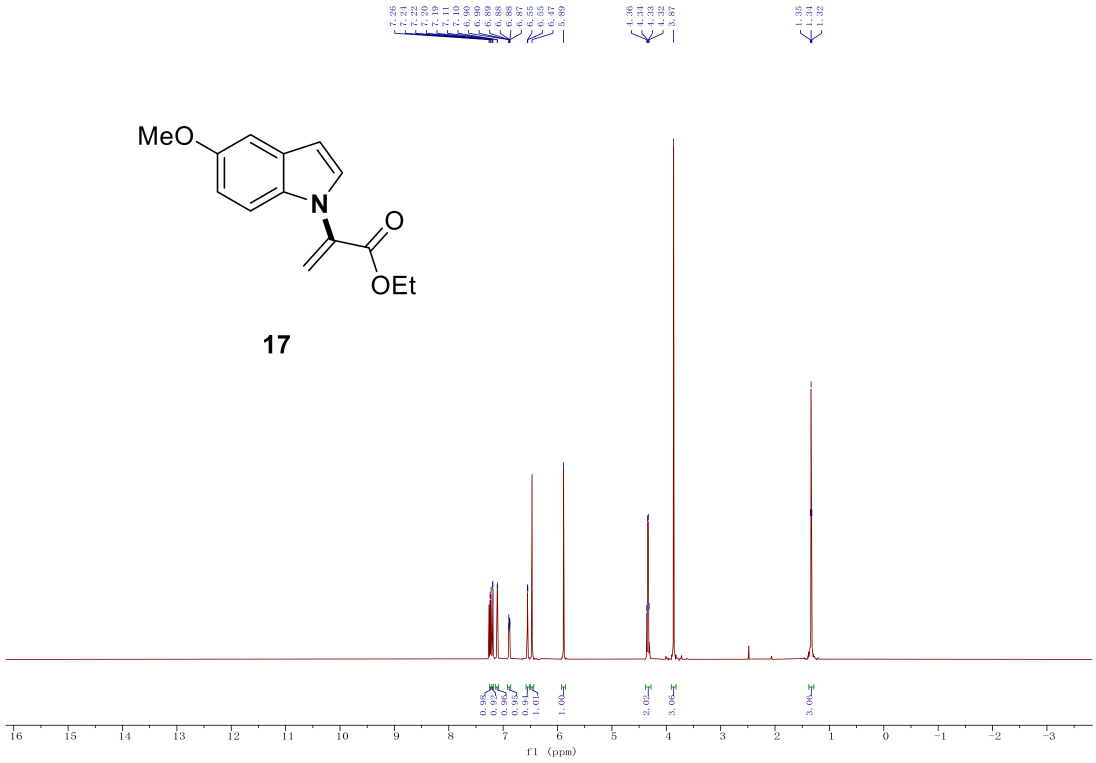

^{1}H NMR spectrum (CDCl$_3$, 500 MHz) of (**17**)

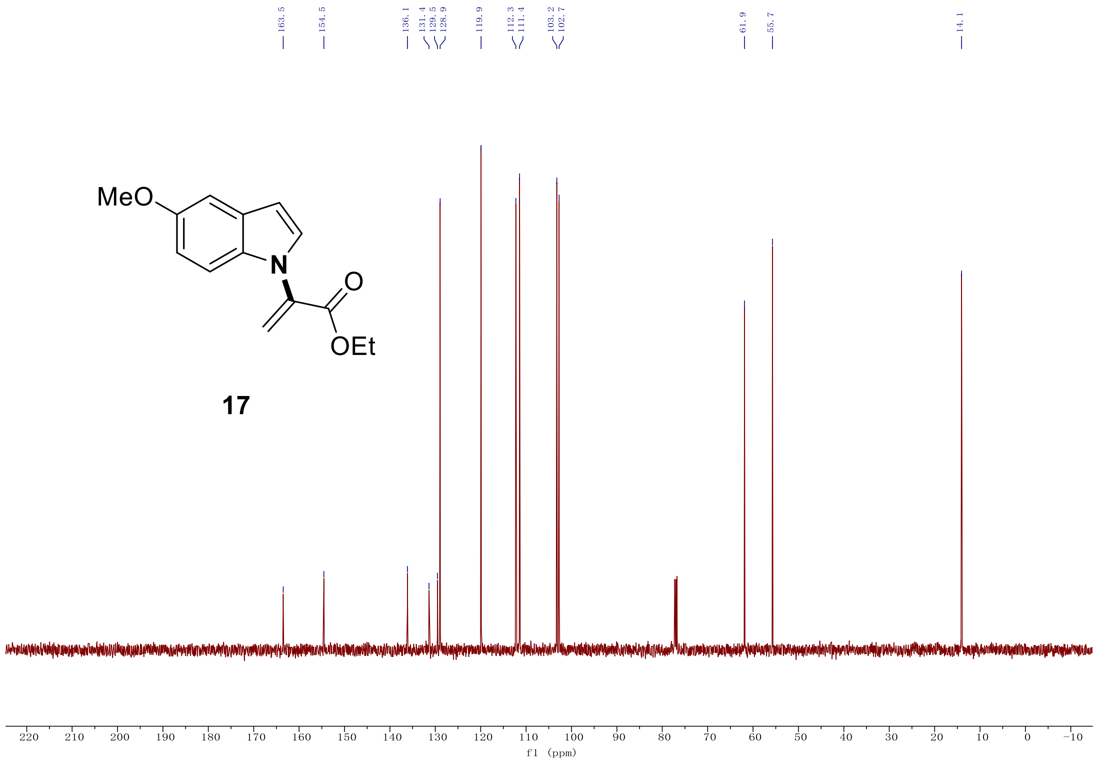

^{13}C NMR spectrum (CDCl$_3$, 126 MHz) of (**17**)

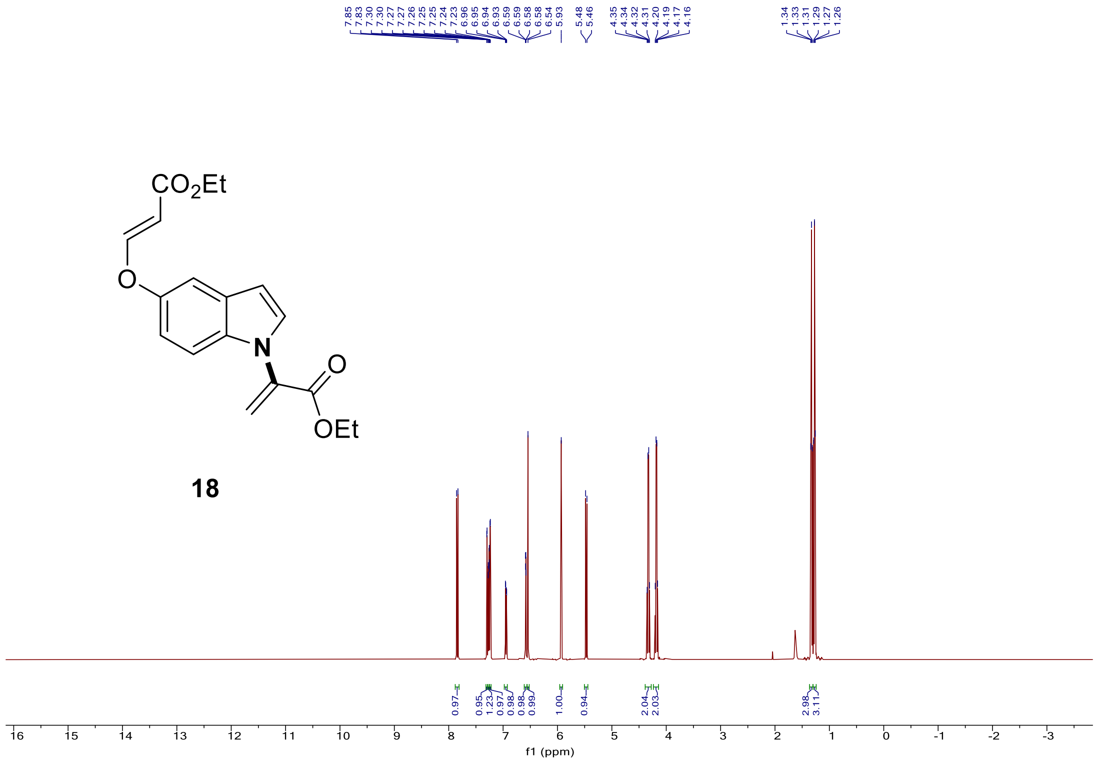

^{1}H NMR spectrum ($CDCl_3$, 500 MHz) of (**18**)

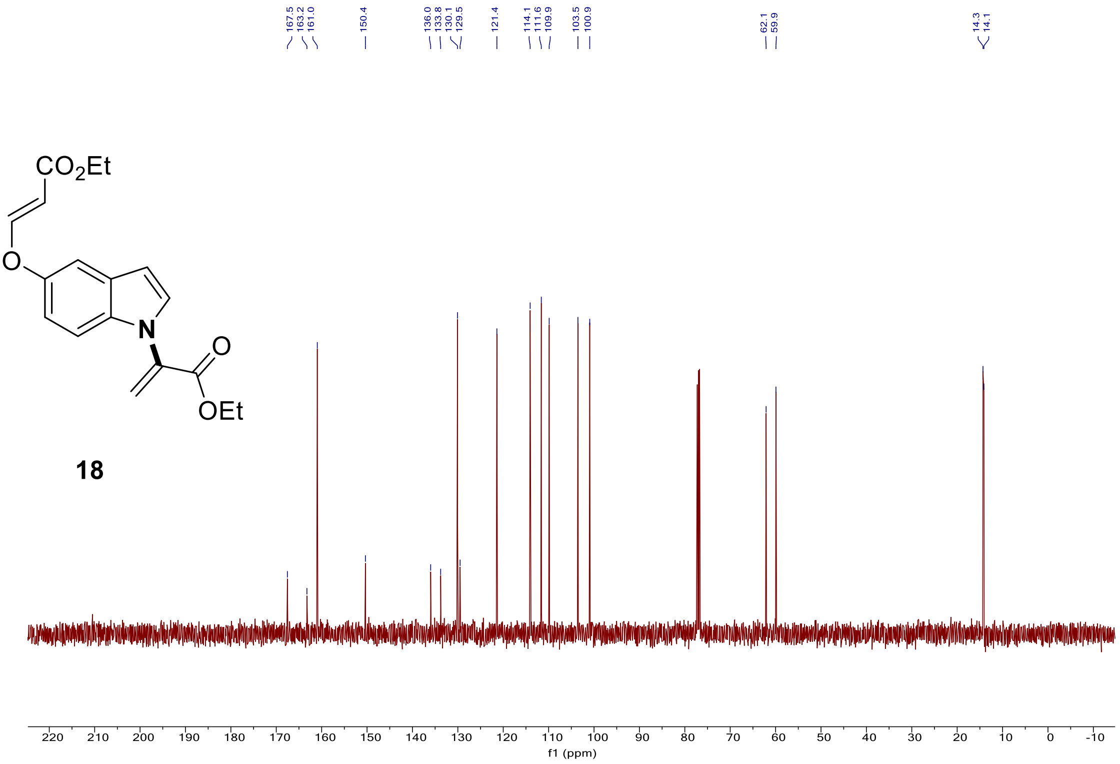

^{13}C NMR spectrum ($CDCl_3$, 126 MHz) of (**18**)

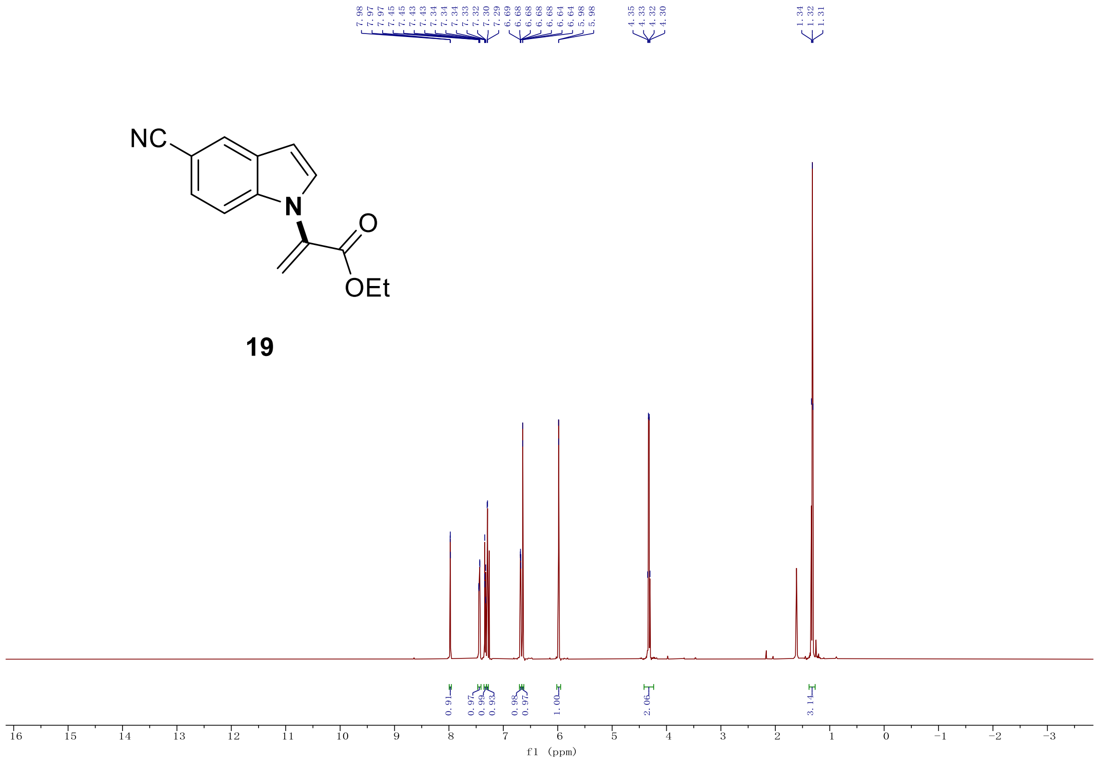

^{1}H NMR spectrum ($CDCl_3$, 500 MHz) of (**19**)

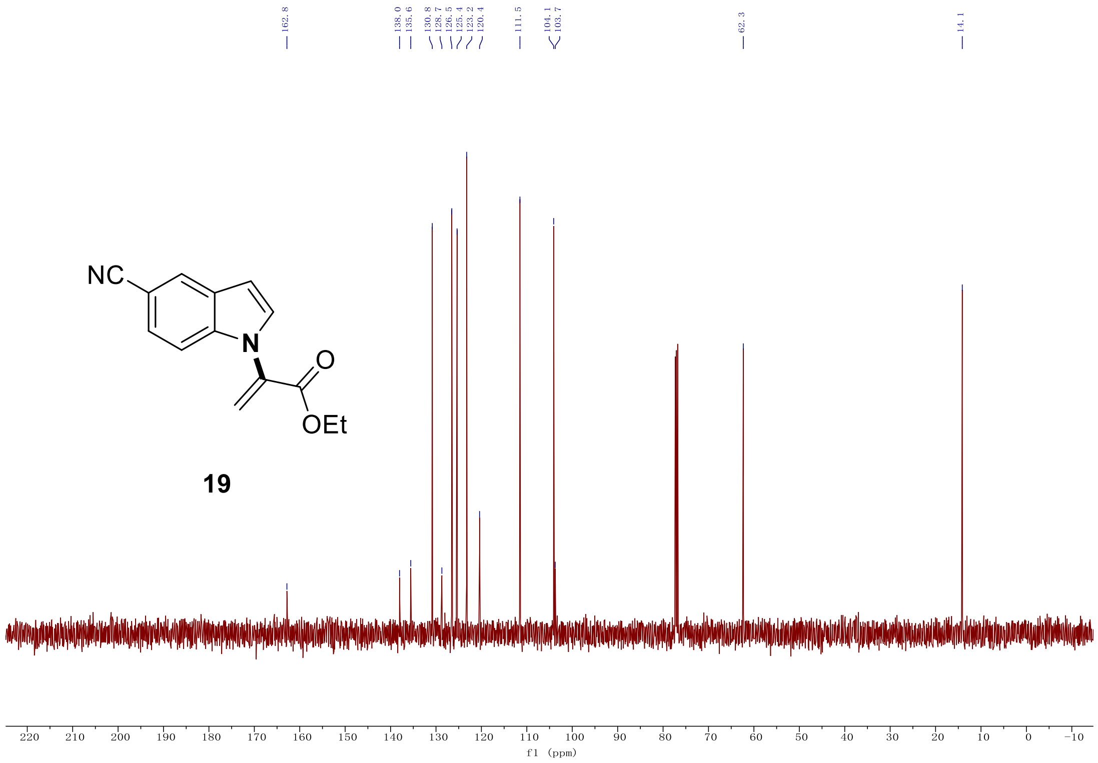

^{13}C NMR spectrum ($CDCl_3$, 126 MHz) of (**19**)

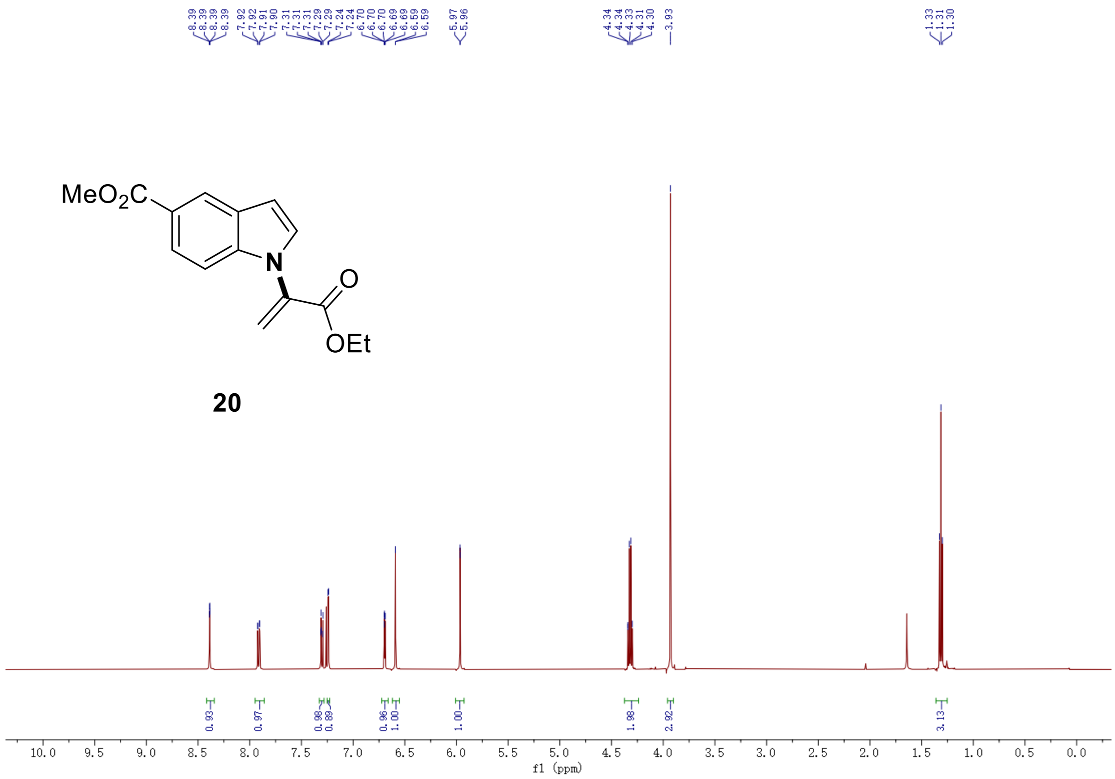

^{1}H NMR spectrum (CDCl₃, 500 MHz) of (**20**)

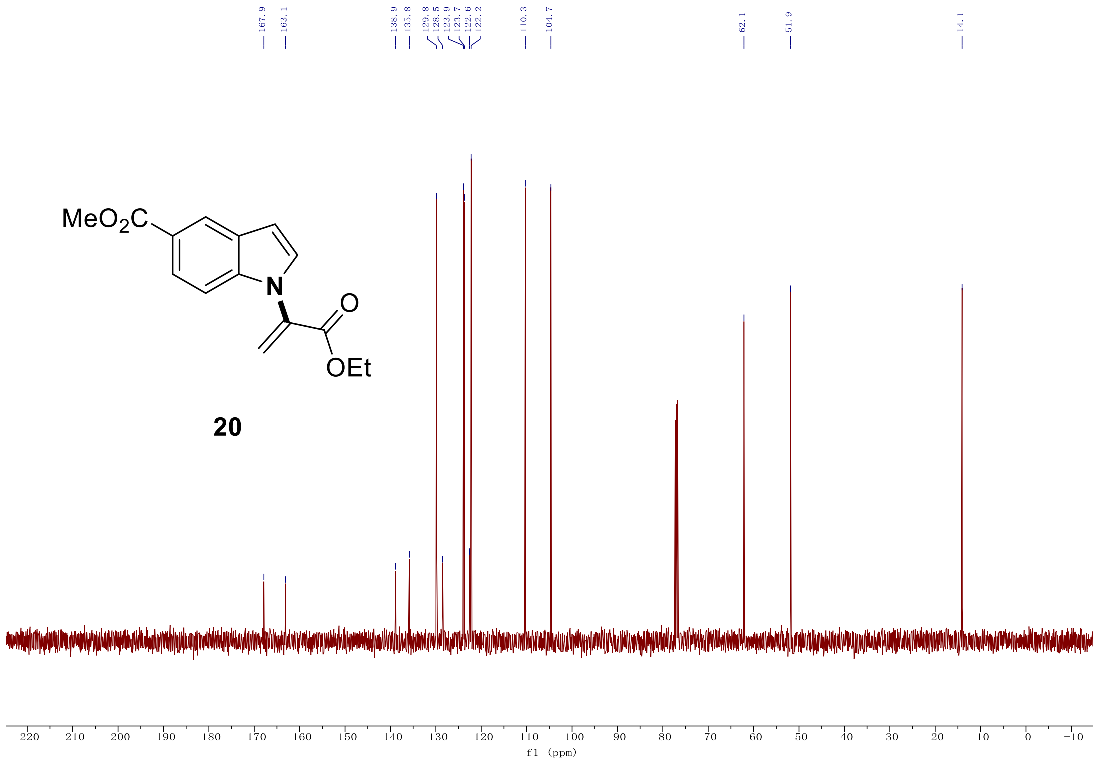

^{13}C NMR spectrum (CDCl₃, 126 MHz) of (**20**)

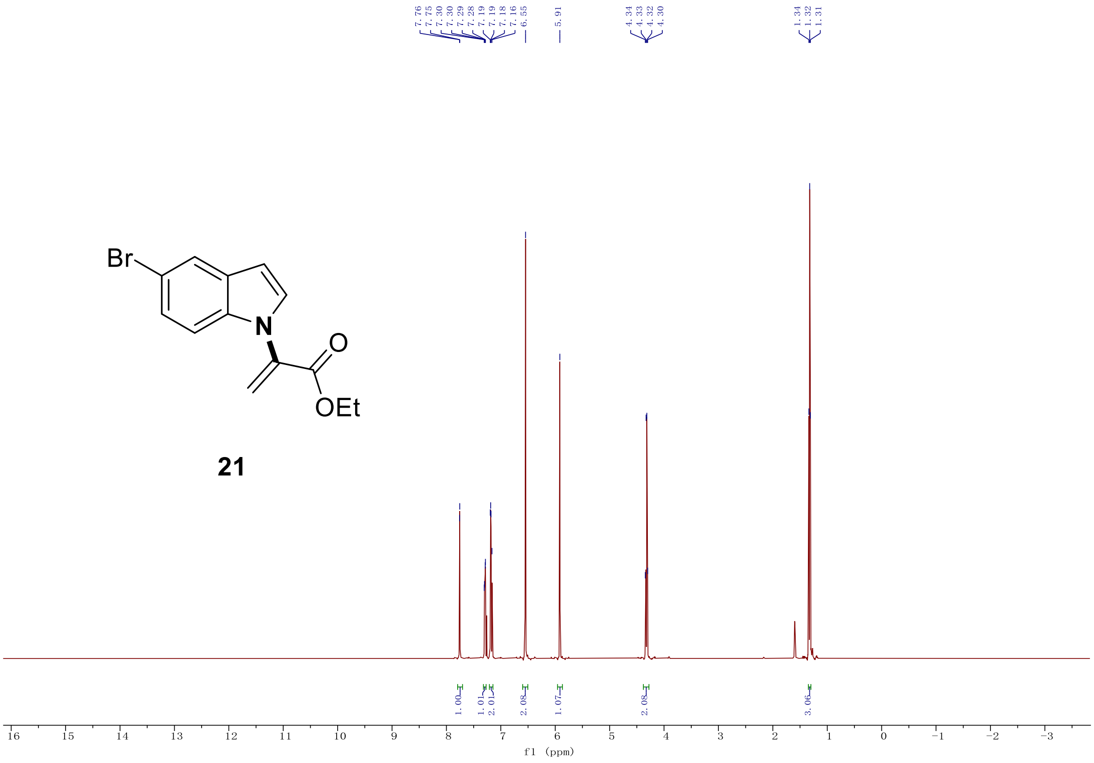

^{1}H NMR spectrum ($CDCl_3$, 500 MHz) of (**21**)

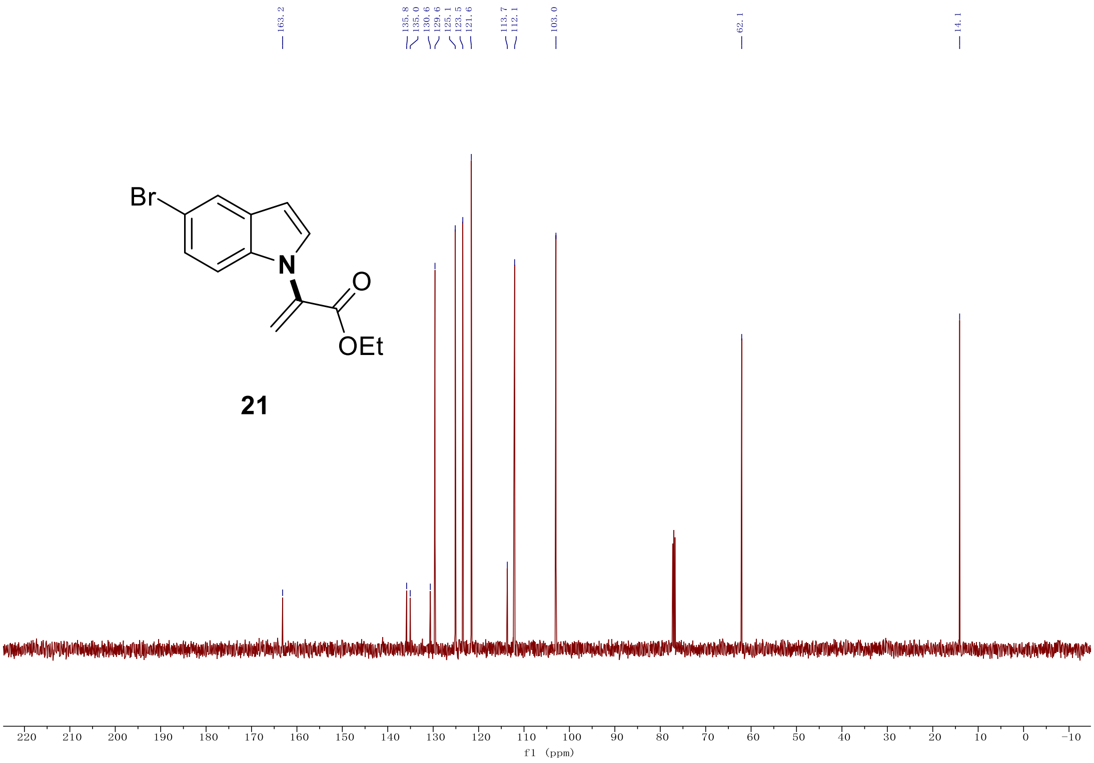

^{13}C NMR spectrum ($CDCl_3$, 126 MHz) of (**21**)

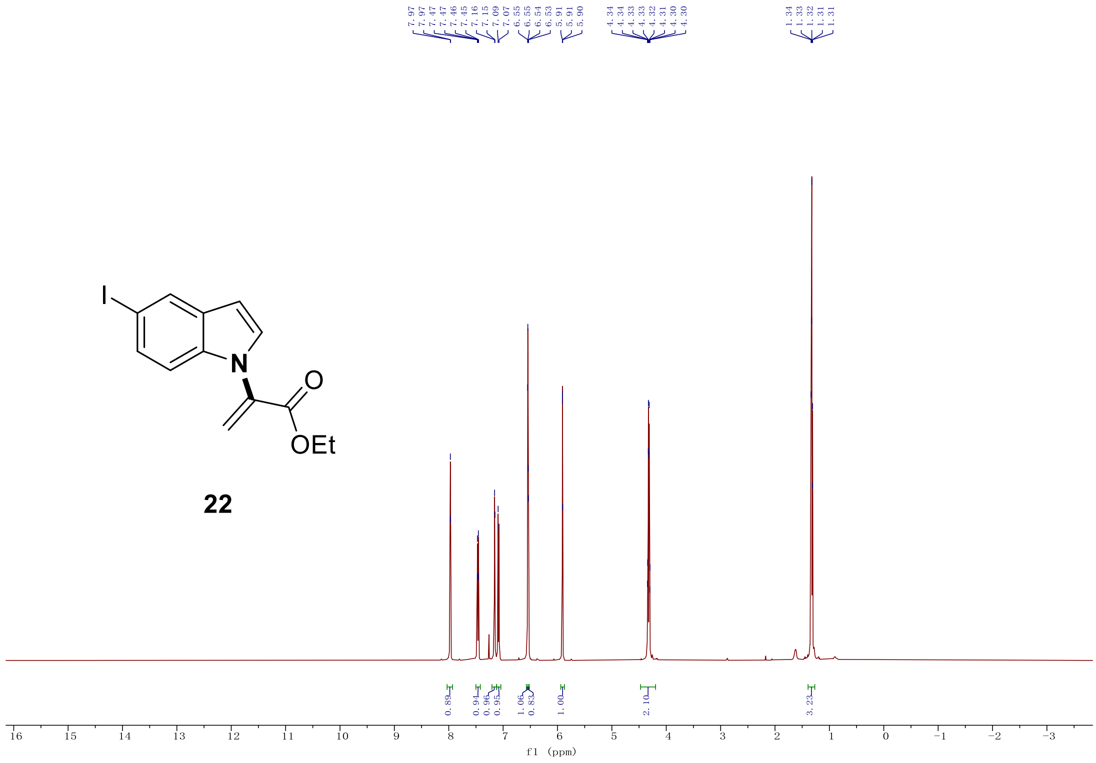

^{1}H NMR spectrum ($CDCl_3$, 500 MHz) of (**22**)

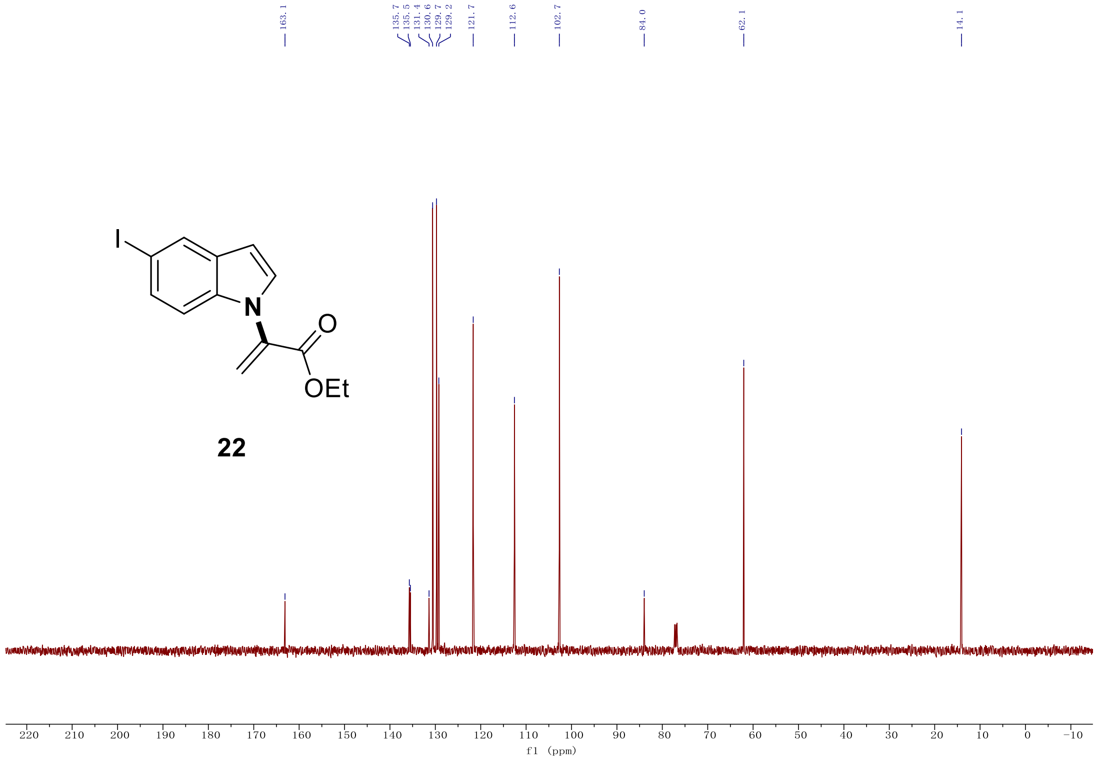

^{13}C NMR spectrum ($CDCl_3$, 126 MHz) of (**22**)

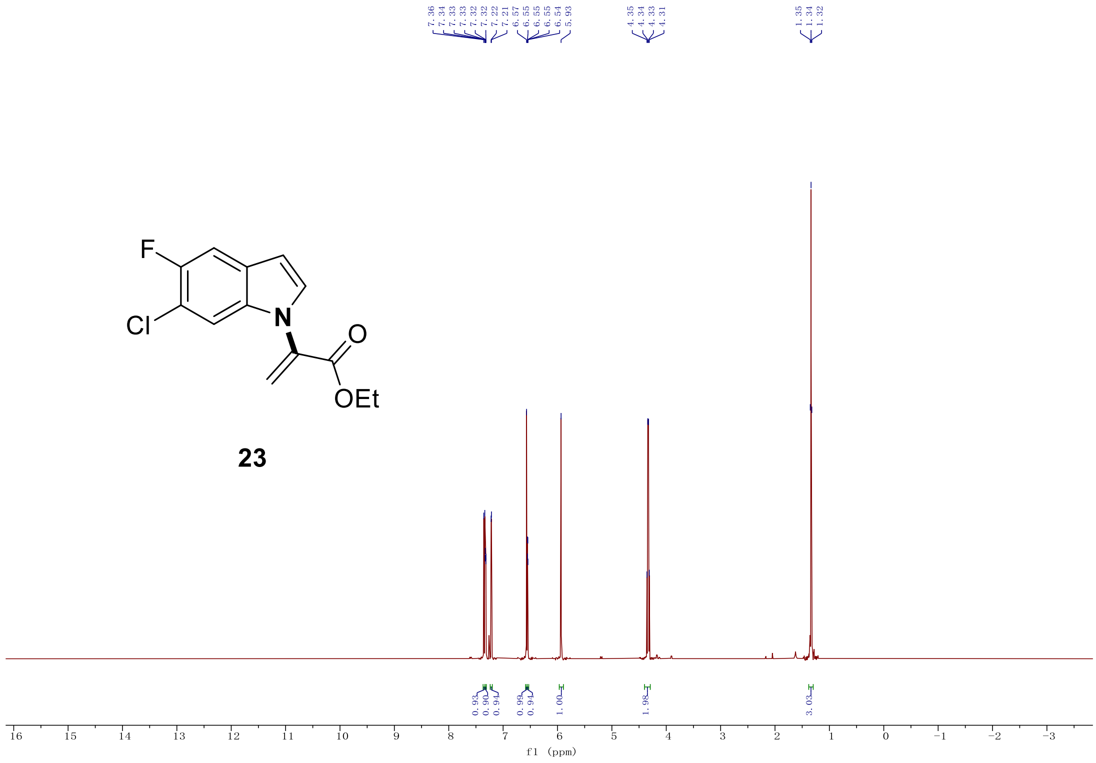

^{1}H NMR spectrum ($CDCl_3$, 500 MHz) of (**23**)

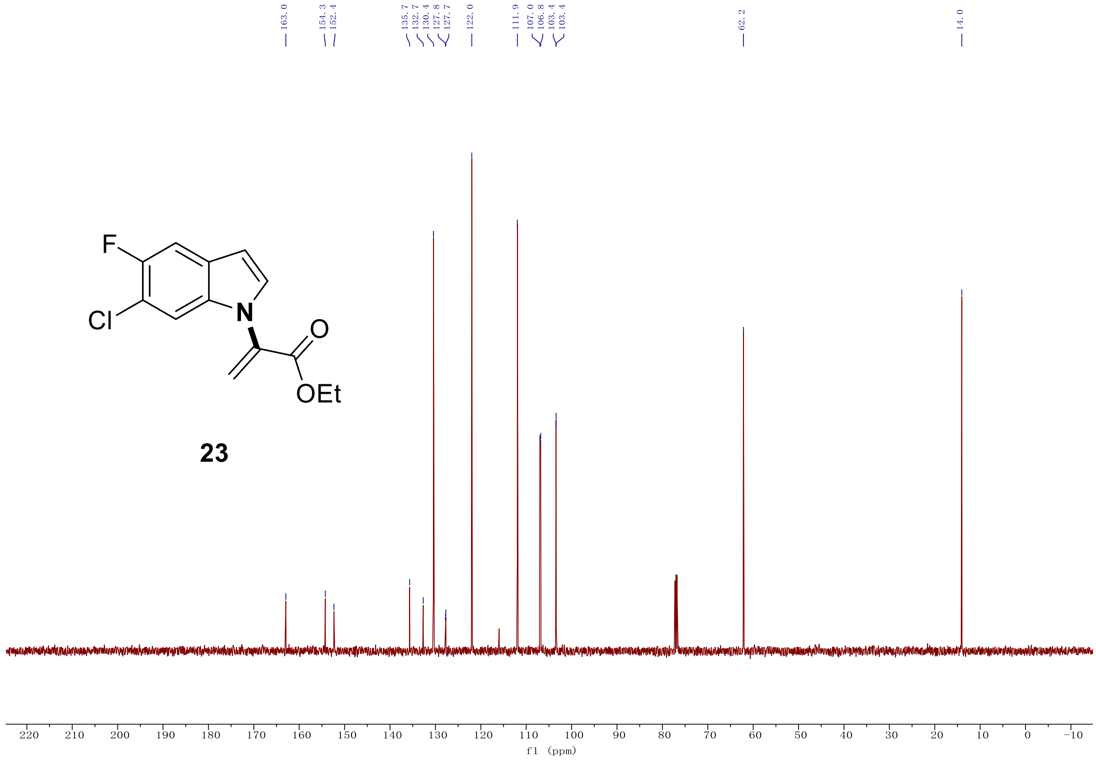

^{13}C NMR spectrum ($CDCl_3$, 126 MHz) of (**23**)

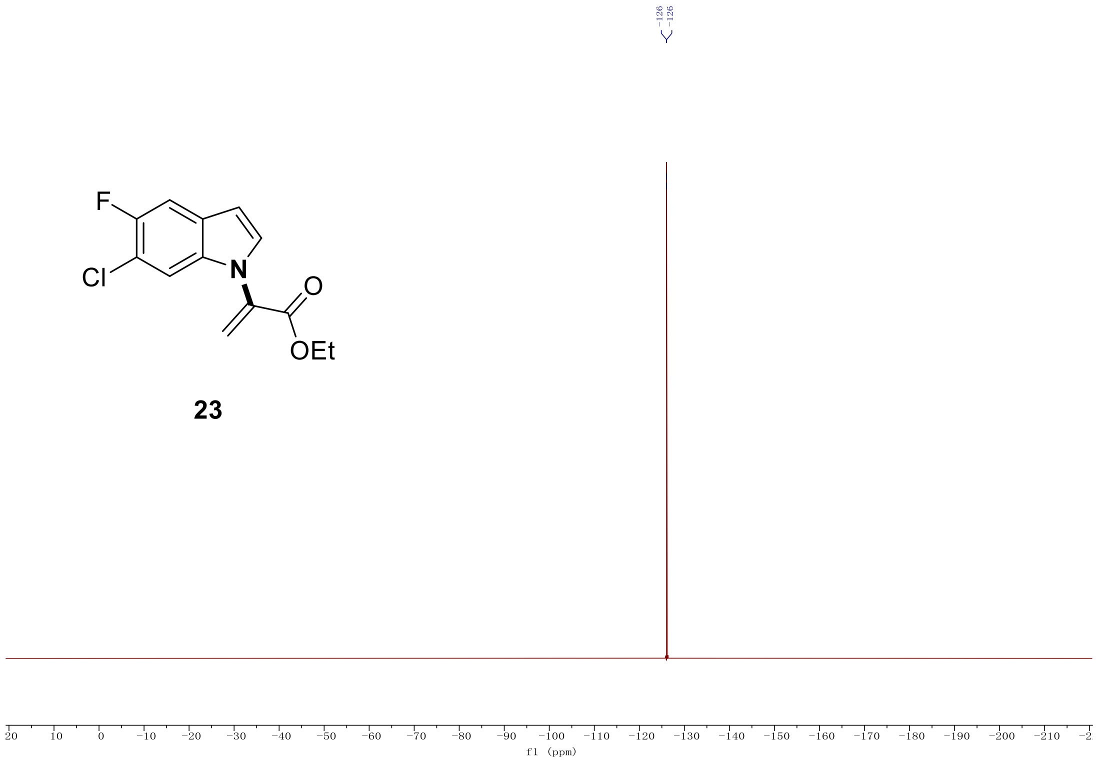

^{19}F NMR spectrum ($CDCl_3$, 471 MHz) of (**23**)

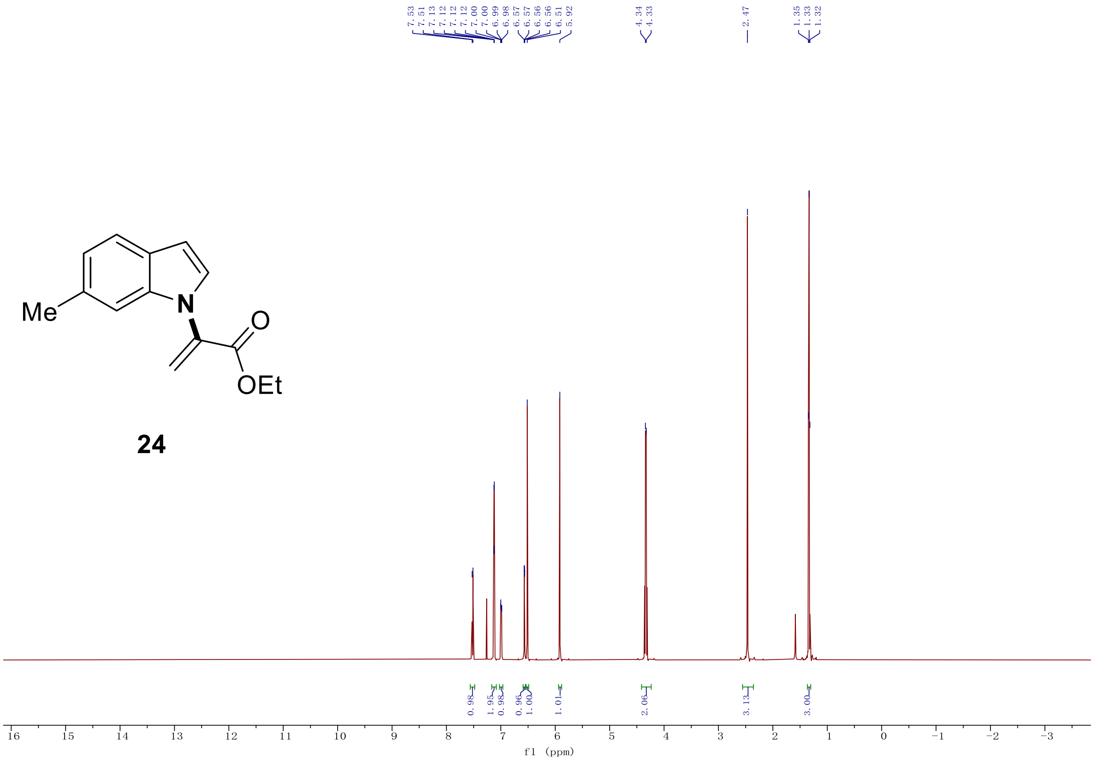

^{1}H NMR spectrum ($CDCl_3$, 500 MHz) of (**24**)

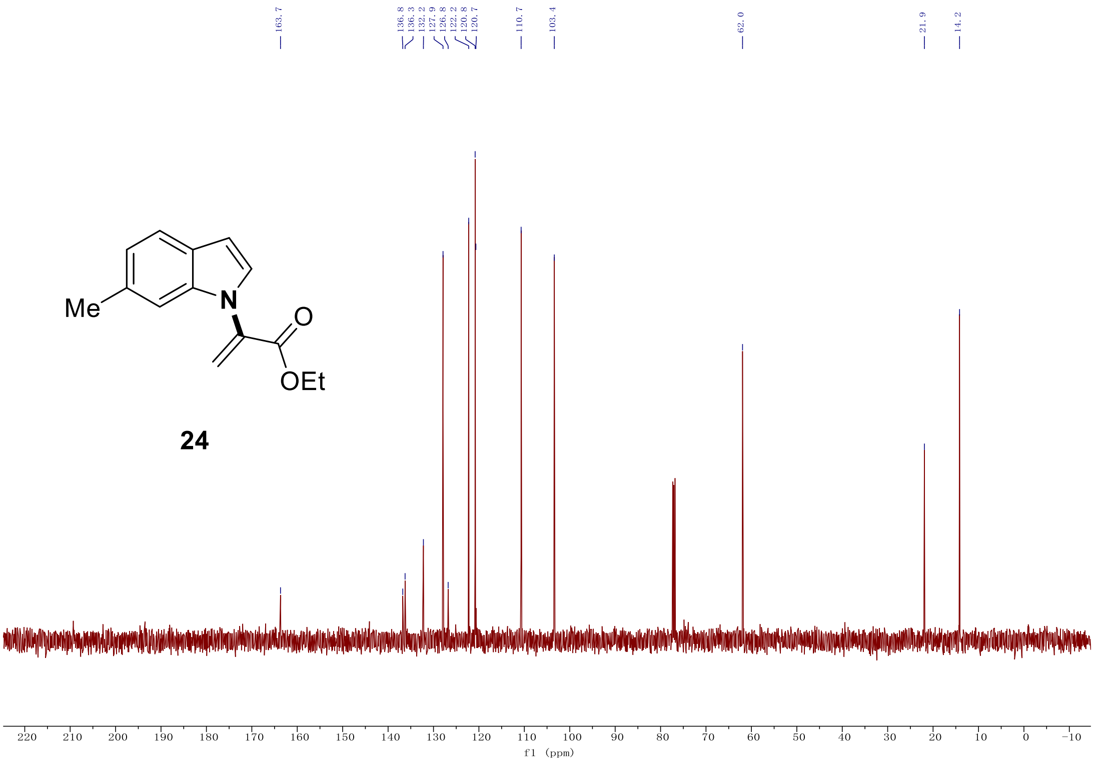

^{13}C NMR spectrum ($CDCl_3$, 126 MHz) of (**24**)

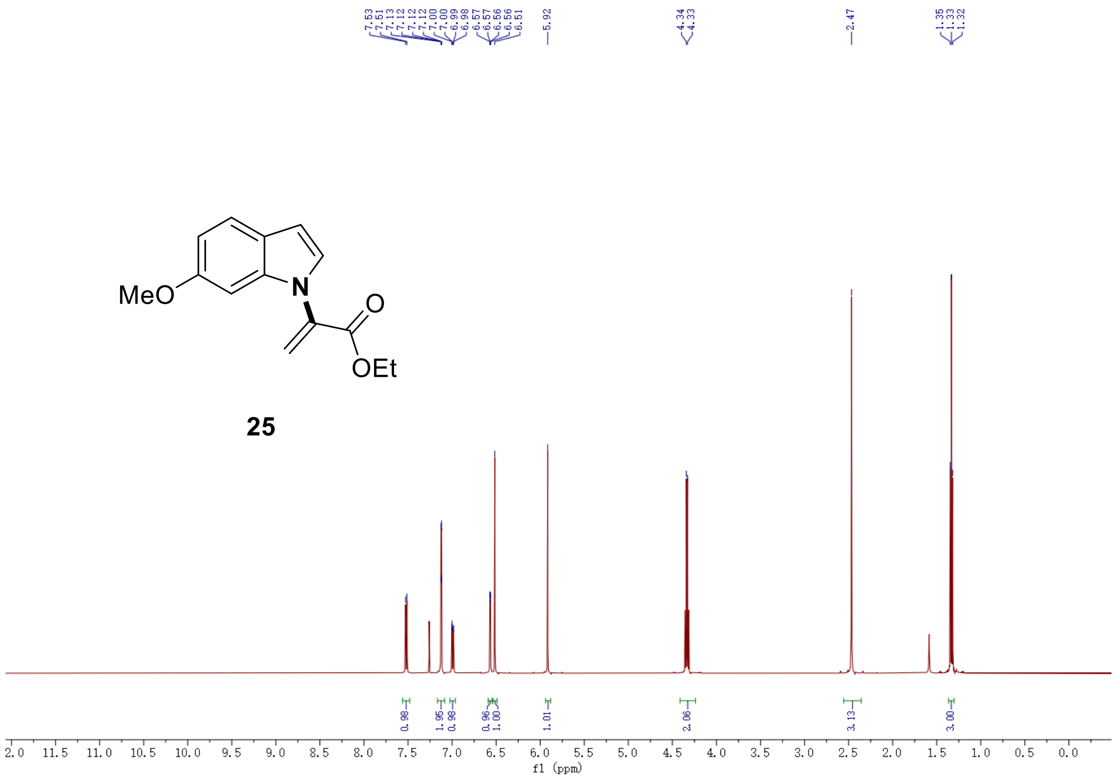

^{1}H NMR spectrum ($CDCl_3$, 500 MHz) of (**25**)

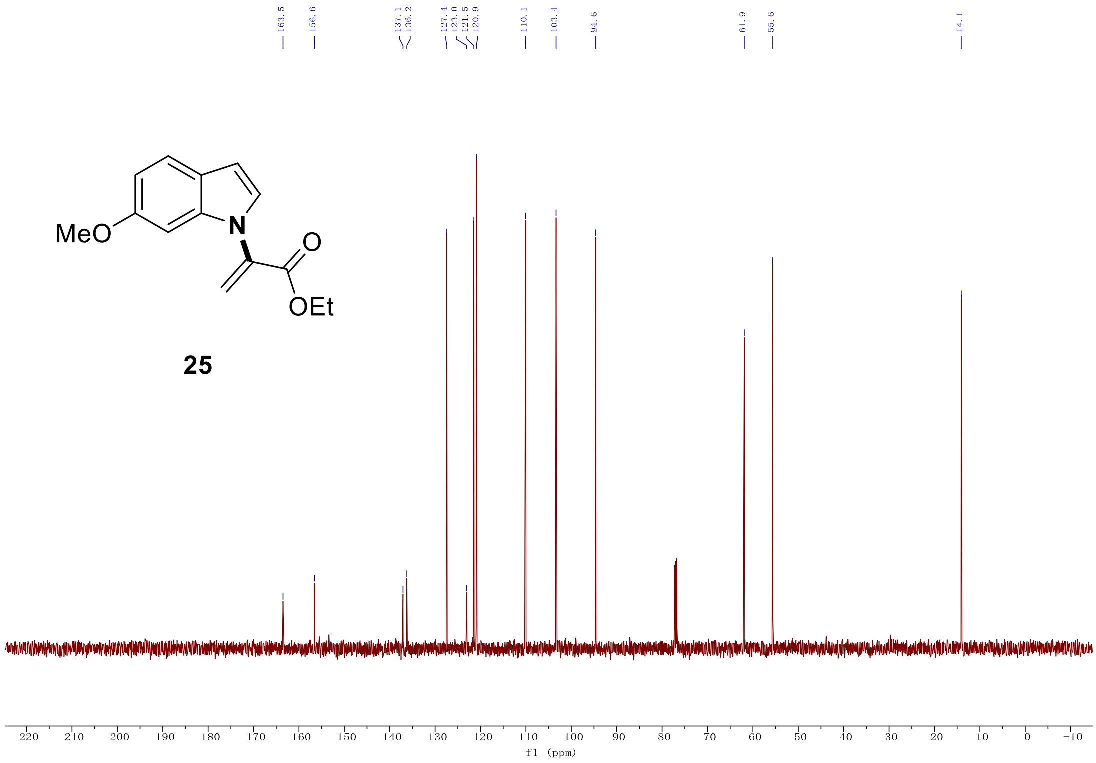

^{13}C NMR spectrum ($CDCl_3$, 126 MHz) of (**25**)

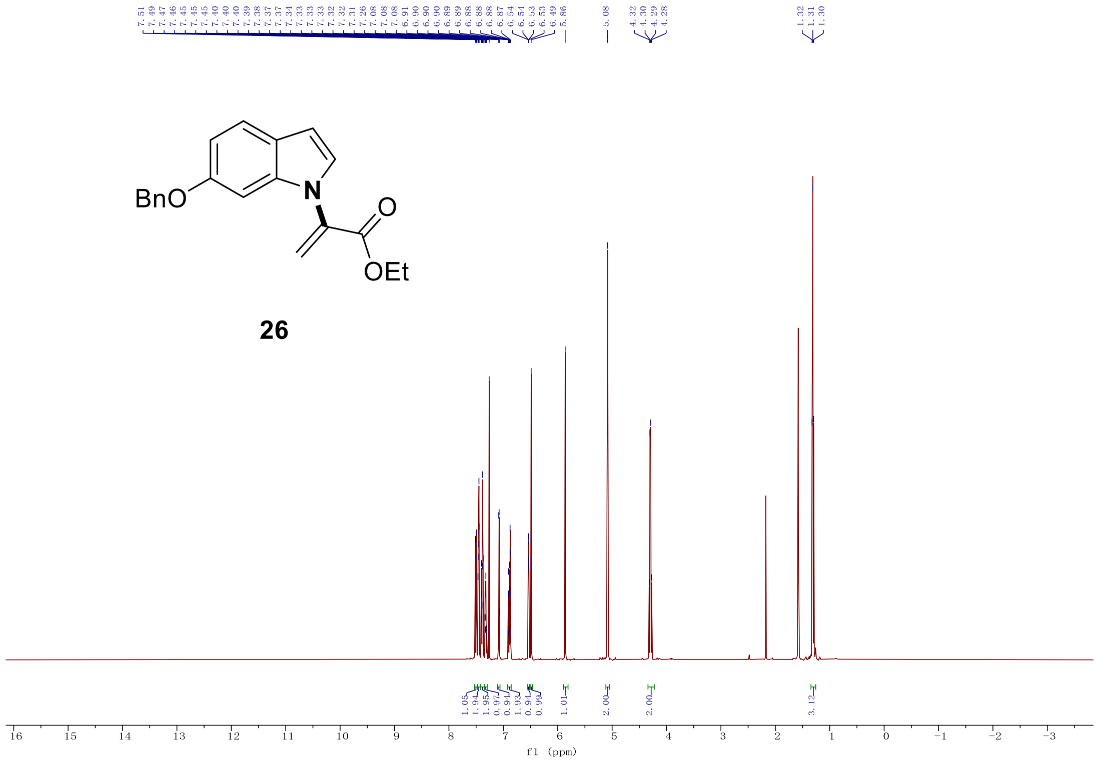

^{1}H NMR spectrum (CDCl3, 500 MHz) of (**26**)

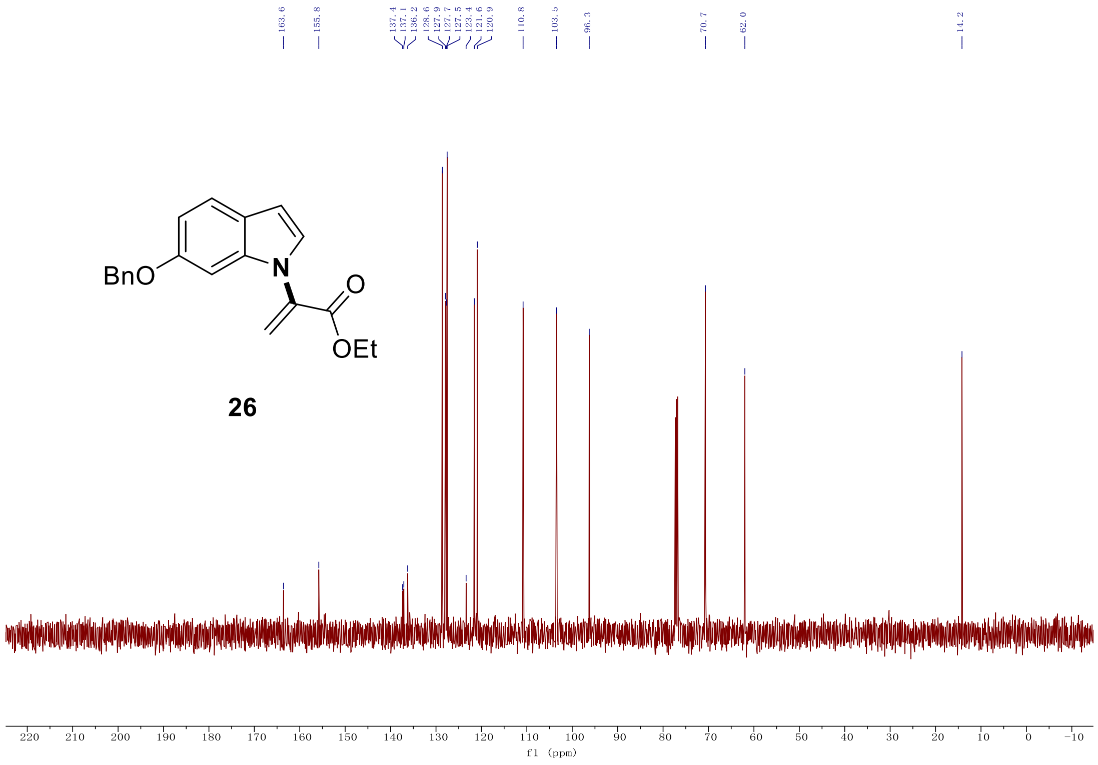

^{13}C NMR spectrum (CDCl3, 126 MHz) of (**26**)

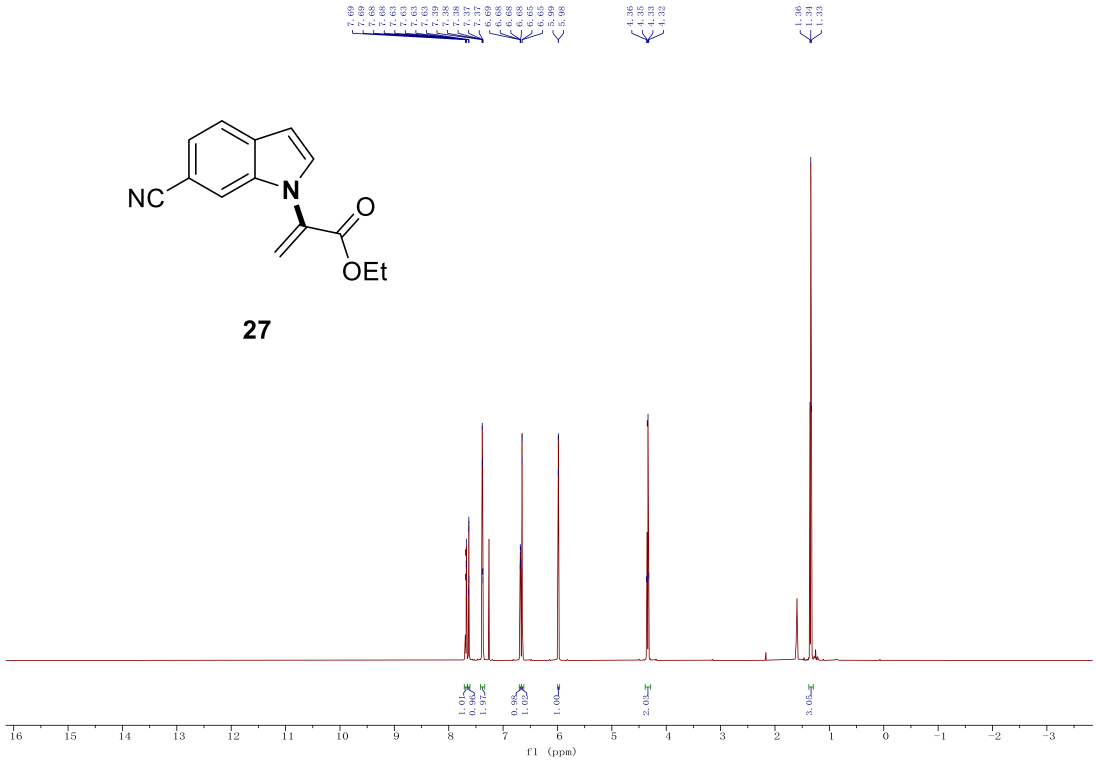

^{1}H NMR spectrum (CDCl$_3$, 500 MHz) of (**27**)

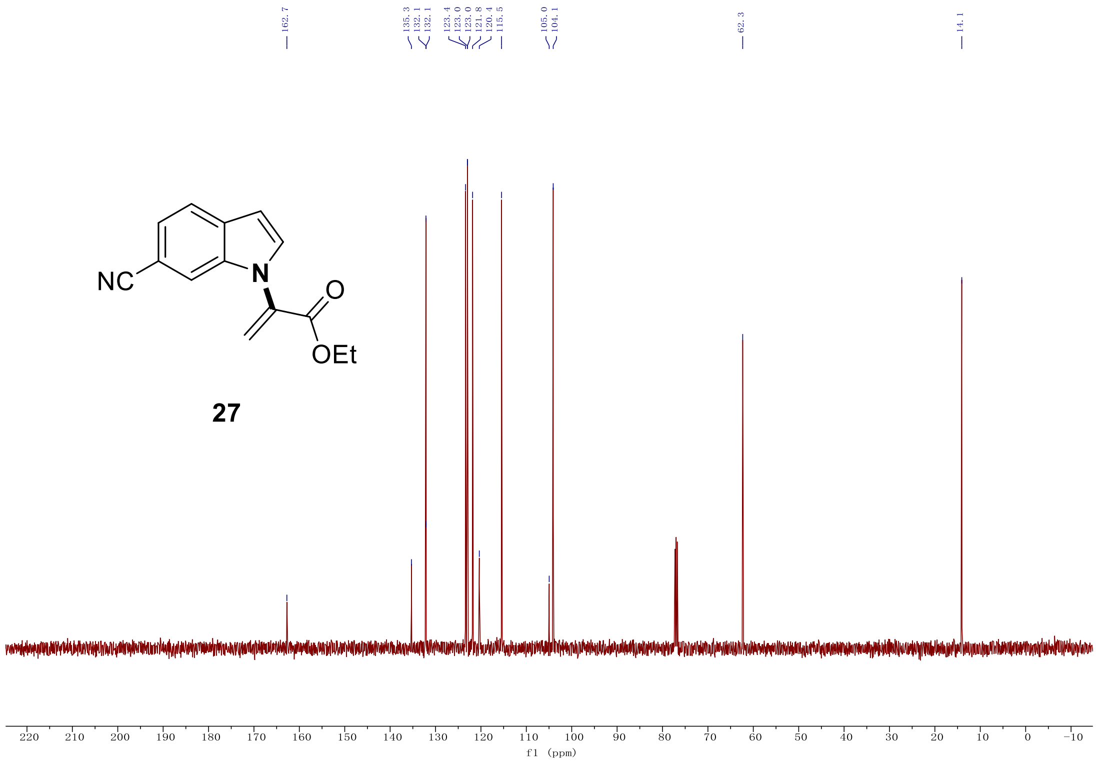

^{13}C NMR spectrum (CDCl$_3$, 126 MHz) of (**27**)

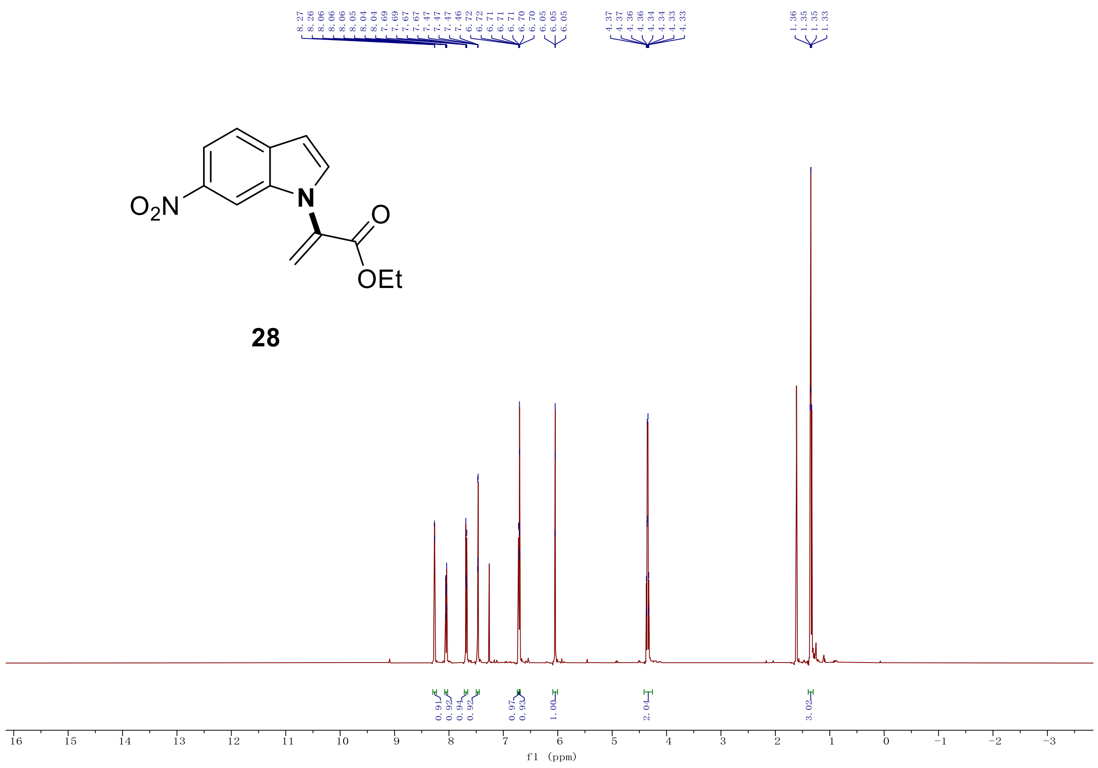

^{1}H NMR spectrum (CDCl$_3$, 500 MHz) of (**28**)

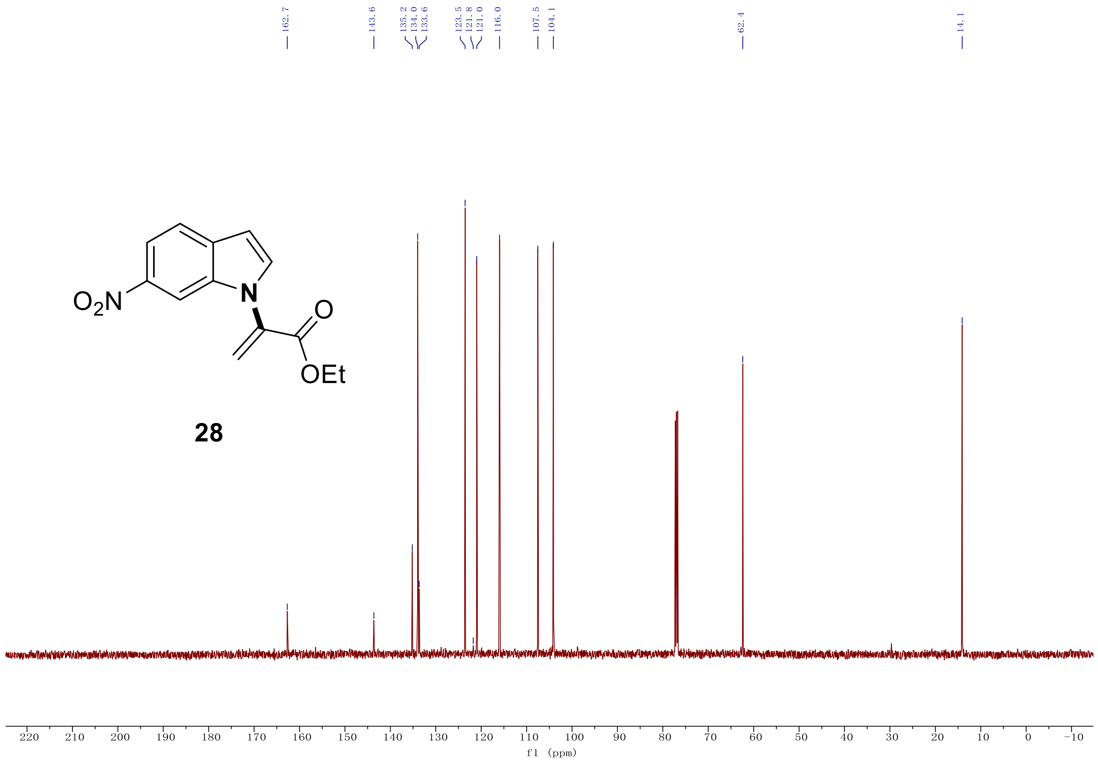

^{13}C NMR spectrum (CDCl$_3$, 126 MHz) of (**28**)

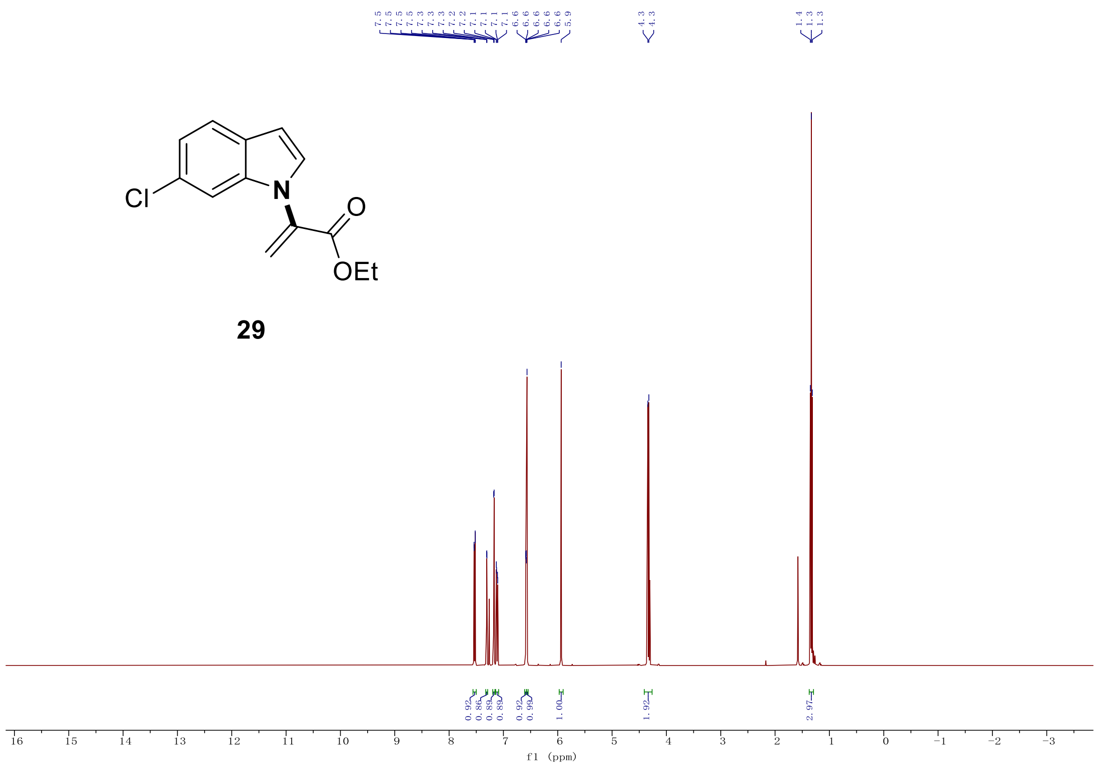

^{1}H NMR spectrum ($CDCl_3$, 400 MHz) of (**29**)

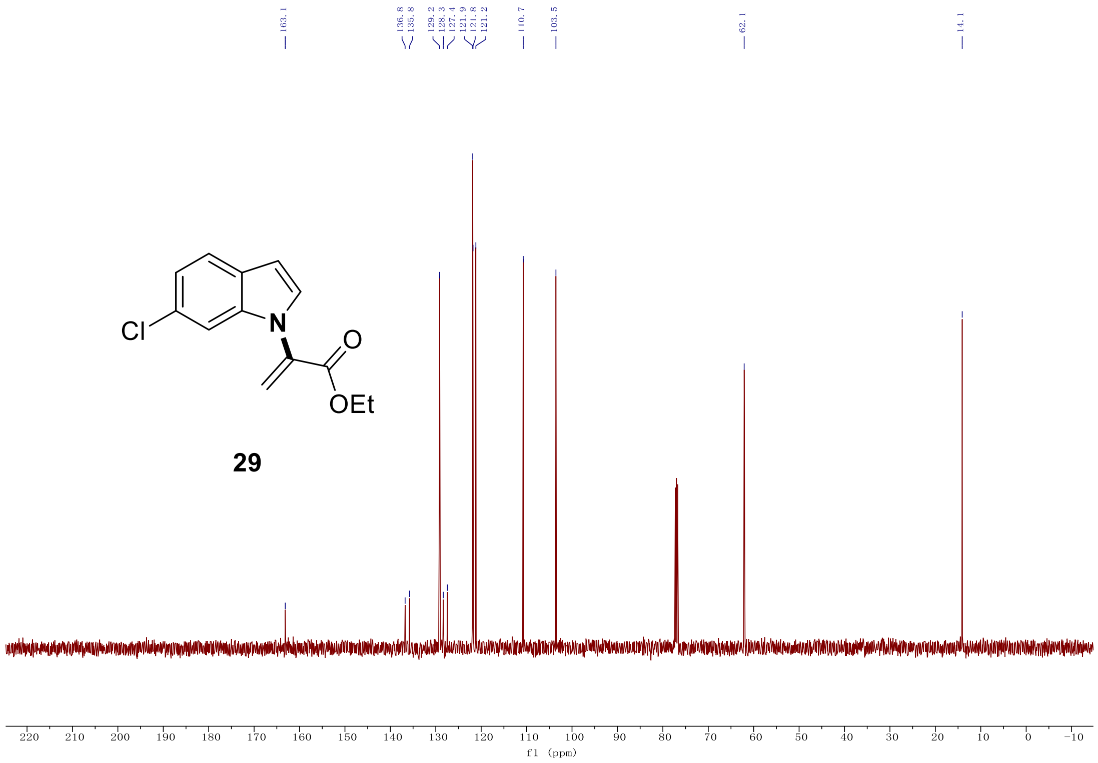

^{13}C NMR spectrum ($CDCl_3$, 126 MHz) of (**29**)

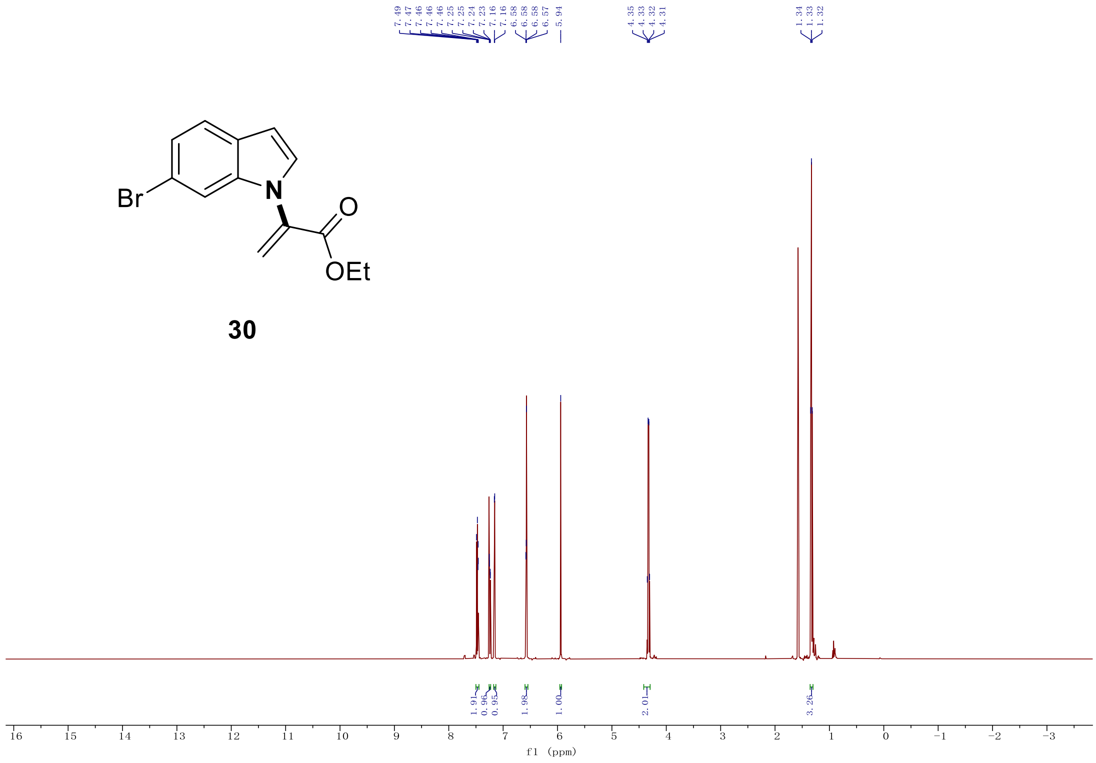

^{1}H NMR spectrum (CDCl3, 500 MHz) of (**30**)

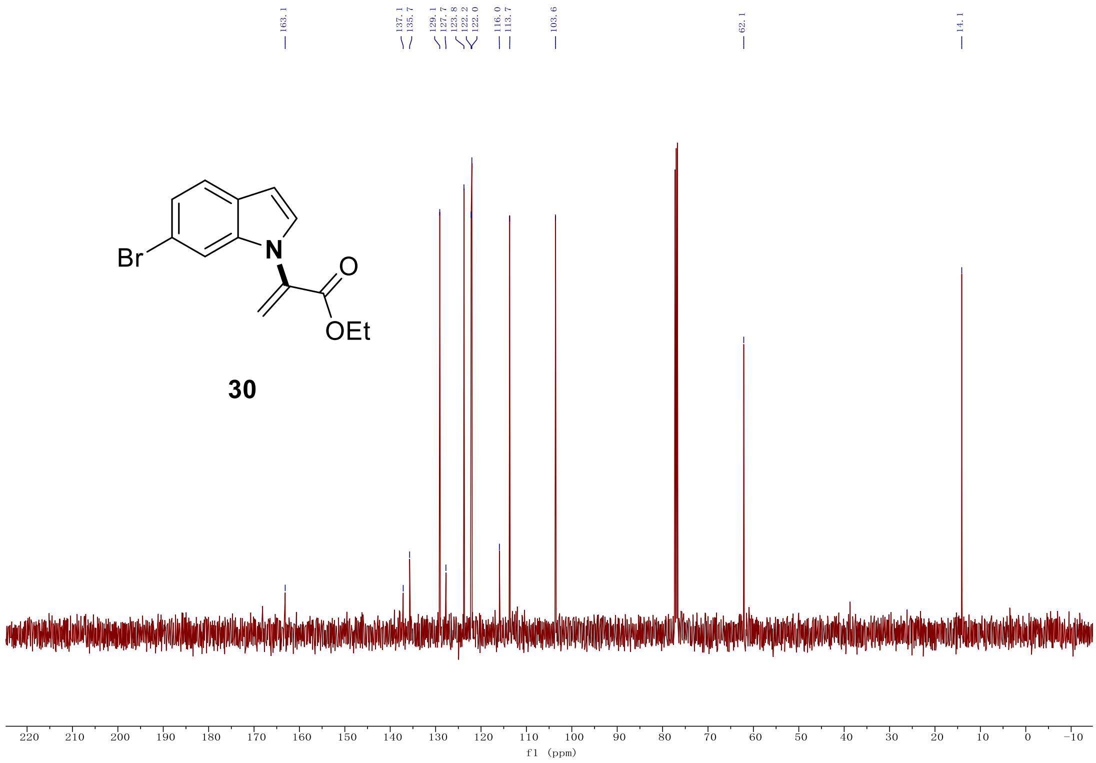

^{13}C NMR spectrum (CDCl3, 126 MHz) of (**30**)

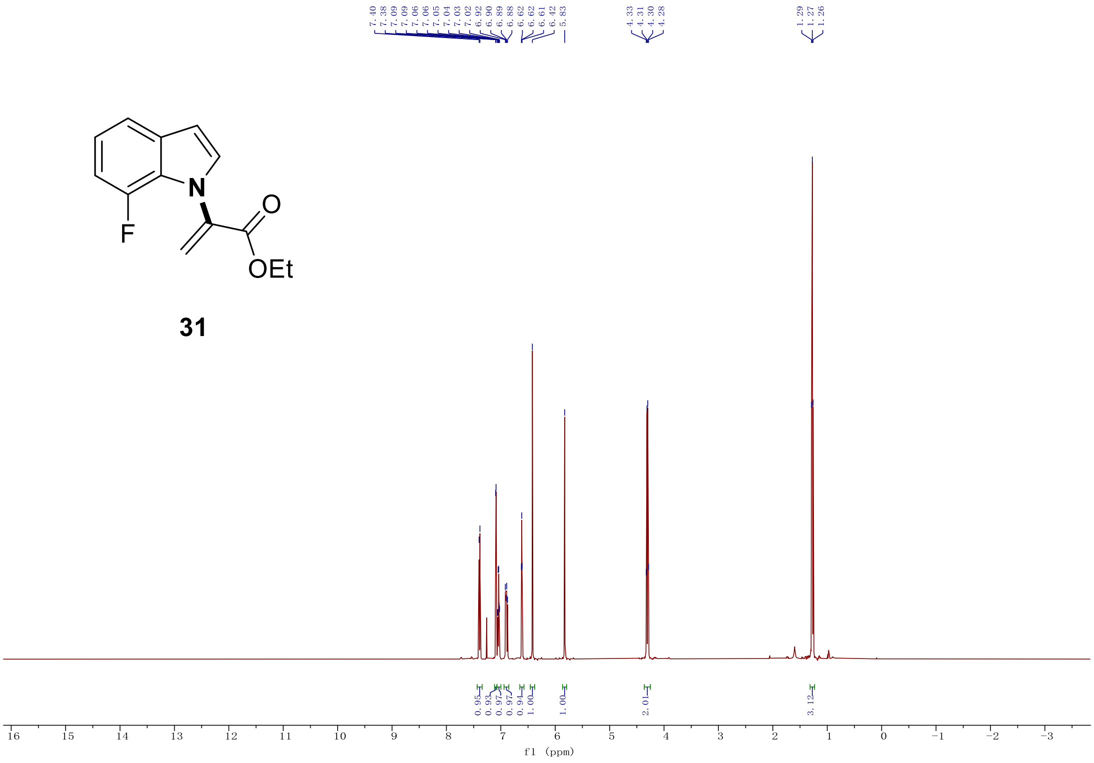

^{1}H NMR spectrum (CDCl$_3$, 500 MHz) of (**31**)

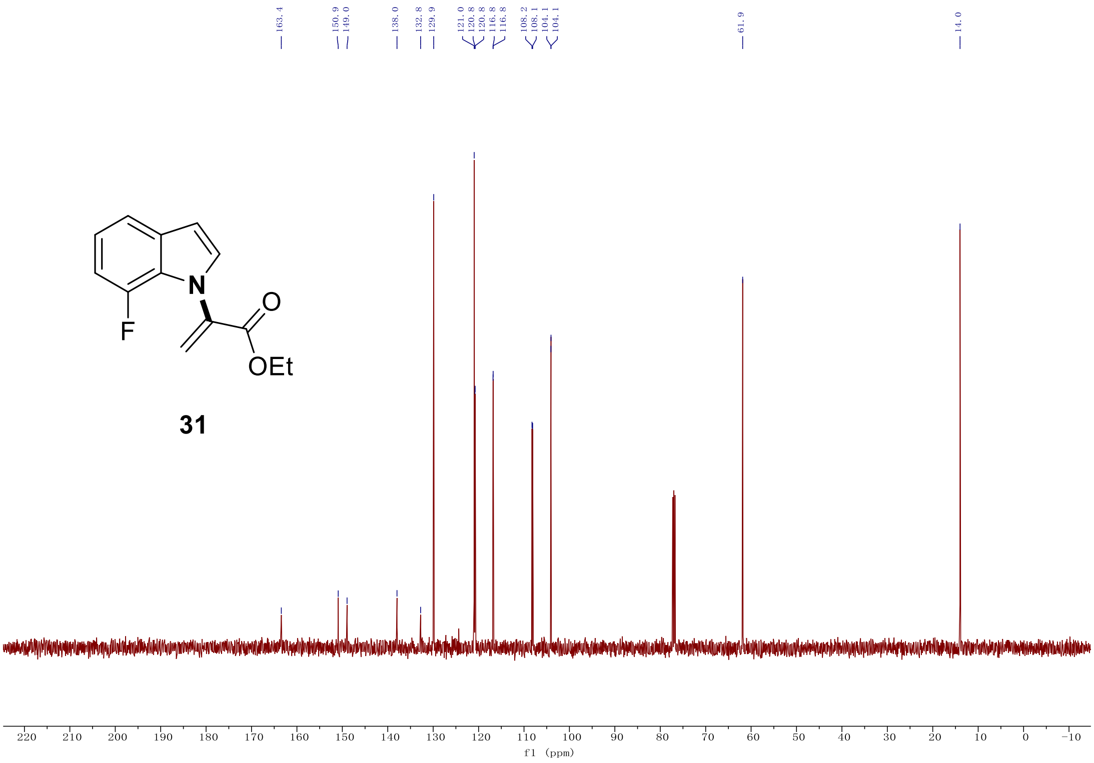

^{13}C NMR spectrum (CDCl$_3$, 126 MHz) of (**31**)

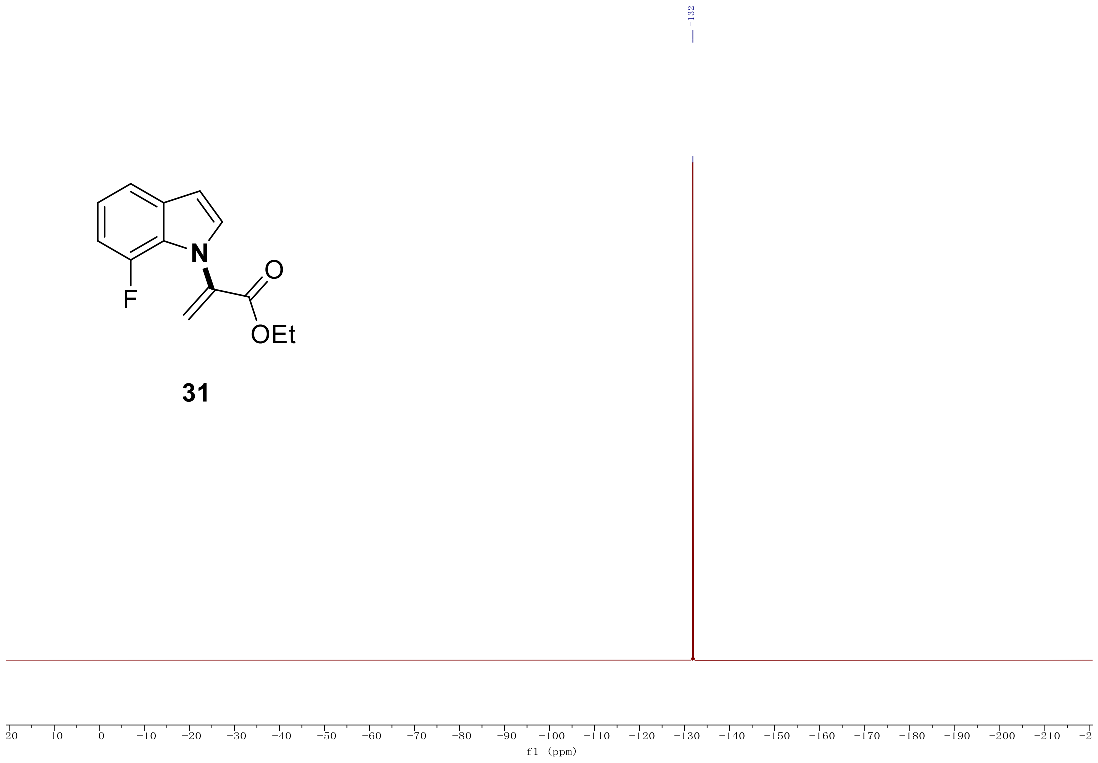

^{19}F NMR spectrum ($CDCl_3$, 471 MHz) of (**31**)

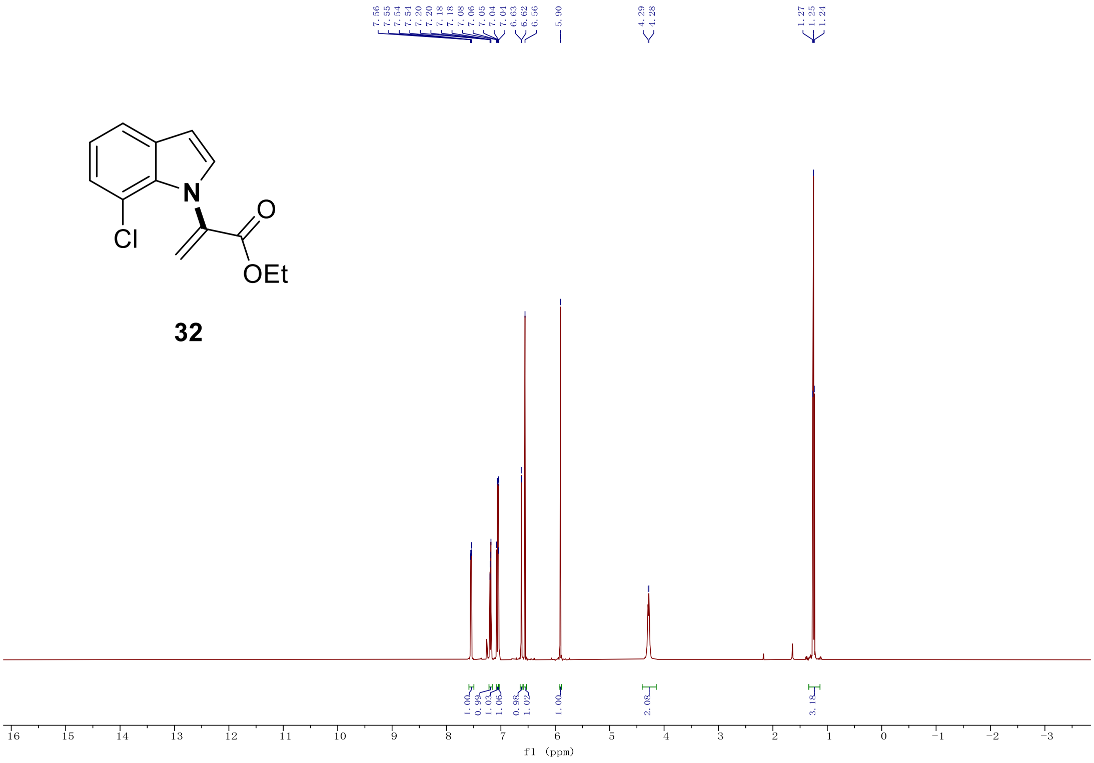

^1H NMR spectrum ($CDCl_3$, 500 MHz) of (**32**)

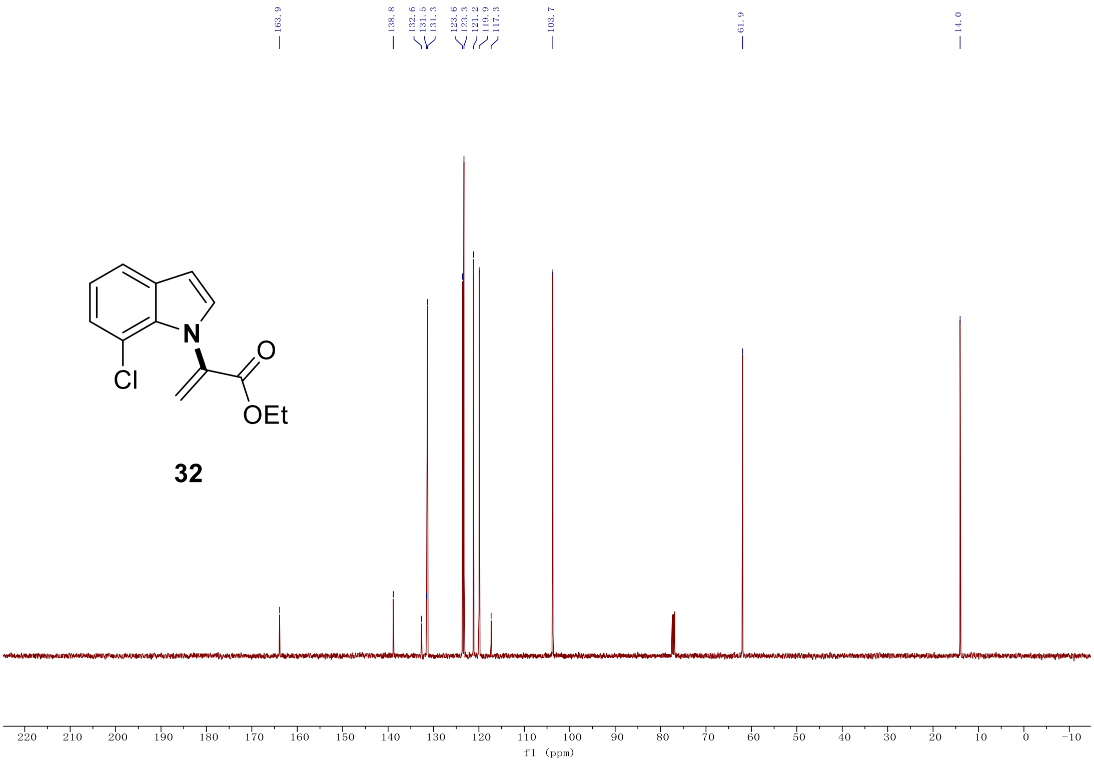

^{13}C NMR spectrum ($CDCl_3$, 126 MHz) of (**32**)

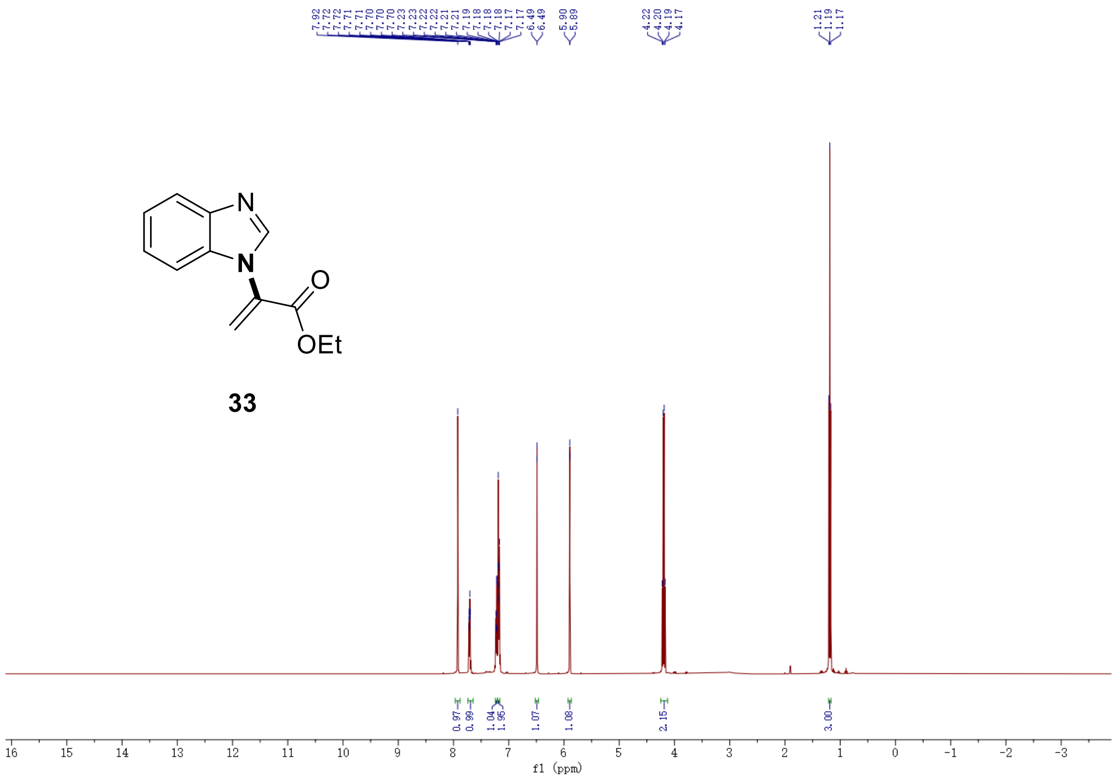

^{1}H NMR spectrum ($CDCl_3$, 400 MHz) of (**33**)

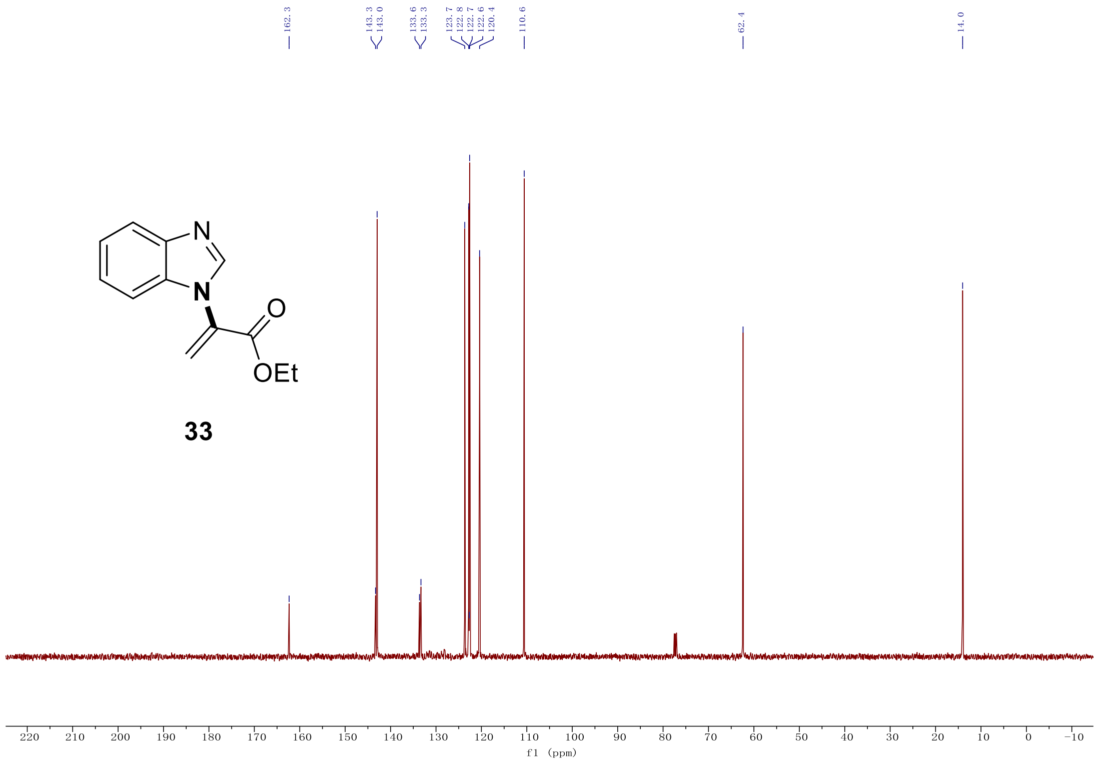

^{13}C NMR spectrum ($CDCl_3$, 126 MHz) of (**33**)

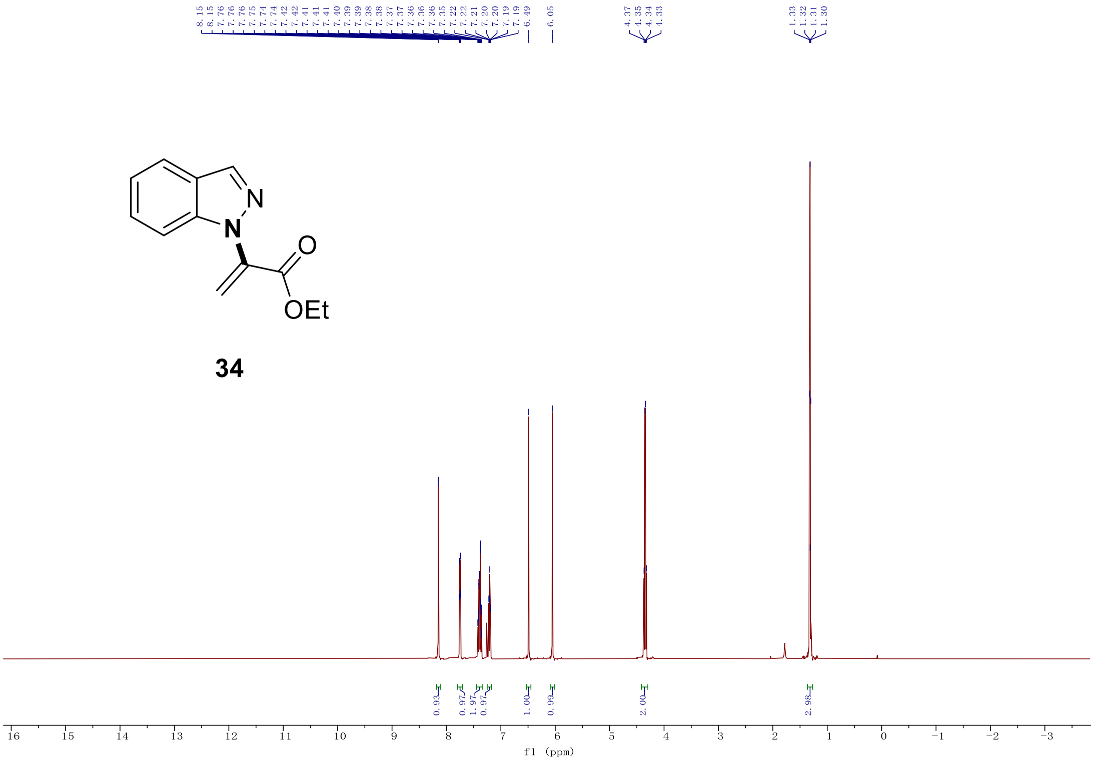

^{1}H NMR spectrum (CDCl$_3$, 500 MHz) of (**34**)

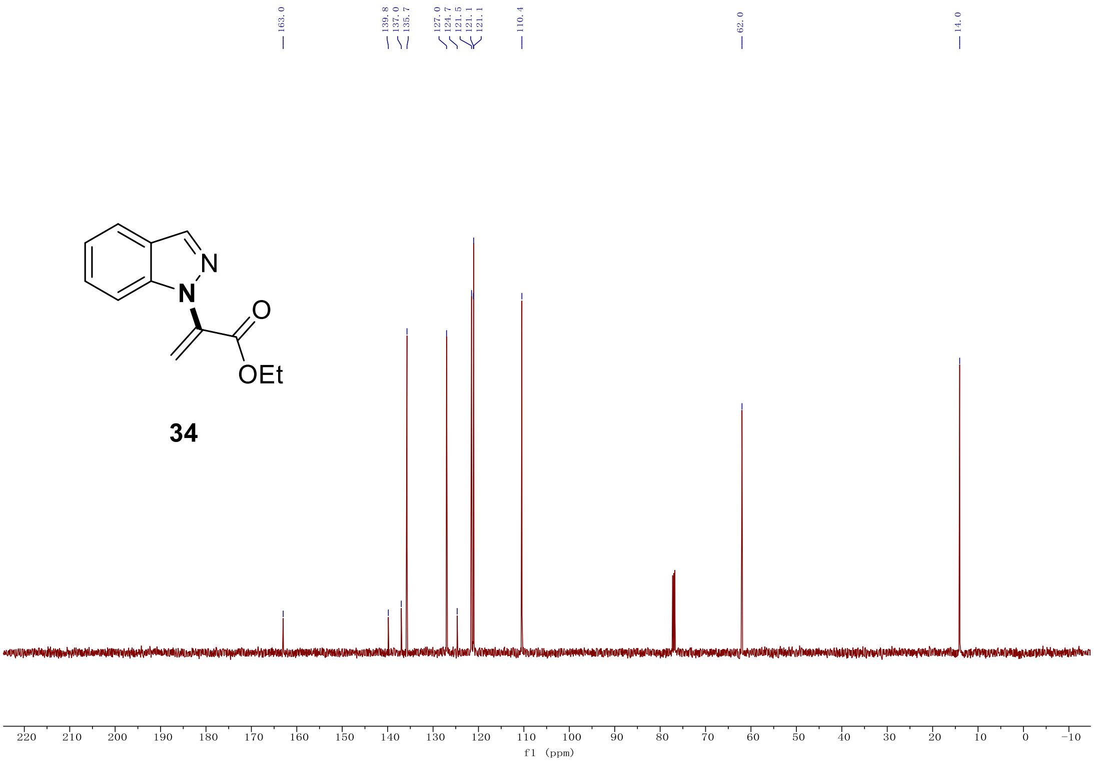

^{13}C NMR spectrum (CDCl$_3$, 126 MHz) of (**34**)

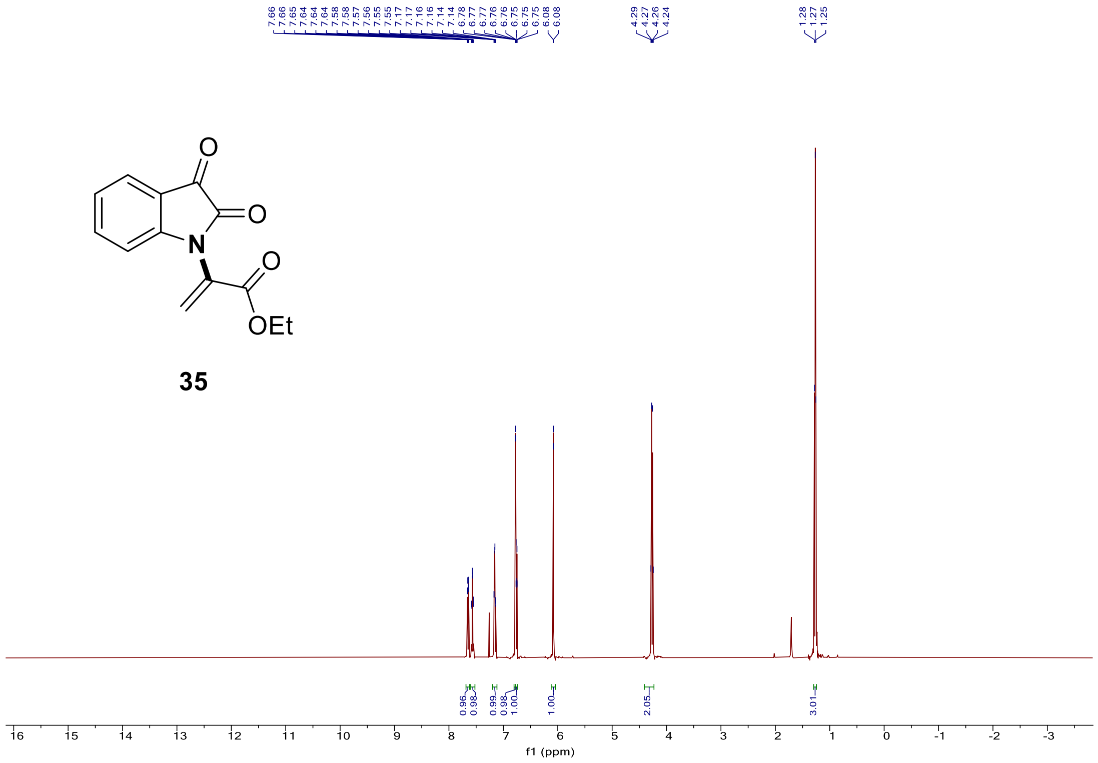

^{1}H NMR spectrum ($CDCl_3$, 500 MHz) of (**35**)

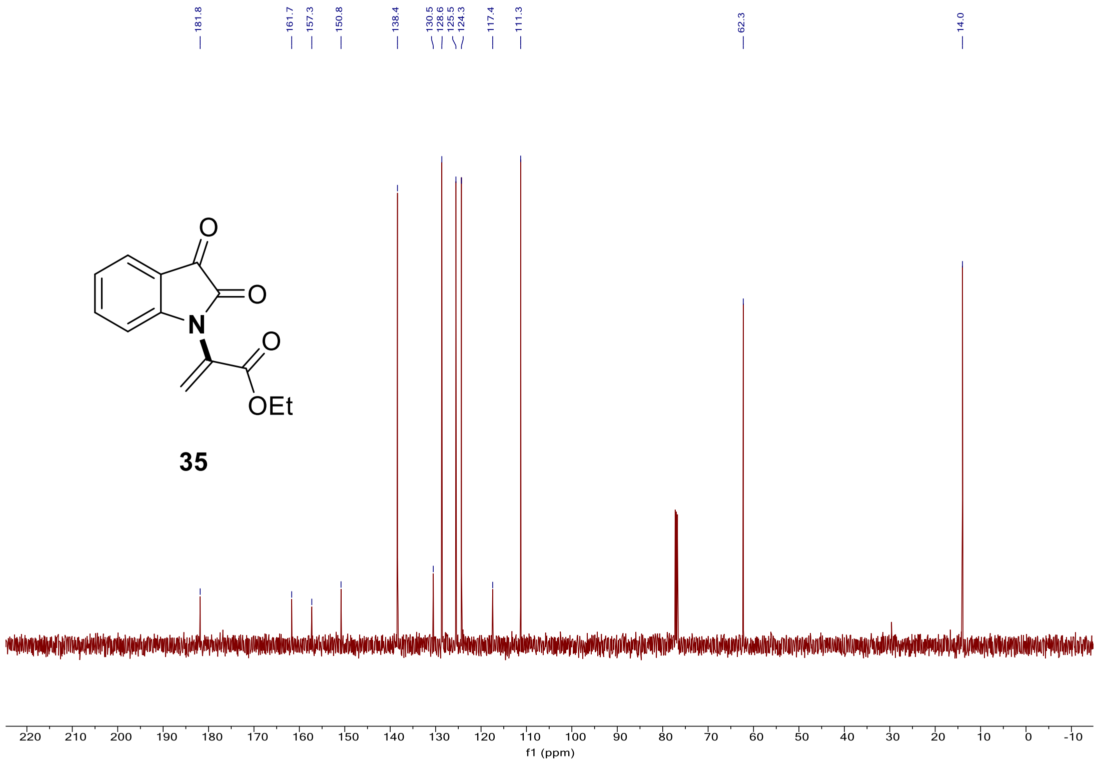

^{13}C NMR spectrum ($CDCl_3$, 126 MHz) of (**35**)

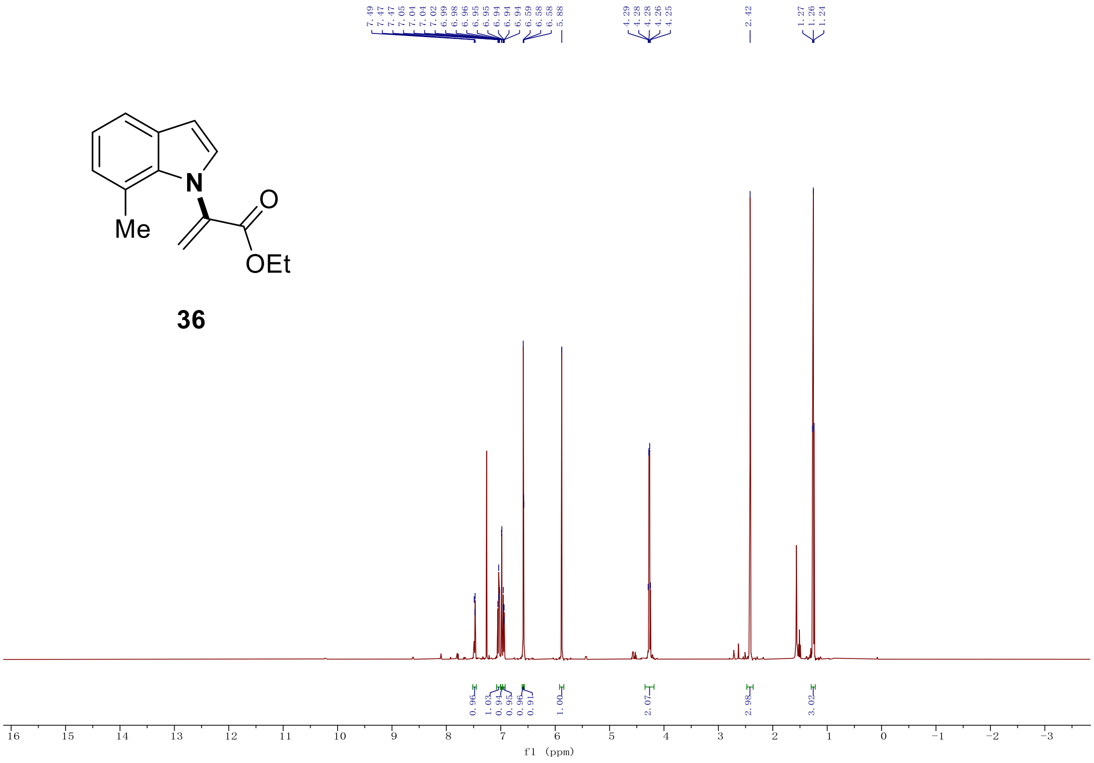

^{1}H NMR spectrum (CDCl$_3$, 500 MHz) of (**36**)

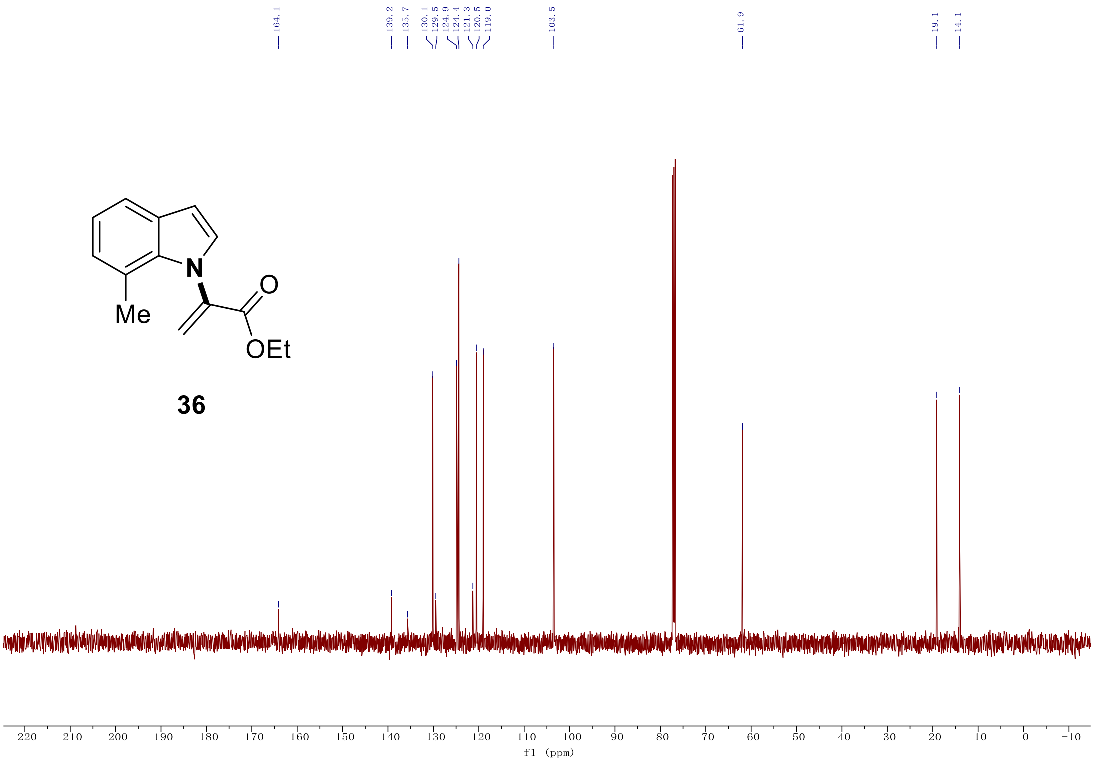

^{13}C NMR spectrum (CDCl$_3$, 126 MHz) of (**36**)

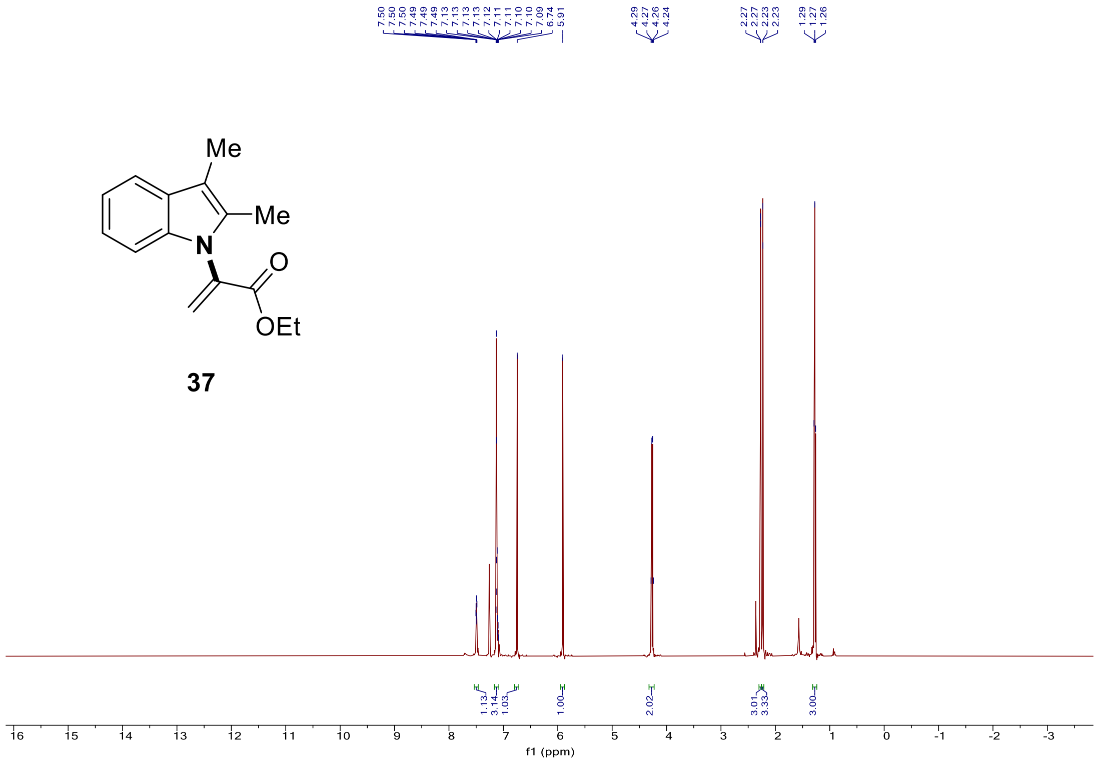

^{1}H NMR spectrum (CDCl3, 500 MHz) of (**37**)

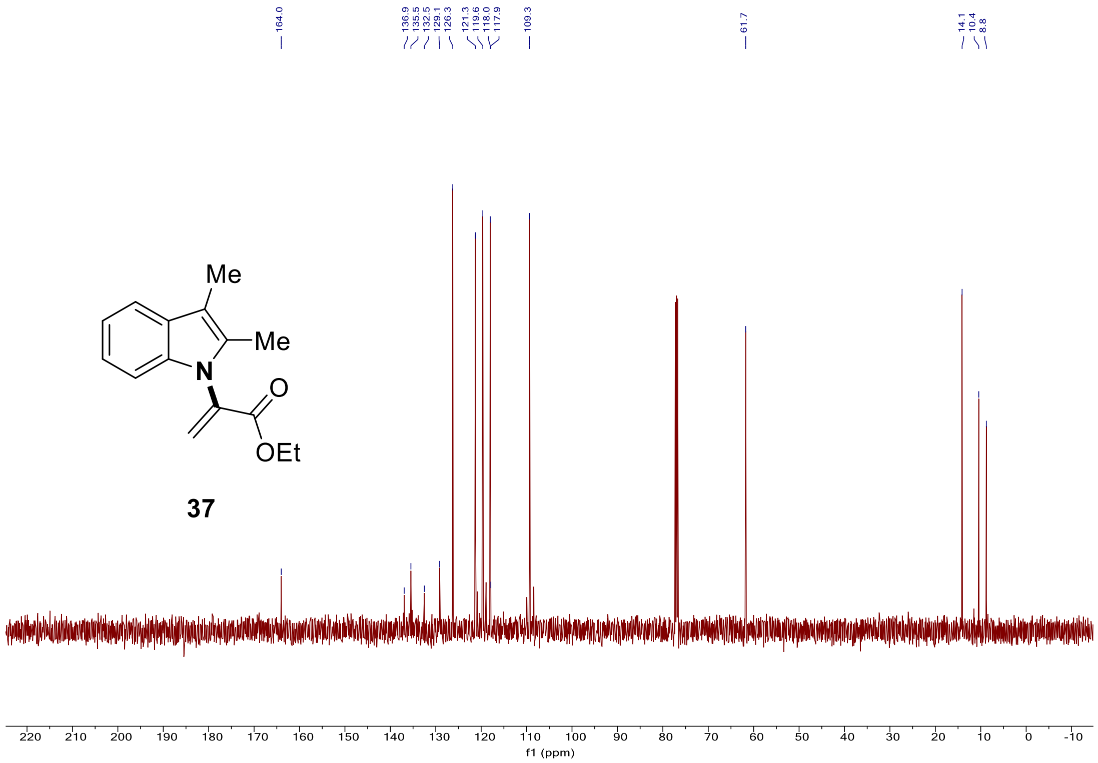

^{13}C NMR spectrum (CDCl3, 126 MHz) of (**37**)

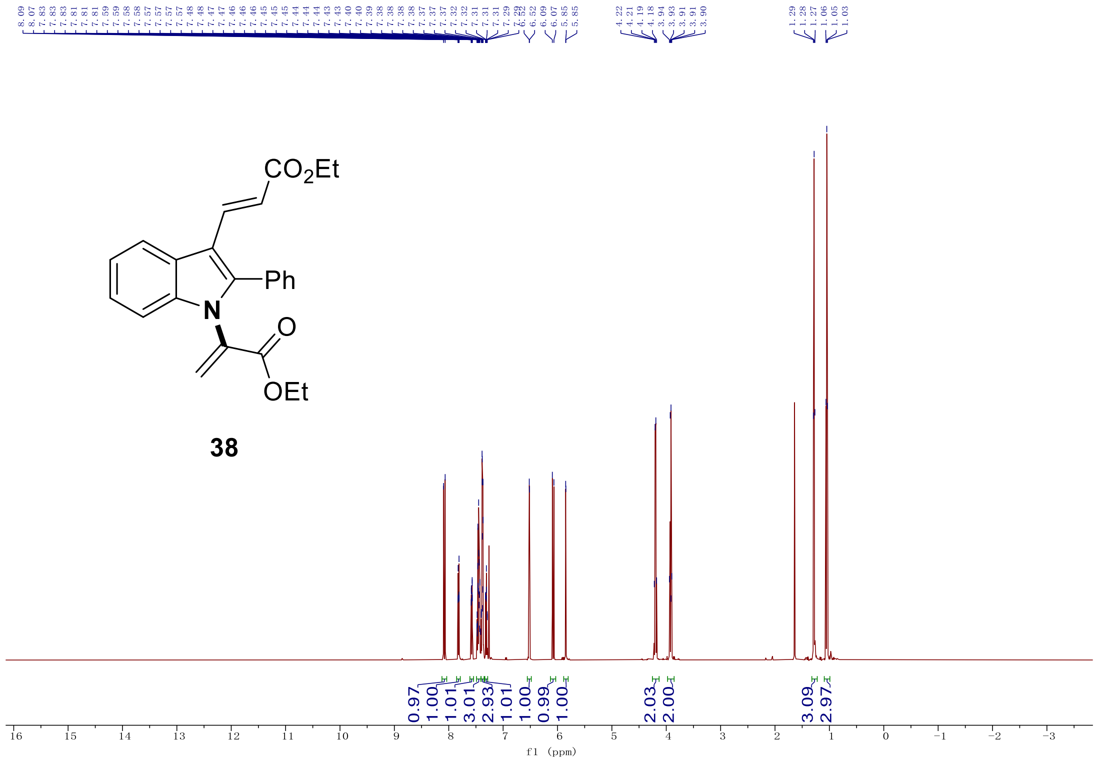

^{1}H NMR spectrum (CDCl$_3$, 500 MHz) of (**38**)

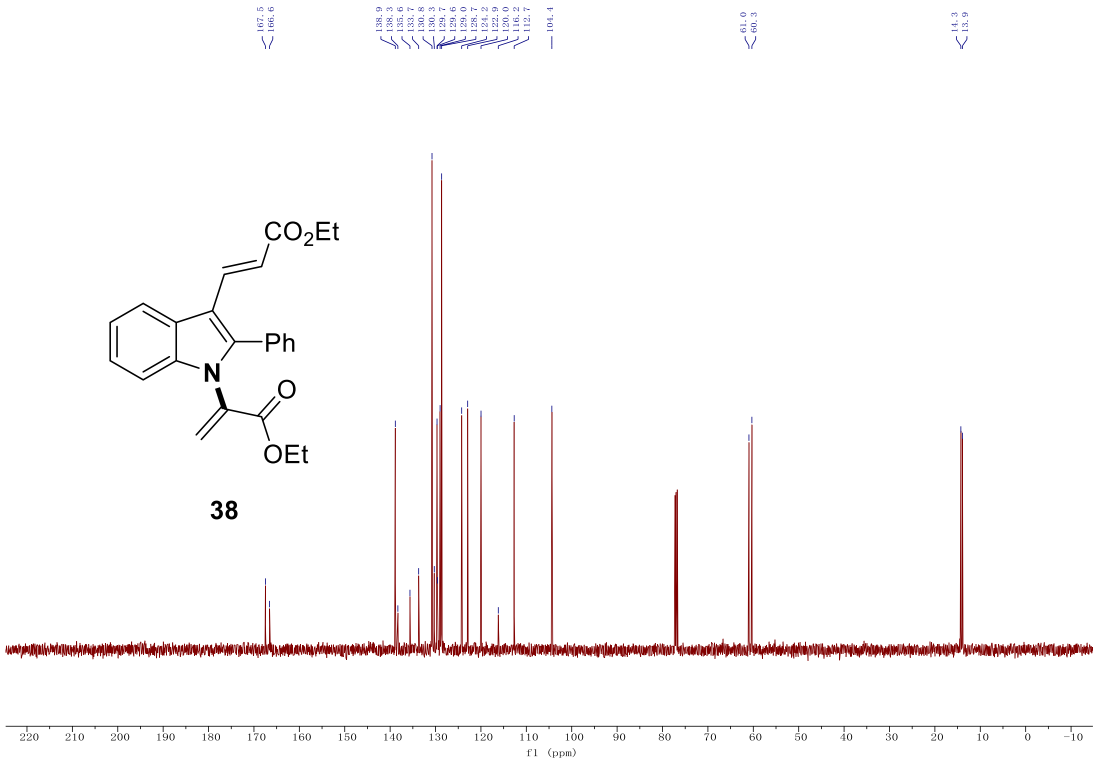

^{13}C NMR spectrum (CDCl$_3$, 126 MHz) of (**38**)

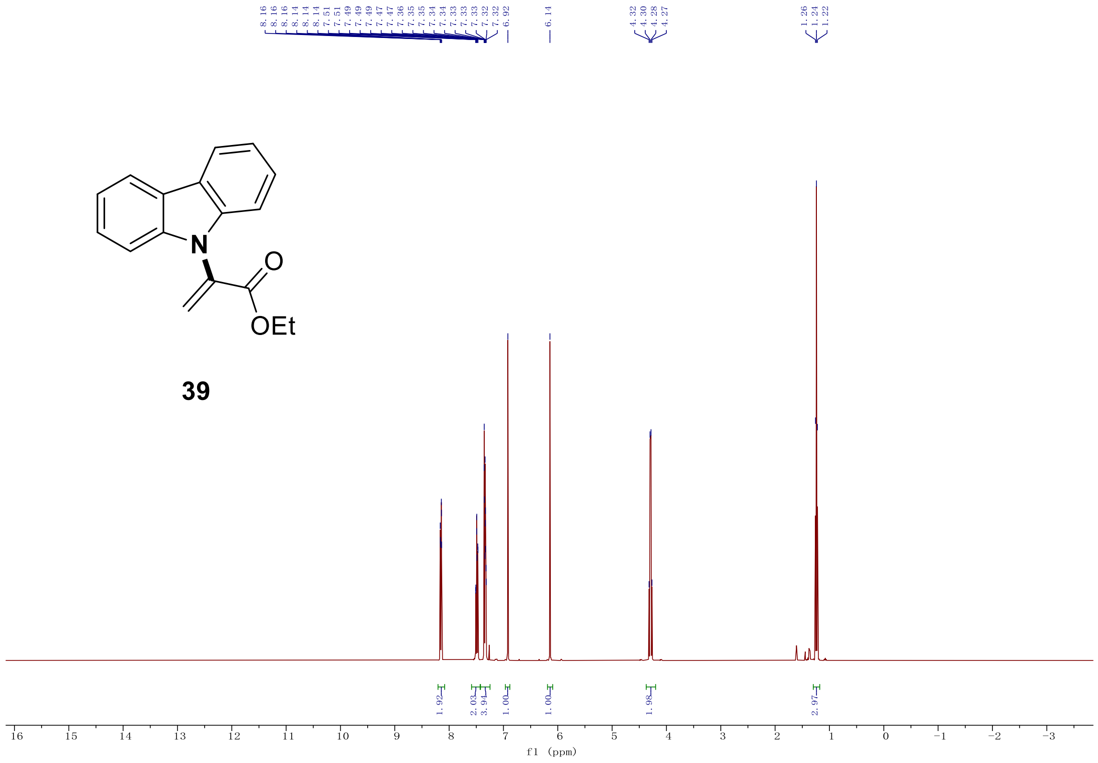

^{1}H NMR spectrum ($CDCl_3$, 500 MHz) of (**39**)

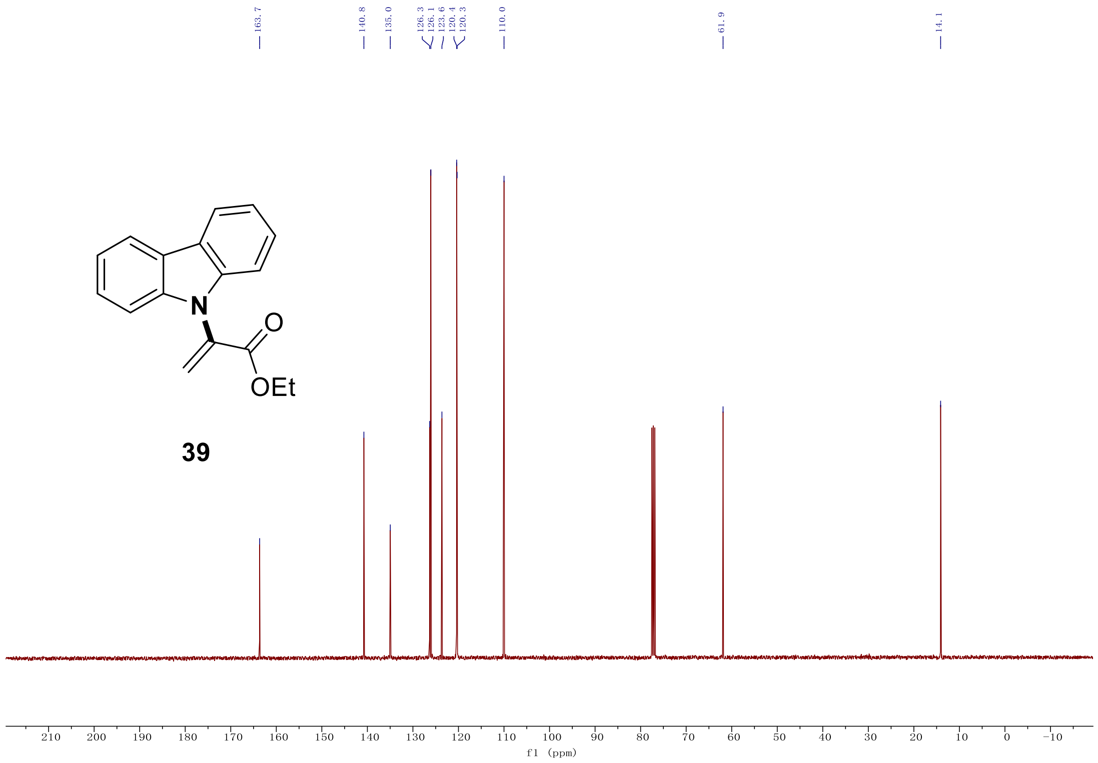

^{13}C NMR spectrum ($CDCl_3$, 126 MHz) of (**39**)

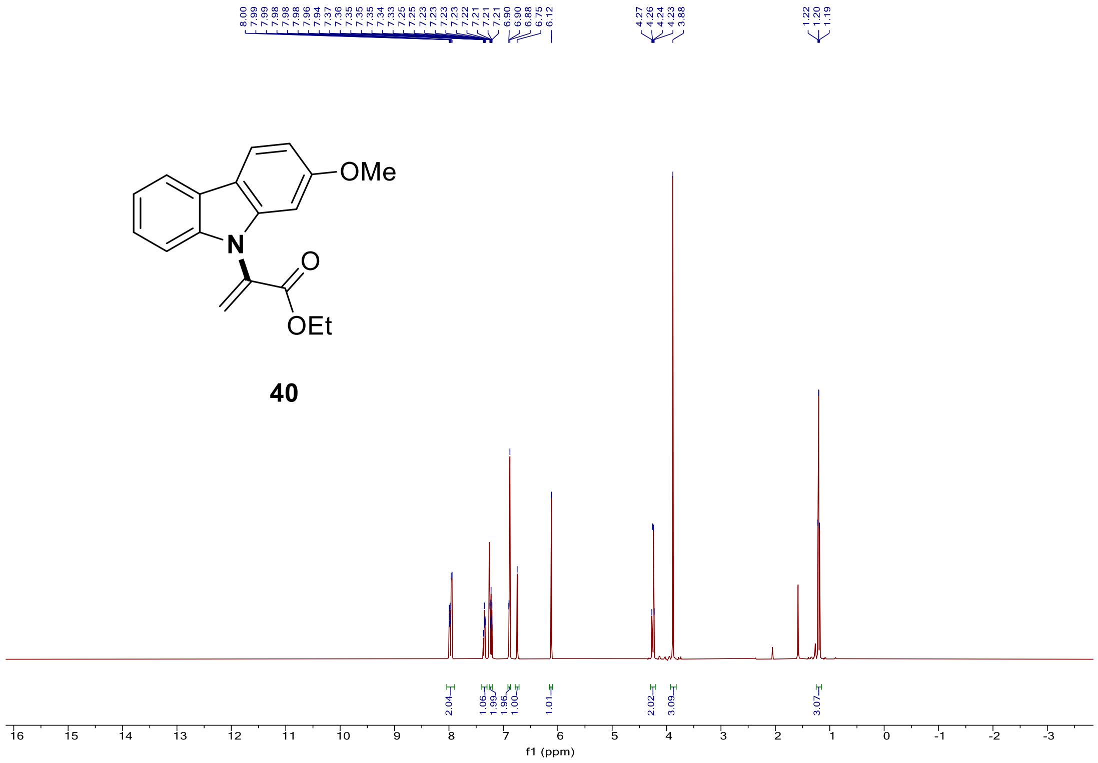

^{1}H NMR spectrum ($CDCl_3$, 500 MHz) of (**40**)

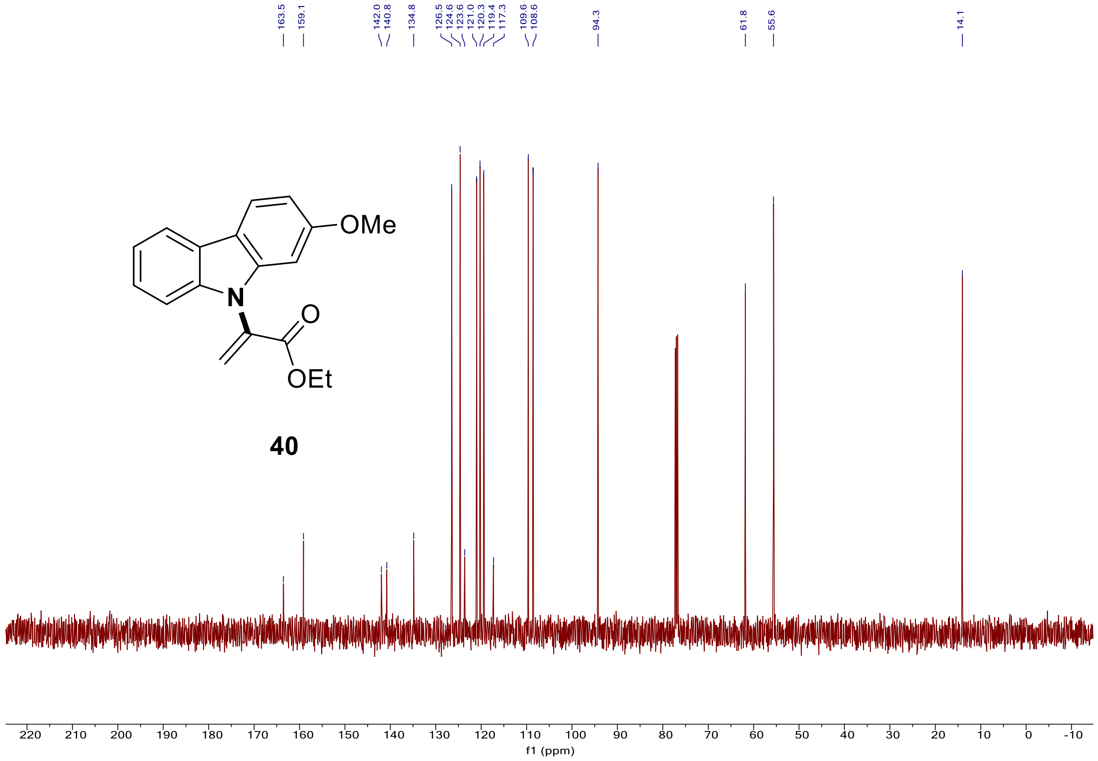

^{13}C NMR spectrum ($CDCl_3$, 126 MHz) of (**40**)

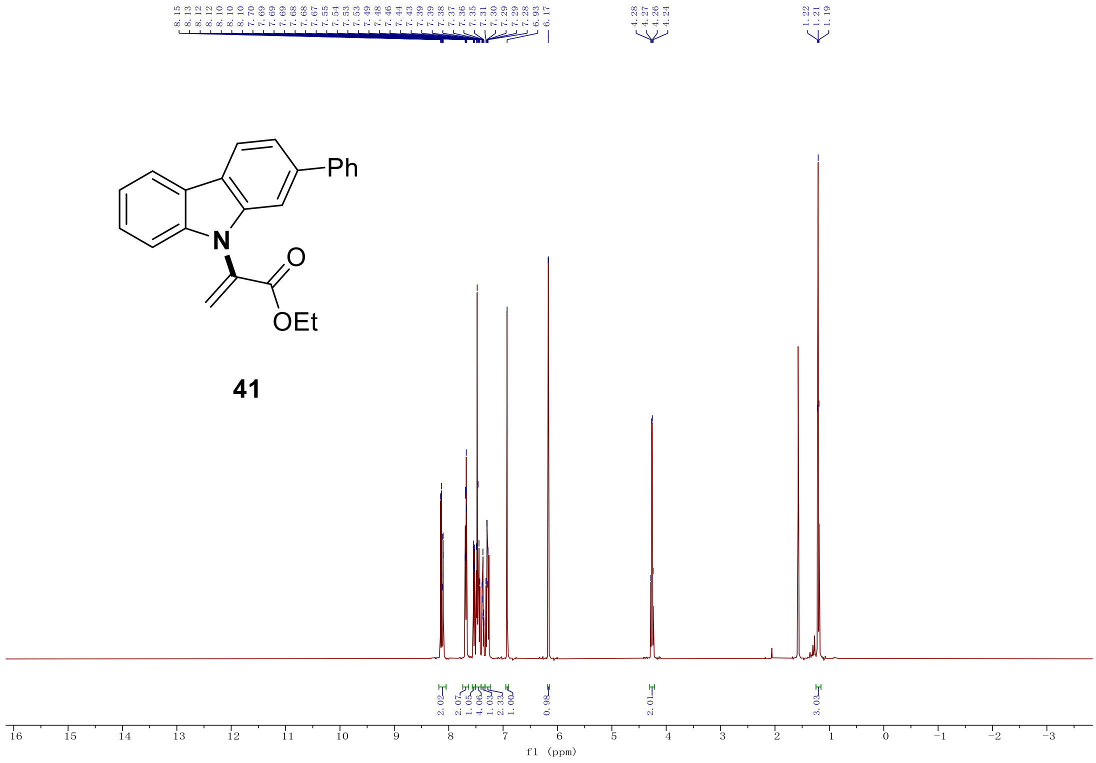

^{1}H NMR spectrum (CDCl$_3$, 500 MHz) of (**41**)

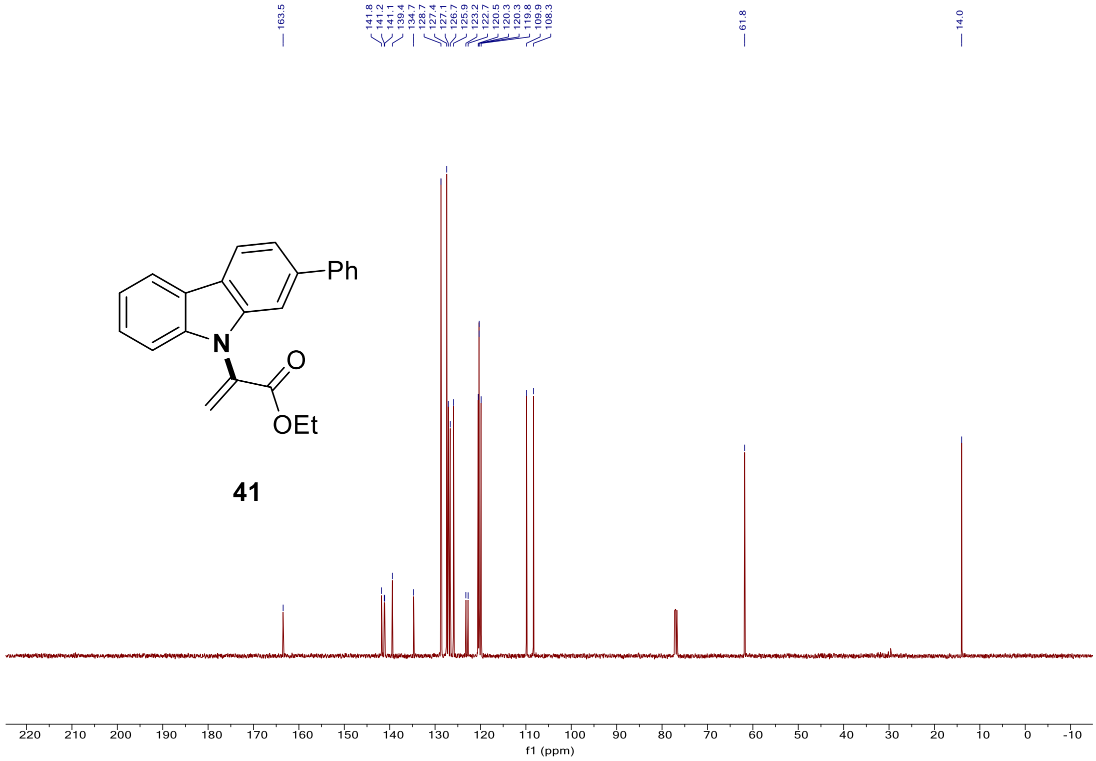

^{13}C NMR spectrum (CDCl$_3$, 126 MHz) of (**41**)

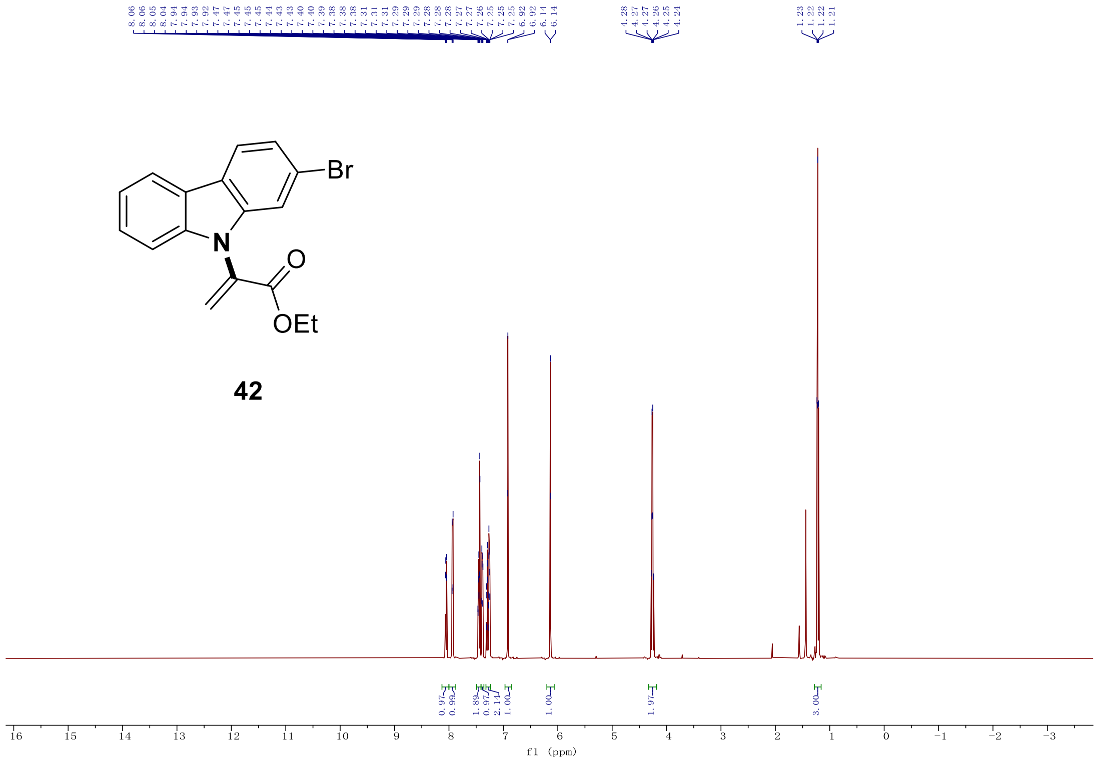

^{1}H NMR spectrum (CDCl3, 500 MHz) of (**42**)

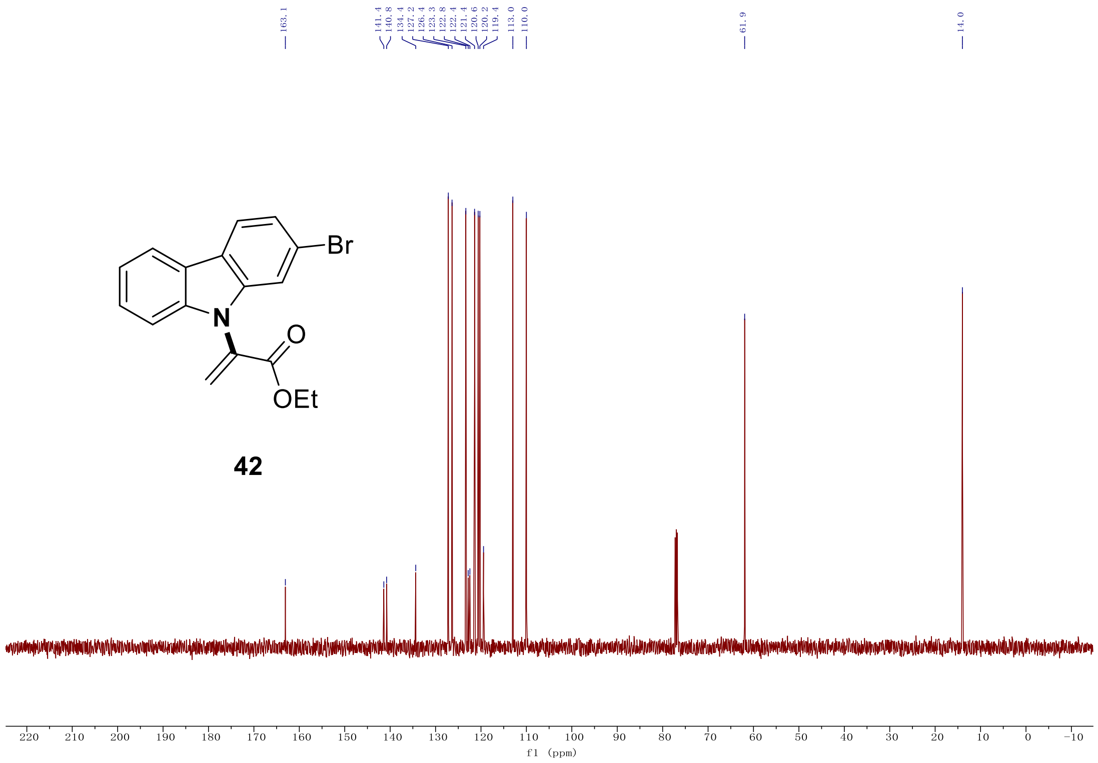

^{13}C NMR spectrum (CDCl3, 126 MHz) of (**42**)

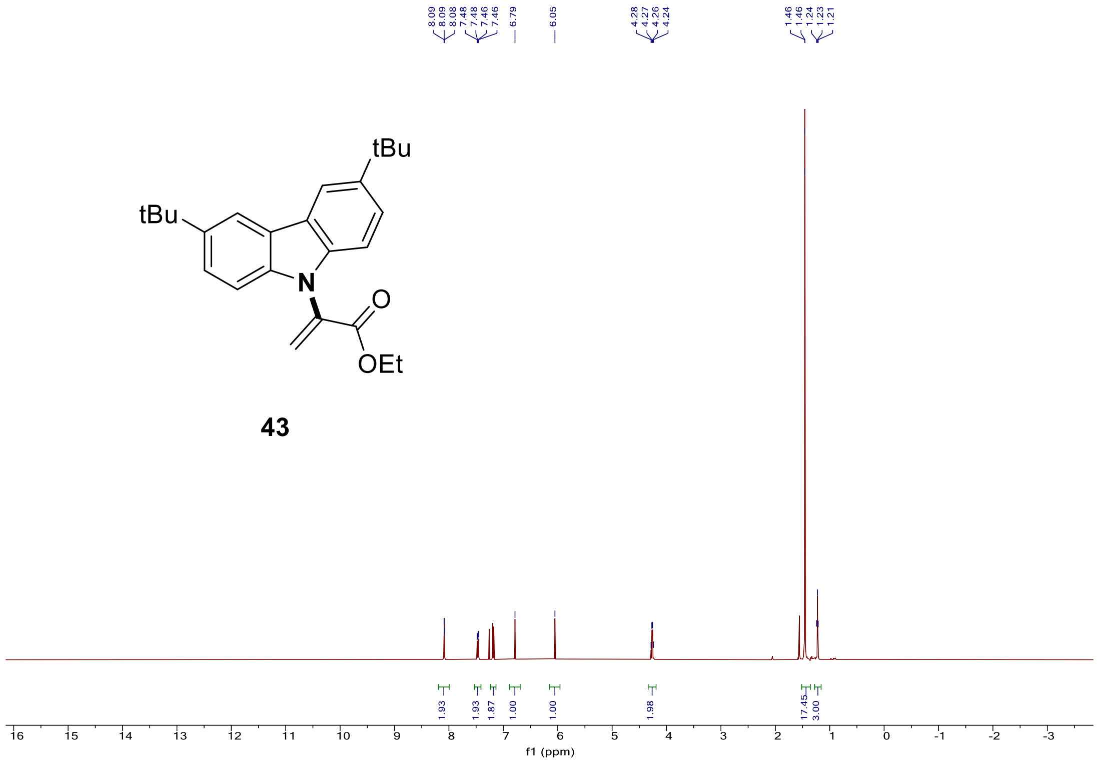

43

^{1}H NMR spectrum ($CDCl_3$, 500 MHz) of (**43**)

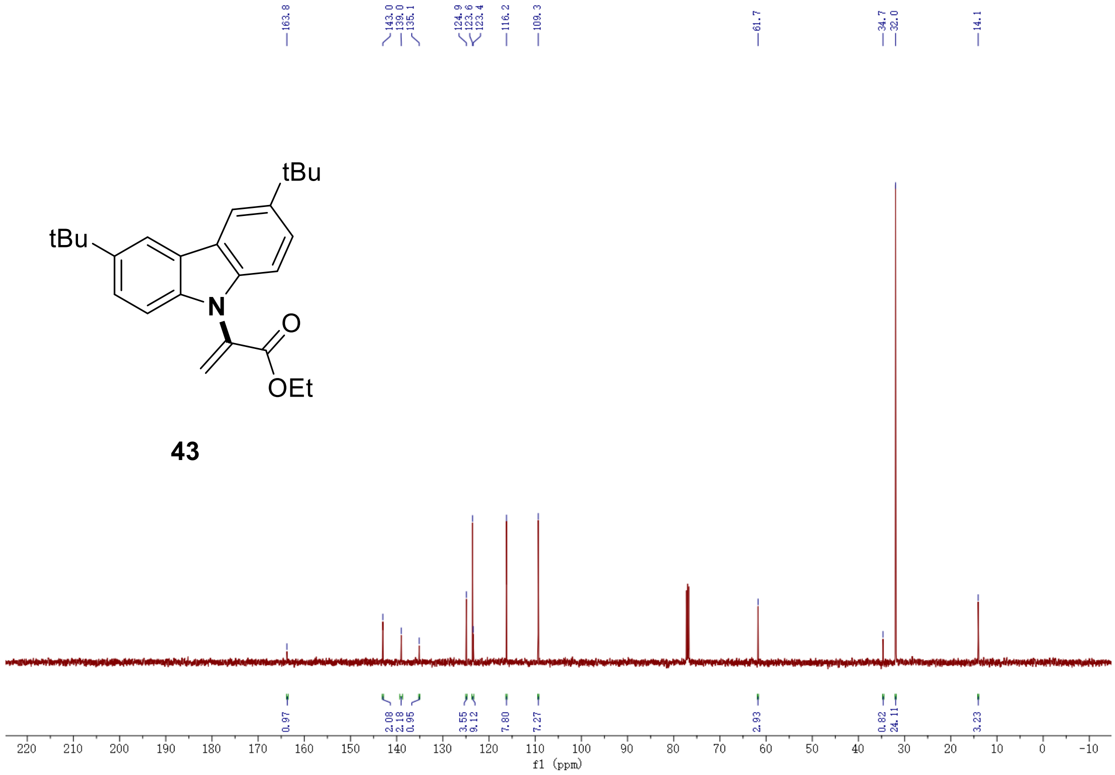

43

^{13}C NMR spectrum ($CDCl_3$, 126 MHz) of (**43**)

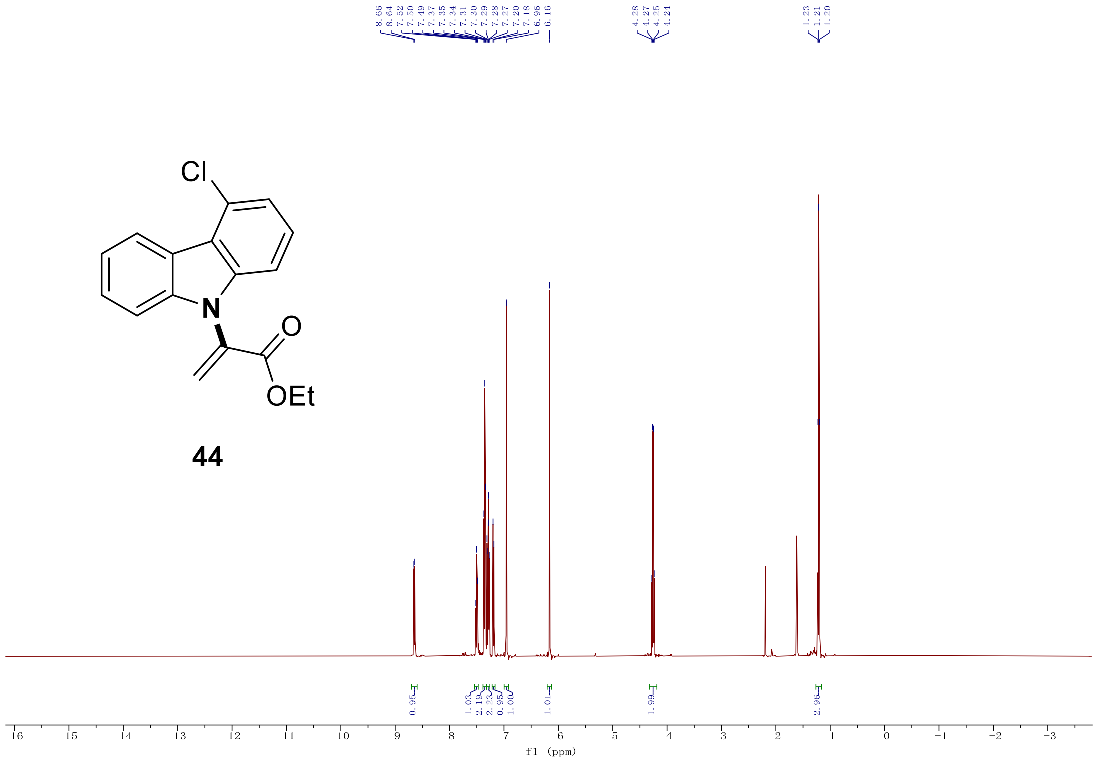

^{1}H NMR spectrum ($CDCl_3$, 500 MHz) of (**44**)

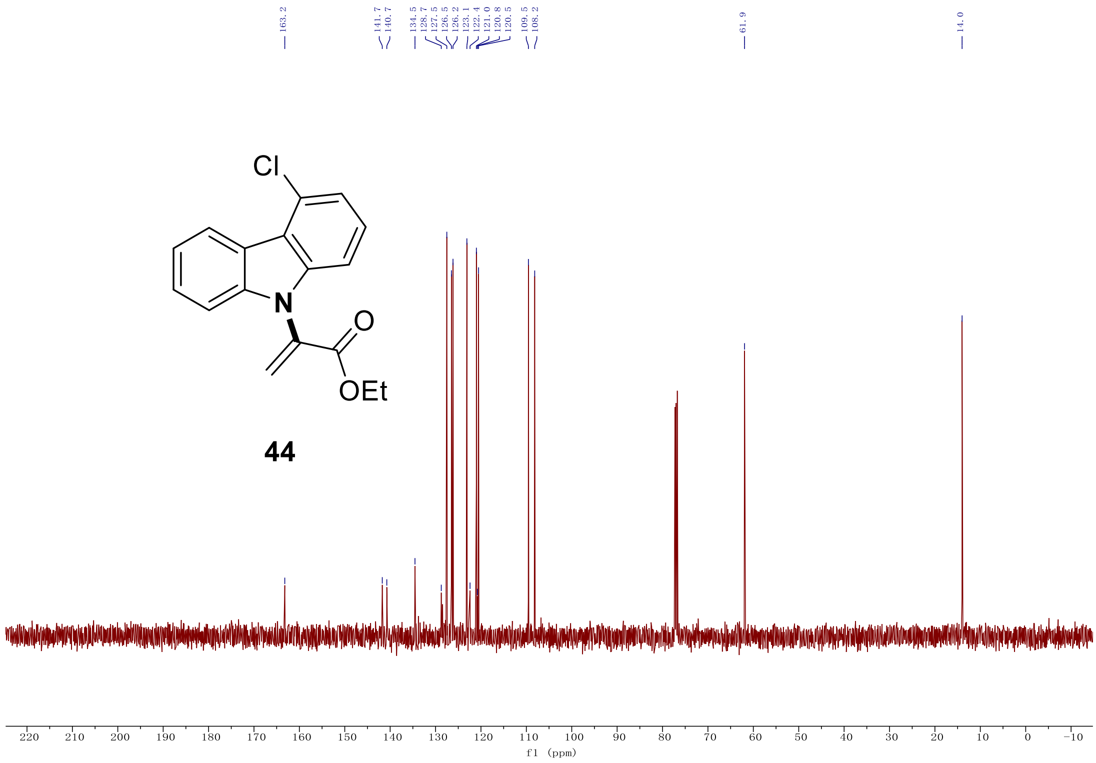

^{13}C NMR spectrum ($CDCl_3$, 126 MHz) of (**44**)

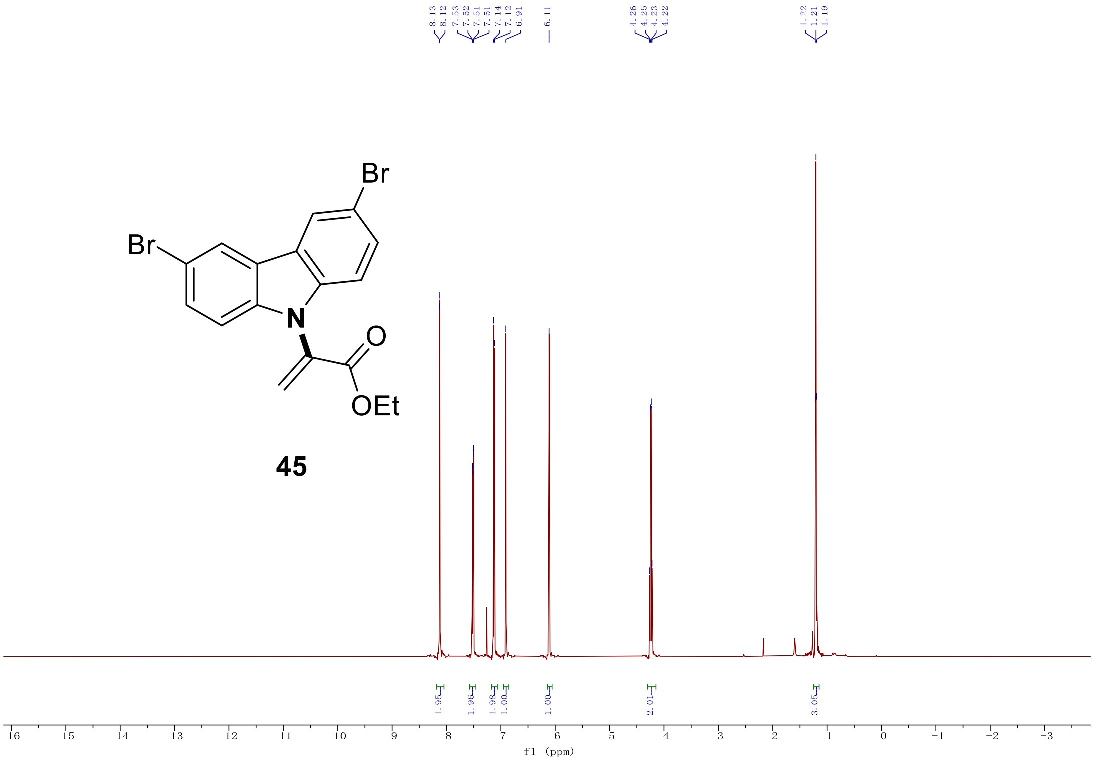

^{1}H NMR spectrum ($CDCl_3$, 500 MHz) of (**45**)

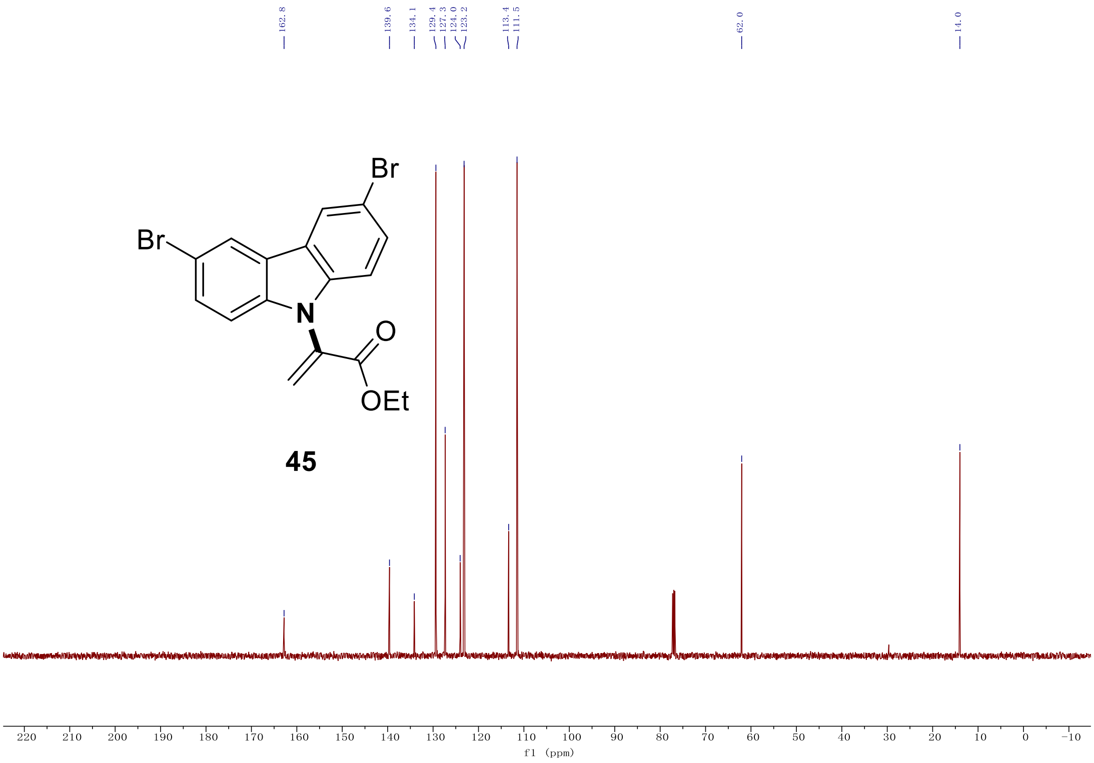

^{13}C NMR spectrum ($CDCl_3$, 126 MHz) of (**45**)

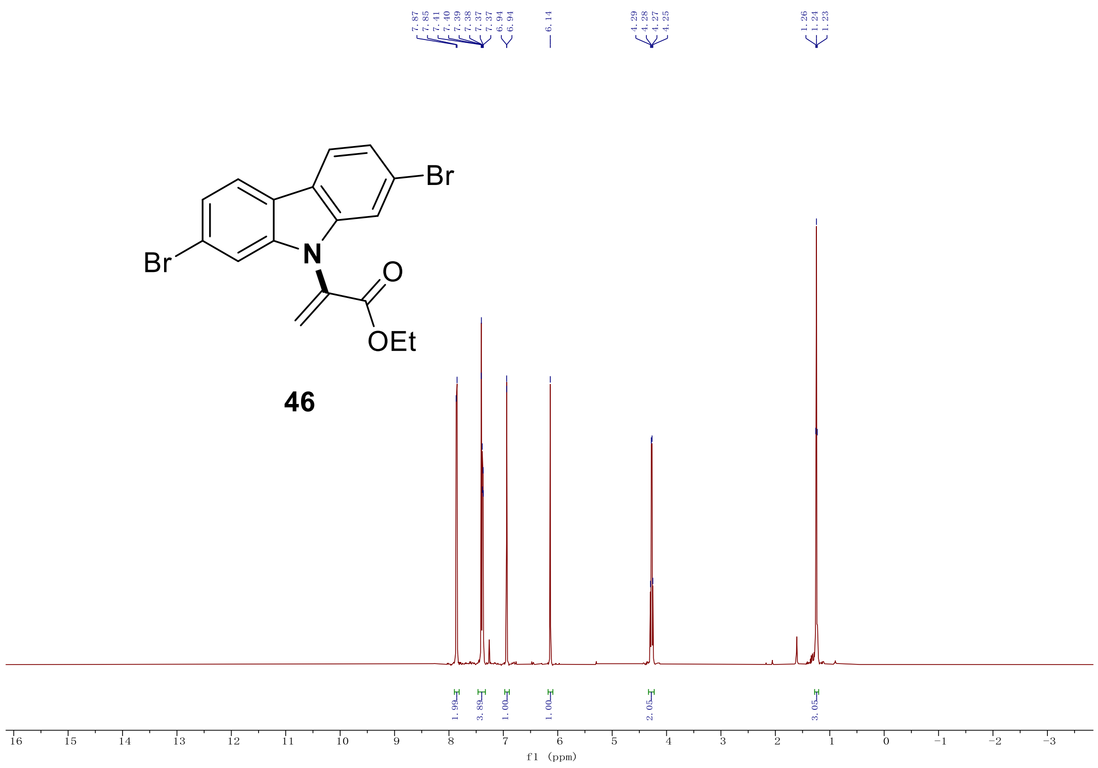

^{1}H NMR spectrum ($CDCl_3$, 500 MHz) of (**46**)

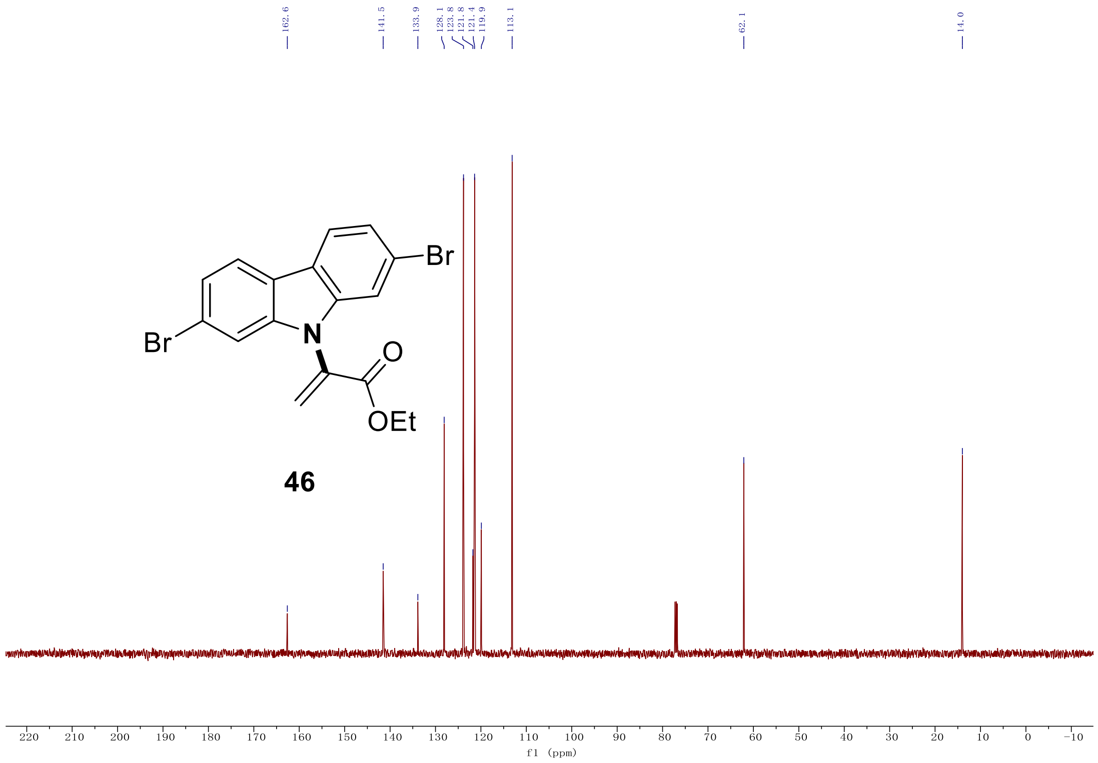

^{13}C NMR spectrum ($CDCl_3$, 126 MHz) of (**46**)

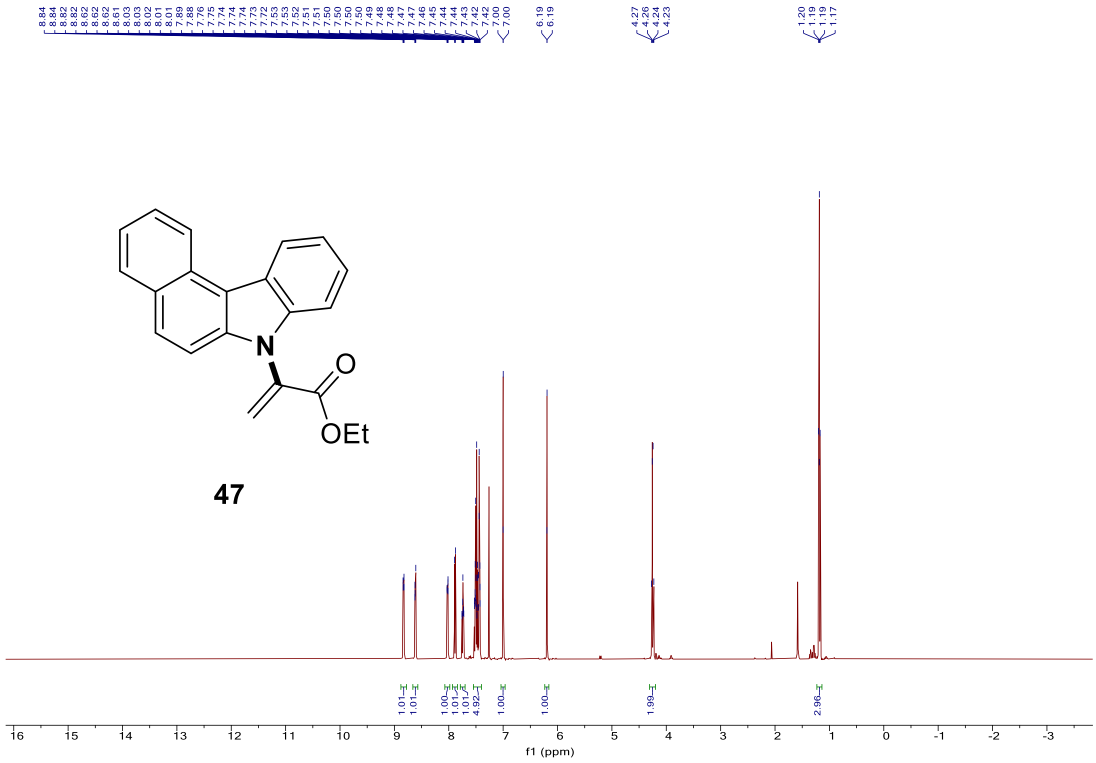

^{1}H NMR spectrum (CDCl$_3$, 500 MHz) of (**47**)

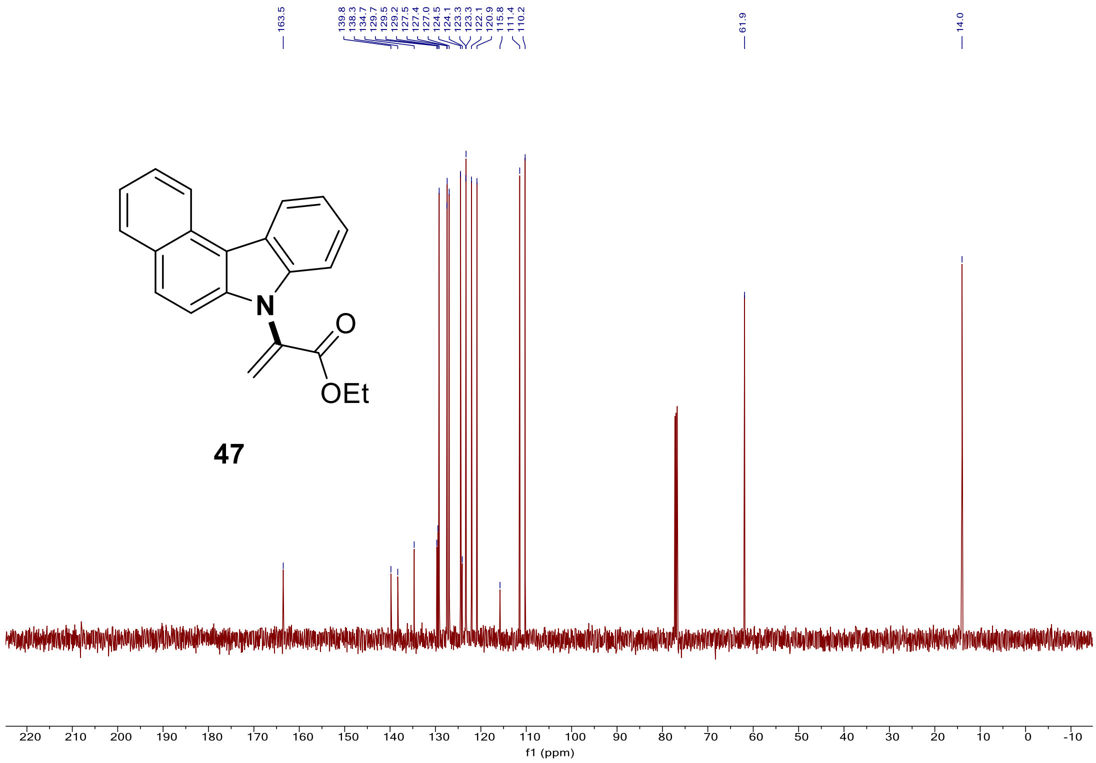

^{13}C NMR spectrum (CDCl$_3$, 126 MHz) of (**47**)

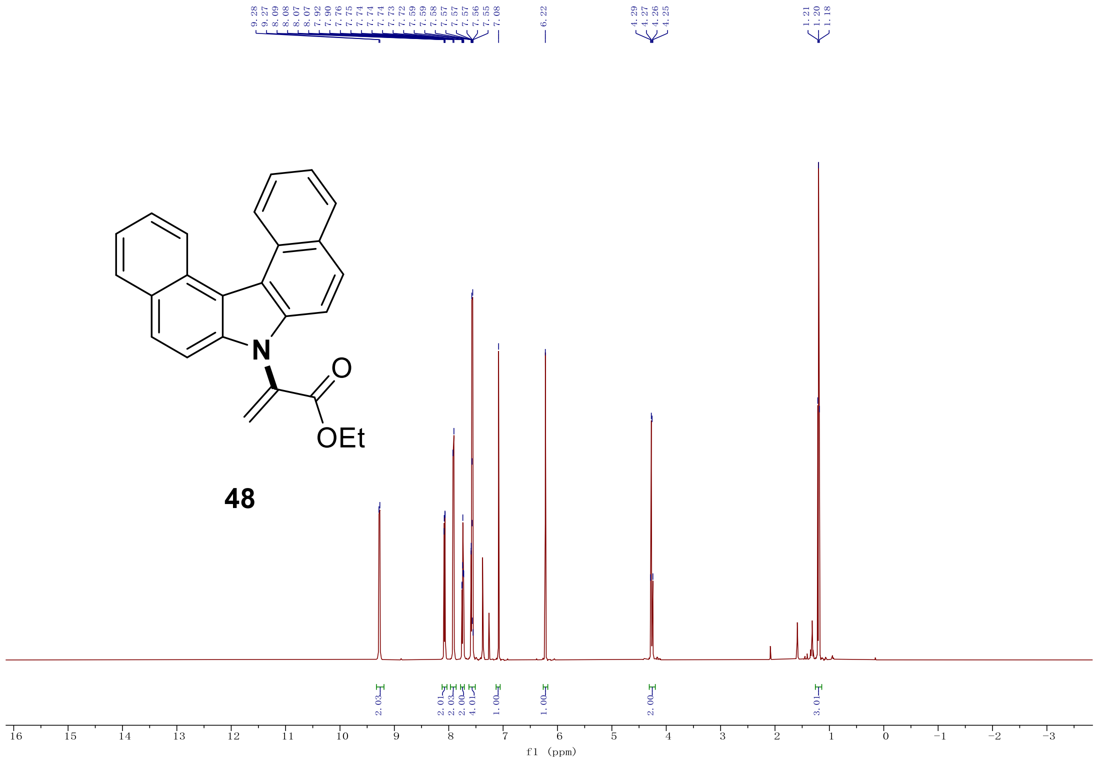

^{1}H NMR spectrum (CDCl3, 500 MHz) of (**48**)

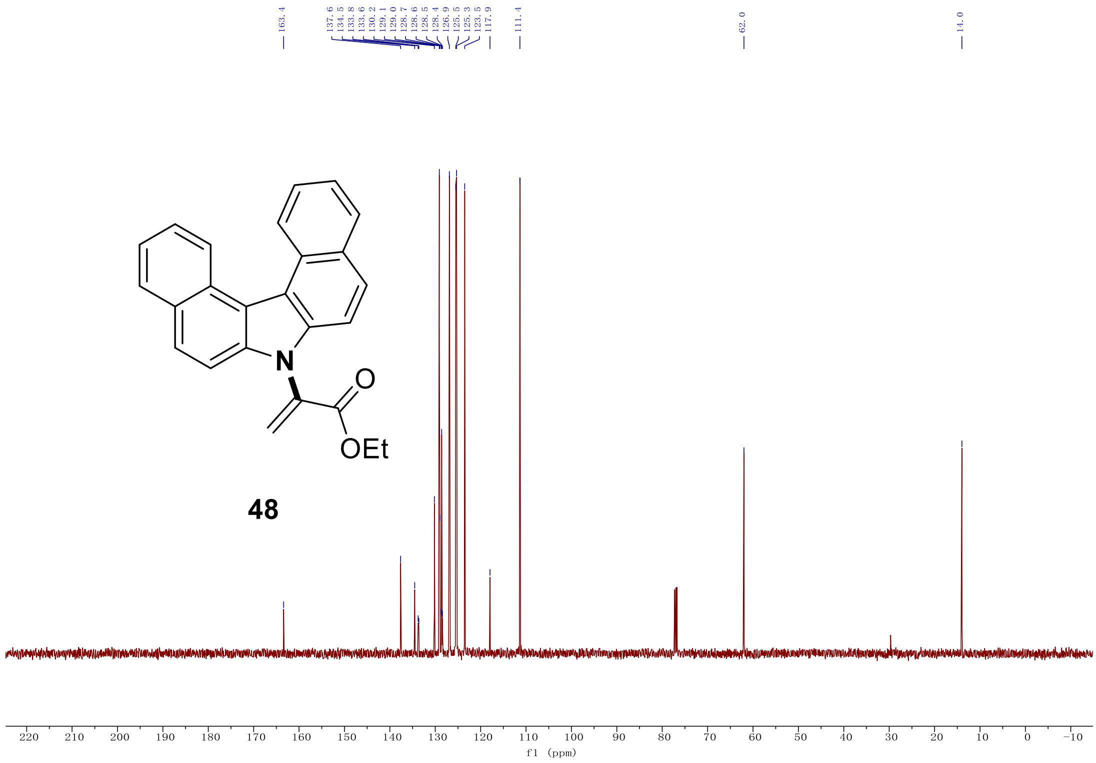

^{13}C NMR spectrum (CDCl3, 126 MHz) of (**48**)

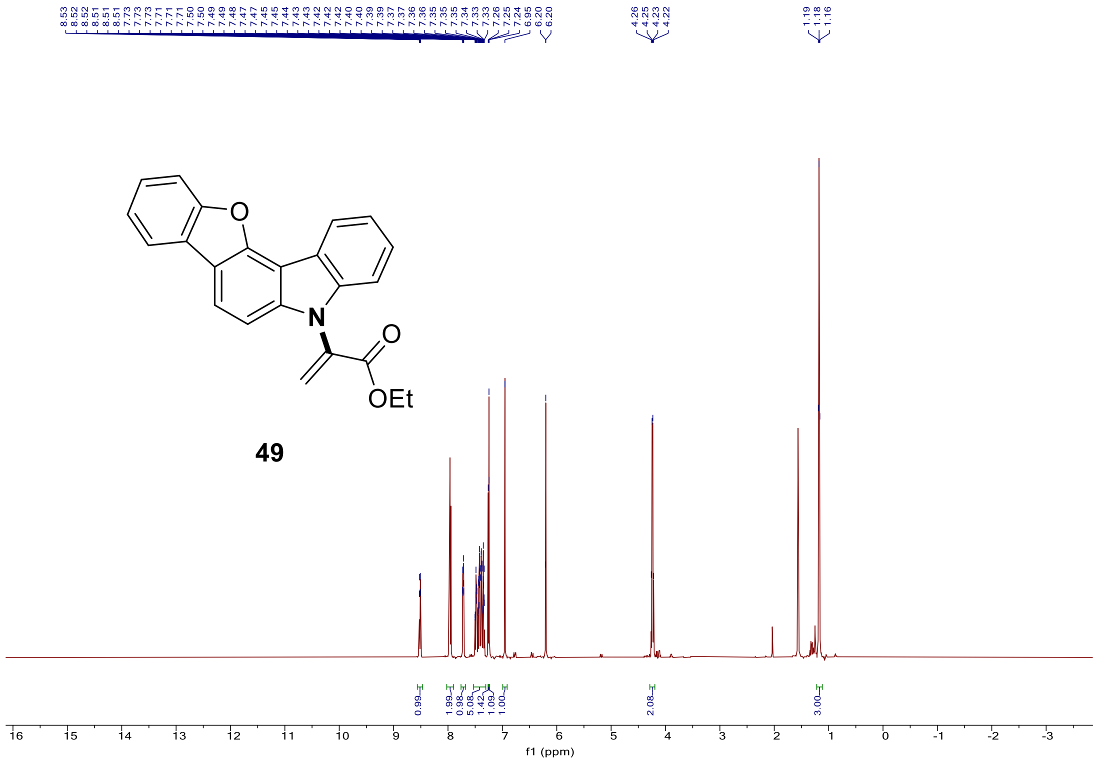

^{1}H NMR spectrum (CDCl₃, 500 MHz) of (**49**)

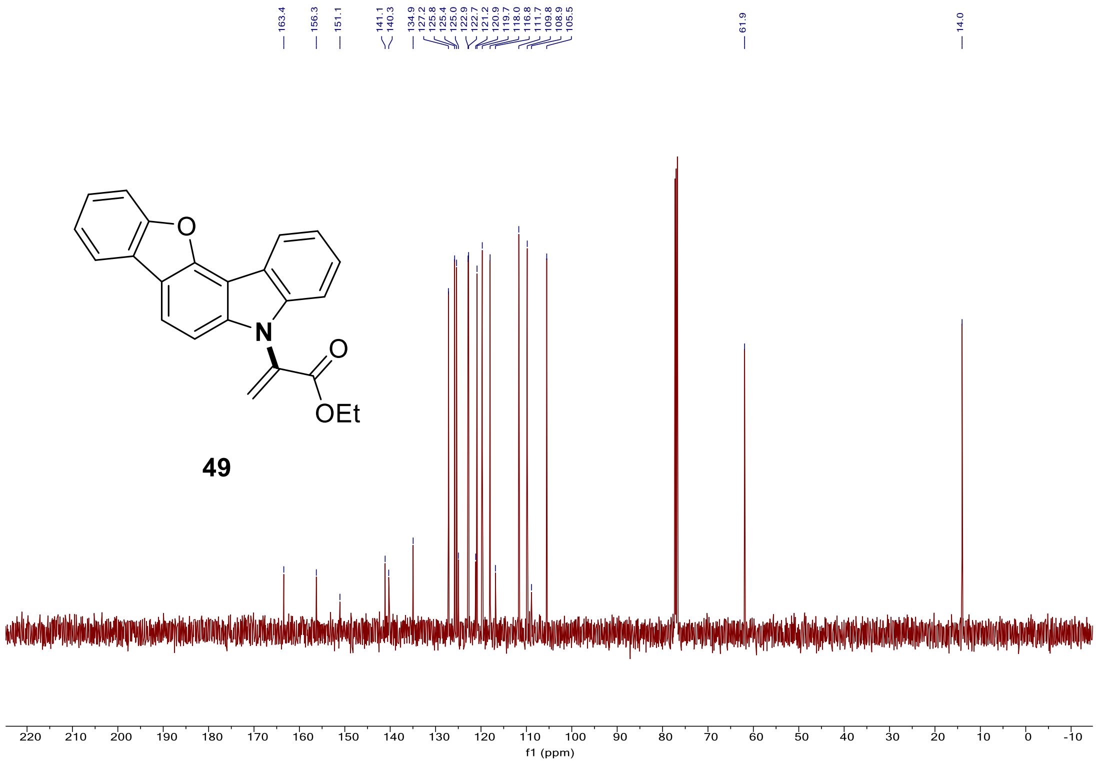

^{13}C NMR spectrum (CDCl₃, 126 MHz) of (**49**)

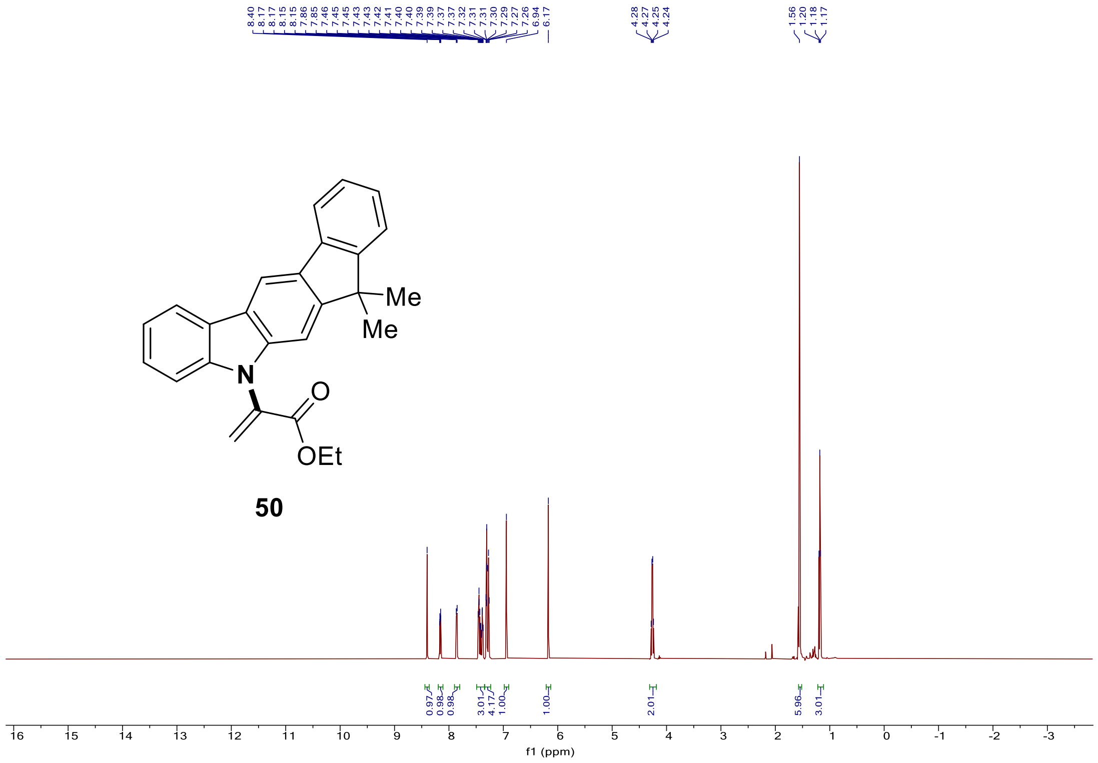

^{1}H NMR spectrum ($CDCl_3$, 500 MHz) of (**50**)

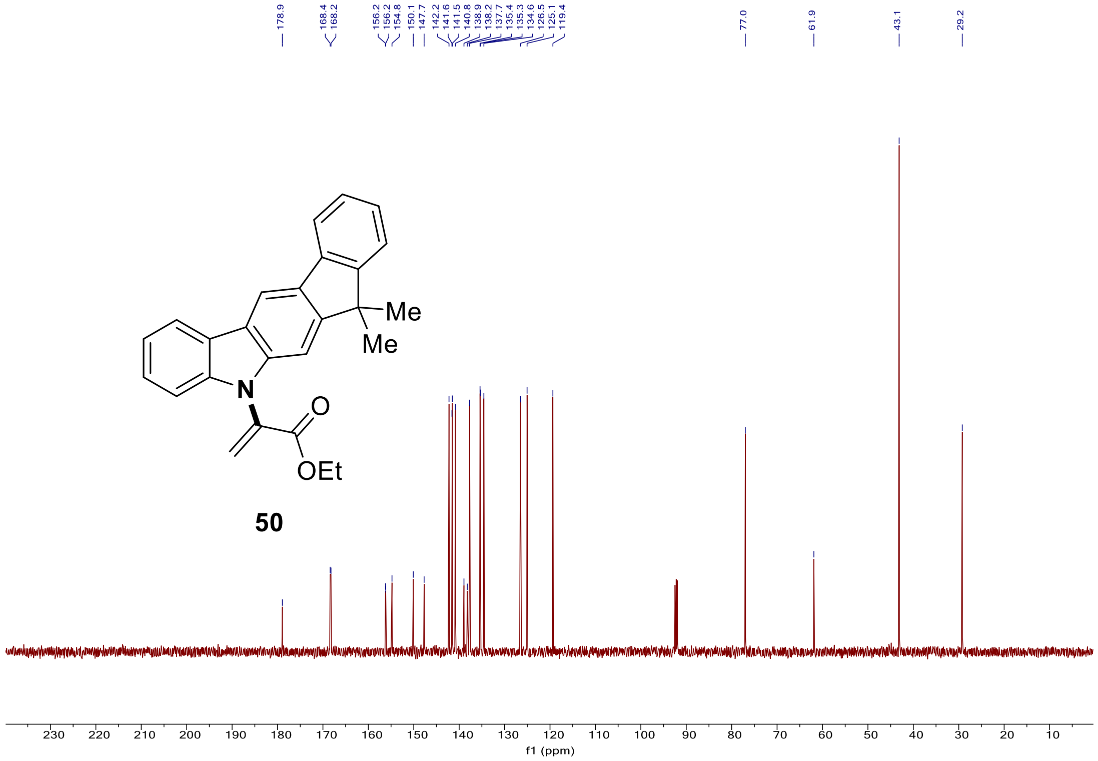

^{13}C NMR spectrum ($CDCl_3$, 126 MHz) of (**50**)

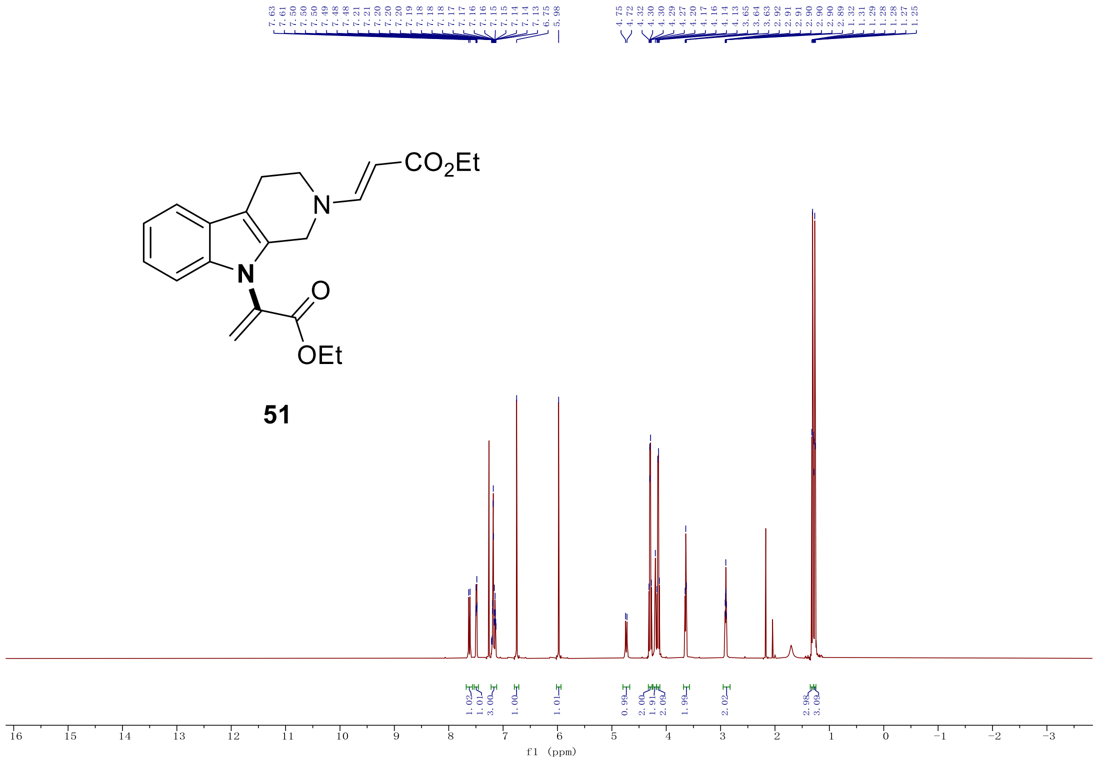

^{1}H NMR spectrum (CDCl$_3$, 500 MHz) of (**51**)

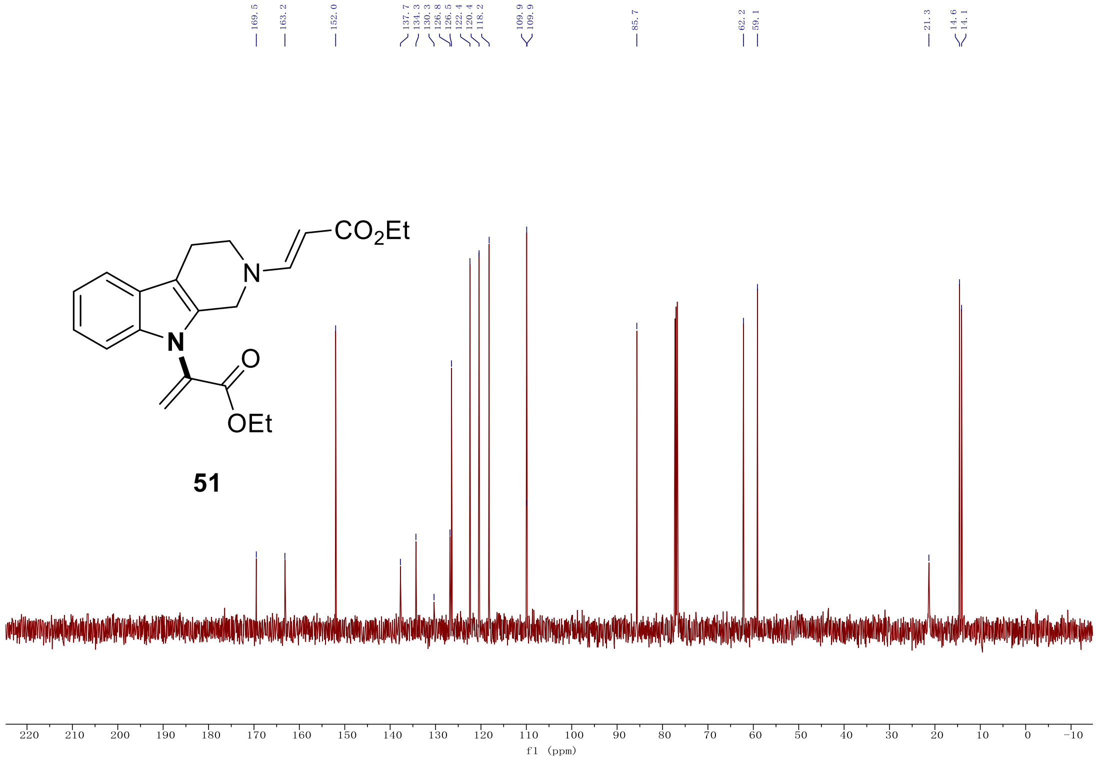

^{13}C NMR spectrum (CDCl$_3$, 126 MHz) of (**51**)

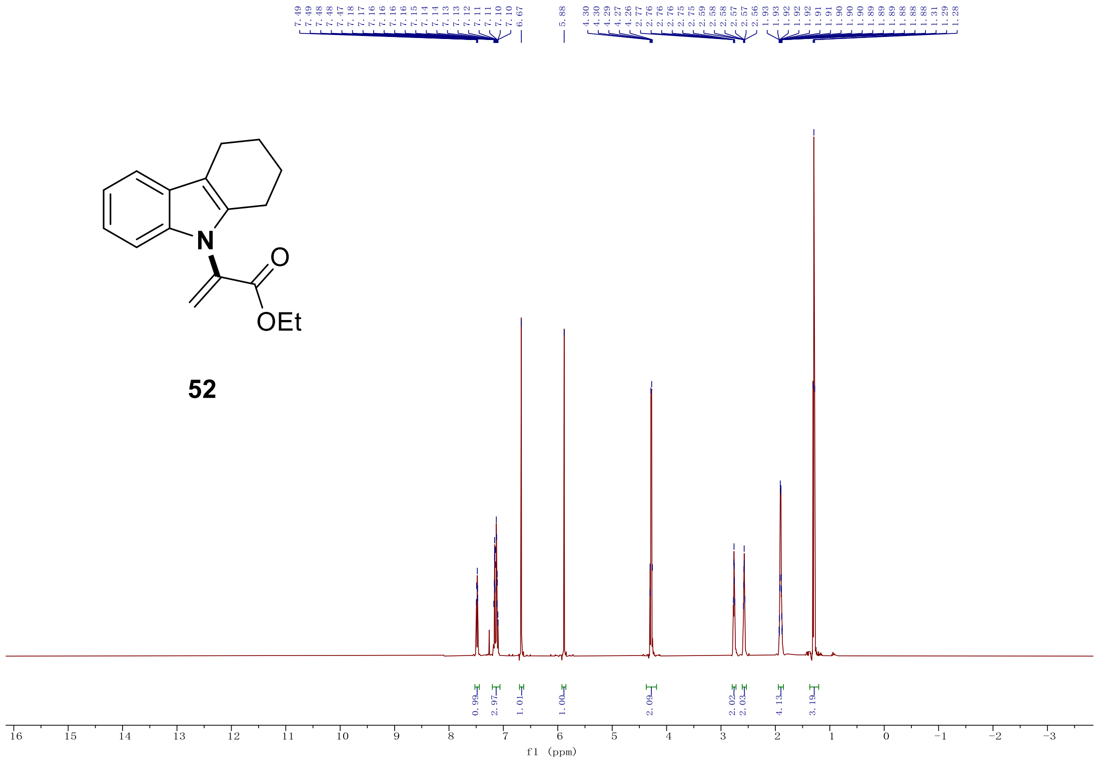

^{1}H NMR spectrum (CDCl$_3$, 500 MHz) of (**52**)

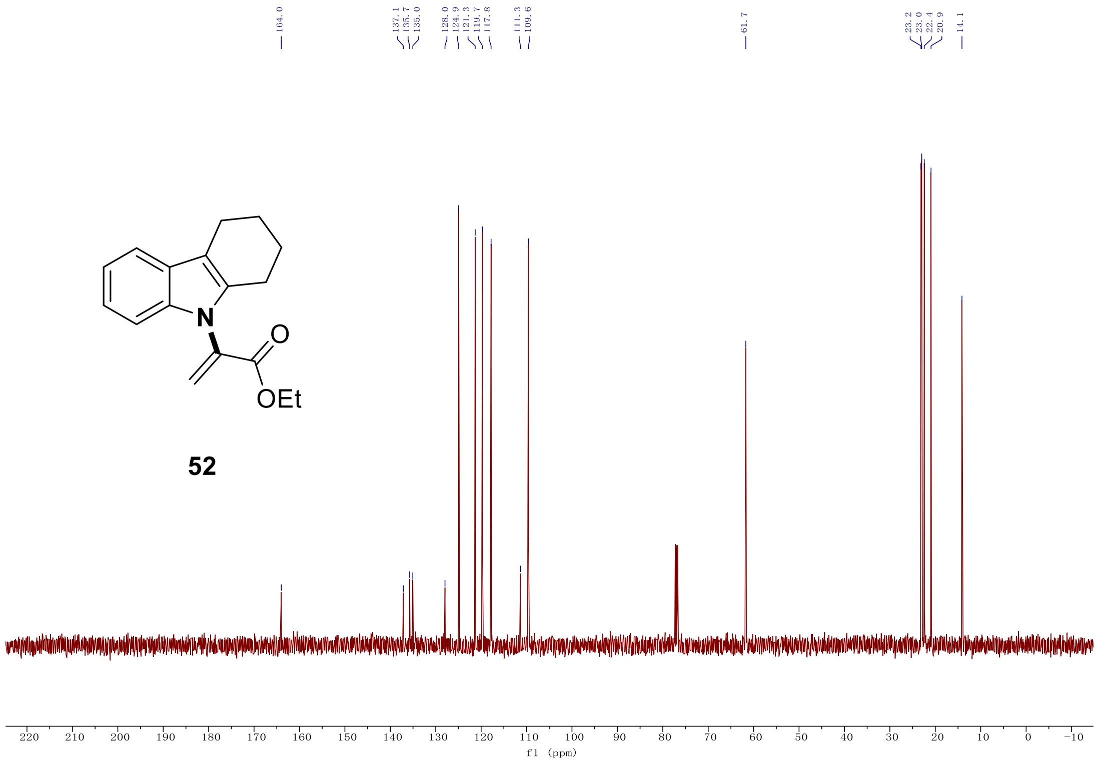

^{13}C NMR spectrum (CDCl$_3$, 126 MHz) of (**52**)

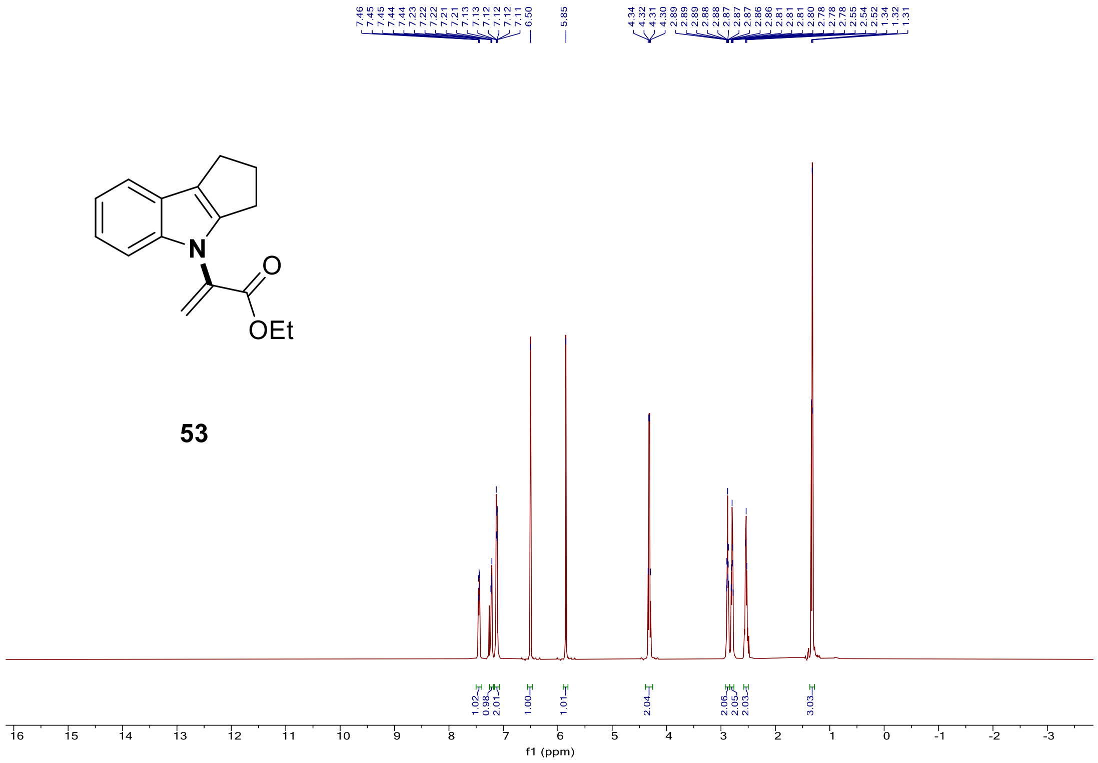

^{1}H NMR spectrum (CDCl$_3$, 500 MHz) of (**53**)

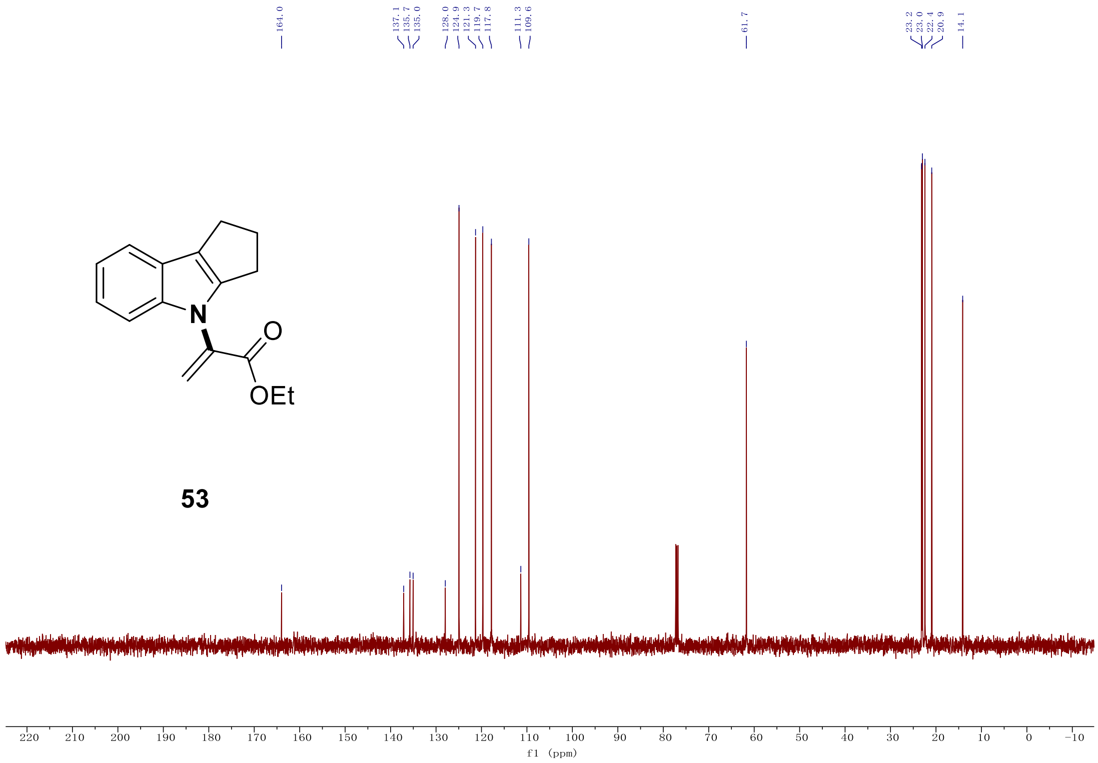

^{13}C NMR spectrum (CDCl$_3$, 126 MHz) of (**53**)

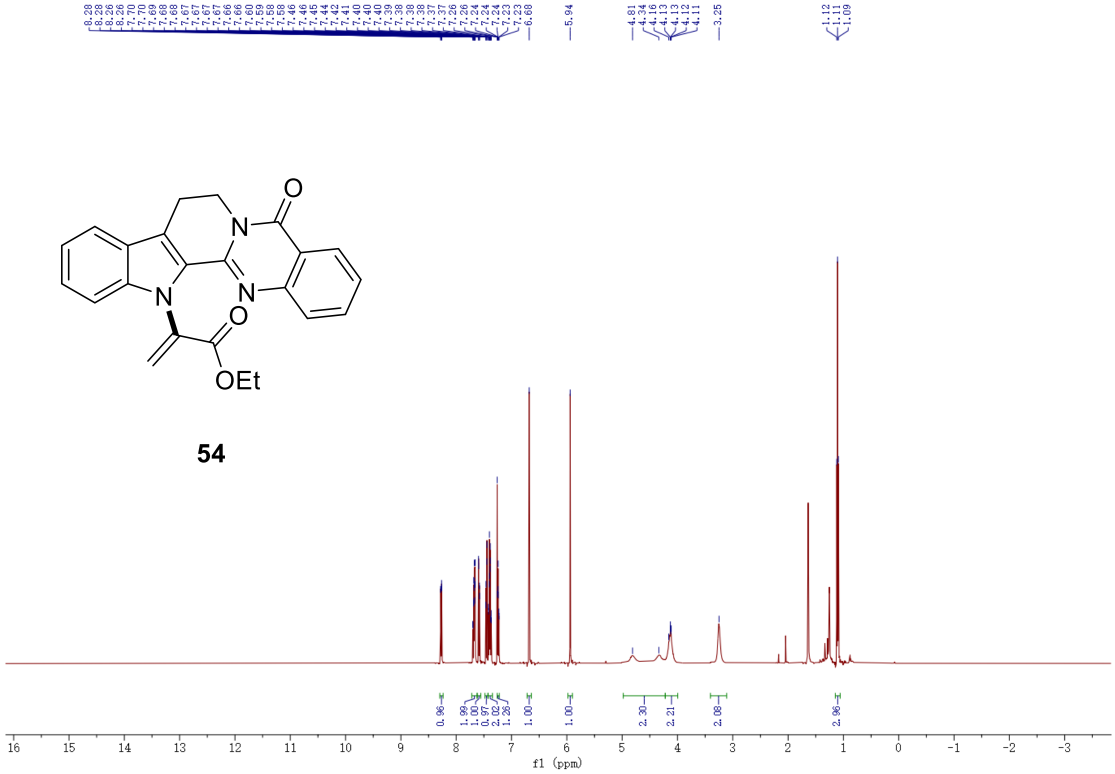

^{1}H NMR spectrum ($CDCl_3$, 500 MHz) of (**54**)

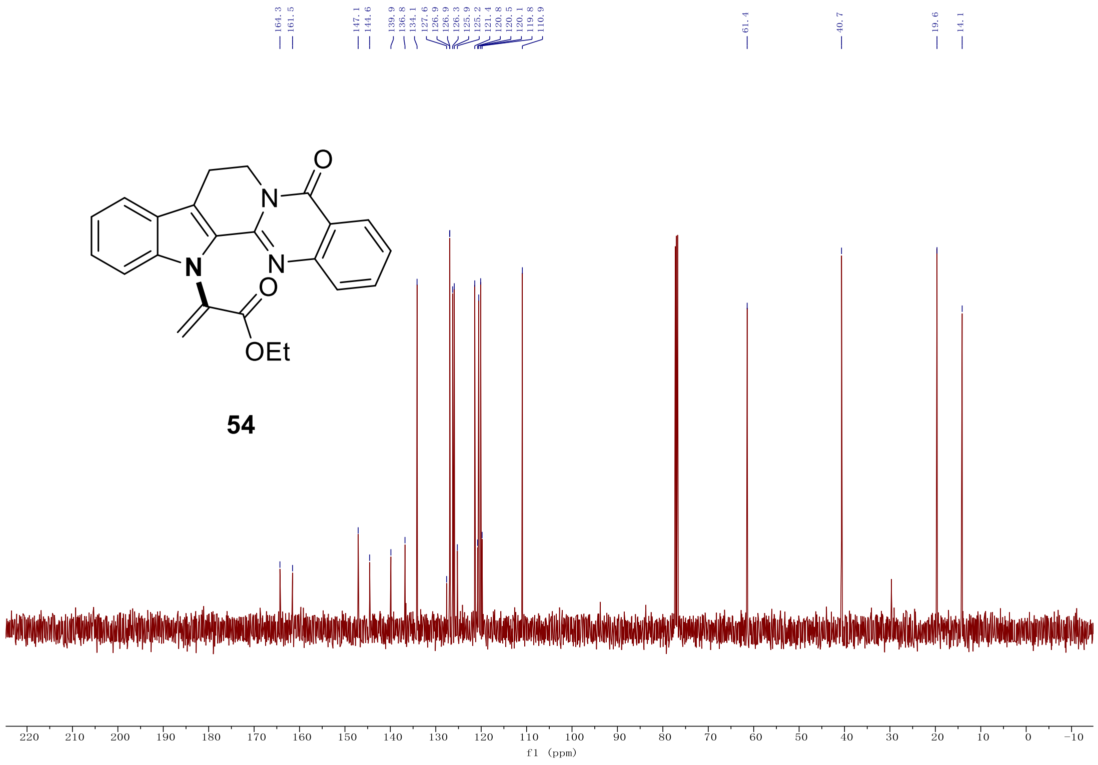

^{13}C NMR spectrum ($CDCl_3$, 126 MHz) of (**54**)

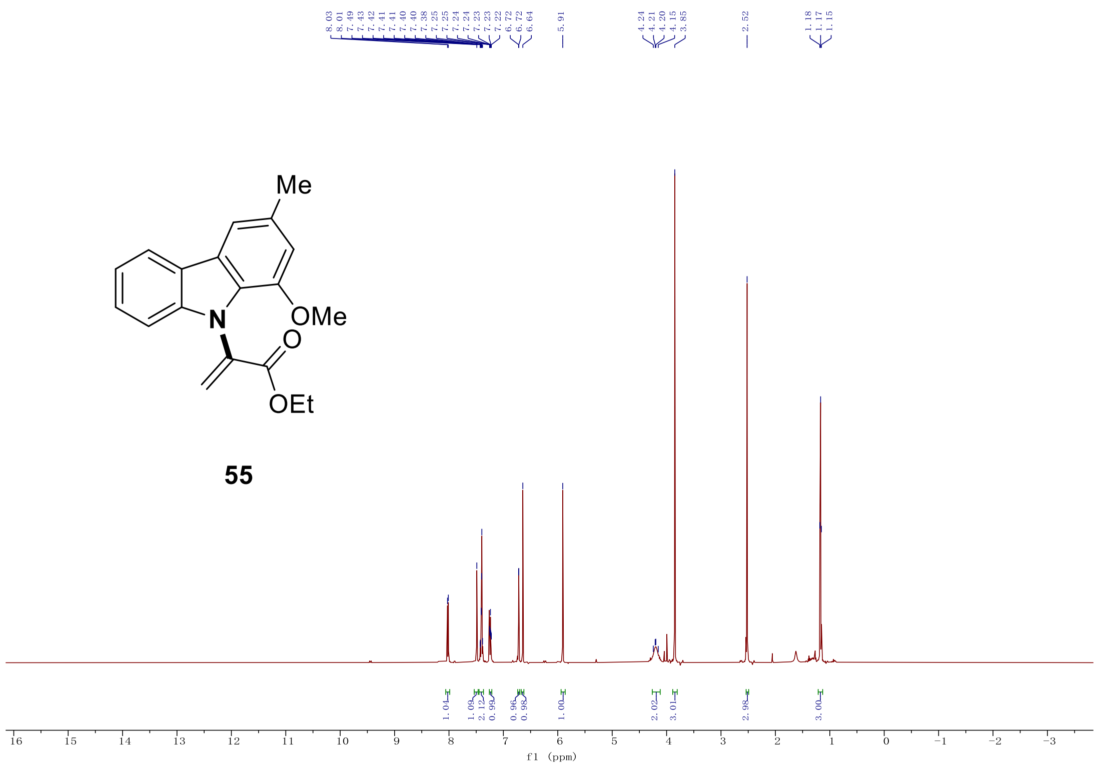

^{1}H NMR spectrum ($CDCl_3$, 500 MHz) of (**55**)

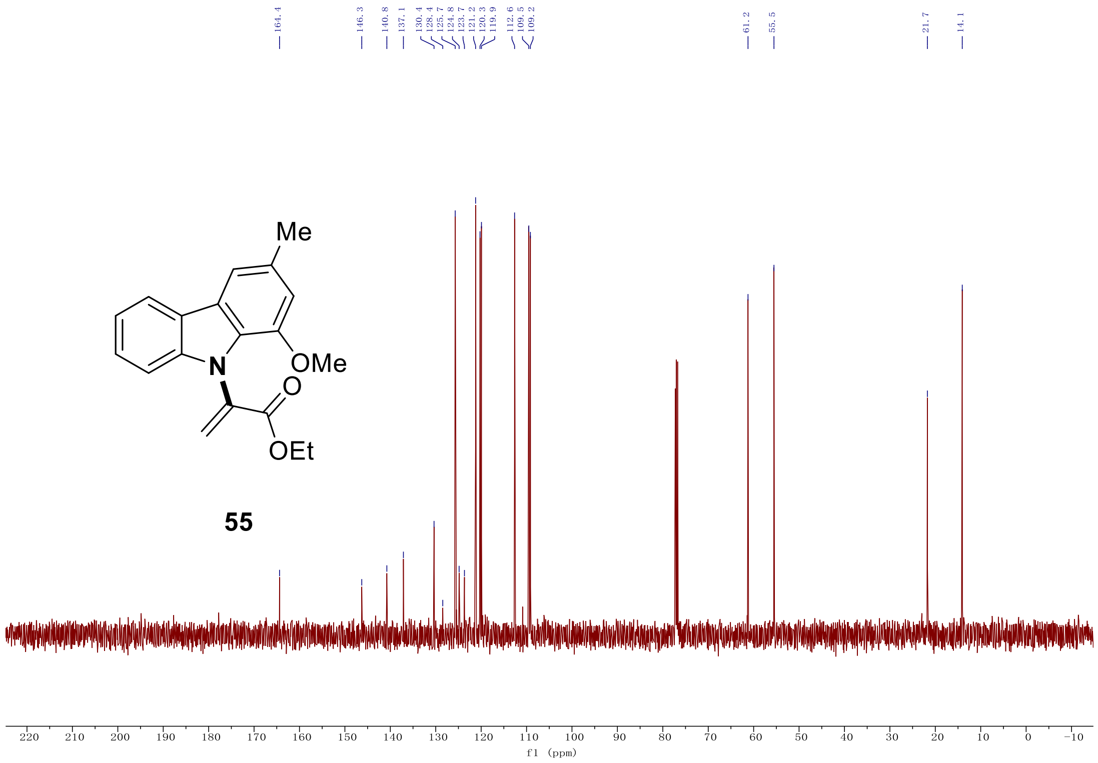

^{13}C NMR spectrum ($CDCl_3$, 126 MHz) of (**55**)

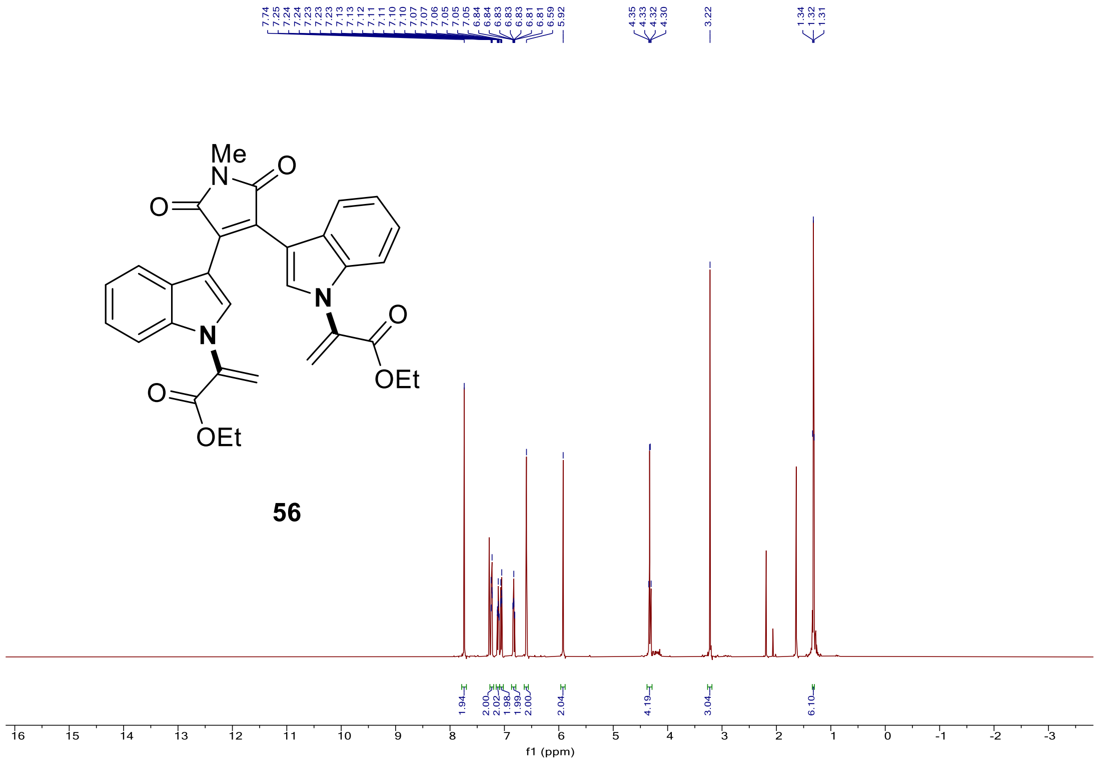

^{1}H NMR spectrum ($CDCl_3$, 500 MHz) of (**56**)

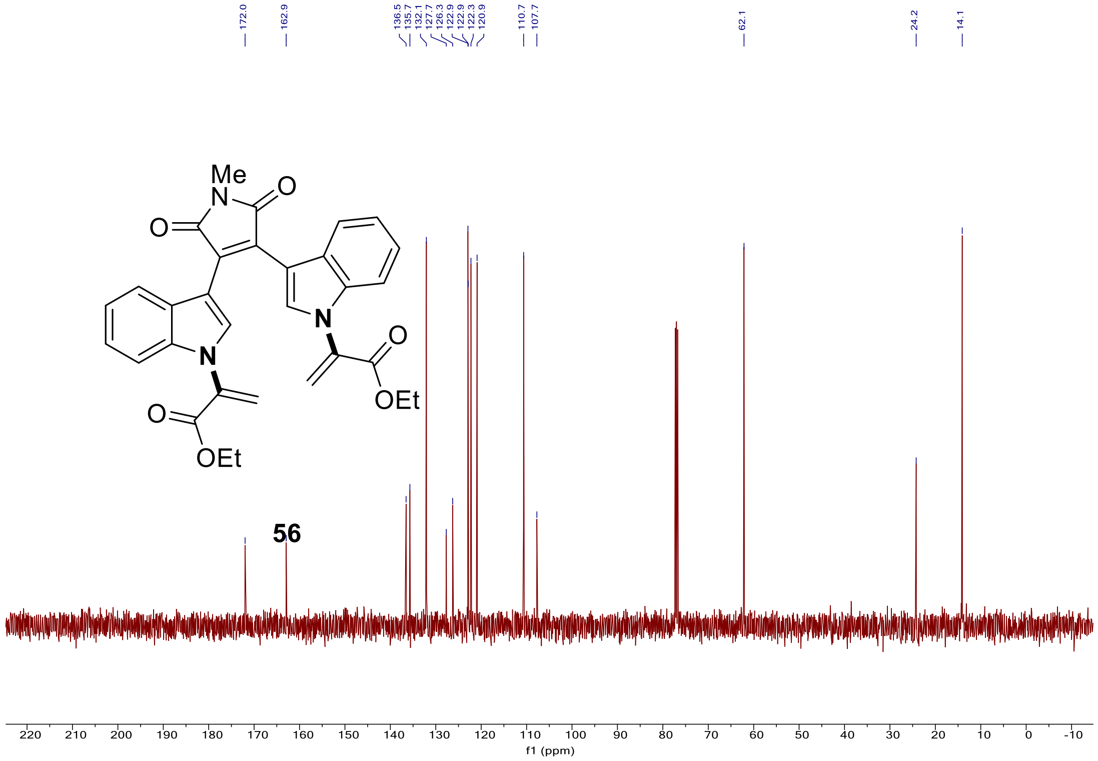

^{13}C NMR spectrum ($CDCl_3$, 126 MHz) of (**56**)

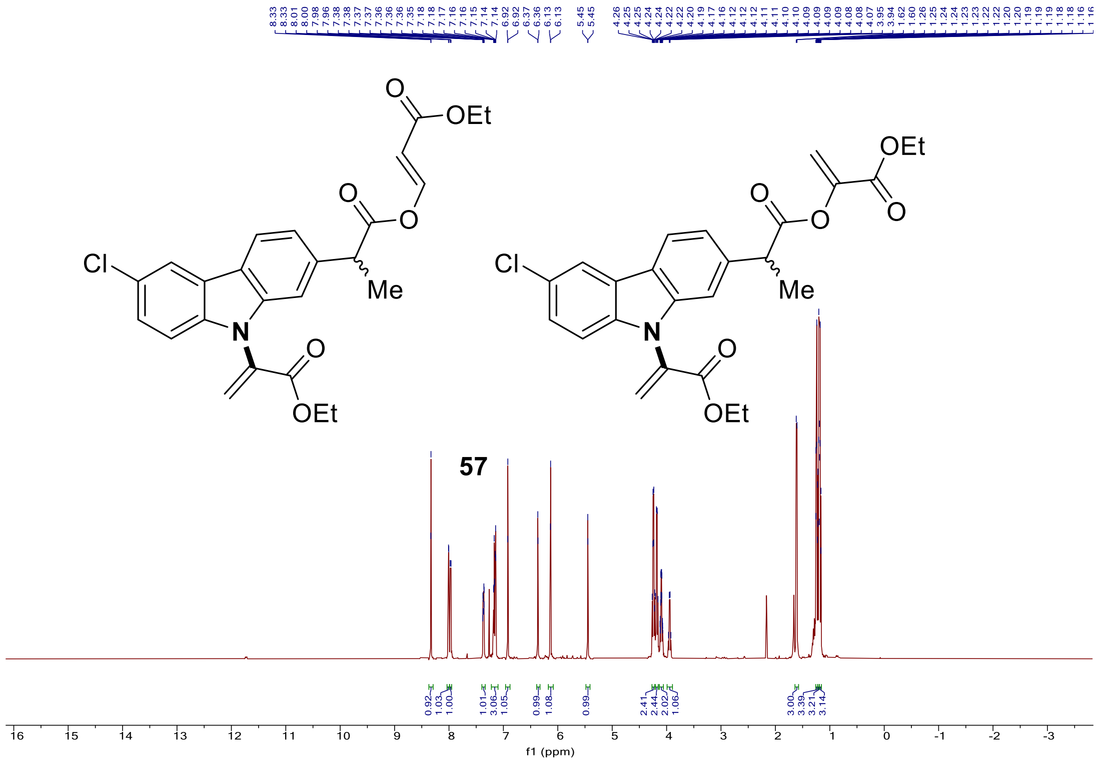

^{1}H NMR spectrum ($CDCl_3$, 500 MHz) of (**57**)

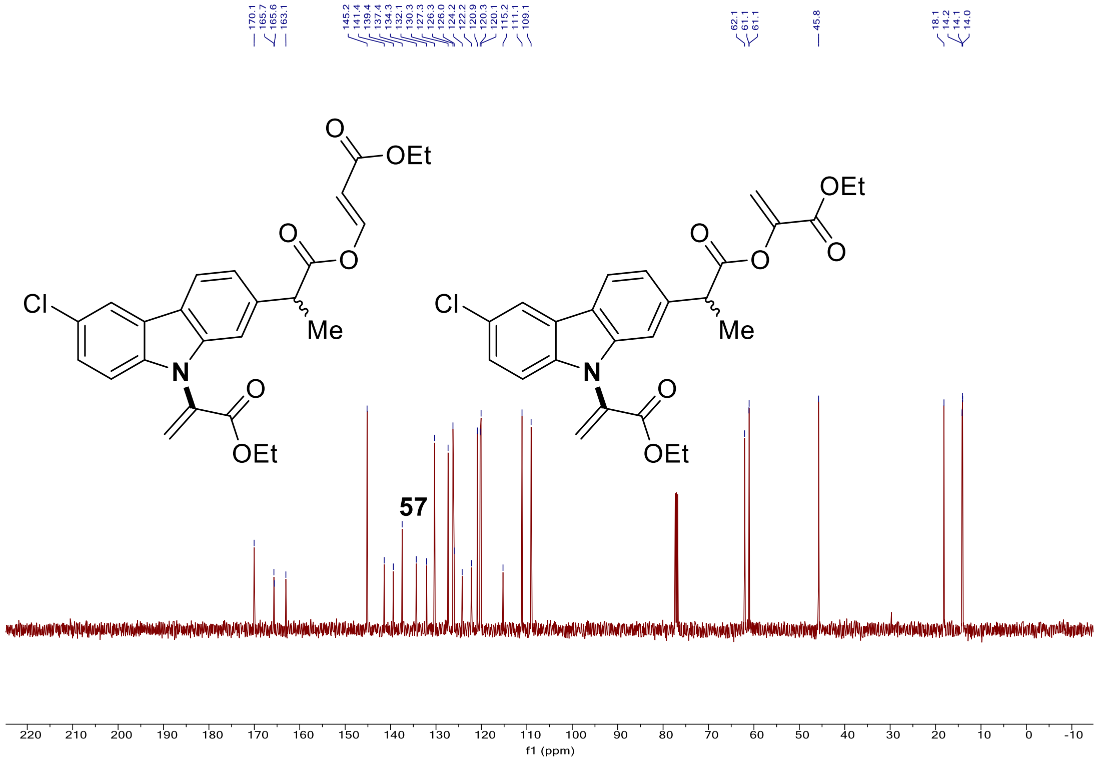

^{13}C NMR spectrum ($CDCl_3$, 126 MHz) of (**57**)

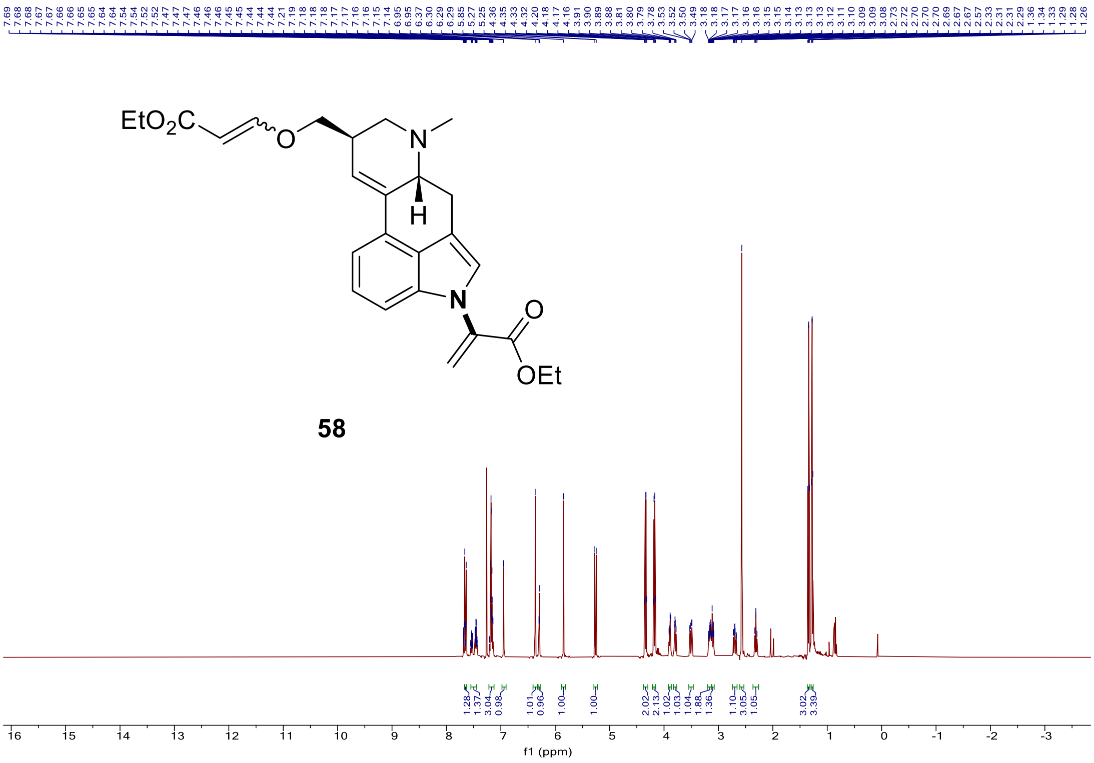

^{1}H NMR spectrum ($CDCl_3$, 500 MHz) of (**58**)

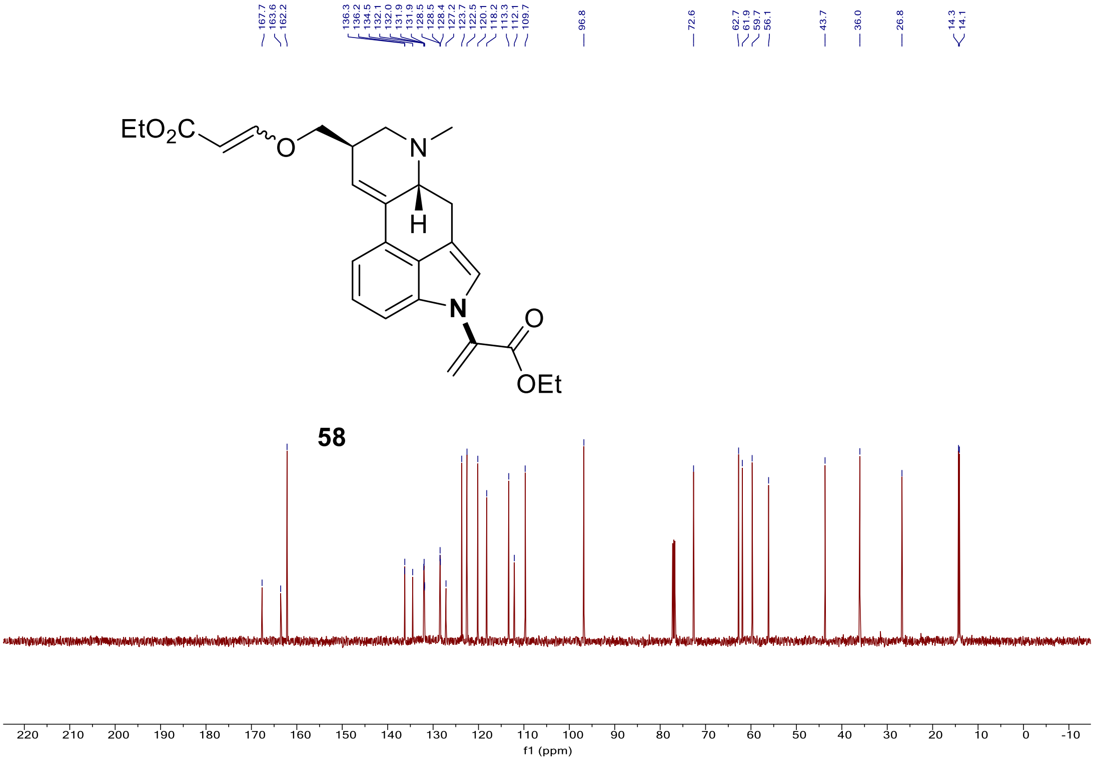

^{13}C NMR spectrum ($CDCl_3$, 126 MHz) of (**58**)

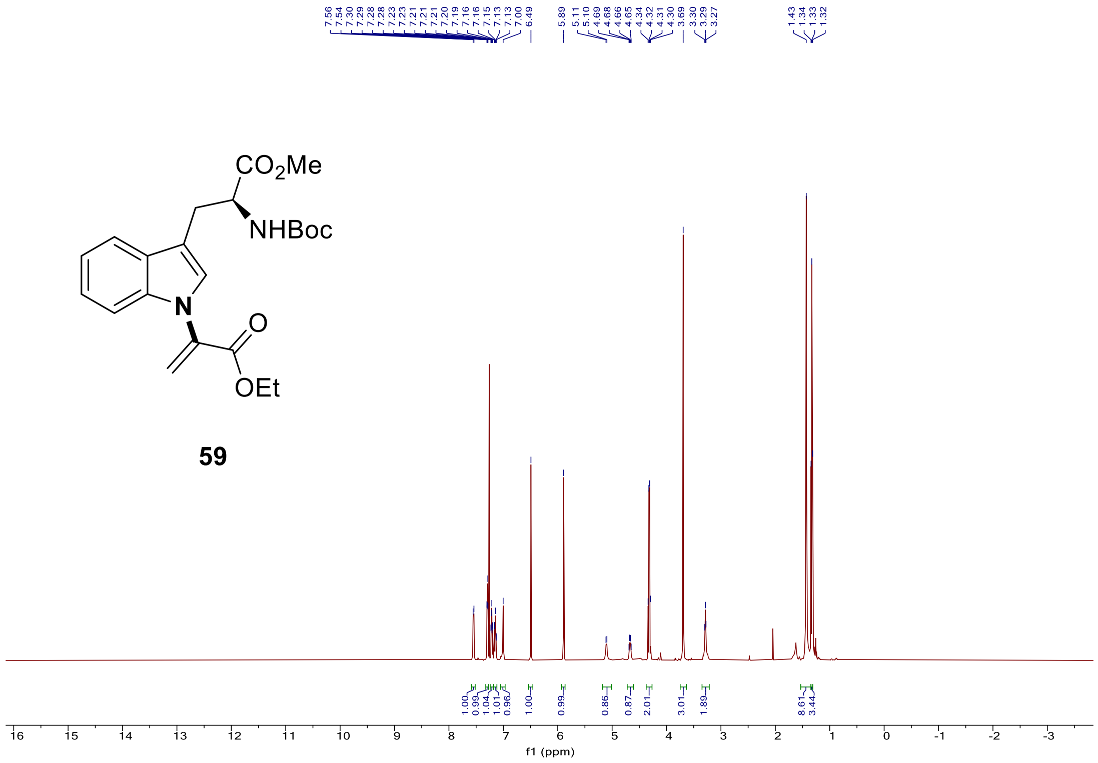

^{1}H NMR spectrum ($CDCl_3$, 500 MHz) of (**59**)

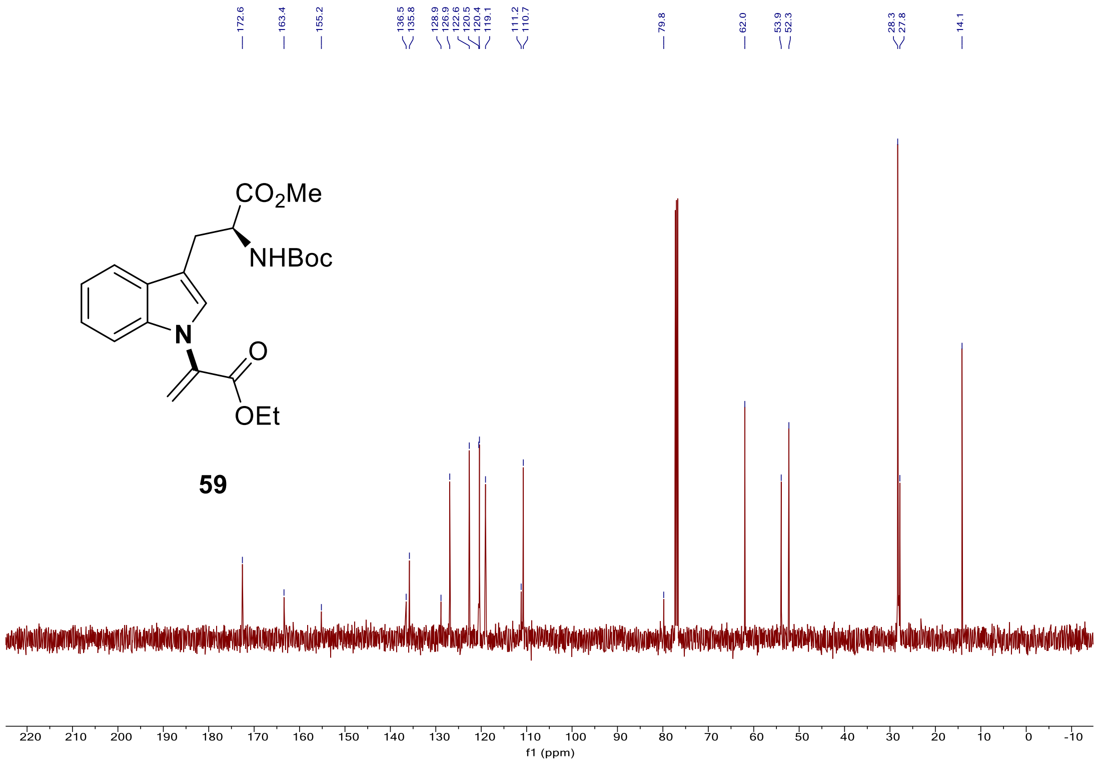

^{13}C NMR spectrum ($CDCl_3$, 126 MHz) of (**59**)

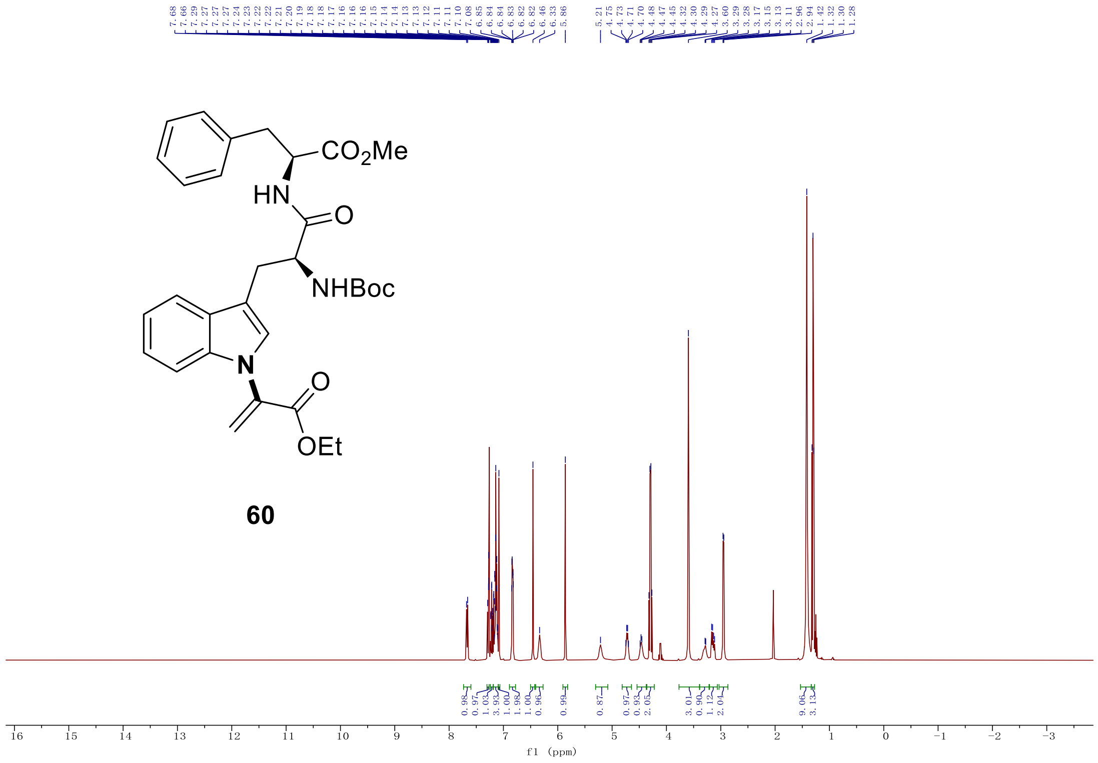

^{1}H NMR spectrum ($CDCl_3$, 400 MHz) of (**60**)

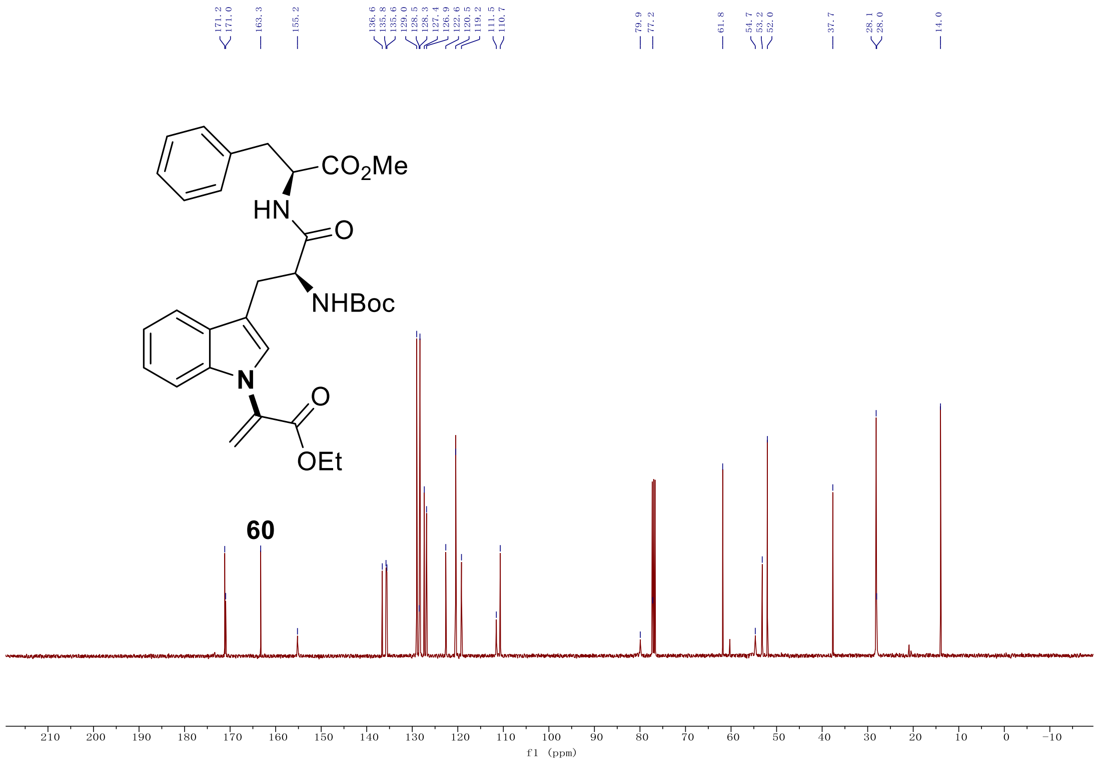

^{13}C NMR spectrum ($CDCl_3$, 101 MHz) of (**60**)

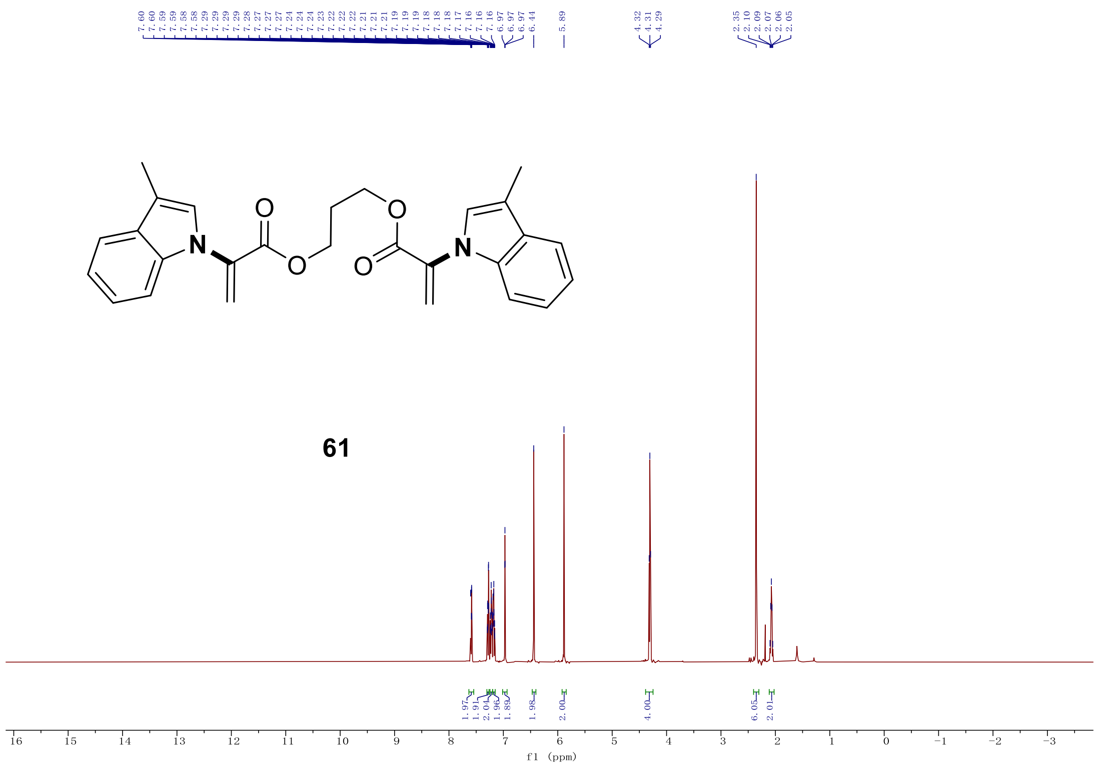

^{1}H NMR spectrum ($CDCl_3$, 500 MHz) of (**61**)

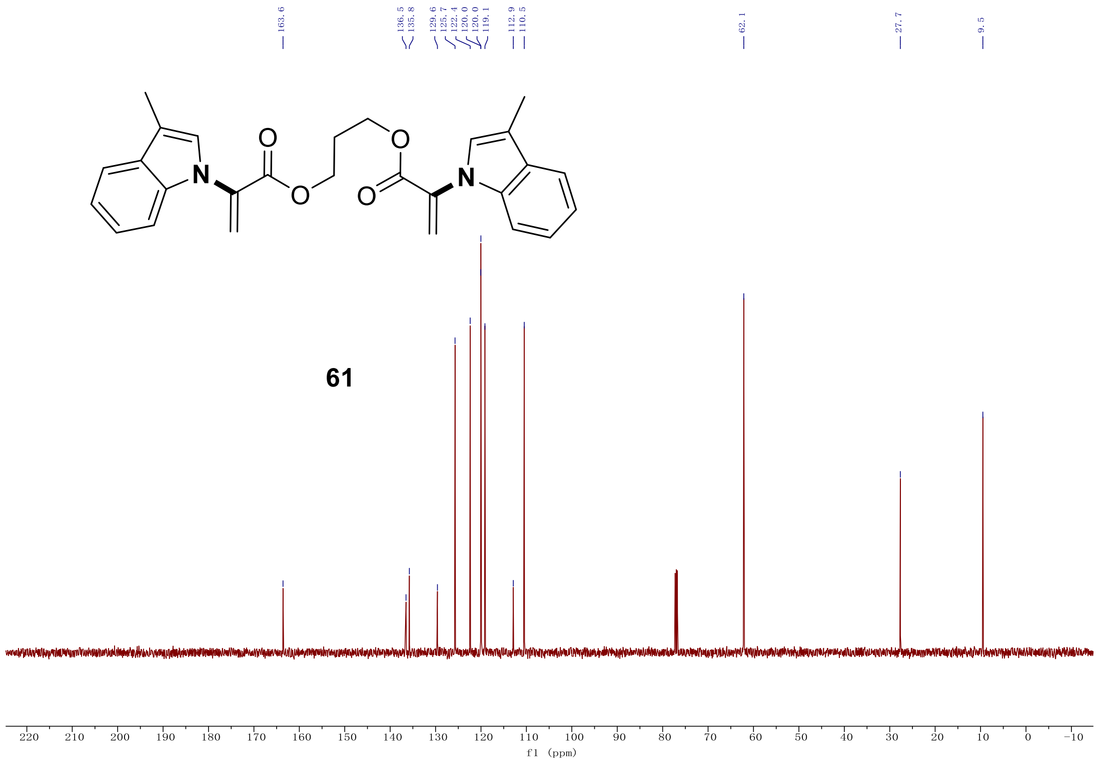

^{13}C NMR spectrum ($CDCl_3$, 126 MHz) of (**61**)

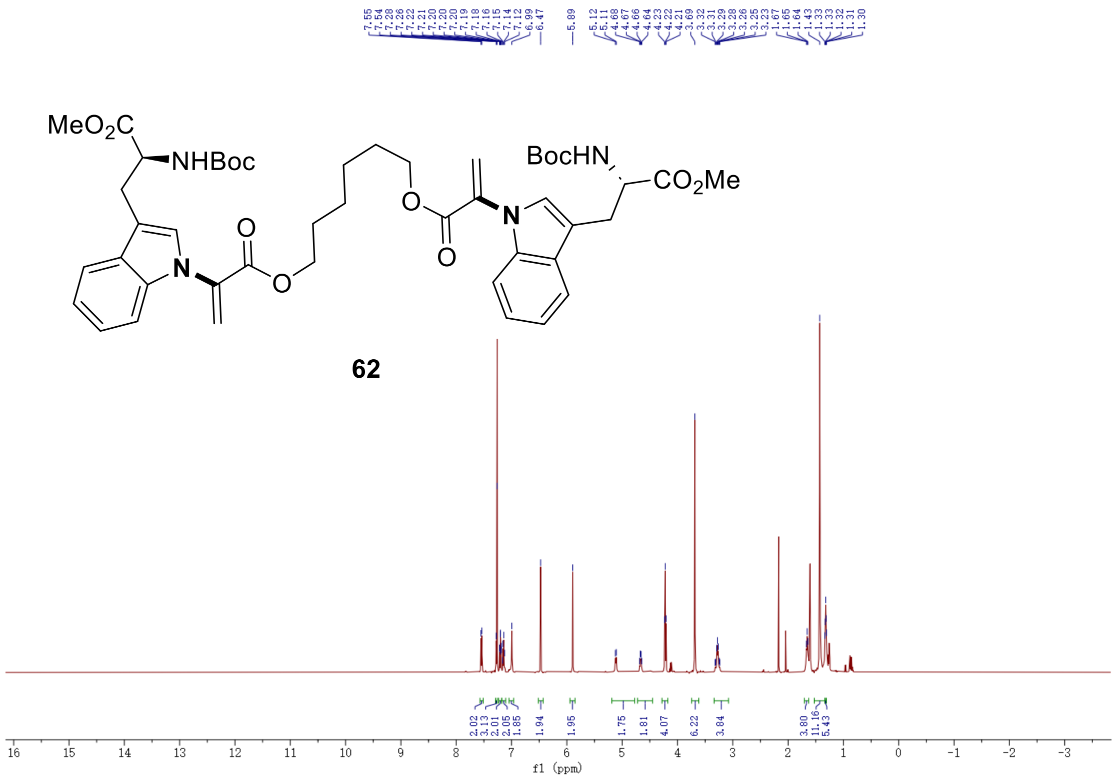

^{1}H NMR spectrum ($CDCl_3$, 500 MHz) of (**62**)

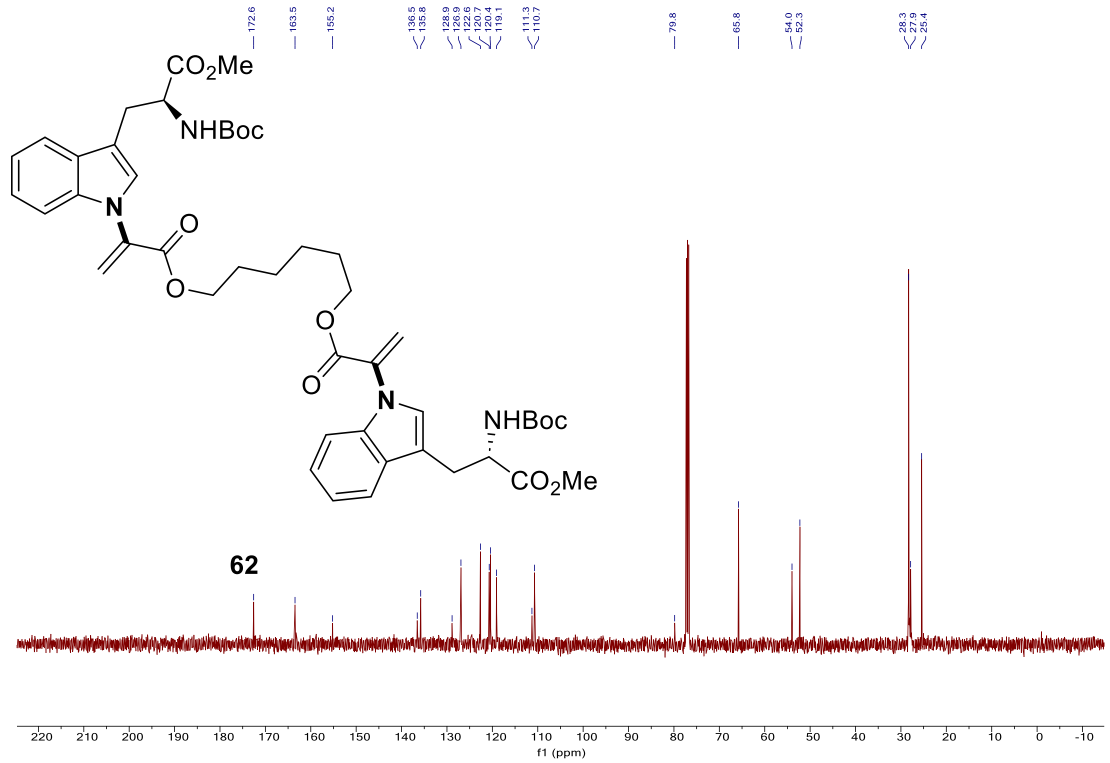

^{13}C NMR spectrum ($CDCl_3$, 126 MHz) of (**62**)

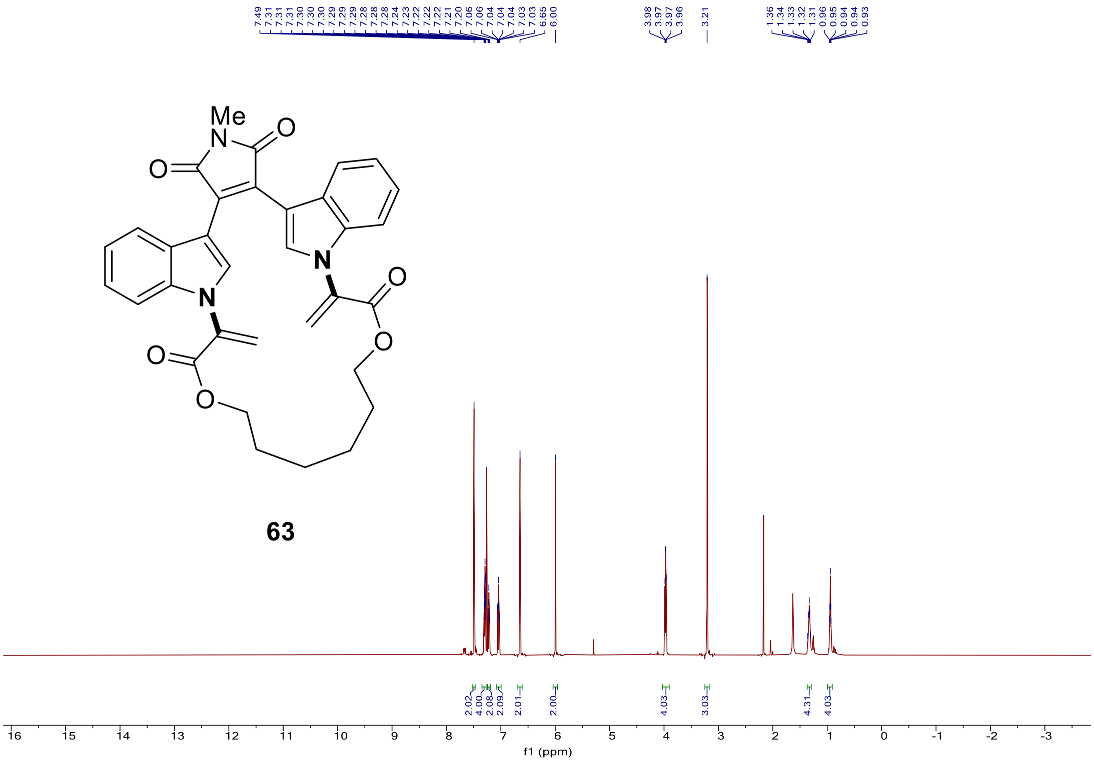

^{1}H NMR spectrum ($CDCl_3$, 500 MHz) of (**63**)

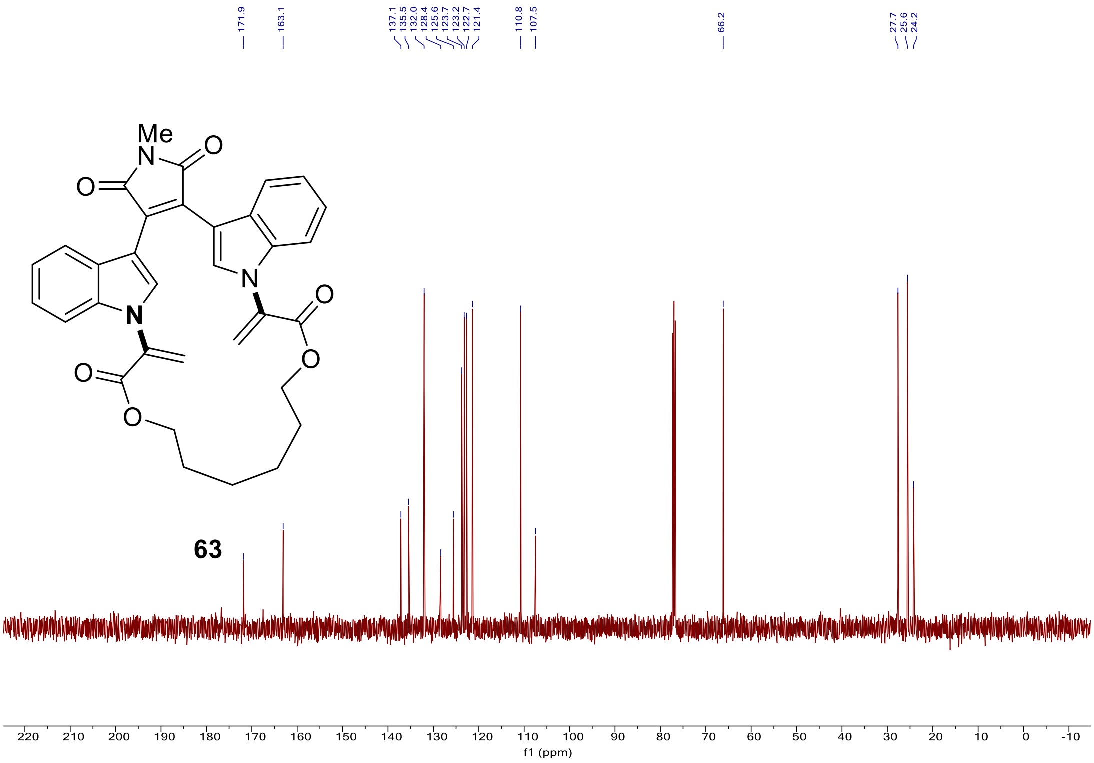

^{13}C NMR spectrum ($CDCl_3$, 126 MHz) of (**63**)

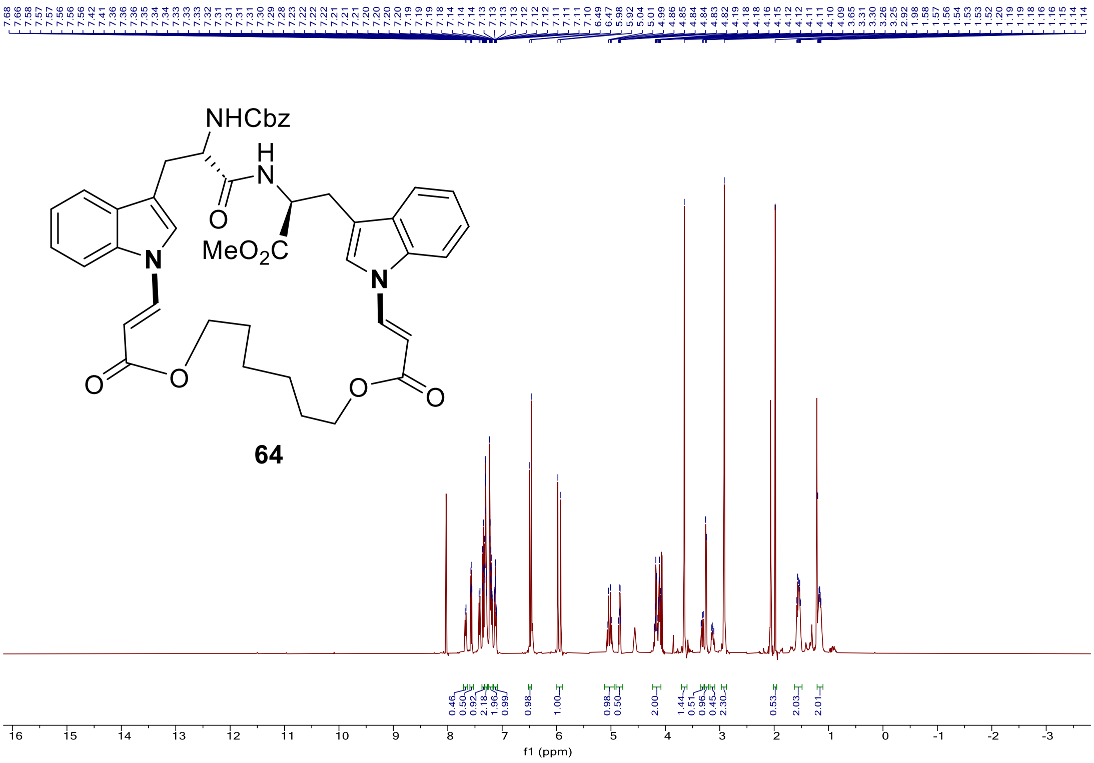

^{1}H NMR spectrum (Acetone-d_6, 500 MHz) of (**64**)

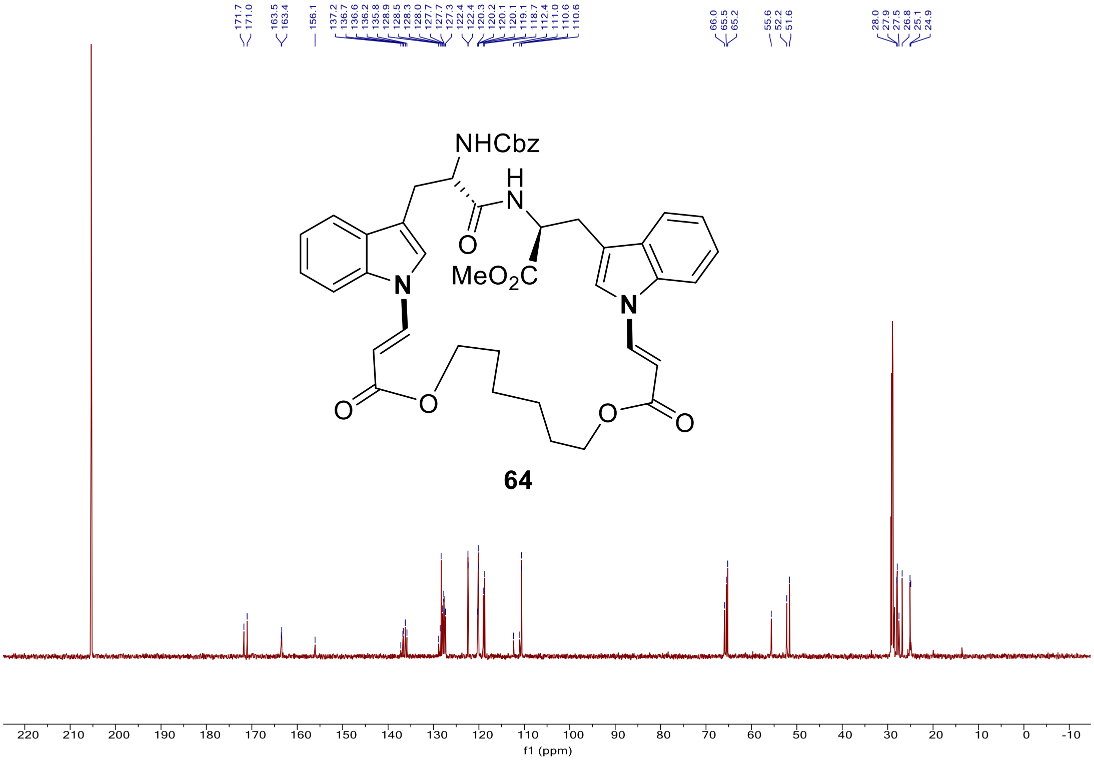

^{13}C NMR spectrum (Acetone-d_6, 126 MHz) of (**64**)

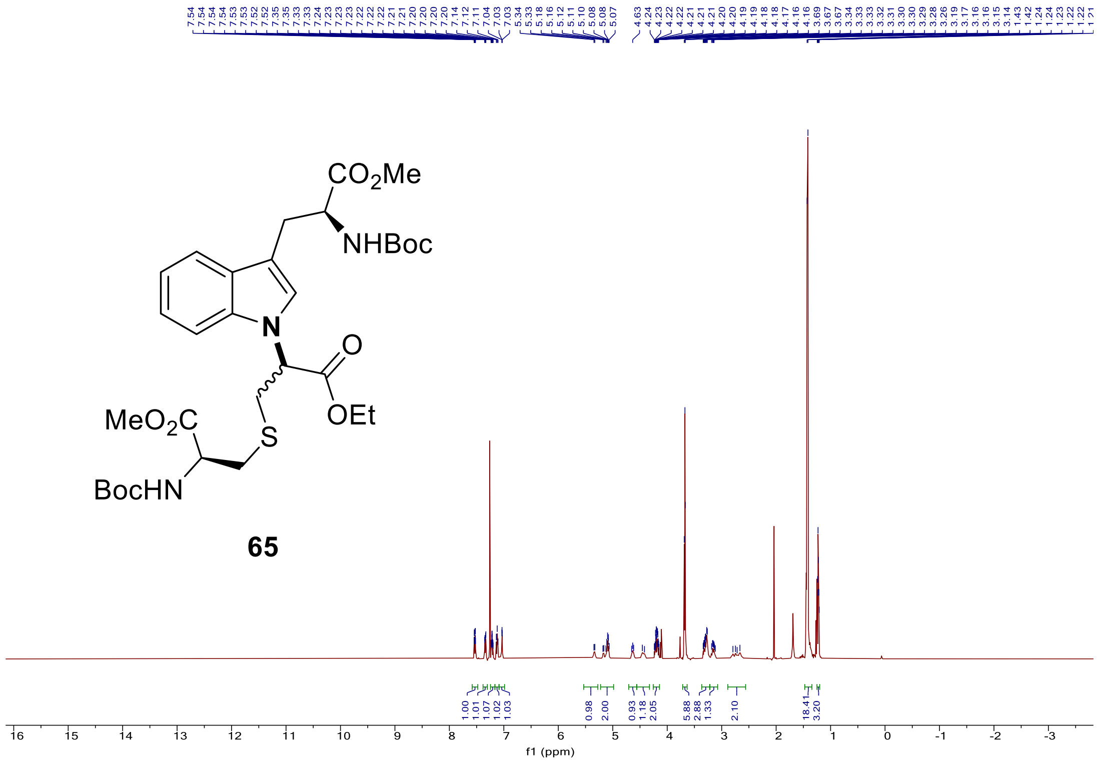

^{1}H NMR spectrum ($CDCl_3$, 500 MHz) of (**65**)

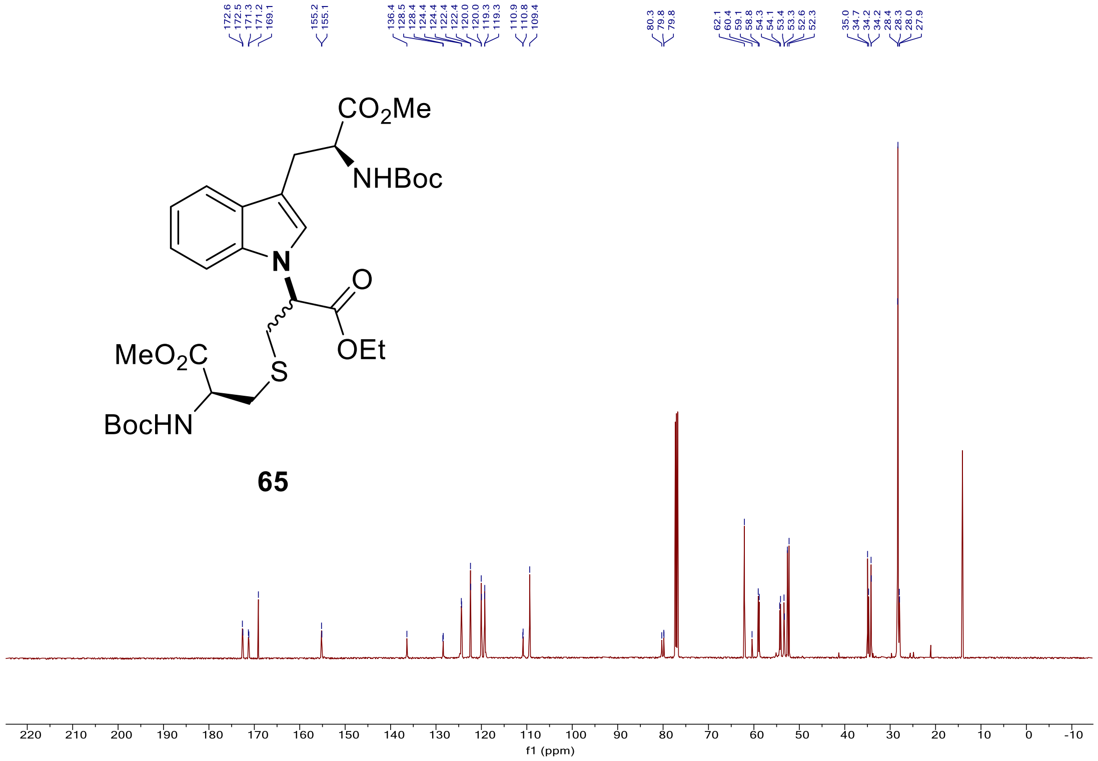

^{13}C NMR spectrum ($CDCl_3$, 126 MHz) of (**65**)

References

1. M.J. Frisch, G.W. Trucks, H.B. Schlegel, G.E. Scuseria, M.A. Robb, J.R. Cheeseman, et al., Gaussian 09, Revision D.01 ed., Gaussian Inc., Wallingford, CT, 2013.
2. Y. Zhao, D.G. Truhlar, The M06 suite of density functionals for main group thermochemistry, thermochemical kinetics, noncovalent interactions, excited states, and transition elements: two new functionals and systematic testing of four M06-class functionals and 12 other functionals. *Theor. Chem. Acc.* **2008**, *120,* 215–241.
3. A.V. Marenich, C.J. Cramer, D.G. Truhlar, Universal Solvation Model Based on Solute Electron Density and on a Continuum Model of the Solvent Defined by the Bulk Dielectric Constant and Atomic Surface Tensions. *J. Phys. Chem. B.* **2009**, *113*, 6378–6396.
4. C.Y. Legault, CYLView 10b, Universite de Sherbrooke,Sherbrooke, Quebec, Canada, (2009).
5. T. Lu, F. Chen, Multiwfn: a multifunctional wavefunction analyzer. *J. Comput. Chem.* **2012**, *33*, 580–592.
6. E.R. Johnson, S. Keinan, P. Mori-Sánchez, J. Contreras-García, A.J. Cohen, W. Yang, Revealing Noncovalent Interactions. *J. Am. Chem. Soc.* **2010**, *132*, 6498–6506.
7. W. Humphrey, A. Dalke, K. Schulten, VMD: Visual molecular dynamics. *J. Mol. Graph.* **1996**, *14,* 33–38.
8. Yavari, I.; Norouzi-Arasi, H. Triphenylphosphine-Catalyzed Nucleophilic α-Addition to Alkyl Propiolates: Synthesis of α-Substituted Alkyl Acrylates. *Phosphorus, Sulfur, Silicon Relat. Elem.* **2002**, *177*, 87–92; **2010**, *177*, 87
